# Novel functional microRNAs from virus-free and infected *Vitis vinifera* plants under water stress

**DOI:** 10.1038/srep20167

**Published:** 2016-02-02

**Authors:** Vitantonio Pantaleo, Marco Vitali, Paolo Boccacci, Laura Miozzi, Danila Cuozzo, Walter Chitarra, Franco Mannini, Claudio Lovisolo, Giorgio Gambino

**Affiliations:** 1 Institute for Sustainable Plant Protection, National Research Council (IPSP-CNR), Research Unit of Bari. Via Amendola 165/a, 70126 Bari, Italy; 2 Department of Agricultural, Forest and Food Sciences, University of Torino. Largo P. Braccini 2, 10095 Grugliasco-TO, Italy; 3 Institute for Sustainable Plant Protection, National Research Council (IPSP-CNR), Grugliasco Unit. Largo P. Braccini 2, 10095 Grugliasco-TO, Italy; 4 Institute for Sustainable Plant Protection, National Research Council (IPSP-CNR), Torino. Strada delle Cacce 73, 10135 Torino, Italy

## Abstract

MicroRNAs (miRNAs) are small non-coding RNAs that regulate the post-transcriptional control of several pathway intermediates, thus playing pivotal roles in plant growth, development and response to biotic and abiotic stresses. In recent years, the grapevine genome release, small(s)-RNAseq and degradome-RNAseq together has allowed the discovery and characterisation of many miRNA species, thus rendering the discovery of additional miRNAs difficult and uncertain. Taking advantage of the miRNA responsiveness to stresses and the availability of virus-free *Vitis vinifera* plants and those infected only by a latent virus, we have analysed grapevines subjected to drought in greenhouse conditions. The sRNA-seq and other sequence-specific molecular analyses have allowed us to characterise conserved miRNA expression profiles in association with specific eco-physiological parameters. In addition, we here report 12 novel grapevine-specific miRNA candidates and describe their expression profile. We show that latent viral infection can influence the miRNA profiles of *V. vinifera* in response to drought. Moreover, study of eco-physiological parameters showed that photosynthetic rate, stomatal conductance and hydraulic resistance to water transport were significantly influenced by drought and viral infection. Although no unequivocal cause–effect explanation could be attributed to each miRNA target, their contribution to the drought response is discussed.

RNA silencing is a conserved mechanism of gene regulation that acts both at the transcriptional level through DNA methylation and at the post-transcriptional level through direct mRNA targeting and cleavage mediated by small(s) RNAs^1^. In plants and invertebrates, the same pathways also function directly in host defence against viruses by targeting viral RNA for degradation^2^. Many 20–24 nt endogenous sRNAs serve as regulatory molecules and two major categories are recognised to date, based on the nature of the RNA precursors: micro(mi)RNAs and endogenous short interfering RNAs (siRNAs), which are processed from stem–loop regions of single-stranded primary RNAs or from double-stranded RNAs, respectively^3^.

The miRNA precursors are transcribed by RNA polymerase II into long primary transcripts, termed pri-microRNAs^4^. The release of the mature miRNA requires two subsequent cuts: the first removes the unpaired ends of the pri-miRNA and releases the pre-miRNA^5^, then, the pre-miRNA is processed by DICER-LIKE 1 protein (DCL1) to release mature miRNA duplexes in the nucleus^6^. The miRNA duplex is incorporated into the RNA-induced silencing complex (RISC) core protein ARGONAUTE (AGO), mainly AGO1, and the miRNA star strand (miRNA*) is degraded^7^,^8^. The miRNA guide strand is usually more abundant than the miRNA* and guides the RISC to the mRNA target in a sequence-specific manner. Growing evidence has suggested that miRNA* also can be loaded into AGO proteins and inhibit gene expression^9^. miRNAs are widely recognised as key players in plant development, tissue differentiation, flowering time and many other important physiological processes, including plant adaptation to biotic and abiotic stresses^10^,^11^.

The phytohormone abscisic acid (ABA) plays a pivotal role in responses to various abiotic and biotic stresses. Nearly 10% of *Arabidopsis* genes are responsive to and are modulated by ABA via the presence of ABA responsive elements (ABRE) in their promoters^12^; ABRE elements have been described in several miRNA genes^13^,^14^,^15^, including in the promoter of miR168^16^. Notably, miR168 is the regulator of AGO1 expression through homeostasis mechanisms^17^.

In addition to drought, pri-miRNA, pre-miRNA and mature miRNA accumulation is clearly influenced by plant viruses through numerous mechanisms and various approaches have been used to reveal such effects^18^.

Plant miRNA genes were initially identified in the model species *Arabidopsis thaliana* and *Oryza sativa* using a combination of computational prediction tools and experimental approaches^10^,^19^,^20^,^21^. In the last decade, these methods have been overtaken by small RNA sequencing using next-generation DNA sequencing technologies (NGS). Moreover, with the recent release of fruit-tree genomes, the application of NGS has considerably extended miRNA discovery beyond a few economically important plants. Among these species, *Vitis vinifera* has become a model for the genome-wide discovery of novel miRNA genes, their targets and functions in various steps of the vegetative and reproductive phases. To date, 163 grapevine miRNA precursor and 186 mature miRNA sequences are registered in the miRBase 2.0, which represent 47 different miRNA families^22^,^23^,^24^,^25^.

Taking advantage of the availability of *Vitis vinifera* plants that are free from viruses, which might have impaired the discovery and characterisation of novel grapevine-specific miRNA in previous studies, here we propose an experimental design that can dissect the effects of drought on grapevine under controlled conditions in the greenhouse. Plants were monitored for eco-physiological parameters and sRNA-seq and other sequence-specific molecular analyses allowed us to characterise the conserved miRNA expression profile in association with severe water stress conditions. Moreover, for the first time, sRNA-seq analysis has been carried out in the absence of the *Grapevine rupestris stem pitting associated virus* (GRSPaV), a very widespread latent grapevine virus. This virus is characterised by wide genetic diversity and it is generally associated with “Rupestris Stem Pitting”, a disorder of the “Rugose Wood complex”^26^. However, GRSPaV is usually found in *V. vinifera* cultivars in a latent state^26^.

Here, we show that GRSPaV alters the expression profile of several miRNAs, including those involved in adaptation to water stress, and can modify the eco-physiological parameters of infected plants. Although no unequivocal explanation was found for these phenomena, the interactions between GRSPaV and drought are discussed here.

## Results

### Deep sequencing of sRNAs from grapevine under well-watered and drought conditions

**Figure 1:**
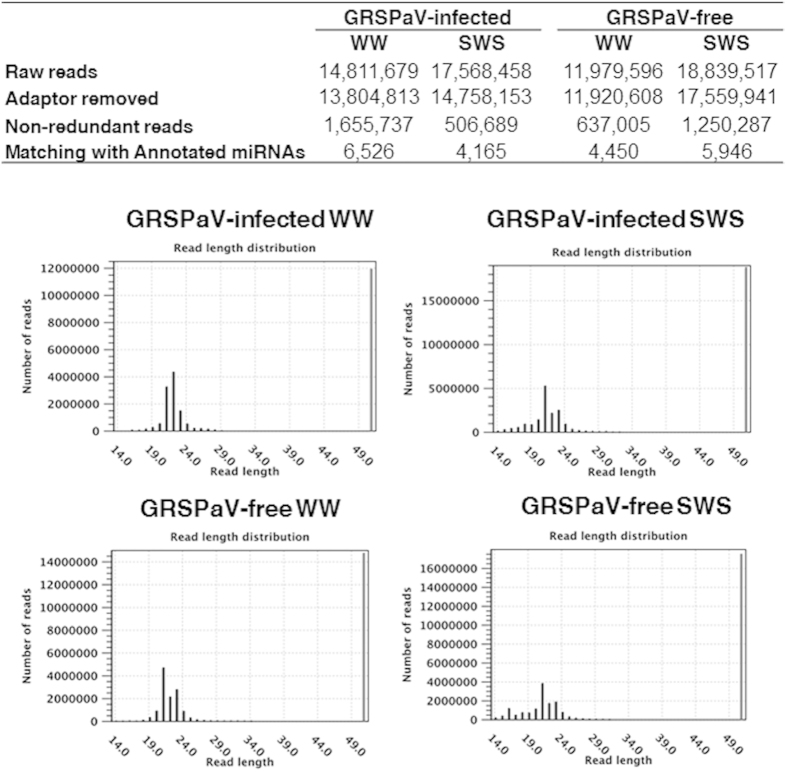
Statistics of short RNA sequences from *Vitis vinifera* ‘Bosco’ leaves collected from infected and *Grapevine rupestris stem pitting-associated virus*(GRSPaV)-free plants under well watered (WW) and severe water stress (SWS) conditions.

To identify drought-regulated miRNAs in virus-free and GRSPaV-infected (‘infected plants’ hereafter) grapevine, plants of *V. vinifera* ‘Bosco’ were subjected to water stress under controlled greenhouse conditions. Leaves from six GRSPaV-free and six infected plants were collected following two imposed stress regimes: well-watered (WW) and severe water stress (SWS; stomatal conductance, g_s_ ≈ 25 mmol H_2_O m^−2^ s^−1^; stem potential, Ψstem ≈ −1.5MPa) (Supplementary Fig. S1). Small endogenous RNAs with 5′-phosphate and 3′-OH groups were isolated from leaves and were used to generate cDNA libraries for Illumina sequencing (data are available in the GEO under the series entry GSE63244, http://www.ncbi.nlm.nih.gov/geo/query/acc.cgi ? token = uzmrqceonlyzxqb&acc = GSE63244). The metrics of the four libraries are shown in Fig. 1. The abundance of the 21-nt class of sRNAs was higher than that of 24-nt sRNAs in all four libraries. The analysis of virus-derived small-interfering RNAs (vsiRNAs) confirmed that no known viruses were present in the GRSPaV-free plants and only GRSPaV (GRSPaV-1 sequence variant^27^) was present in the infected grapevines. In WW conditions, the GRSPaV genome (NCBI # AY368590) was represented by 1,591 vsiRNA reads covering 50% of the reference genome, whereas in SWS, 2,470 vsiRNA reads, covering 51% of the viral reference genome were found. The presence/absence of the virus was confirmed by quantitative real-time RT-PCR (qRT-PCR) on each starting plant. The GRSPaV titre did not change significantly in drought conditions, and no amplification signal was obtained from virus-free plants (Supplementary Fig. S2).

### Conserved families of miRNAs

Conserved families of miRNAs were identified by aligning the sRNA reads of the four libraries with sequences from the miRBase^28^ dataset. The normalised numbers of reads found in each library are shown in Table 1. As expected, we identified members of almost all known miRNAs. The expression profiles of several miRNAs varied considerably among the libraries and a selection of these were validated by qRT-PCR. In the following sections, we discuss only the results for known miRNAs that were validated by qRT-PCR. In addition, qRT-PCR of miRNAs was performed on leaves in water-stress conditions (WS; g_s _≈ 60 mmol H_2_O m^−2^ s^−1^; Ψstem ≈ −1MPa). For each miRNA and housekeeping genes (5.8S rRNA and U6), the melting curves from the PCR amplification showed a unique peak, and the amplification products showed the correct product sizes in gel electrophoresis and the expected sequence (Supplementary Fig. S3). These data allowed us to exclude non-specific pre-miRNA amplification. The qRT-PCR results generally confirmed the sequencing data; however, in a few cases (miR2950, miR3624, miR3631, miR3633, miR3634, miR3637), we observed some discrepancies (Table 1, Supplementary Fig. S4).

**Figure 2:**
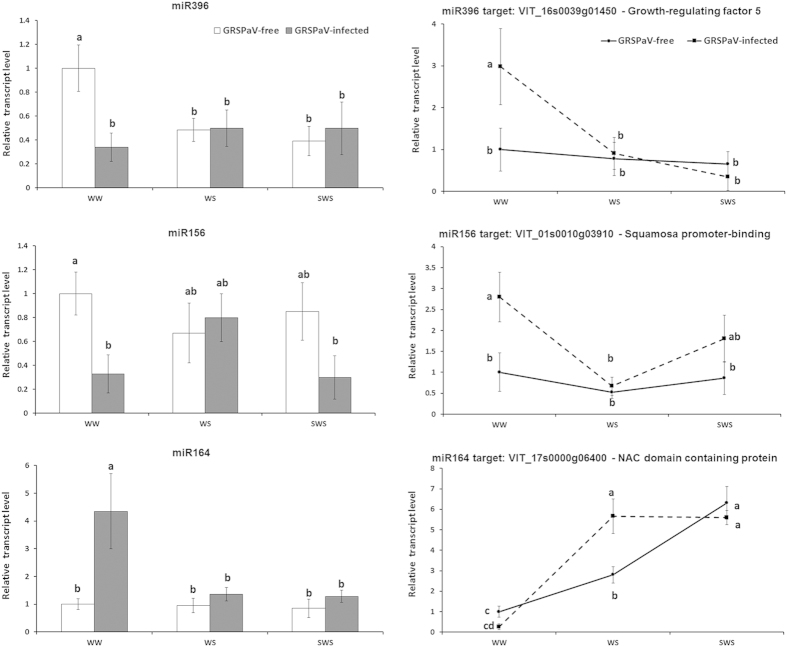
Relative expression levels of miR396, miR156, and miR164 and their respective targets in *Grapevine rupestris stem pitting-associated virus*(GRSPaV)-free and infected ‘Bosco’ leaves as determined by qRT-PCR. Samples were collected under well-watered (WW), water stress (WS) and severe water stress (SWS) conditions. qRT-PCR signals were normalised to U6 and 5.8 rRNA for miRNA quantification, and to actin and ubiquitin transcripts for target quantification. Data are presented as mean ± standard deviation of three biological replicates; different letters denote significant differences at *p* ≤ 0.05.

In infected plants, miR166, miR319, miR396, miR3631, mi3633 and miR3639 were down-regulated in SWS, whereas miR168, miR482, miR535, miR2111, miR3624 and miR3634 were up-regulated (Fig. 2, Supplementary Fig. S4). Thus, the latent viral infection considerably affected the accumulation of many known miRNAs (Table 1). Indeed, in WW conditions, most conserved miRNAs that were validated by qRT-PCR (miR164, miR168, miR172, miR319, miR390, miR394, miR2111, miR3624, miR3629, miR3639) were expressed at higher levels in infected plants than in GRSPaV-free plants (Fig. 2, Supplementary Fig. S4). Notably, most differences in miRNA accumulation between infected and GRSPaV-free plants disappeared in SWS conditions and significant differences in expression remained only for miR166, miR319, miR390, and miR3639 (Supplementary Fig. S4). Our data suggest that the virus-induced changes in miRNA expression between WW and SWS conditions; in particular, the presence of GRSPaV, significantly affected the accumulation of miR156, miR164, miR166, miR394, miR396, and miR3639 in response to SWS (Fig. 2, Supplementary Fig. S4) and the relevance of this is discussed later.

### Identification of novel miRNA candidates

Novel miRNA candidates were identified using the sRNA datasets as inputs for the mircat tool (http://srna-workbench.cmp.uea.ac.uk/tools/analysis-tools/mircat/) and using the latest release of the *V. vinifera* genome as a reference (http://www.genoscope.cns.fr/externe/GenomeBrowser/Vitis/). Based on hairpin predictions, we selected 12 miRNA candidates that were potentially generated from 17 loci (Table 2, Supplementary Fig. S5). Furthermore, we found the miRNA* strands for five out of the 12 new miRNAs (Table 2, Supplementary Fig. S5).

By searching miRBase, we observed that some novel miRNA candidates (four out of 12) were similar to described miRNAs from other plant species (Supplementary Fig. S6): miRC104 was highly similar to a sequence from *Manihot esculenta* and *Populus trichocarpa* and miRC108 contained a seed sequence (position 5–16) that was identical to *Solanum tuberosum* miR7982a and b. Furthermore, the miRC129 sequence was highly similar to miR7122a from *Malus domestica* and to miR7122 from *S. tuberosum* (from position 2–19 and from 1–21, respectively) and since we identified the presence of the miRNA*, we suggest that it be annotated as the *V. vinifera* homologue of miR7122 (Supplementary Fig. S6). miRC128 originated from the stem loop of the vvi-miR3659, thus suggesting the presence of a multifunctional stem loop^29^. Notably, we previously described a sRNA that was similar to miRC128, however, this was considered to be a secondary product of the stem loop, due to a partially overlapping sequence with the miR3659-5p^24^.

**Figure 3:**
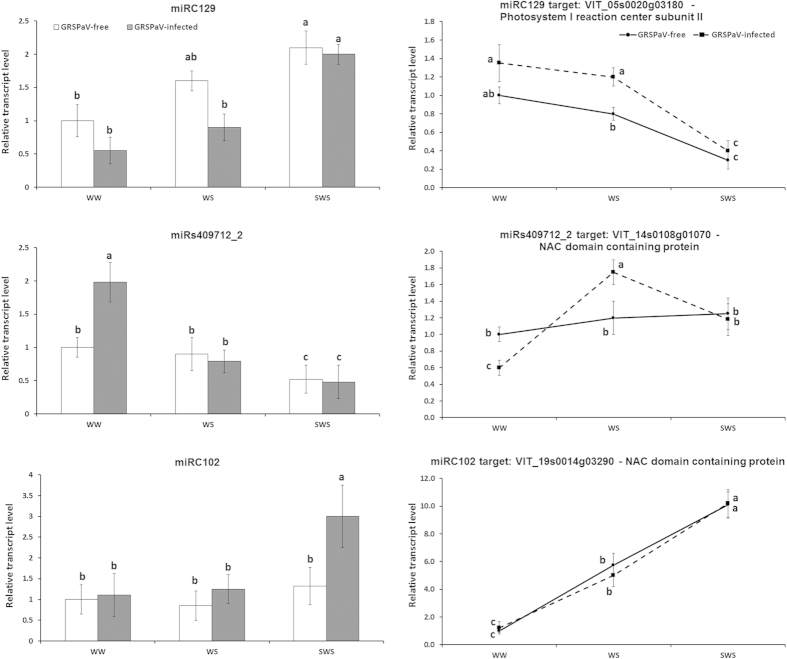
Relative expression levels of miRC102, miRC129 and miRs409712_2 and their respective targets in *Grapevine rupestris stem pitting-associated virus*(GRSPaV)-free and infected ‘Bosco’ leaves as determined by qRT-PCR. Samples were collected under well-watered (WW), water stress (WS) and severe water stress (SWS) conditions. qRT-PCR signals were normalised to U6 and 5.8 rRNA for miRNA quantification, and to actin and ubiquitin transcripts for target quantification. Data are presented as mean ± standard deviation of three biological replicates; different letters denote significant differences at *p* ≤ 0.05.

The expression levels of all novel miRNA candidates were validated by qRT-PCR and the specificity of amplification was confirmed by melting-curve analysis, gel electrophoresis and sequencing as reported above for conserved miRNAs (Supplementary Fig. S3). The qRT-PCR amplification was very sensitive and all miRNAs were amplified from all samples, even from those that had not been retrieved by sequencing analysis (Table 3). In virus-free grapevines, miRC116 and miRs409712_2 were down-regulated in SWS, whereas miRC104, miRC128 and miRC129 were up-regulated by drought (Fig. 3, Supplementary Fig. S7). Similar to the known miRNAs, the expression profiles of some novel miRNAs also differed between infected and GRSPaV-free plants. Notably, miRC122 expression was barely affected by drought, but expression in infected plants was at least two-fold higher than in GRSPaV-free grapevines in all three sampling conditions (WW, WS and SWS). The responses of miRC108 and miRC129 to drought were independent of the virus and were induced by SWS in both infected and GRSPaV-free plants, although in different ways (Fig. 3, Supplementary Fig. S6).

### Targets of novel grapevine miRNAs

**Figure 4:**
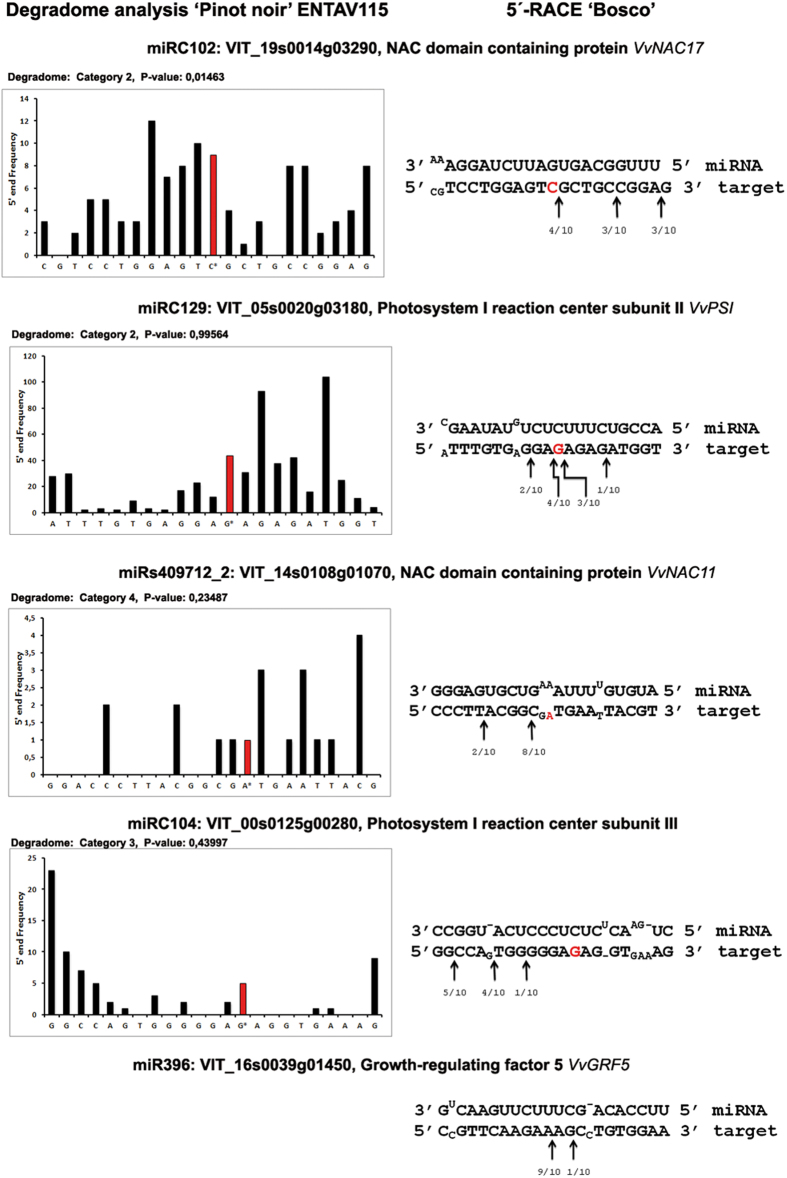
Comparison between degradome analyses of ‘Pinot noir’ ENTAV115 and 5′-RACE in ‘Bosco’ for the identification of miRNA-mediated cleavage of target transcripts. In red, the cleavage site provided by the degradome, arrows indicate the 5′-end of the transcript degradation fragments mapped by 5′-RACE. Fractions indicate the number of RACE clones with 5′-end mapping at a determined position with respect to the total number of sequenced clones. For miR396 the target gene VIT_16s0039g01450 was predicted by bioinformatics and confirmed by 5′-RACE.

The novel miRNA candidates described here were not identified by our previous genome-wide analysis in ‘Pinot noir’ ENTAV115 carried out using stems and leaves^24^ (GEO accession No. GSE18405). However, taking advantage of the higher sensitivity of qRT-PCR (see above), we explored the expression of these novel miRNAs in leaves from one ENTAV115 plant conserved in our collection in more detail. As shown in Supplementary Fig. S8, all novel miRNAs were detected by qRT-PCR in this clone. We therefore concluded that novel miRNAs escaped detection in the previous genome-wide sequence analysis and we then took advantage of the availability of the degradome library from the same plant to search for their mRNA targets^24^. We applied the Cleaveland pipeline (see methods) to search for targeted transcripts from the degradome library of ENTAV115 (GEO accession No. GSE18406) and the abundance of the sequenced tags was plotted for each transcript (Supplementary Fig. S9). The cleaved transcripts were categorised into five classes (0, 1, 2, 3 and 4). Out of the 12 new miRNAs, we identified potential targets for 10 new miRNAs; four with the star strand (miRC106, miRC116, miRC123 and miRC129) and six without it (Supplementary Table S1). Many potential targets were linked to photosynthesis (genes linked to Photosystem; miRC129) and stress (NAC domain-containing protein; miRC102, miRs409712_2) (Supplementary Table S1), two categories of genes that are widely known to be linked to drought. Selected targets were transferred to our experimental design by confirming their presence in ‘Bosco’ through 5′-rapid amplification of cDNA ends (5′-RACE). The target of miRC102 (VIT_19s0014g03290, a NAC domain-containing protein, named *VvNAC17*^30^) with a *p*-value ≤ 0.05 was considered highly reliable by CleaveLand analysis, and indeed, was confirmed in ‘Bosco’ by 5′-RACE (Fig. 4). In addition, three putative targets with a *p*-value > 0.05 were analysed in ‘Bosco’, and for two of these – VIT_05s0020g03180 (PSI reaction centre subunit II, *VvPSI* target of miRC129) and VIT_14s0108g01070 (a NAC domain-containing protein, named VvNAC11, a target of miRs409712_2^30^) −5′-RACE confirmed the degradome approach (Fig. 4).

### Eco-physiological parameters of grapevine in drought conditions

The changes in the accumulation of miRNAs and their targets in the transition from WW and SWS conditions might result in specific traits of plant physiological adaptation to drought stress that are also influenced by the viral infection. Thus, plants subjected to water stress under controlled greenhouse conditions were monitored daily for several eco-physiological parameters.

**Figure 5:**
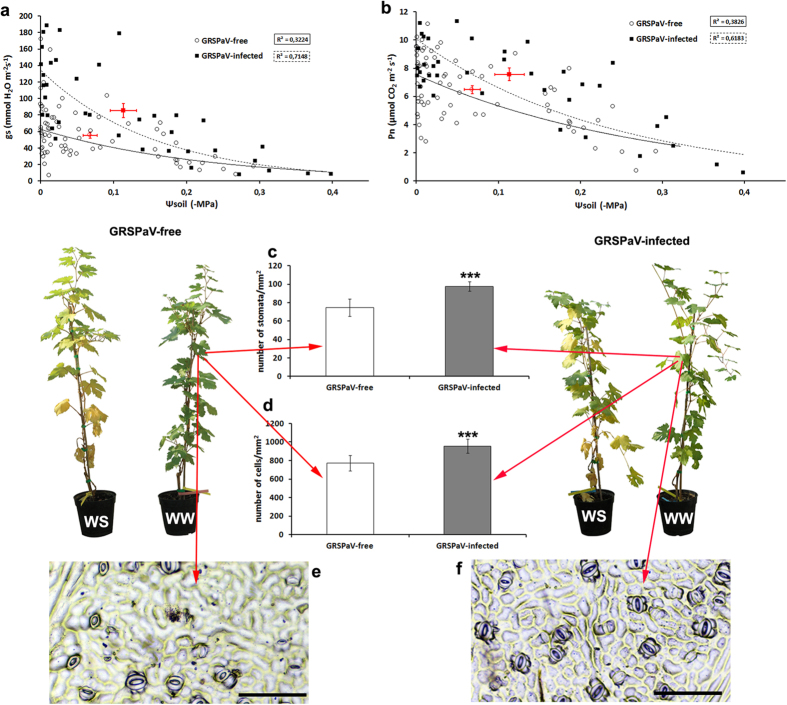
Relationship between (**a**) the stomatal conductance (g_s_) and soil water potential (Ψsoil) and between (**b**) net photosynthesis (P_n_) and Ψsoil in *Grapevine rupestris stem pitting-associated virus*(GRSPaV)-infected (filled squares, dashed trend line) and GRSPaV-free plants (open circles, continuous trend line). **(c**) Number of stomata and (**d**) number of cells per square millimetre in leaves from infected and GRSPaV-free grapevines in well-watered (WW) conditions. Bars represent the mean ± standard error (n = 72); asterisks indicate significant difference among the means (*p* ≤ 0.01). Abaxial prints of epidermis from (**e**) GRSPaV-free and (**f**) GRSPaV-infected leaves. Scale: 100 mm. In panels (**a**,**b**), averages and standard error bars calculated for GRSPaV-free and GRSPaV-infected plants are indicated in red.

During the progressive drying of soil, the stomatal conductance (g_s_) of both sets of plants decreased in response to water stress; however, this response was delayed in infected plants. The stomata of GRSPaV-free plants were less regulated throughout the whole experiment, as observed by gas exchange measurements (Fig. 5a) or by losses in pot weight (Supplementary Fig. S10a). In addition, infected grapevines developed leaves with a significantly higher stomatal density (*p* ≤ 0.05) and more cells than GRSPaV-free plants (Fig. 5c–f). The photosynthetic rate (P_n_) was also higher in the infected plants over time (Fig. 5b). The maximum fluorescence (F_m_) in dark-adapted leaves showed no significant difference between treatments (F_0_; Supplementary Fig. S10b). Conversely, responses to water stress revealed by a decrease in variable fluorescence (F_v_/F_m_), electron transfer rate (ETR) and PS II quantum yield were observed at lower values of soil water potential (Ψsoil) in infected plants (Supplementary Fig. S10c–e). Mechanisms to protect the photosystems from photo-oxidation, such as effects on non-photochemical quenching (NPQ; Supplementary Fig. S10f), coupled with differences in P_n_, were also revealed at lower values of Ψsoil in infected plants compared to virus-free plants (Supplementary Fig. S10f). No differences were observed in the CO_2_ transport pathway, at the level of sub-stomatal internal carbon (c_i_) (Supplementary Fig. S10g) and mesophyll conductance to CO_2_ over time (gmes; Supplementary Fig. S10h).

**Figure 6:**
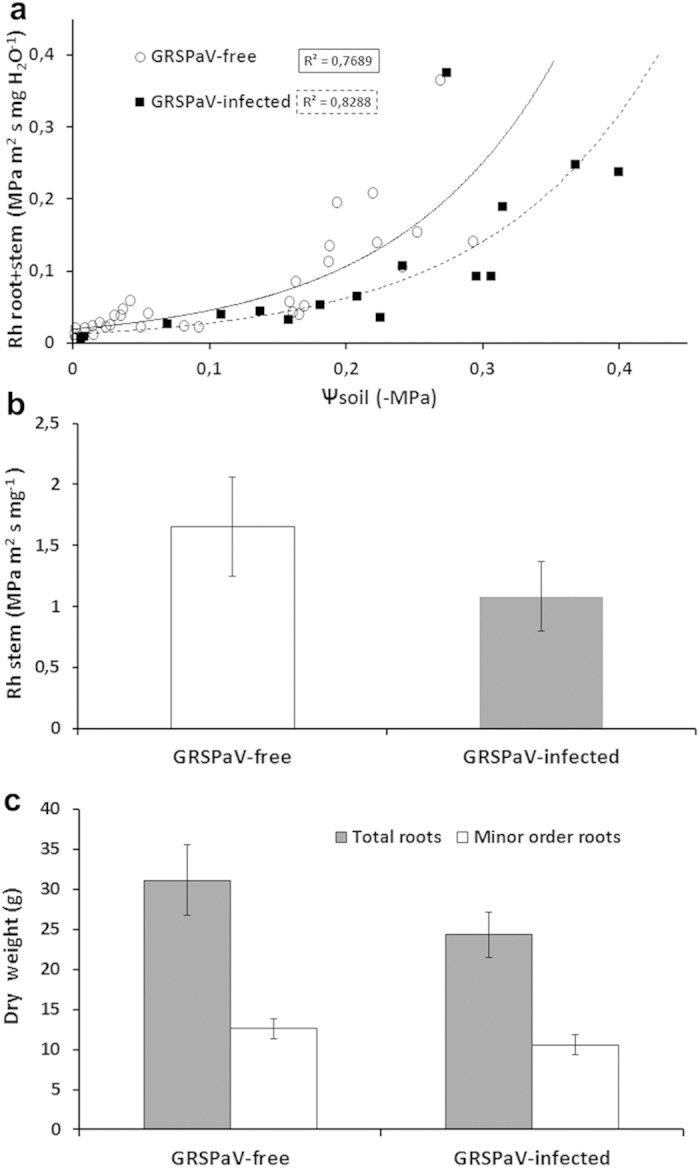
(**a**) Relationship between the specific root + shoot hydraulic resistance (Rh_root + shoot_) relative to leaf surface area and soil water potential (Ψsoil) in *Grapevine rupestris stem pitting-associated virus*(GRSPaV)-infected (filled squares, dashed trend line) and GRSPaV-free plants (open circles, continuous trend line). (**b**) Specific stem hydraulic resistance (Rh _stem_) relative to stem cross section area. (**c**) Dry weight of whole roots (filled columns) and minor order roots (empty columns) in GRSPaV-free and GRSPaV-infected plants. Bars represent the mean ± standard error (n = 6); different letters denote significant differences at *p* ≤ 0.05.

The root + shoot hydraulic resistance (Rh_root + stem_) increased inversely to the decrease in Ψsoil in both treatments; however, in infected plants, this occurred at a lower water potential (i.e., higher water stress) than in virus-free plants (Fig. 6a). By dissecting any single component of water transport, we observed that the specific stem hydraulic resistance (Rh_stem_) showed a lower resistance (Rh) in infected plants, although this was not statistically significant compared to that non-infected plants (Fig. 6b), and no differences were observed in root dry weight (Fig. 6c).

### Expression of target genes involved in photosynthesis and drought tolerance

From the study of the eco-physiological parameters, we conclude that leaf development/stomata density, P_n_ and g_s_ were significantly influenced by drought and viral infection. Therefore, the expression of selected target genes of miRNAs involved in these physiological pathways was analysed using qRT-PCR.

*Growth-regulating factor 5* (*VvGRF5*, VIT_16s0039g01450) is a target of miR396, and was previously predicted by an bioinformatic approach and was confirmed in ‘Bosco’ by 5′-RACE analysis (Fig. 4). This gene was more highly expressed in infected leaves in the WW treatment, than in uninfected leaves, showing a negative correlation with the expression of miR396 (Fig. 2, left-upper panel). No change in the expression of either *VvGRF5* or miR396 was observed in the WS and SWS treatments (Fig. 2, right-upper panel).

The *Squamosa promoter-binding protein* gene (*VvSBP*, VIT_01s0010g03910) is a member of a plant-specific transcription factor family with a broad range of functions, and was previously demonstrated to be a target of miR156^24^. A negative correlation was found between miR156 and *VvSBP* expression in WW and SWS conditions. However, the expression of both genes was influenced only by GRSPaV infection and not by drought (Fig. 2, middle panels).

The *VvPSI* gene (VIT_05s0020g03180) is a target of novel miRC129 and has been previously annotated as a component of the subunit II of the Photosystem I^31^. We showed that *VvPSI* expression was higher in infected plants in WW and WS conditions. Moreover, in infected and GRSPaV-free plants, the gene was down-regulated in SWS and showed a clear negative correlation with the expression of miRC129, which was highly expressed in SWS (Fig. 3, upper panels).

Evidence for the multiple regulation of target genes by drought has been shown for three NAC domain-containing protein targets of several miRNAs. The *VvNAC05* (VIT_17s0000g06400^30^) gene is a target of miR164^24^ and showed a negative correlation with the expression of miR164 in the WW treatment (Fig. 2). In response to drought, the expression of *VvNAC05* increased more rapidly in infected grapevines than in GRSPaV-free plants. In infected plants, we observed a significant increase in the target expression level in the WS treatment, whereas a comparable increase in virus-free plants was observed only in response to higher water stress (SWS condition) (Fig. 2, right-lower panel). No clear correlation was observed between miR164 and its target gene expression in WS and SWS conditions.

We observed a clear negative responsiveness of miR s409712_2 to drought conditions (Fig. 3 left, middle panel). This effect was particularly clear following the transition from WW to WS for GRSPaV-infected plants (grey vs. white bars). The combination *VvNAC11* (VIT_14s0108g01070)/miR s409712_2 showed the same regulation as *VvNAC5*/miR164, even though the increased expression of this *NAC* gene in response to WS was not as great as that observed for *VvNAC05* (Fig. 3 vs. Fig. 2).

Finally, we observed a more gradual transcriptional activation under WS and SWS conditions for *VvNAC17*, a target of miRC102, no substantial differences between GRSPaV-free and infected plants were observed (Fig. 3, lower panels) and no clear correlation with miRC102 expression was observed.

## Discussion

The experimental strategy adopted in this study was designed to investigate the profile of grapevine miRNAs in response to drought and virus infection. Furthermore, the simultaneous application of these two different types of stresses provided new information regarding the regulation of miRNAs in the interaction between biotic and abiotic stresses and this is discussed below.

Almost all known miRNAs^24^ were found in sRNA datasets from grapevine leaves; in addition, 12 novel miRNA candidates, some of which were particularly influenced by drought and/or virus infection, were identified. The miRNA profiles measured by qRT-PCR generally confirmed the sequencing data; only in a few cases were substantial discrepancies observed. Similar inconsistencies were previously reported for some miRNAs when high-throughput sequencing and northern blot analyses were compared, for example, for miR3633 and some other conserved and grapevine-specific miRNAs^24^. Bias in the production and sequencing of sRNA libraries has been reported in previous studies^32^. Some discrepancies were also observed for novel miRNA candidates (miRC122 and miRC129); however, this comparison was difficult due to the very low number of reads obtained by sequencing.

The miRNA candidates miRC104 and miRs409712_2 have previously been identified in grapevines by other authors^33^,^34^. In addition, they were amplified by qRT-PCR and the targets were identified. Although we could not identify the miRNA*, these data suggest that they should be tentatively evaluated as novel grapevine-specific miRNAs.

The described novel miRNA candidates were also revealed by qRT-PCR in the ‘Pinot Noir’ cultivar in previous studies, thus enabling the use of the published degradome library^24^. This approach helped us to identify novel targets that were further confirmed by 5′-RACE analyses in ‘Bosco’ leaves (Fig. 4). This demonstrates that degradome analysis provides robust and stable data for different varieties of the same species and in some cases, can effectively exploit the libraries already published.

In response to water stress, several miRNAs, including those from conserved families are differentially expressed in different plant species^35^,^36^. For example, in GRSPaV-free grapevines, miR166 and miR396 were down-regulated by drought, similar to their reported expression in *O. Sativa*^37^; miR168 was up-regulated in *Arabidopsis thaliana*, in agreement with its response to ABA-inducing stresses^16^ and miR156, miR167 and miR397 were not affected by drought in grapevine, thus confirming their species-specific drought response^35^,^38^. Indeed, the variability in miRNA regulation reported in response to drought is widely considered to be linked to species-specific physiological strategies^36^. However, this study clearly shows that the presence of the latent virus constitutes an additional player in the species-specific miRNA regulation during drought stress.

Plants infected with GRSPaV showed a higher g_s_ in all treatments, which was closely related to the higher stomatal density observed in infected plants (Fig. 5). Several environmental stimuli (light, CO_2_ and soil temperature) that affect stomatal density and size have been analysed^39^,^40^. However, in the experimental design here, plants were grown in the same environmental conditions from budburst, confirming that these differences can be ascribed to the presence or absence of GRSPaV.

Notably, the infected plants maintained higher levels of carbon assimilation than the GRSPaV-free plants during progressive soil drying. This difference in the decrease in P_n_ was coupled to F_v_/F_m_, ETR, and to the quantum yield of PSII in light adapted condition. This behaviour can be explained by the scheme proposed by Medrano *et al.*^41^, which suggests that a decrease in P_n_ is reflected by a reduction in ETR when g_s_ falls below 100 mmol H_2_O m^−2^ s^−1^. No increase in the F_m_ in F_0_ was recorded in infected plants, which suggests that an irreversible inactivation of the PSII reaction centre does not occur in infected grapevines. Therefore, no damage to the photosystems occurred, in agreement with the poor susceptibility to photo-oxidation indicated by Flexas *et al.*^42^ for grapevines. In addition, the carbon-transport capacity analysed by measuring the c_i_ and gmes showed no differences between infected and GRSPaV-free plants. These results indicate that no change in the photochemistry reaction occurred in infected and GRSPaV-free plants and that carbon metabolism was influenced only by higher g_s_, which allowed a higher P_n_.

Water transport was addressed by analysing the organ(s) in which metabolism could be altered in infected plants. The Rh_root + stem_ value was lower, regardless of the values of Ψsoil in infected plants, and consequently, major effects on water transport could be hypothesised. This is supported by the Rh_stem_, which was half the value in infected plants than in GRSPaV-free plants, although the difference was not statistically significant (Fig. 6b). Considering the roots, infected plants have a lower resistance with respect to ‘root-to-shoot’ water transport, despite possessing a similar root mass, considering either whole roots or root hairs (Fig. 6c). Therefore, infected plants should possess a root system that is suitable for absorbing and transporting water from the soil, since a higher osmotic adjustment capacity occurs during water stress.

Together, these results suggest some hypothetical interactions between miRNAs and the physiological changes induced in grapevine by this virus under drought. For example, in infected grapevines, we observed that the regulation of some miRNAs and their targets involved in leaf development was influenced by virus infection (miR156, miR164, miR319, miR394, miR396; Fig. 2, Supplementary Fig. S4).

In *Arabidopsis*, the overexpression of miR396 and repression of its targets (*Growth-regulating factors*), result in leaves with fewer cells, a lower stomatal density and an increased drought tolerance^43^,^44^. Indeed, a higher stomatal density in *Arabidopsis* was shown to be negatively correlated with drought resistance^45^,^46^. Notably, we observed higher levels of miR396, a decrease in the transcript level of *VvGRF5* and a reduction in both stomata and cell numbers only in GRSPaV-free grapevines in the WW treatment (Figs 2 and 5). This apparent contradiction can be explained since drought resistance in *Arabidopsis* is related to the negative control of stomatal function to avoid water losses from plants, whereas grapevines often tolerate (and do not avoid) water deprivation by increasing water uptake from the soil^47^. The higher tolerance to water stress in infected grapevines as shown by physiological data (Figs 5 and 6) reinforces this explanation. In addition, the stomatal patchiness of grapevine leaves can benefit from redundancy in the system when stomatal regulation occurs^48^. In *Arabidopsis*, Usami *et al.*^49^ demonstrated that miR156 and *SBP* genes are involved in determining leaf cell number and size; overexpression of *SBP* genes with a deletion in the miR156 target site leads to an increase in cell number. Accordingly, we observed a similar regulation in response to an increase in *VvSBP* expression and low miR156 levels only in leaves of infected plants. In addition, the accumulation of *VvPSI*/miRC129 transcripts might be positively correlated with the higher levels of photosynthesis observed in infected plants (Figs 3 and 5b). However, further experiments are necessary to confirm these intriguing hypotheses.

The NAC proteins play important roles in abiotic stress responses in several species^50^. *NAC* genes are generally up-regulated in grapevines during water stress^51^,^52^; they respond to ABA and represent a node between various signalling pathways^50^,^52^,^53^. In our experiments, we observed that *VvNAC05, VvNAC11* and *VvNAC17* (targets of miR164, miRs409712_2 and miRC102, respectively) were induced by water stress. Furthermore, infected plants appear to be more responsive to water stress; *VvNAC05*and *VvNAC11* were induced more rapidly than in GRSPaV-free grapevines. The multiple levels of regulation that control the transcription of these *NAC* genes (post-transcriptionally by miRNAs and transcriptionally probably by ABA) induced a rapid response to drought in infected plants that might be positively linked to drought tolerance.

The interaction between biotic (virus) and abiotic (drought) stresses in grapevine induced some relevant changes in miRNA accumulation. Furthermore, the general sanitary conditions should be carefully considered when analysing sRNA profiles in plants, in particular for perennial woody species that are vegetatively propagated. In addition, considering the high level of heterozygosity of *V. vinifera*, the modulation of some miRNAs in response to drought might vary in different cultivars, rootstock–scion combinations or in the same cultivar infected by different pathogens.

The interaction between GRSPaV and *V. Vinifera* cv. Bosco appears to improve the drought tolerance of infected grapevines in greenhouse conditions. It would be relevant to further explore and confirm this in field conditions, using different cultivars or symptomatic *Vitis* spp. and considering the high molecular variability of GRSPaV, with different strains of this virus.

## Methods

### Plant material

The study was performed on the Italian white grape cultivar Bosco (*V. vinifera* L.) using self-rooted plants grown in pots in a greenhouse. Woody material was collected in the field from previously identified GRSPaV-infected and GRSPaV-free plants^54^. All ‘Bosco’ plants were derived from vegetative propagation from a single mother plant originally infected by GRSPaV and were further subjected to sanitation.

Six GRSPaV-free and six infected plants were monitored during water deprivation by daily measurements of leaf gas-exchange parameters and Ψstem. Following the progressive decrease in eco-physiological performance, leaves for molecular analyses (siRNA libraries and qRT-PCR) were collected based of three selected levels of stress: well-watered (WW), water stress (WS: stomatal conductance, g_s _≈ 60 mmol H_2_O m^−2^ s^−1^; Ψstem ≈ −1 MPa) and severe water stress (SWS: g_s_ ≈ 25 mmol H_2_O m^−2^ s^−1^; Ψstem ≈ −1.5 MPa) (Supplementary Fig. S1), according to the three drought classes previously reported by Medrano *et al.*^41^. In addition, the pot weight was recorded daily at 18:00 to assess Ψsoil by calculating the difference between two consecutive weights.

### Small RNA library construction and analysis

Low-molecular-weight RNA was extracted^55^ from a pool of leaves (two mature leaves for each plant) collected from six infected and six GRSPaV-free grapevines in WW and SWS conditions. Libraries of sRNAs were then created using a TrueSeq Small RNA Sample Kit (Illumina, San Diego, CA, USA) and were sequenced using the HISeq 2500 Illumina platform. All data were processed by first converting FASTQ to FASTA format, and then removing 3′-adaptor sequences using the CLC Genomics Workbench software (Aarhus, Denmark). Viral vsiRNAs were retrieved from the library with the ‘vsiRNA extractor pipeline’ using the universal Illumina adapter sequence during the trimming step^56^. The miRNA predictions were performed using mircat^57^,^58^. The detection of similarities between novel miRNA candidates and annotated miRNAs was performed using BLAST against the miRBase database^59^.

### Targets of novel miRNAs

The identification of grapevine transcripts targeted by miRNAs was first performed using CleaveLand 4.3^60^ (http://sites.psu.edu/axtell/). Input datasets consisted of the previously published Degradome analysis of ‘Pinot Noir’^24^, 12X grapevine V1 gene prediction (http://genomes.cribi.unipd.it/DATA/V1/) from the grapevine reference genome PN40024, a near homozygous ‘Pinot noir’ genotype^61^, and all novel miRNA candidates identified in ‘Bosco’. Only cleavage sites with a *p*-value ≤ 0.05 were considered statistically significant. Each cleavage site was categorised according to the system implemented in CleaveLand 4.3. The degradation fragments resulting from miRNA cleavage were analysed in ‘Bosco’ plants by 5´-RACE, as described in the Supplementary Methods. For each targets identified, the annotation of V1 version of the 12X grapevine genome (http://genomes.cribi.unipd.it/DATA/V1/) was integrated by Gene Ontology (GO) classifications using Blast2GO tool (https://www.blast2go.com/).

### qRT-PCR analysis

For qRT-PCR analysis, were analysed three independent biological replicates. The quantification of miRNA expression by qRT-PCR was carried out following the protocol of Shi and Chiang^62^, with some modifications reported in the Supplementary Methods. The specificity of amplification was analysed by: i) the dissociation kinetics performed at the end of each PCR run; ii) the PCR product sizes by electrophoresis using a 5% agarose gel; and iii) cloning and sequencing the PCR products. For target and GRSPaV quantification, the primers reported in Supplementary Table S2 and the previously published qRT-PCR protocol were used^54^.

### Drought experiment

The analysis of physiological performance under drought was carried out on six GRSPaV-free and six infected plants. Transpiration rate (E) and g_s_ were measured via pot weight, and using the GFS-3000 portable gas-exchange fluorescence system (Walz Heinz GmbH, Effeltrich, Germany) as well as by measuring P_n_ and sub-stomatal internal carbon (c_i_) (see Supplementary Methods). Furthermore, chlorophyll fluorescence parameters were recorded for one leaf (previously dark-adapted) per plant, taken at approximately 12:00, with a light intensity set at 1,200 μmol photon m^−2^ s^−1^, 400 p.p.m [CO_2_] and a cuvette temperature of 25 °C.

The midday Ψstem was measured for one embedded leaf per plant using a Scholander-type pressure chamber (Soil Moisture Equipment Corp., Santa Barbara, CA, USA) as previously described by Begg and Turner^63^ and Chone *et al.*^64^. The Ψsoil was calculated following the equation obtained by pressure-plate analysis^65^ (see Supplementary Methods).

The estimation of the Rh_root + shoot_ was performed following the evaporative flux method reported by Sack *et al.*^66^, modified by applying the following formula: Rh_root + shoot_ = (Ψstem–Ψsoil)/E. The Rh_stem_ was measured using a High-Pressure Flow Meter (Dynamax, Houston, TX, USA) apparatus on stems collected from plants subjected to water stress at the end of the drought treatment by a transient-pressure method, following the manufacturer’s instructions.

The enumeration of stomata and cells was determined on two leaves per six plants for infected and GRSPaV-free plants, and the dry weight of the roots was quantified at the end of the experiments (see Supplementary Methods).

### Statistical analyses

Data management and calculations were performed using a Microsoft EXCEL spreadsheet. Significant differences among treatments for molecular and physiological data were statistically analysed by applying a one-way ANOVA test and Tukey’s post hoc test was used for mean separation when ANOVA results were significant (*p* ≤ 0.05). The SPSS statistical software package (SPSS Inc., Cary, NC, USA, v.22) was used to perform statistical analyses.

## Additional Information

**How to cite this article**: Pantaleo, V. *et al.* Novel functional microRNAs from virus-free and infected *Vitis vinifera* plants under water stress. *Sci. Rep.* **6**, 20167; doi: 10.1038/srep20167 (2016).

## Supplementary Material

Supplementary Information

Supplementary Table S1

Supplementary Table S2

## Figures and Tables

**Table 1:** Known miRNAs in GRSPaV-infected and GRSPaV-free grapevine in well watered (WW) and severe water stress (SWS) conditions.

miRNA	Relative number of miRNA reads			
	GRSPaV-infected		GRSPaV-free	
	WW	SWS	WW	SWS
miR156	494.3	6421.6	17263.2	5624.3
miR159	3088.7	2096.0	3228.2	2526.3
miR160	4.1	2.2	3.7	1.7
miR162	522.8	1362.8	1038.5	1458.7
miR164	2.1	0.7	0.9	0.7
miR166	139871.5	134525.5	152237.3	104632.1
miR167	92.2	110.2	126.2	128.5
miR168	147.7	208.3	150.0	231.2
miR169	7.7	8.1	7.2	7.1
miR171	10.5	35.9	38.0	26.7
miR172	2.7	0.6	0.7	0.3
miR319	191.6	16.7	49.7	17.2
miR390	8.8	22.8	18.6	20.2
miR393	20.8	9.1	16.4	11.5
miR394	5.8	1.3	1.4	1.3
miR395	9.5	15.9	20.2	13.7
miR396	6470.5	2309.2	3357.9	2194.0
miR398	47598.4	12173.0	19426.5	11528.8
miR399	4.7	6.2	6.3	5.4
miR408	2608.6	5326.7	8597.2	5193.0
miR479	3.8	3.1	3.3	3.1
miR535	19.0	79.0	66.5	68.3
miR397	224.1	78.8	199.8	89.6
miR398	47579.9	12166.5	19411.8	11521.1
miR399	0.5	1.2	1.2	1.8
miR403	185.2	456.1	1063.0	516.0
miR477	1.6	16.4	8.7	21.2
miR482	109.2	693.5	531.6	585.1
miR828	2.5	1.4	2.6	0.5
miR845	2.1	1.2	1.4	2.9
miR2111	2.4	1.6	1.3	2.2
miR3623	265.4	615.9	835.2	637.9
miR2950	10.1	4.7	7.6	151.7
miR3624	395.6	1042.0	1932.7	1052.7
miR3625	0.6	1.3	0.9	1.8
miR3626	8.2	13.4	11.9	14.2
miR3627	32.1	146.2	175.9	151.1
miR3628	—	0.8	0.1	—
miR3629	1.4	0.2	0.6	3.1
miR3630	2.3	3.2	7.2	2.4
miR3631	9.7	7.7	2.0	5.8
miR3632	2.3	2.7	5.1	3.1
miR3633	19.9	120.6	1.3	105.2
miR3634	81479.8	93669.5	130903.2	77805.7
miR3635	0.3	0.1	0.1	—
miR3636	29.2	10.4	11.5	10.8
miR3637	2.6	4.9	8.0	4.0
miR3638	0.3	0.2	0.1	0.1
miR3639	124.3	43.9	78.7	40.3
miR3640	5.2	8.4	10.0	9.0

The relative number of reads was obtained by dividing the number of reads of each miRNA with the total number of reads of each cDNA library.

**Table 2:** Novel putative grapevine miRNAs.

	Chromosome	Start	End	Orientation	Abundance (infected WW, SWS and free WW and SWS)	Sequence (5′-3′)	sRNA length (nt)	# Genomic Hits	Hairpin Length	Minimum Free Energy	miRNA* (5′-3′) (aboundance in GRSPaV-infected WW, SWS and GRSPaV-free WW and SWS)
mirC102	chr12	22422891	22422911	—	4, 10, 4, 0	TTTGGCAGTGATTCTAGGAAA	21	3	118	−35,4	NO
	chr19	19300144	19300164	—	4, 10, 4, 0	TTTGGCAGTGATTCTAGGAAA	21	3	119	−42,7	NO
miRC103	chr7	15865768	15865789	+	28, 41, 35, 32	AAGTGTTTCTGGGCTTTATAAC	22	2	75	−26	NO
miRC104	chr14	24560699	24560720	—	86, 71, 134, 41	CTGAACTCTCTCCCTCATGGCC	22	1	122	−65,4	NO
miRC106	chr16	18836667	18836687	+	29, 79, 146, 71	CTTTTGTTTCTTCGTGTCGGT	21	2	125	−66,82	CGACACGAAGAAACGAAAGCC(2,2,3,0) CGACACGAAGAAACGAAAGCCG (0, 0, 1, 0)
	chr16	18867640	18867660	+	29, 79, 146, 71	CTTTTGTTTCTTCGTGTCGGT	21	2	125	−66,82	
miRC108	chr19	10790715	10790735	—	0, 2, 0, 0	TACTTTGGATGATAATGGGCCT	21	1	158	−94,9	NO
miRC116	chr9	3184798	3184818	—	5, 63, 77, 46	TTCTTCTCACCGTTTCTCAGC	21	1	146	−48	TGAGAAACTGGGGGGAAAATG(0, 1, 2, 0) TTGCTGAGAAACTGGGGGGAA(0, 3, 0,0) TGAGAAACTGGGGGGAAAATGA (0, 2, 0,0)
miRC117	chr9	5984686	5984706	+	0, 11, 0, 0	TCTTAGAACCAAGGGCACCCT	21	6	88	−27	NO
	chr14	19624264	19624284	—	0, 11, 0, 0	TCTTAGAACCAAGGGCACCCT	21	6	92	−30,4	NO
miRC122	chr3	17925351	17925372	—	0, 0, 5, 0	TTATGGGACTACATGTGTTACA	22	1	151	−82,6	TACACATGTAGTGCCATCATA (0,0,5,1)
miRC123	chr14	14721702	14721722	—	48, 84, 47, 64	TACCAAGGTCGAGGATTGACT	21	1	207	−90,5	TCAAACCTCAACCTTGGTATG(1,1,1,0)
miRC128	chr9	21823140	21823161	+	0, 0, 0, 21	AGAAGTCAATCCAAACAAGGTC	22	2	90	−59	NO
miRC129	chr5	6820744	6820764	—	0, 31, 0, 13	ACCGTCTTTCTCTGTATAAGC	21	1	99	−49,64	TTACACAGAGAGATGACGGTGG (0,4,0,2)
miRs409712_2	chr10	4096605	4096626	—	0, 0, 0, 8	ATGTGTTTTAAAGTCGTGAGGG	22	5	91	−27,2	NO
	chr14	24304140	24304161	—	0, 0, 0, 8	ATGTGTTTTAAAGTCGTGAGGG	22	5	130	−70,7	NO
	chr19	20397809	20397830	+	0, 0, 0, 8	ATGTGTTTTAAAGTCGTGAGGG	22	5	206	−56,6	NO

**Table 3:** Novel grapevine miRNA candidates (miRC) abundance in GRSPaV-infected and GRSPaV-free grapevine in well watered (WW) and in severe water stress (SWS) conditions.

miRNA Candidate	Relative number of miRC reads			
	GRSPaV-infected		GRSPaV-free	
	WW	SWS	WW	SWS
miRC102	0.8	0.6	0.6	—
miRC103	5.6	2.5	5.6	5.4
miRC104	17.2	4.3	21.4	6.9
miRC106	5.8	4.7	23.4	12.0
miRC108	—	0.1	—	—
miRC116	1	3.8	12.3	7.8
miRC117	—	0.7	—	—
miRC122	—	—	0.8	—
miRC123	9.6	5.0	7.5	10.8
miRC128	—	—	—	3.5
miRC129	—	1.9	—	2.2
miRs409712_2	—	—	—	1.4

The relative number of reads was obtained by dividing the number of reads of each miRNA with the total number of reads of each cDNA library.
