# Supplementary material for: Novel functional microRNAs from virus-free and infected Vitis vinifera plants under water stress
Source: Sci Rep. 2016 Feb 2;6:20167. doi: 10.1038/srep20167 (PMC4735847; doi:10.1038/srep20167)
Supplement: Supplementary Information [file srep20167-s1.pdf]

**Novel functional microRNAs from virus-free and infected *Vitis vinifera* plants under water stress.**

Vitantonio Pantaleo, Marco Vitali, Paolo Boccacci, Laura Miozzi, Danila Cuozzo, Walter Chitarra, Franco Mannini, Claudio Lovisolo, Giorgio Gambino

## Supplementary Methods

*5'-RACE*

The degradation fragments resulting from miRNA cleavage were analysed by 5'-RACE. The RNA from free and GRSPaV-infected 'Bosco' leaves was extracted using the Spectrum Plant Total RNA Kit (Sigma-Aldrich, St. Louis, MO, USA), and was treated with DNase I (Life Technologies, Carlsbad, CA, USA), incubated with a RNA adaptor (Table S1) and 5U of T4 RNA ligase (Life Technologies) following the manufacturer's instructions. The RNA was reverse-transcribed using random nonamers (Sigma-Aldrich) and M-MLV reverse transcriptase (Life Technologies). First-round and nested PCR analyses were performed using a universal forward primer and gene-specific reverse primers (Supplementary Table S2). The nested products were gel-purified, cloned into the pGEM-T Easy vector (Promega, Madison, WI, USA), and ten independent clones from each product were sequenced using a Big-Dye Terminator v1.1 Cycle Sequencing kit (Applied Biosystems, Life Technologies), and analysed using a 3130 Genetic Analyser capillary sequencer (Applied Biosystems, Life Technologies).

*qRT-PCR analysis*

The quantification of miRNA expression by qRT-PCR was carried out following the protocol of Shi and Chiang (2005) with some modifications. Two μg of RNA extracted from GRSPaV-free or infected leaves collected under WW, WS and SWS conditions were treated with DNaseI (Life Technologies) and polyadenylated using the Poly(A) Tailing Kit (Life Technologies) following the manufacturer's instructions. The RNA was precipitated by ethanol and reverse-transcribed with M-MLV reverse transcriptase (Life Technologies) and 0.5 μg poly(T) adapter (Supplementary Table S2).

For the amplification of all miRNAs and housekeeping genes, a universal 3′-adapter reverse primer was used (Supplementary Table S2) and the forward primer was designed based on the specific miRNA sequence. For some primers containing high GC content at the 3′ end, one or two A residues were added to the 3′-end to facilitate binding to the target site (Supplementary Table S2).

The relative expression was calculated based on the comparative $C_t$ ($2^{-\Delta\Delta Ct}$) method as described by Livak and Schmittgen (2001). The PCR mix (10 μL) contained 5 μL PowerSYBR Green master mix (Applied Biosystems, Life Technologies), 0.25 μM of each primer and 1 μL of cDNA diluted 1:100 or 1:250, based on initial miRNA concentrations. Cycling conditions for all primer pairs consisted of initial denaturation at 95 °C for 10 min, followed by 45 cycles at 95 °C for 15 s, 58 °C for 20 s and 60 °C for 40 s. The 5.8S rRNA and U6, which have been extensively used with the grapevine and other species as reference in qRT-PCR and northern hybridisations for sRNA analysis, were used as housekeeping genes. The geometric mean of their expression ratios was used as the normalisation factor in all samples. Accumulation of miRNAs was expressed as the mean and standard deviation calculated for three biological replicate.

*Ecophysiological measurements*

Transpiration rates (E) and stomatal conductance ($g_s$) were measured by two approaches. Firstly, we estimated E and $g_s$ via pot weight. Daily E was calculated as the difference between two consecutive weights, and the daily grams of water loss per plant were converted into mmol $H_2O$ $m^{-2}$ $s^{-1}$. The $g_s$ was then calculated by dividing E by the environmental vapour-pressure deficit on the basis of the air temperature and relative humidity that was recorded every day during the experiment (Figure S2A). Secondly, leaf gas-exchange parameters ($P_n$, E, $g_s$, $c_i$) were measured by the GFS-3000 portable gas-exchange fluorescence system (Walz Heinz GmbH, Effeltrich, Germany) on two different mature leaves per plant, on each experimental day (setting: cuvette temperature 27 °C, [$CO_2$], photosynthetic photon flux density (PPFD) and Rh were those of the greenhouse environmental conditions).

The Ψsoil was calculated following the equation obtained by pressure-plate analysis (Richards and Ogata, 1961): $\Psi soil = 3e^{-10.9\theta}$, where $\theta$ represents the soil volumetric water content, calculated as described by Hochberg *et al.* (2013). The bulk density ($\rho$) of the peat substrate used was 0.383 kg $L^{-1}$.The weight of the sealed pots was measured at the end of each day (6 p.m.) using an electronic balance.

The enumeration of stomata and cells was determined on two leaves per six plants for infected and GRSPaV-free plants. Two leaf imprints were collected in the WW condition and three counts for each imprint were performed using an optical microscope with a 10-XT objective (Nikon Eclipse 55i, Tokyo, Japan).

At the end of the experiments, dry weight of the root was quantified by eradicating, washing and drying the roots in oven at 70°C until reaching of constant root weigh. Roots of minor orders were separated manually from main roots after drying.

**Supplementary References**

Livak, K.J. & Schmittgen, T,D. Analysis of relative gene expression data using real-time quantitative PCR and the $2^{-\Delta\Delta CT}$ method. *Methods* **25,** 402- 408 (2001).

Hochberg, U., Degu, A., Fait, A. & Rachmilevitch S. Near isohydric grapevine cultivar displays higher photosynthetic efficiency and photorespiration rates under drought stress as compared with near anisohydric grapevine cultivar. *Physiol. Plantarum* **147,** 443–452 (2013).

## Supplementary Figures

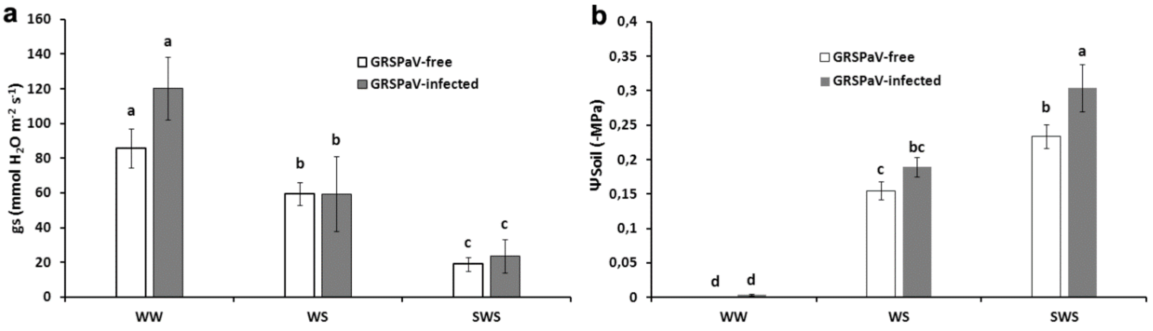


**Supplementary Figure S1**. a) stomatal conductance ($g_s$) measured and b) relative water stem potential (Ψstem) during the sampling time for small RNAs analysis. Bars represent the mean ± standard error (n=6). Different letters indicate significant difference among the means ($p \leq 0.05$). WW, well watered condition; WS, water stress condition; SWS severe water stress condition.

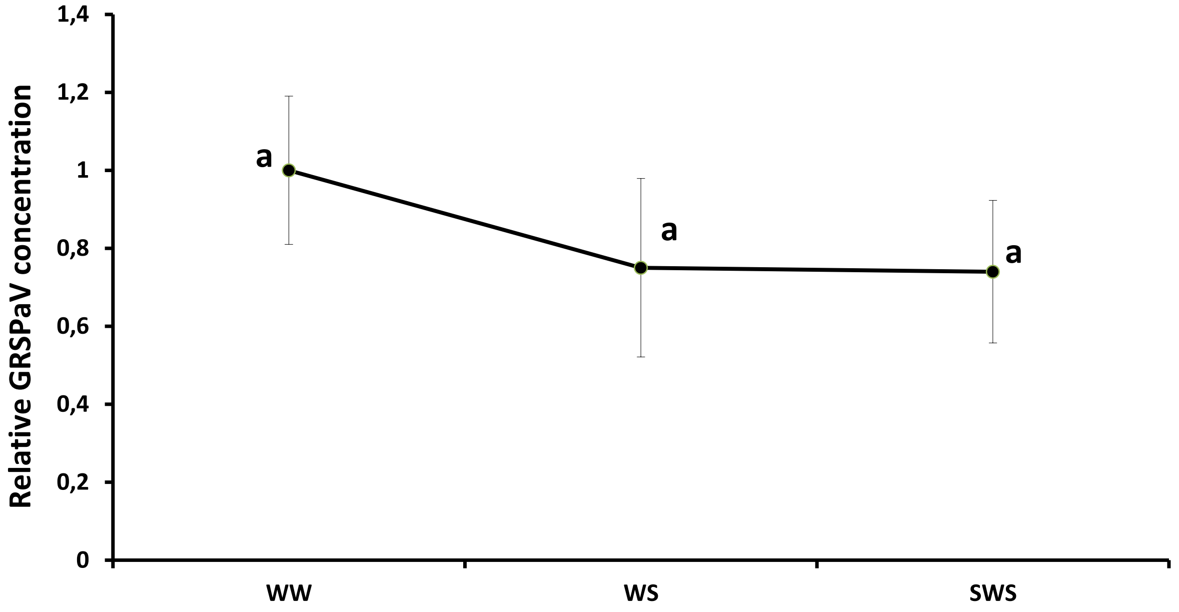


**Supplementary Figure S2**.Quantification of *Grapevine rupestris stem pitting-associated virus* (GRSPaV) RNA in 'Bosco' leaf as determined by qRT PCR. Samples were collected under well watered (WW), water stress (WS) and severe water stress (SWS) conditions. qRT-PCR signals were normalised to actin and ubiquitin levels. Data are presented as mean ± standard deviation of six plants and three technical replicates; different letters denote significant differences at $p \leq 0.05$.

**Supplementary Fig. S3:** Validation of qRT-PCR for quantitation of microRNAs (miRNAs). a) dissociation curves of qRT-PCR amplification and b) qRT-PCR products resolved on ethidium bromide-stained 5% agarose gel of all miRNAs and novel miRNA candidates analysed.

**a**

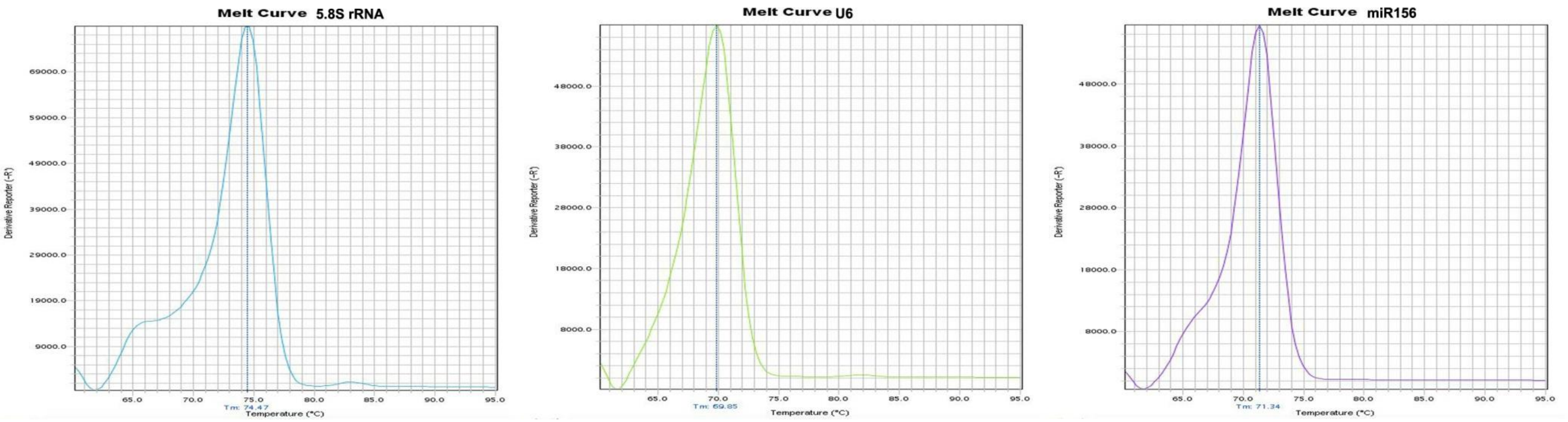

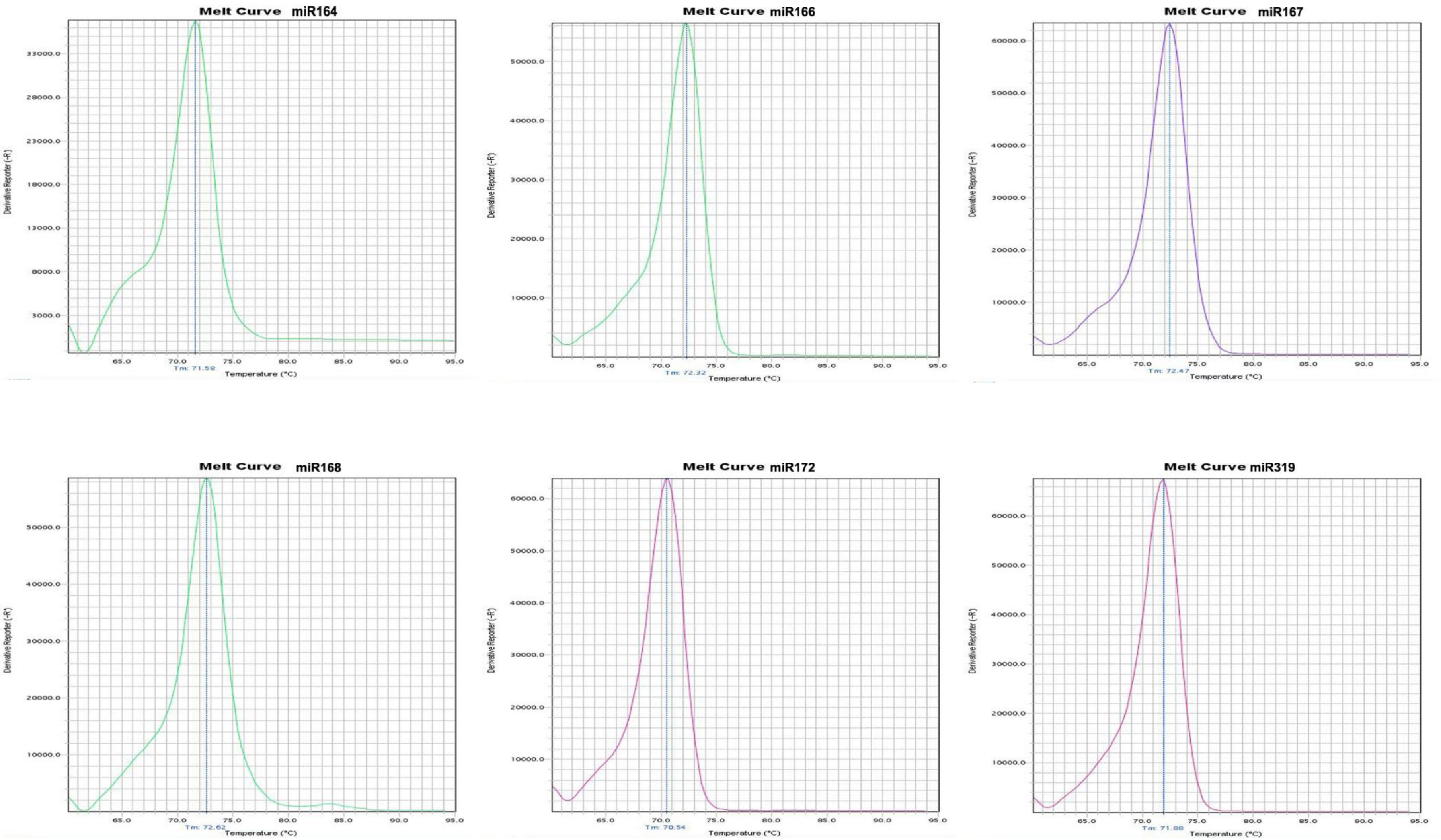

Melt Curve miR164
Tm: 71.58
Melt Curve miR166
Tm: 72.32
Melt Curve miR167
Tm: 72.47
Melt Curve miR168
Tm: 72.62
Melt Curve miR172
Tm: 70.54
Melt Curve miR319
Tm: 71.88
Derivative Reporter (-R')
Temperature (°C)

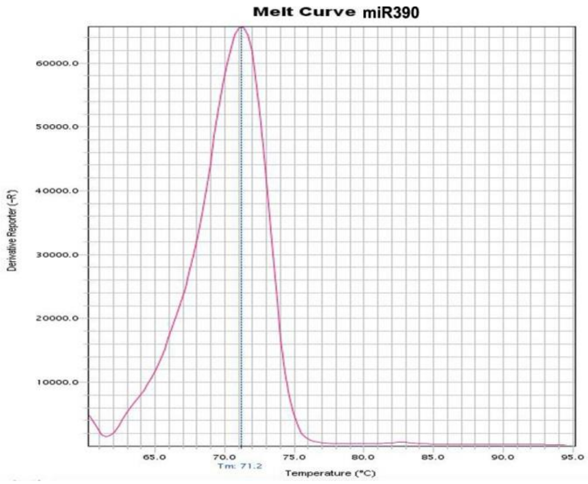
Melt Curve miR390
Derivative Reporter (-R)
Temperature (°C)
Tm: 71.2

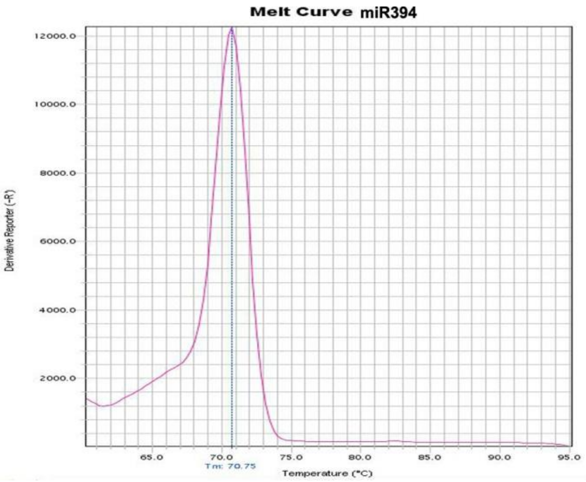
Melt Curve miR394
Derivative Reporter (-R)
Temperature (°C)
Tm: 70.75

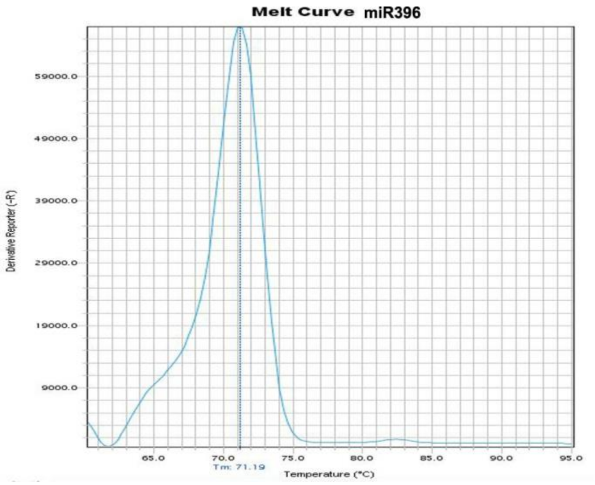
Melt Curve miR396
Derivative Reporter (-R)
Temperature (°C)
Tm: 71.19

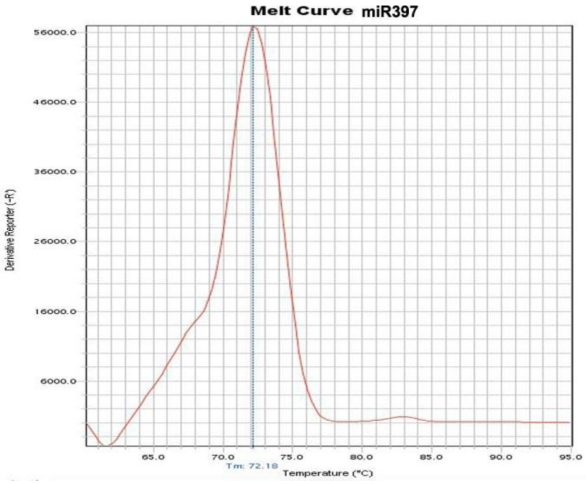
Melt Curve miR397
Derivative Reporter (-R)
Temperature (°C)
Tm: 72.18

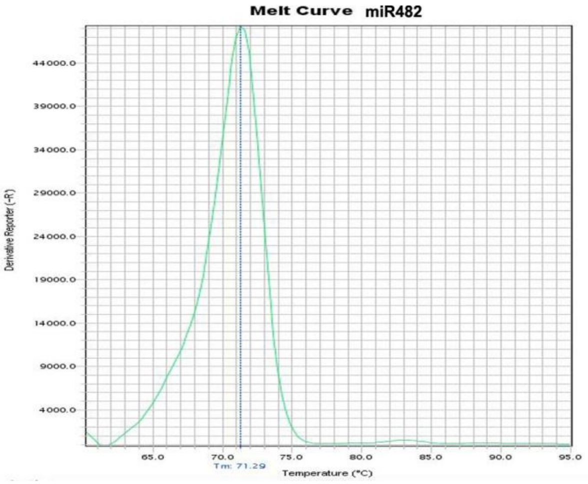
Melt Curve miR482
Derivative Reporter (-R)
Temperature (°C)
Tm: 71.29

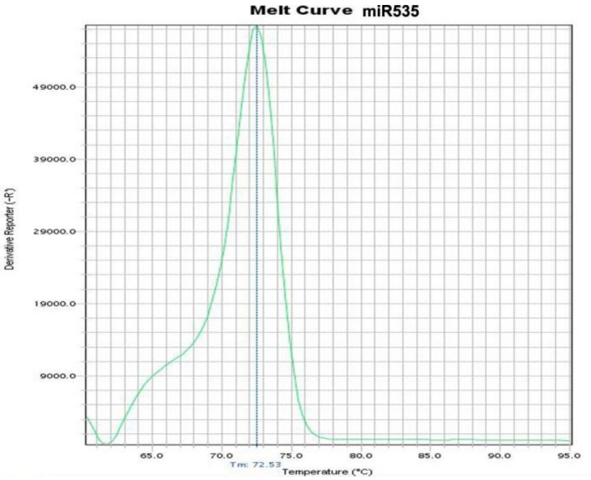
Melt Curve miR535
Derivative Reporter (-R)
Temperature (°C)
Tm: 72.53

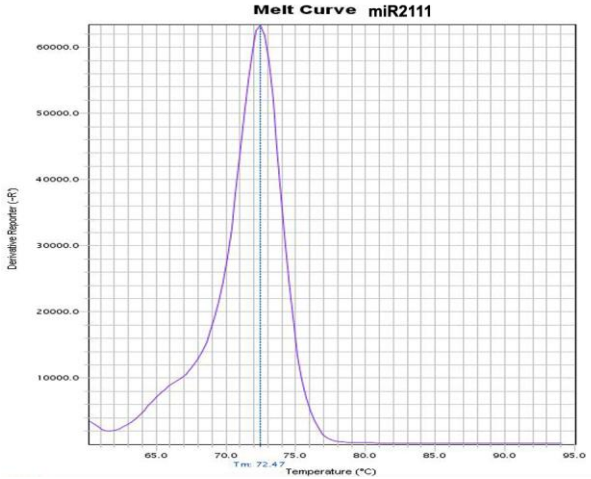
Melt Curve miR2111
Derivative Reporter (-R')
Temperature (°C)
Tm: 72.47

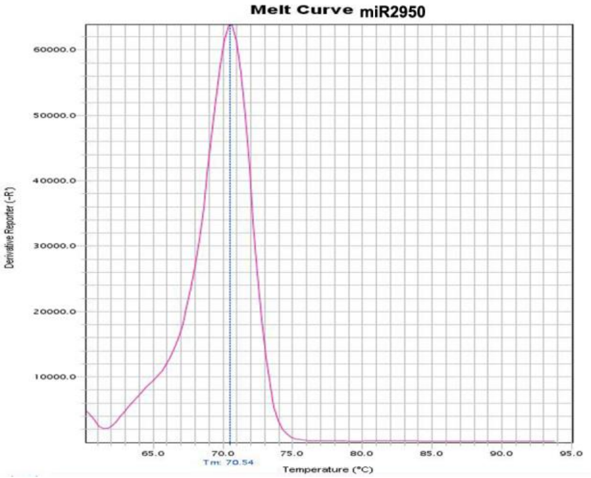
Melt Curve miR2950
Derivative Reporter (-R')
Temperature (°C)
Tm: 70.54

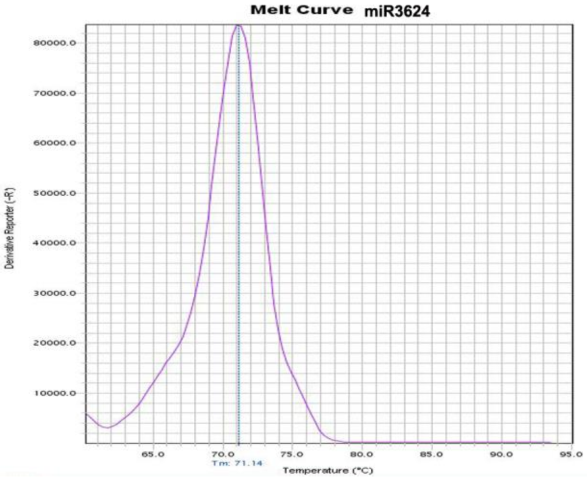
Melt Curve miR3624
Derivative Reporter (-R')
Temperature (°C)
Tm: 71.14

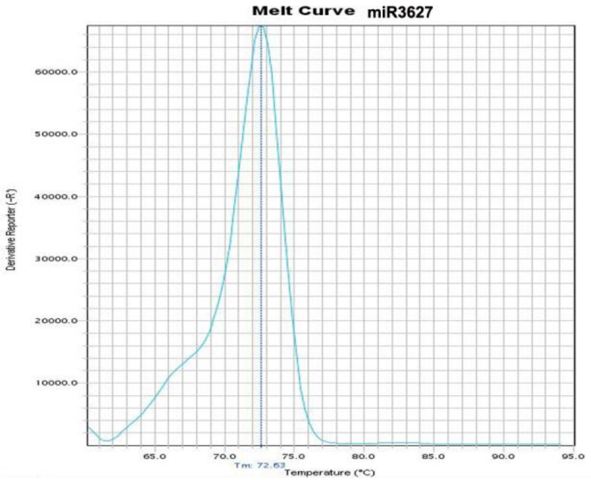
Melt Curve miR3627
Derivative Reporter (-R')
Temperature (°C)
Tm: 72.63

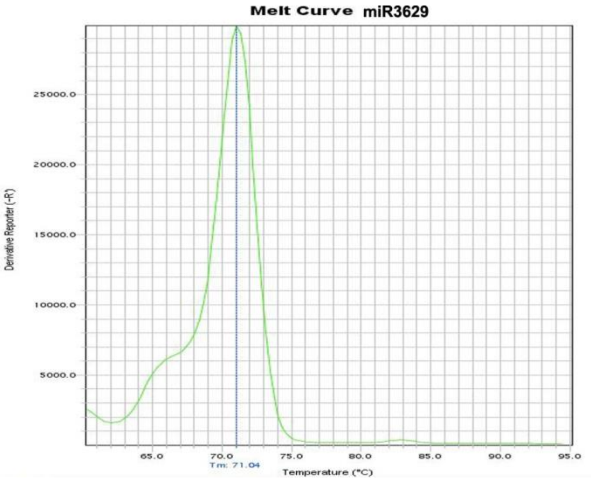
Melt Curve miR3629
Derivative Reporter (-R')
Temperature (°C)
Tm: 71.04

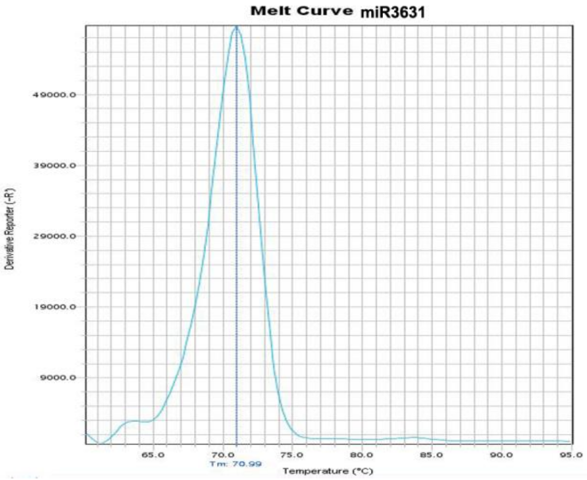
Melt Curve miR3631
Derivative Reporter (-R')
Temperature (°C)
Tm: 70.99

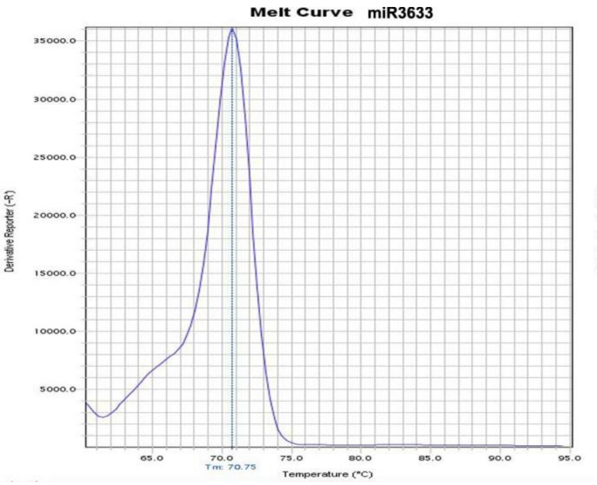
Melt Curve miR3633
Derivative Reporter (-R')
35000.0
30000.0
25000.0
20000.0
15000.0
10000.0
5000.0
65.0
70.0
75.0
80.0
85.0
90.0
95.0
Tm: 70.75
Temperature (°C)

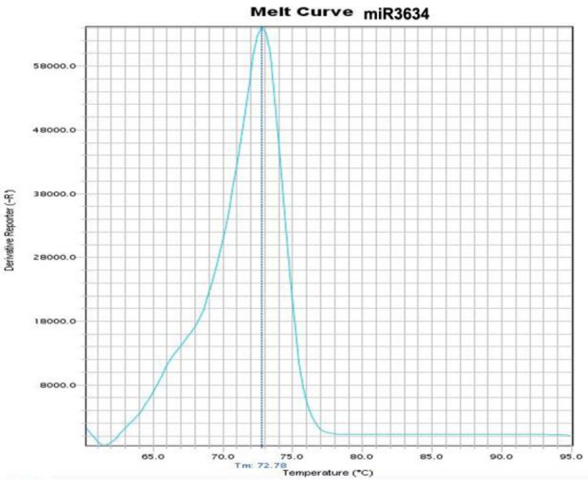
Melt Curve miR3634
Derivative Reporter (-R')
58000.0
48000.0
38000.0
28000.0
18000.0
8000.0
65.0
70.0
75.0
80.0
85.0
90.0
95.0
Tm: 72.78
Temperature (°C)

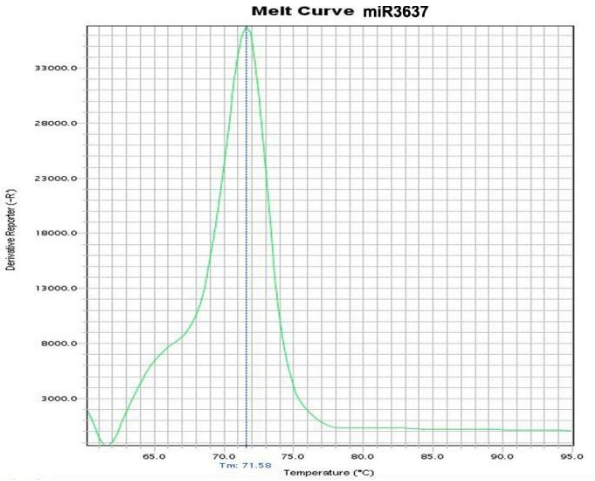
Melt Curve miR3637
Derivative Reporter (-R')
33000.0
28000.0
23000.0
18000.0
13000.0
8000.0
3000.0
65.0
70.0
75.0
80.0
85.0
90.0
95.0
Tm: 71.58
Temperature (°C)

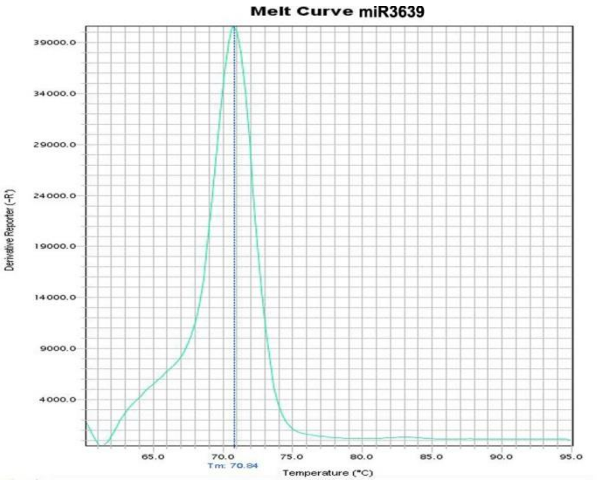
Melt Curve miR3639
Derivative Reporter (-R')
39000.0
34000.0
29000.0
24000.0
19000.0
14000.0
9000.0
4000.0
65.0
70.0
75.0
80.0
85.0
90.0
95.0
Tm: 70.84
Temperature (°C)

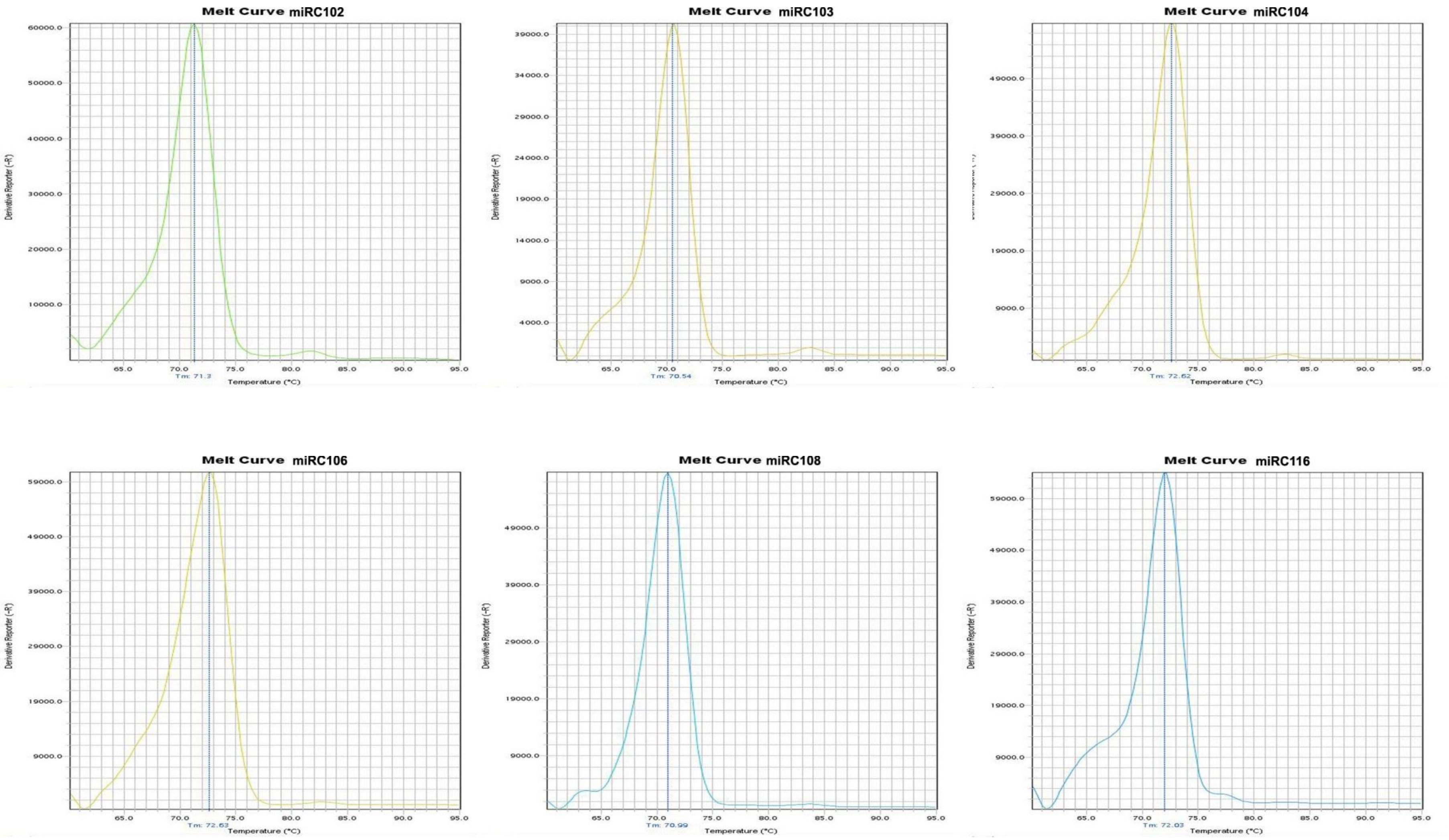

Melt Curve miRC102
Tm: 71.3
Melt Curve miRC103
Tm: 70.54
Melt Curve miRC104
Tm: 72.62
Melt Curve miRC106
Tm: 72.63
Melt Curve miRC108
Tm: 70.99
Melt Curve miRC116
Tm: 72.03
Derivative Reporter (-R)
Temperature (°C)

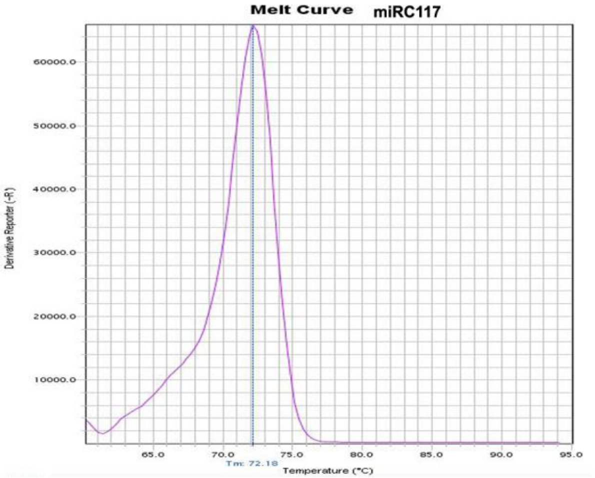
Melt Curve miRC117
Derivative Reporter (-R)
Tm: 72.18
Temperature (°C)

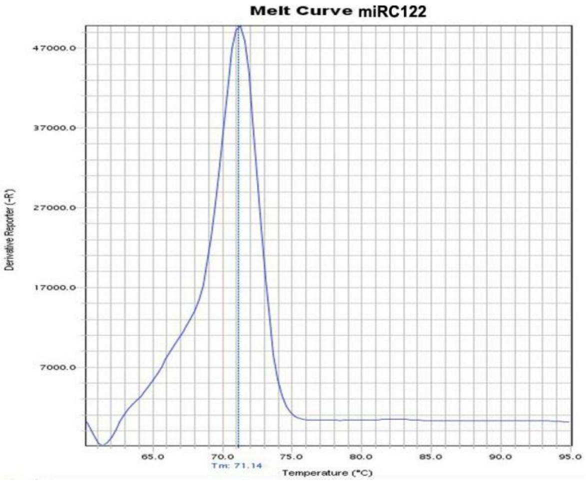
Melt Curve miRC122
Derivative Reporter (-R)
Tm: 71.14
Temperature (°C)

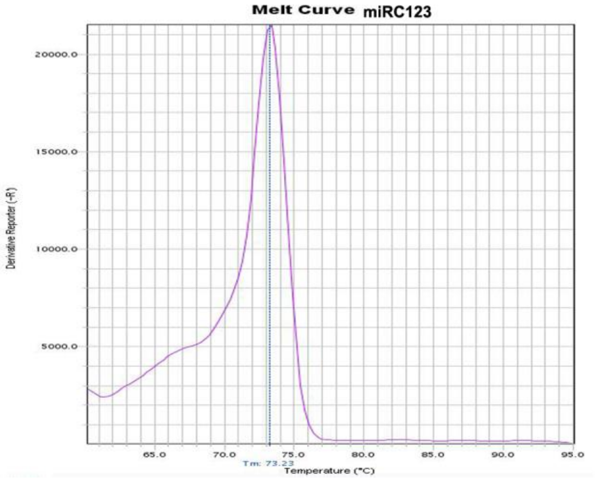
Melt Curve miRC123
Derivative Reporter (-R)
Tm: 73.23
Temperature (°C)

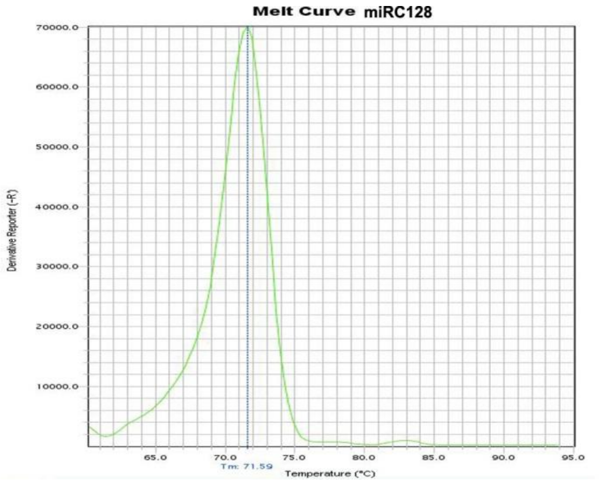
Melt Curve miRC128
Derivative Reporter (-R)
Tm: 71.59
Temperature (°C)

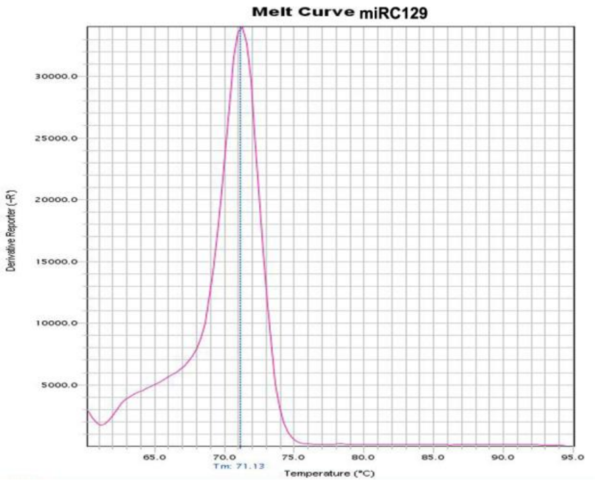
Melt Curve miRC129
Derivative Reporter (-R)
Tm: 71.13
Temperature (°C)

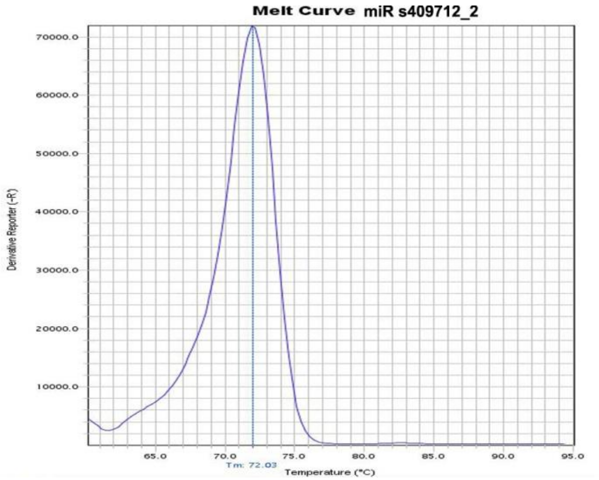
Melt Curve miR s409712_2
Derivative Reporter (-R)
Tm: 72.03
Temperature (°C)

**b**

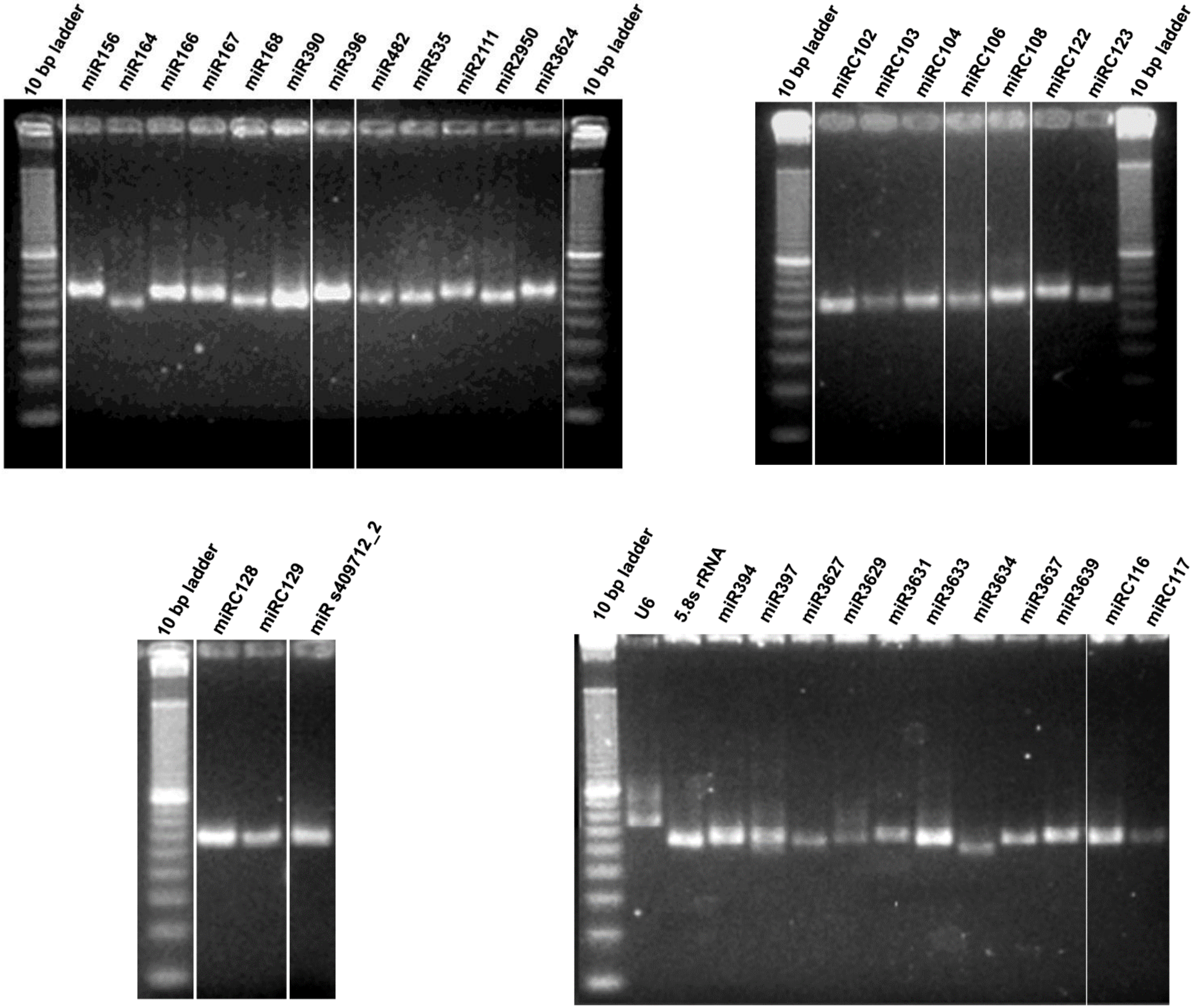
10 bp ladder
miR156
miR164
miR166
miR167
miR168
miR390
miR396
miR482
miR535
miR2111
miR2950
miR3624
10 bp ladder
10 bp ladder
miRC102
miRC103
miRC104
miRC106
miRC108
miRC122
miRC123
10 bp ladder
10 bp ladder
miRC128
miRC129
miR s409712_2
10 bp ladder
U6
5.8s rRNA
miR394
miR397
miR3627
miR3629
miR3631
miR3633
miR3634
miR3637
miR3639
miRC116
miRC117

**Supplementary Figure S4**. Relative expression level of miR166, miR167, miR168, miR172, miR319, miR390, miR394, miR397, miR482, miR535, miR2111, miR2950, miR3624, miR3627, miR3629, miR3631, miR3633, miR3634, miR3637 and miR3639 in *Grapevine rupestris stem pitting-associated virus* (GRSPaV)-free and infected 'Bosco' leaf as determined by qRT-PCR. Samples were collected under: well watered (WW), water stress (SW), severe water stress (SWS) conditions.  qRT-PCR signals were normalised to U6 and 5.8 rRNA. Data are presented as mean ± standard deviation of three biological replicates; different letters denote significant differences at $p \leq 0.05$.

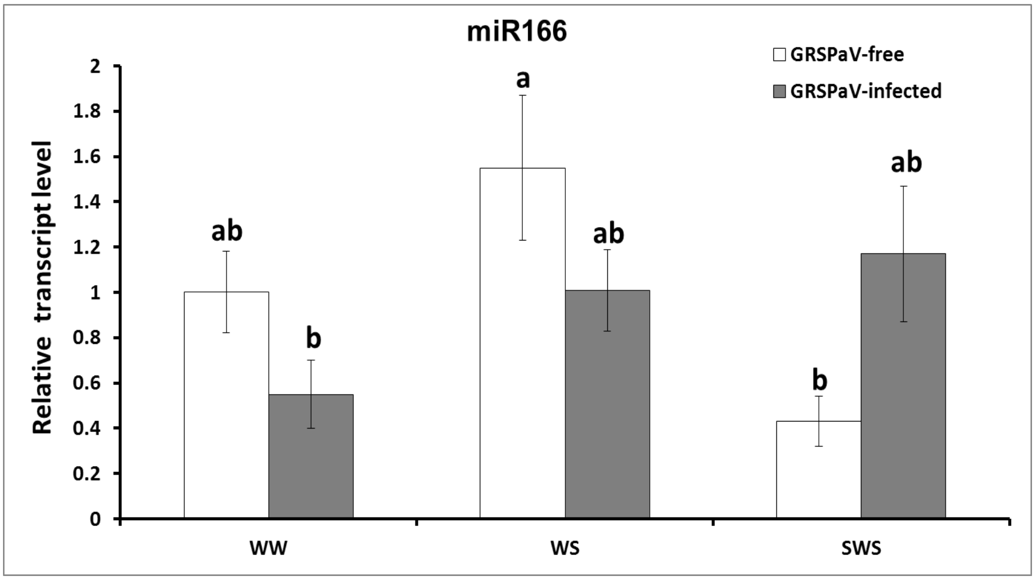


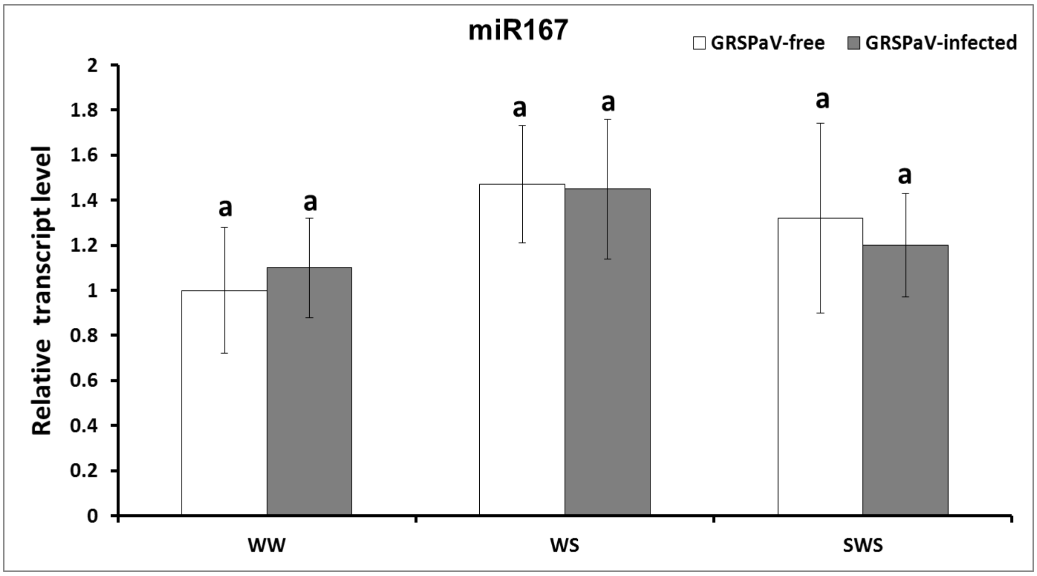

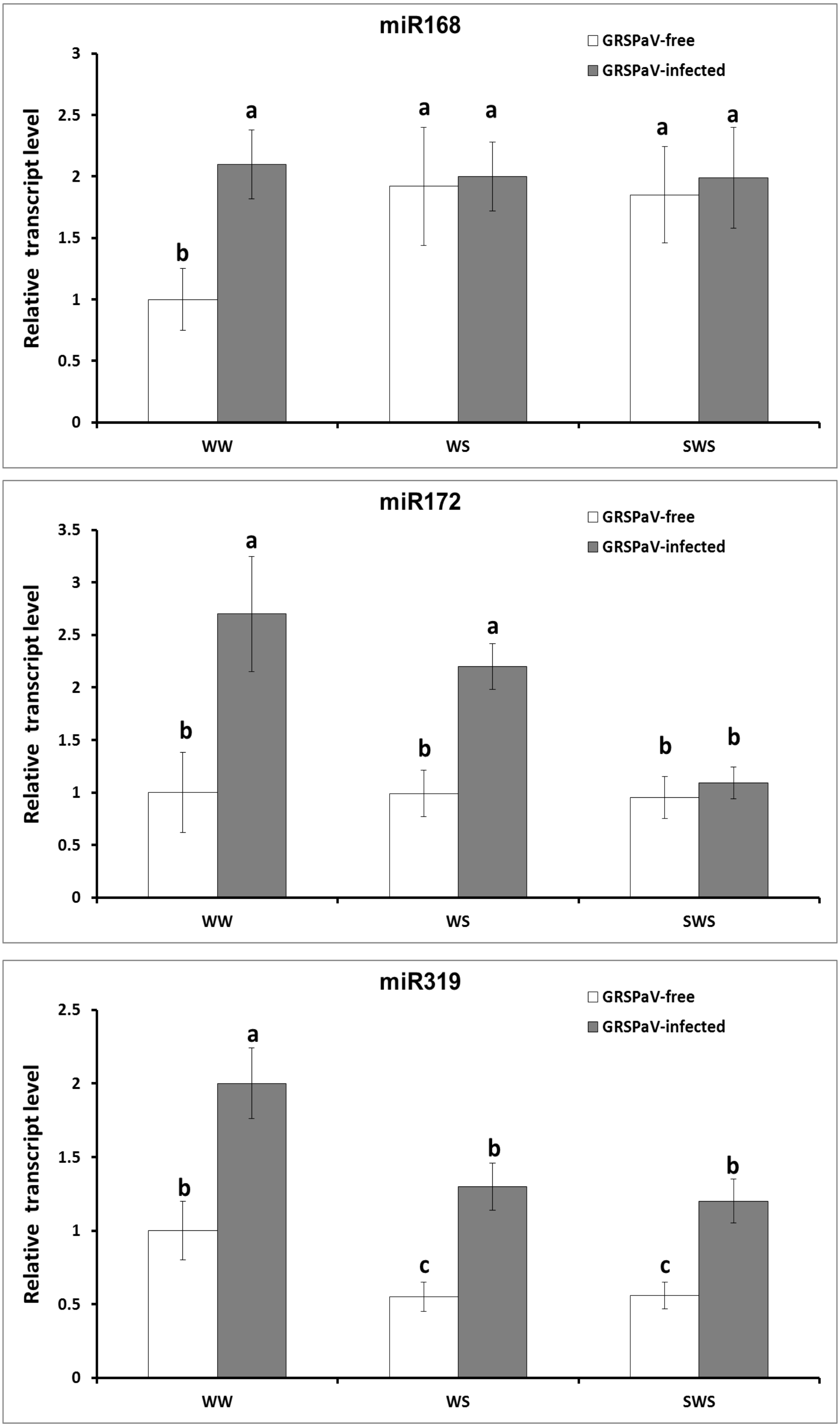

miR168
GRSPaV-free
GRSPaV-infected
Relative transcript level
0
0.5
1
1.5
2
2.5
3
a
b
a
a
a
a
WW
WS
SWS
miR172
GRSPaV-free
GRSPaV-infected
Relative transcript level
0
0.5
1
1.5
2
2.5
3
3.5
a
b
a
b
b
b
WW
WS
SWS
miR319
GRSPaV-free
GRSPaV-infected
Relative transcript level
0
0.5
1
1.5
2
2.5
a
b
b
c
b
c
WW
WS
SWS

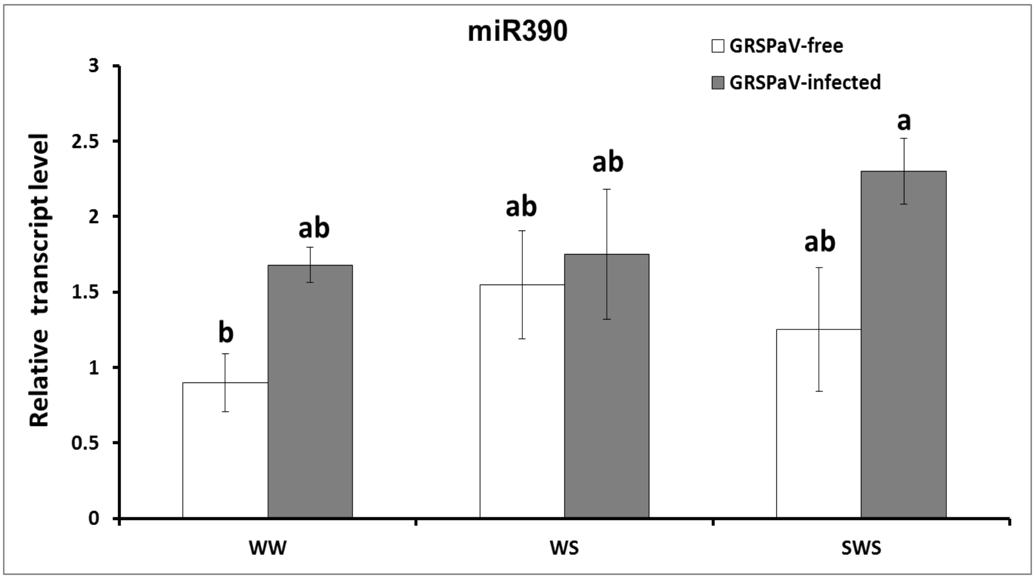
miR390
GRSPaV-free
GRSPaV-infected
Relative transcript level
0
0.5
1
1.5
2
2.5
3
b
ab
ab
ab
ab
a
WW
WS
SWS

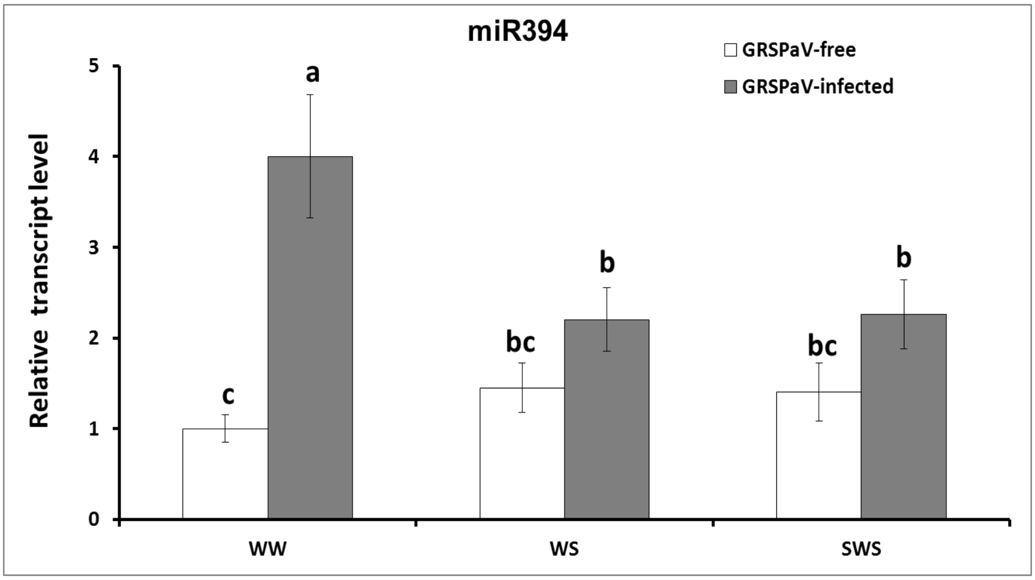
miR394
GRSPaV-free
GRSPaV-infected
Relative transcript level
0
1
2
3
4
5
c
a
bc
b
bc
b
WW
WS
SWS

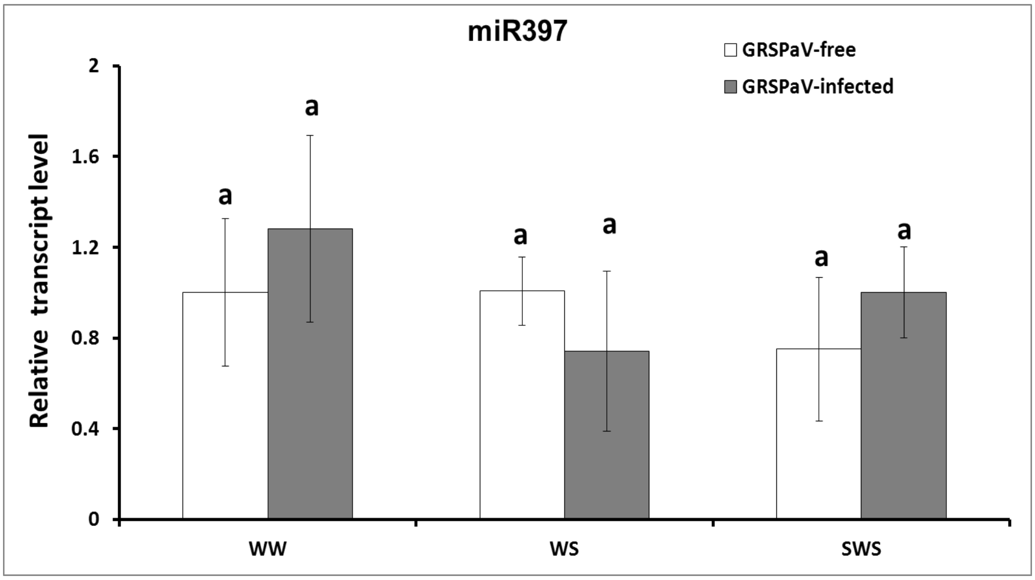
miR397
GRSPaV-free
GRSPaV-infected
Relative transcript level
0
0.4
0.8
1.2
1.6
2
a
a
a
a
a
a
WW
WS
SWS

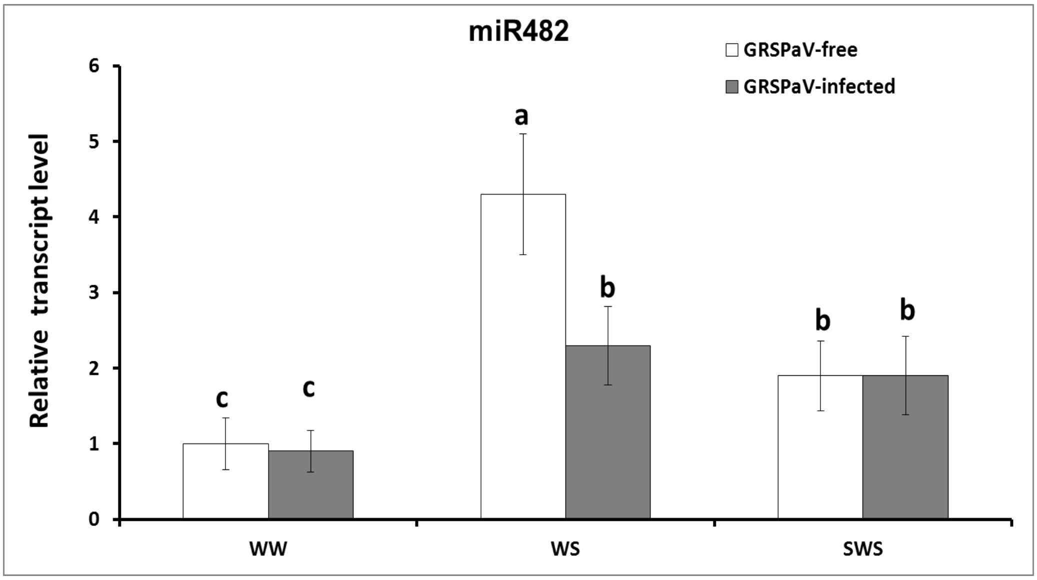
miR482
GRSPaV-free
GRSPaV-infected
Relative transcript level
0
1
2
3
4
5
6
a
b
b
b
c
c
WW
WS
SWS

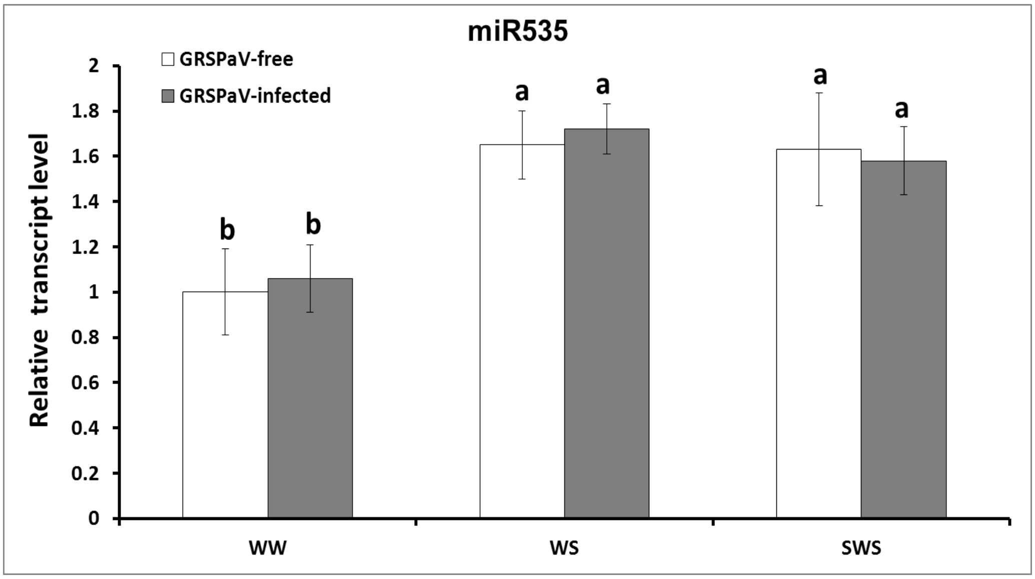
miR535
GRSPaV-free
GRSPaV-infected
Relative transcript level
0
0.2
0.4
0.6
0.8
1
1.2
1.4
1.6
1.8
2
a
a
a
a
b
b
WW
WS
SWS

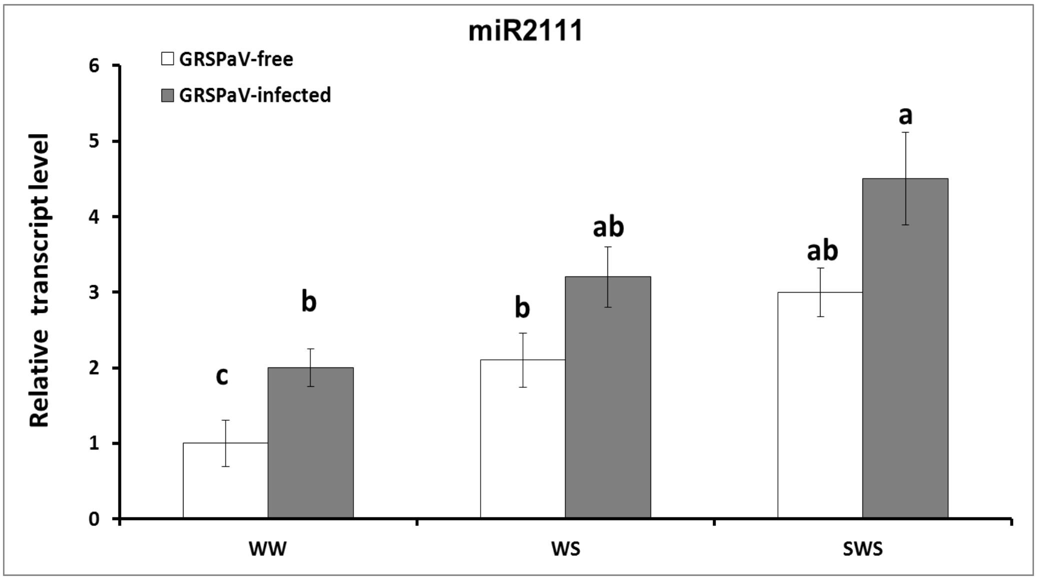
miR2111
GRSPaV-free
GRSPaV-infected
Relative transcript level
0
1
2
3
4
5
6
a
ab
ab
b
b
c
WW
WS
SWS

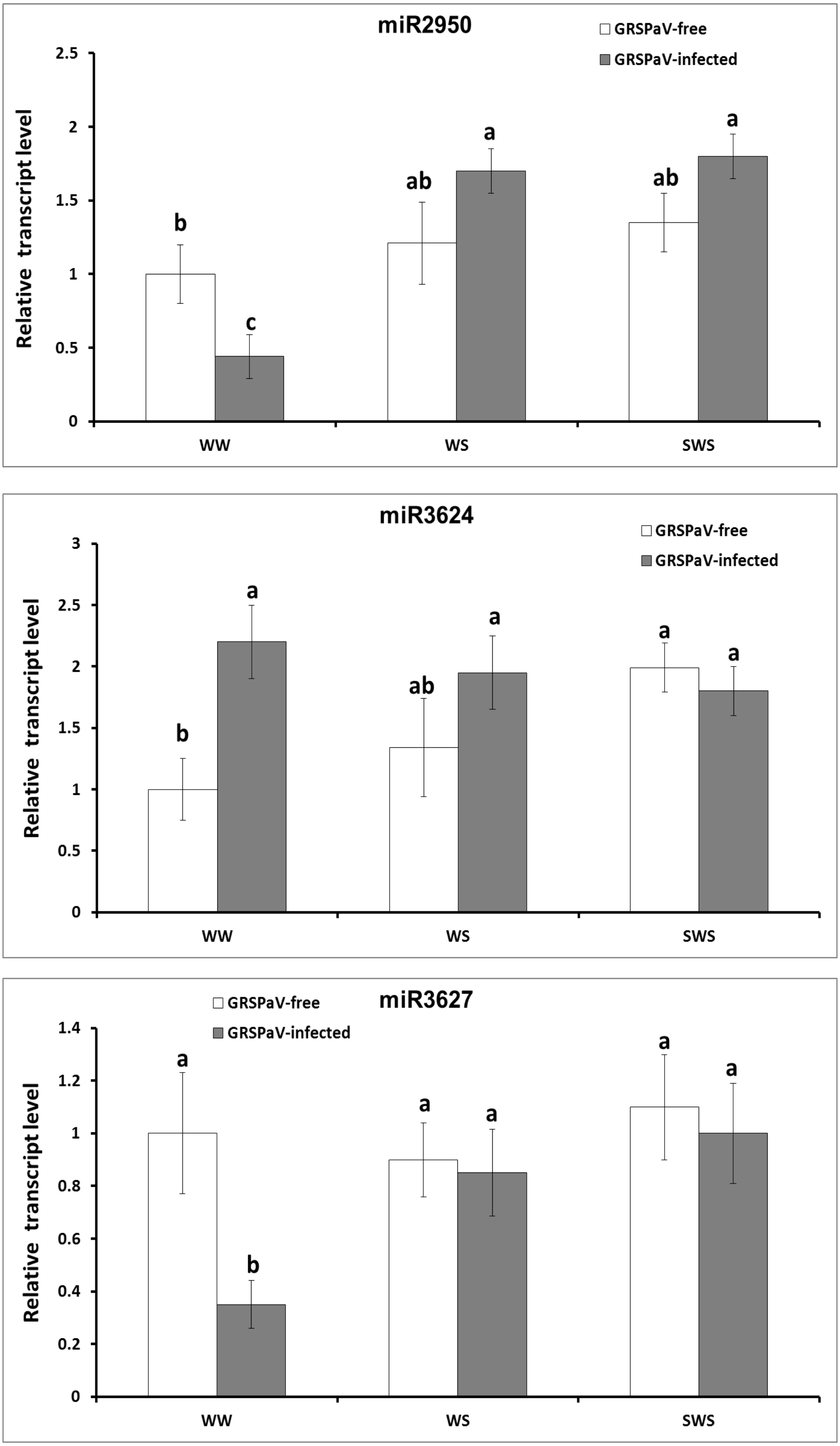
miR2950
GRSPaV-free
GRSPaV-infected
Relative transcript level
2.5
2
1.5
1
0.5
0
b
c
ab
a
ab
a
WW
WS
SWS
miR3624
GRSPaV-free
GRSPaV-infected
Relative transcript level
3
2.5
2
1.5
1
0.5
0
b
a
ab
a
a
a
WW
WS
SWS
miR3627
GRSPaV-free
GRSPaV-infected
Relative transcript level
1.4
1.2
1
0.8
0.6
0.4
0.2
0
a
b
a
a
a
a
WW
WS
SWS

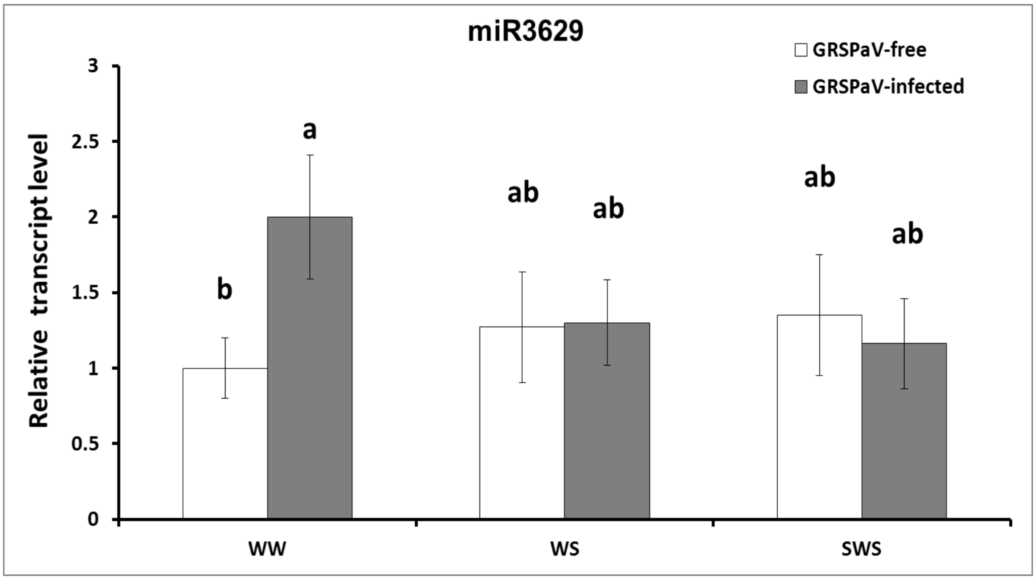
miR3629
GRSPaV-free
GRSPaV-infected
Relative transcript level
3
2.5
2
1.5
1
0.5
0
a
b
ab
ab
ab
ab
WW
WS
SWS

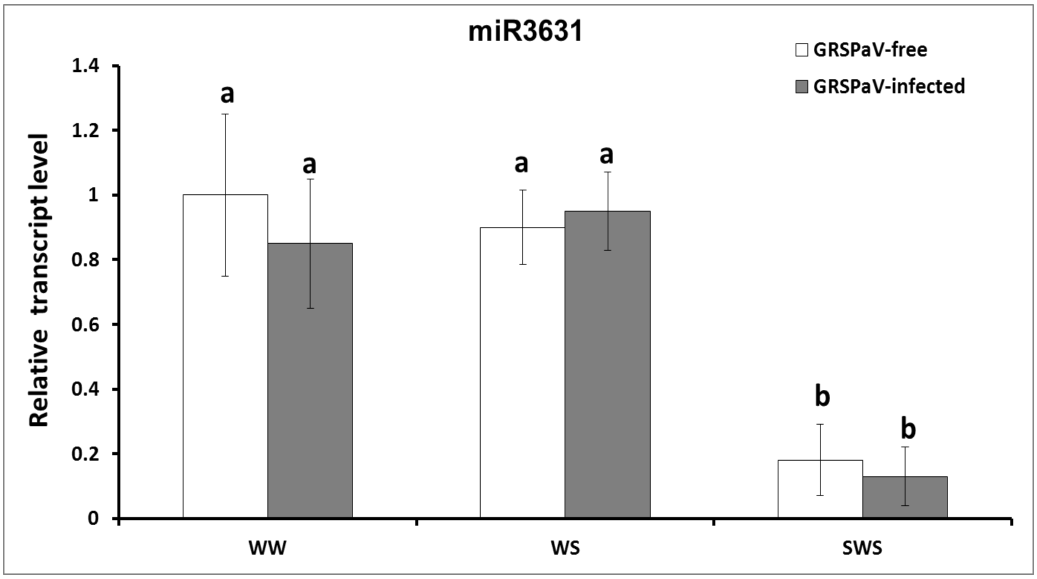
miR3631
GRSPaV-free
GRSPaV-infected
Relative transcript level
1.4
1.2
1
0.8
0.6
0.4
0.2
0
a
a
a
a
b
b
WW
WS
SWS

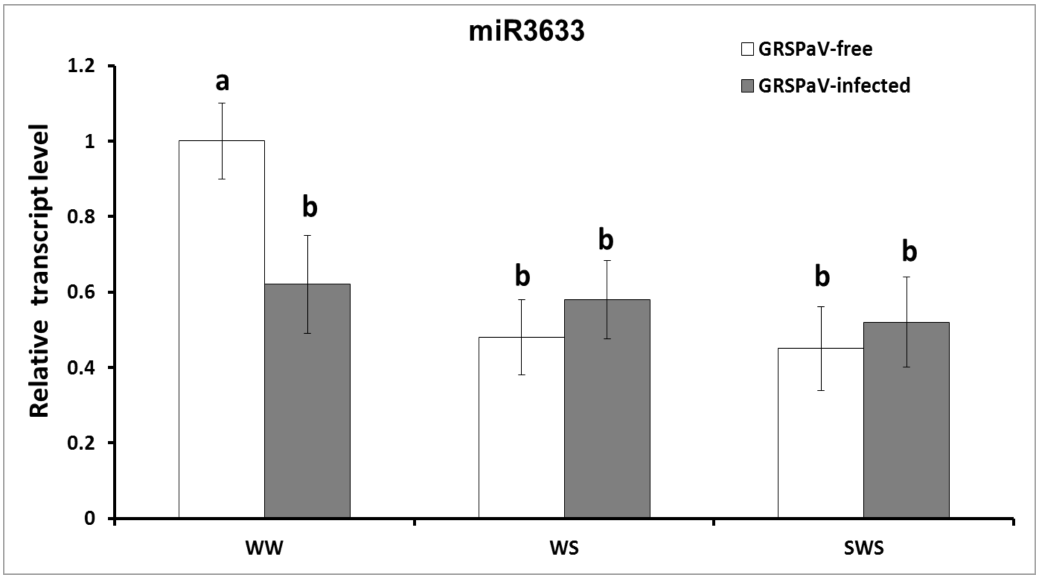
miR3633
GRSPaV-free
GRSPaV-infected
Relative transcript level
1.2
1
0.8
0.6
0.4
0.2
0
a
b
b
b
b
b
WW
WS
SWS

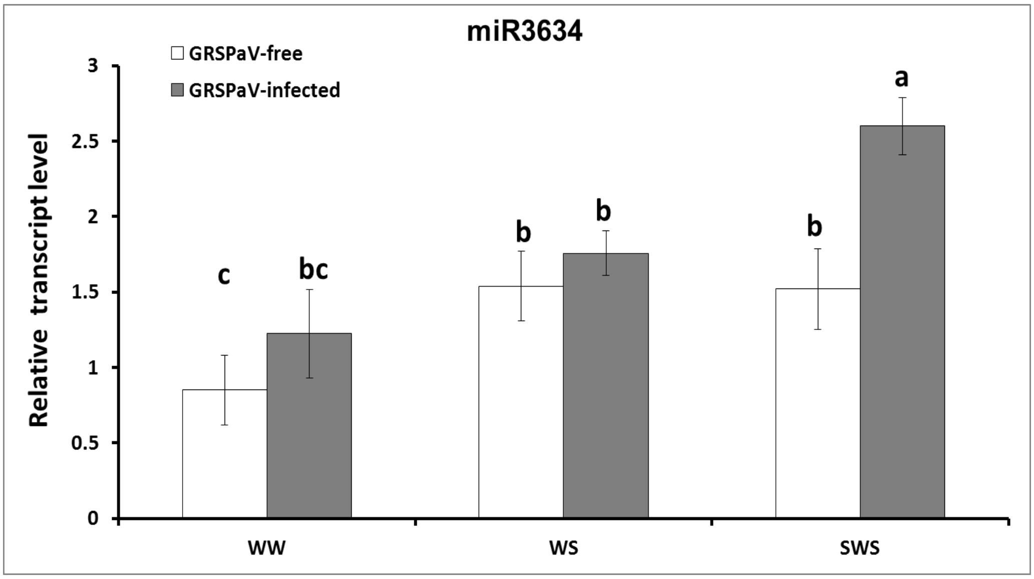
miR3634
GRSPaV-free
GRSPaV-infected
Relative transcript level
0
0.5
1
1.5
2
2.5
3
WW
WS
SWS
c
bc
b
b
b
a

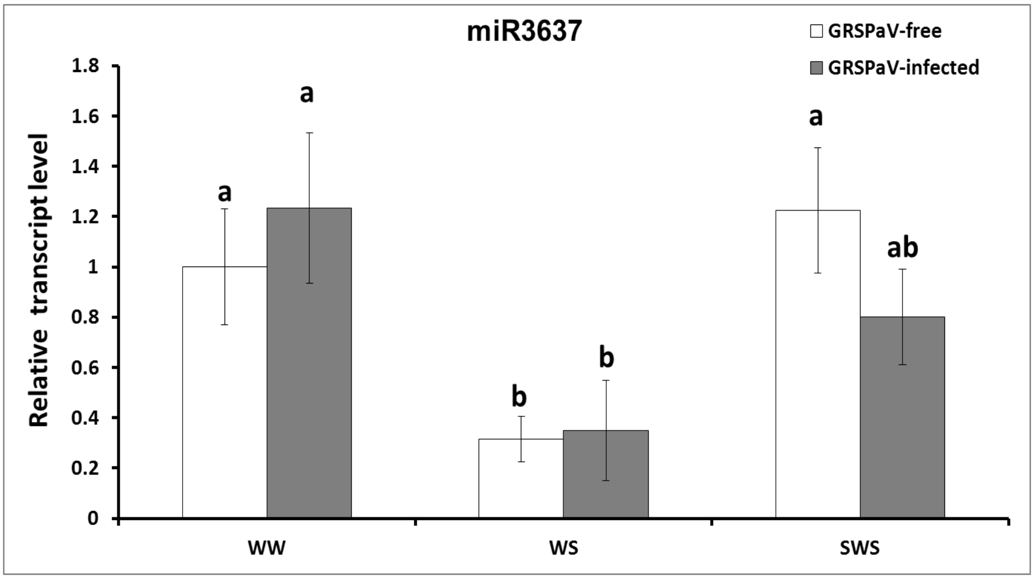
miR3637
GRSPaV-free
GRSPaV-infected
Relative transcript level
0
0.2
0.4
0.6
0.8
1
1.2
1.4
1.6
1.8
WW
WS
SWS
a
a
b
b
a
ab

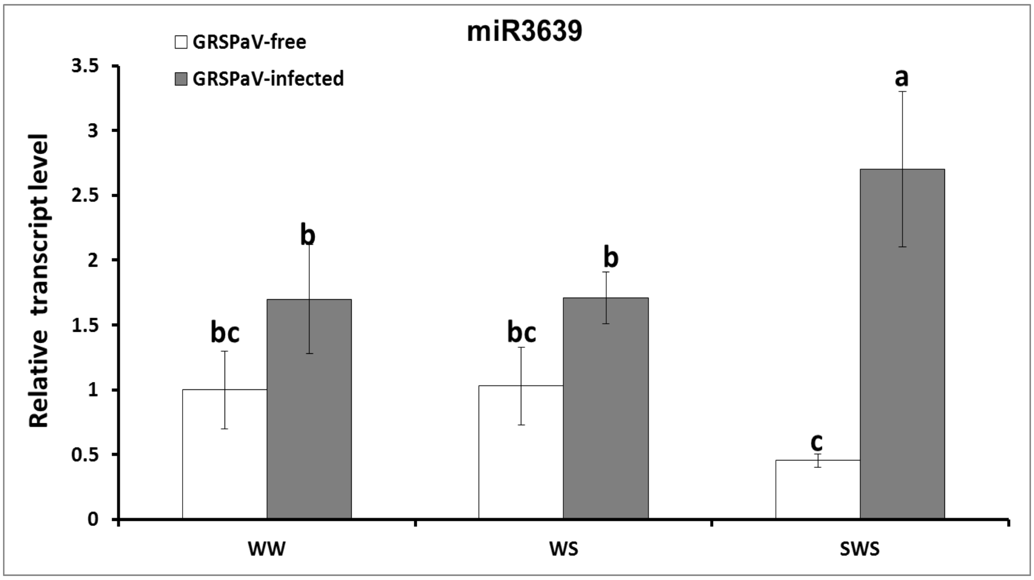
miR3639
GRSPaV-free
GRSPaV-infected
Relative transcript level
0
0.5
1
1.5
2
2.5
3
3.5
WW
WS
SWS
bc
b
bc
b
c
a

**Supplementary Figure S5**. Predicted secondary structures of new and putative grapevine specific miRNAs. In red and violet are indicated the miRNA and the miRNA*, respectively. "(" and "." indicate paired and unpaired bases, respectively. Within the mature miRNA "<" and "-" indicate paired an unpaired bases, respectively. Within the miRNA*, "{" and "=" indicate paired an unpaired bases, respectively.

**miRC102**

```
>TTTGGCAGTGATTCTAGGAAA_chr12/22422891-22422911
GTTTGGCAGTGATTCTAGGAAACACATCTAACCTTTTTAGCTTTTGAAAATTTTGATCAAAAGAGTTGTTTTGAAGCAATAGAAAGACTAGAAATACTTCCTAAAATCACTATCAAAC
(<<<<--<<<<<<<<-<<<<<<-....((((..(((((((((((.((((((((.......)))))).))).)))))..)))))))..))))).....))))))).)))))))..)))))
```

```
>TTTGGCAGTGATTCTAGGAAA_chr19/19300144-19300164
AGCCCATTTGGCAGTGATTCTAGGAAACTCTTCTAACATTTCTAGCACTTGAAAATTTTTATCCTTTCAAGCCTTAGAAAAGGCCGGAAACATTTTCTAGAATCATTGCAAAACAGGCT
((((..<<<-<<<<<<<<<<<<<<<<<<...((((..(.((((((((..((((((((.........)))))))..))))))).)...))))...)))))))))))))))))).))))..))))
```

**miRC103**

```
>AGTGTTTCTGGGCTTTATAAC_chr7/15865768-15865789
AGAAAGTGTTTCTGGGCTTTATAACACTTTGGAAATTTTCATCATTCGAGTATTATAAAGTCCAAAAACACTTCT
(((<-<<<<<<<-<<<<<<<<<<<<<-((((..((........))....)))).))))))))))))).))))))))))
```

**miRC104**

```
>CTGAACTCTCTCCCTCATGGCC _chr14/24560699-24560720
AGAGAAATTGACTTTATCTGAACTCTCTCCCTCATGGCCTTTCTAAGCTTTATCCGAATGTCTTAAGAAAGGAGAAGCTGGGGAAGCCAAGAGGGTGGAGTTCAGATAATGTTGGTTTCTCT
((((((((..((.((((<<<<<<<<<<--<<<<<<-<<<<<-(((((.((((((.(((.....((....))..)))))))))).))))))))).))))).))))))))))))).))..))))))))
```

**miRC106**

```
>CTTTTGTTTCTTCGTGTCGGT_chr16/18836667-18836687
GGCCGGAAAAGCGAGCTCGACACGAAGAAACGAAAGCCGGCCAATGCAGTCCGAGAGATCACACAGCGAACTGTTCGAGTCTGGGCTTTTGTTTCTTCGTGTCGGTCTTGCTTCTGTCTCCGGCC
(((((((.((((((((..({{{{{{{{{{{{{{={{{{==(((....(((((.((.............)).)))))....)))..))>>>>>>>>>>>>>>>>>>>>--))))))).....)))))))
```

```
>CTTTTGTTTCTTCGTGTCGGT_chr16/18867640-18867660
GGCCGGAAAAGCGAGCTCGACACGAAGAAACGAAAGCCGGCCAATGCAGTCCGAGAGATCACACAGCGAACTGTTCGAGTCTGGGCTTTTGTTTCTTCGTGTCGGTCTTGCTTCTGTCTCCGGCC
(((((((.((((((((..({{{{{{{{{{{{{{={{{{== (((....(((((.((.............)).)))))....)))..))>>>>>>>>>>>>>>>>>>>>--))))))).....)))))))
```

**miRC108**

```
>TACTTTGGATGATAATGGGCC_chr19/10790715-10790735
GCCCAAACTATGATTTGGAATACTTTGGATGATAATGGGCCTAGTTGAGATTTTATATAATTCAAGGTAGATTTCTGCCCAAGTATATTCACCTTGCATTATATAAAATCTCAACTAGGCCAATTATCATCCAAAGTATGCCAAATCAGAATTTGGGC
((((((...((((((((.(<<<<<<<<<<<<<<<<<-<<<<((((((((((((((((((((.((((((..((..((.....))...))..)))))).))))))))))))))))))))))))).)))))))))))))))))).))))))))...)))))))
```

**miRC116**

>TTCTTCTCACCGTTTCTCAGC_chr9/3184798-3184818
GTCCTCTGTTTGCTTGCTGAGAAACTGGGGGGAAAATGAAAGAAAACTATAAATCTTTTCAACATGTTCGAGTTGGAGTGGCAACTGTGACAATCCGGATTTTTTTTTTTCTTCTTCTCACCGTTTCTCAGCCAACAAATGGAGAAC
((.(((((((((...(({{{{{{{{{={{={={{{={==((((((((.....(((((.((((((........))))))((.((....)).)).....))))))))))))))--->>>>>>->>>>>>>>>>>>...))))))))).))

**miRC117**

>TCTTAGAACCAAGGGCACCCT_chr9/5984686-5984706
TTTTTCTTAGAACCAAGGGCACCCTTAATCCTTCCCCGACTTCGATTCCGATATAAGGTATCAATGGGACCCATTGGTTCAAGAAAGA
((((<<<<<-<<<<<<<<<<<--<<<-....((((........(((....)))...)))).......))).))).))))))))))))))

>TCTTAGAACCAAGGGCACCCT_chr14/19624264-19624284
CTTTTTTCTTAGAACCAAGGGCACCCTTAATATGTCCCTGACTTCAATTCCTAGGTGAGGTATCAATGGGACCCATTGGTTTGAGAATGAAG
((((.(<<<<<<<<-<<<<<<<<--<<-<(.((((.((((((...........)))).)).)))).)).)).))..))).))))))))))))).))))

**miRC122**

>TTATGGGACTACATGTGTTACA_chr3/17925351-17925372
CCGGTCCGCACCAACACACGTATTATTAAAGTGTTACACATGTAGTGCCATCATATGAAGAAAAGTGTATGATTTTTTTAATCAATCAGTTCTTTATATTATGGGACTACATGTGTTACACTTTGATGACATGTGGGTTAGTGTGGACCGG
((((((((((.(((.(((((.((((((((((((={{{{{{{{{{{={{{{={{{((((((((...((..(((((.....)))))..)).)))))))))>->>>>>->>>>>>>>>>>>->>>)))))))))).))))).))).))))))))))

**miRC123**

>TACCAAGGTCGAGGATTGACT_chr14/14721702-14721722
AAGGCTAAACAAACTCTTATACCAAGGTCGAGGATTGACTTCAATACTATTTGTAATGACCCCCTCCTAGCCAATAGGGGAAAAAGTTCTAAAGGCACAAGGGTTAATCATTCTTAGATTTGACAATATCTACATGGTTGAGAATTAGTCGTTACGAATGATATGAGAGTCAAACCTCAACCTTGGTATGAGAGTTTGTTTGTCCTT
((((.(((((((((((((<<<<<<<<<<-<<<<-<<<<<<(..(((.(((((((((((((.((((....(((..((((((.......))))))..)))...))))((((((((...(((((........))))).))))))))......))))))))))))).)))..))){{{{={{{{={{{{{{{{{{{{)))))))))))))).))))

**miRC128**

>AGAAGTCAATCCAAACAAGGTC_chr9/21823140-21823161
ACCTGTTTGGATTGACTTCTGAAAGGCTAAAAGCTCTTATTAAGCTTAAATAAGAGCTTTTGGCTTCTCAGAAGTCAATCCAAACAAGGT
((((((((((((((((((((((.((((((((((((((((((.......)))))))))))))))))))).))>>>>>>>>>>>>>>>>->>>>

**miRC129**

>ACCGTCTTTCTCTGTATAAGC_chr5/6820744-6820764
AGGATCCATGCCATTTCATTACACAGAGAGATGACGGTGGAGATTTGTCTCTATTGCTCTCTACCGTCTTTCTCTGTATAAGCAGATGGAGTGGATCTT
(((((((((.((((((..{{{={{{{{{{{{={{{{{{{{((((..............)))))>>>>>>>>>>>>>>>->>>--)))))).)))))))))

**miRs409712_2**

>ATGTGTTTTAAAGTCGTGAGGG_chr10/4096605-4096626
ACCTTGTAGATGTGTTTTAAAGTCGTGAGGGGCCCTTTGGGTCCAAAGCAGACAATATCTACACGATTGGGAACGAGTCATTACAAATGGT
(((((((((<<-<<<<<---<<<<<<<--<<((((...)))))).....(((.....))).)))))))..))))).)))..)))))..)))

>ATGTGTTTTAAAGTCGTGAGGG_chr14/24304140-24304161
TCAATGGACCCTCATGACTTTAAAATGCATCTACATAACTAAGAAAAGTTCATTGCATATACAGTGTTAGGAACTTTTCCCCTTTACCCTAATTATGTAGATGTGTTTTAAAGTCGTGAGGGTCCATTGA
((((((((((((((((((((((((((((((((((((.((.((((((((((...((((.....))))...))))))))))..........)).))))))))>>>>>>>>>>>>>>>>>>>>>>))))))))

>ATGTGTTTTAAAGTCGTGAGGG_chr19/20397809-20397830
CTCTTAATCTTGTAGATGTGTTTTAAAGTCGTGAGGGTTAAGTCATTACAGATGGTATTAAAGTCGATCCTCGACCCCAATGTGGAGGTTTATTTGGTCCTATGAGGGGTGTTTGTAATATCCCACATTGGATGAGAGAGAAAATTACTTAGACTATATATGTATGAACTCCTCTTAACTTTGTAGACGTGTTTTAAAGTTATGAG
(((.(((..(((.((<--<<<<<<<<<<<<---<<<<<.....((((.(((.(((((....((((....((((.((......)).))))....(((.((((.(((.(((((((.....))))))).))).))))).))).........))))..)))))...)))))))..))))))...)))))).)))))..))))))..))).)))

**miRC104**

```
miRC104             1  cugaacucucucccucau  18
                       ||||||||||||||||||
mes-miR1446         3  cugaacucucucccucau  20

miRC104             1  cugaacucucucccuca  17
                       |||||||||||||||||
ptc-miR1446a        3  cugaacucucucccuca  19
```

**miRC108**

```
miRC108             5  uuggaugauaau  16
                       ||||||||||||
stu-miR7982a        4  uuggaugauaau  15

miRC108             5  uuggaugauaau  16
                       ||||||||||||
stu-miR7982b        4  uuggaugauaau  15
```

**miRC129**

```
miRC129             2  ccgucuuucucuguauaa  19
                       |||| ||||| |||||||
ppe-miR7122a-3p     2  ccguguuucuuuguauaa  19

miRC129             1  accgucuuucucuguauaagc  21
                       || |  ||||||||||||| |
stu-miR7122-3p      1  acagcguuucucuguauaacc  21
```

**Supplementary Figure S6**. BLASTN against selected mature miRNA in miRBASE.

**Supplementary Figure S7**. Relative expression level of miRC103, miRC104, miRC106, miRC108, miRC116, miRC117, miRC122, miRC123 and miRC128 in *Grapevine rupestris stem pitting-associated virus* (GRSPaV) -free and infected 'Bosco' leaf as determined by qRT-PCR. Samples were collected under: well watered (WW), water stress (SW), severe water stress (SWS) conditions. qRT-PCR signals were normalised to U6 and 5.8 rRNA levels. Data are presented as mean ± standard deviation of three biological replicates; different letters denote significant differences at $p \leq 0.05$.

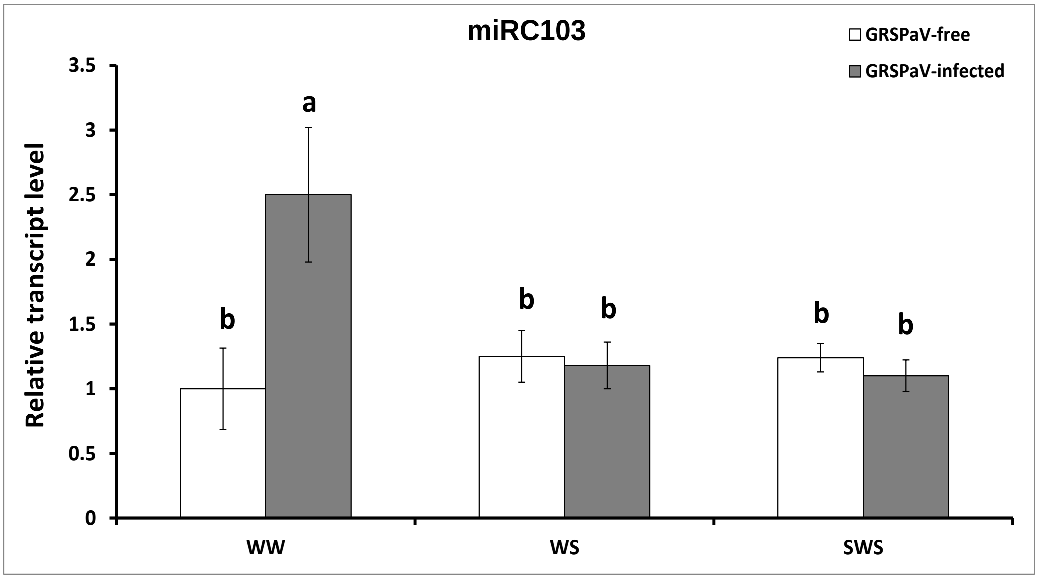


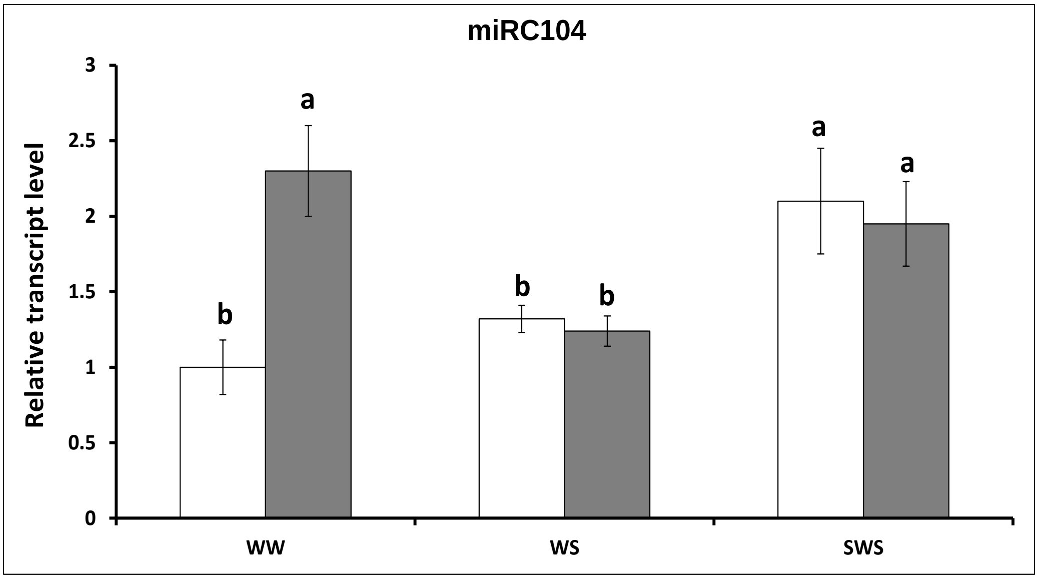

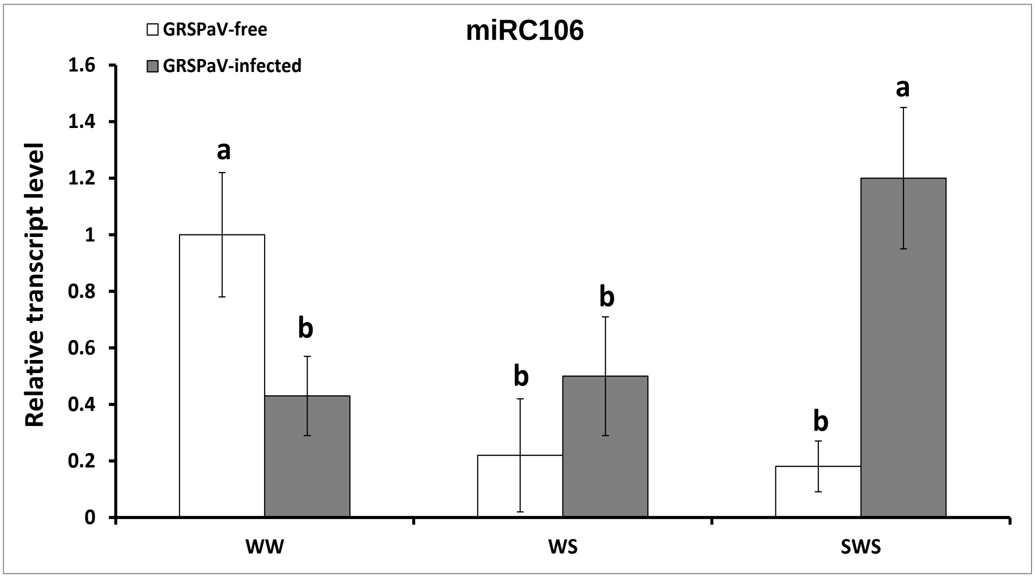

miRC106
GRSPaV-free
GRSPaV-infected
Relative transcript level
1.6
1.4
1.2
1
0.8
0.6
0.4
0.2
0
a
b
b
b
b
a
WW
WS
SWS


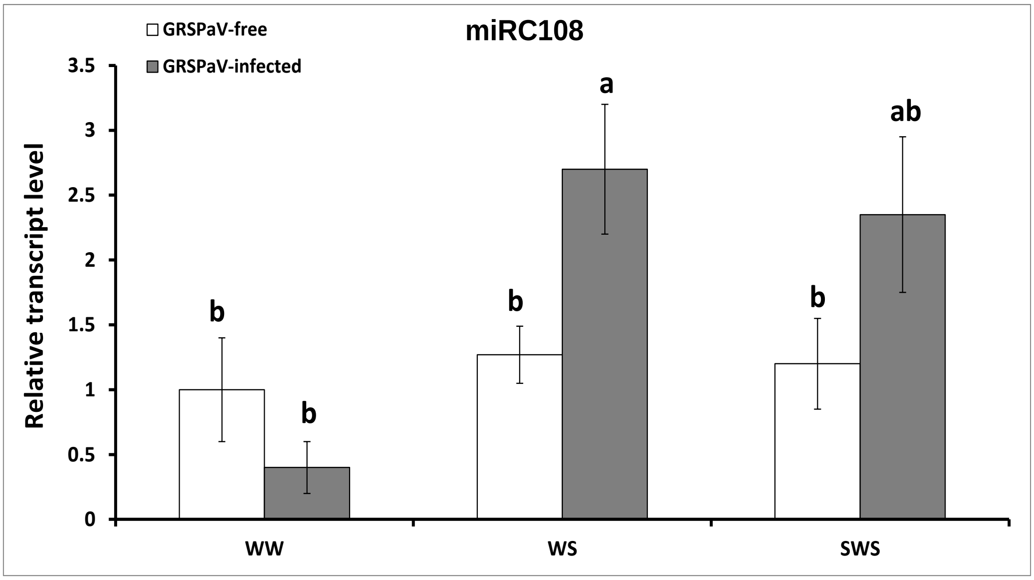

miRC108
GRSPaV-free
GRSPaV-infected
Relative transcript level
3.5
3
2.5
2
1.5
1
0.5
0
b
b
b
a
b
ab
WW
WS
SWS


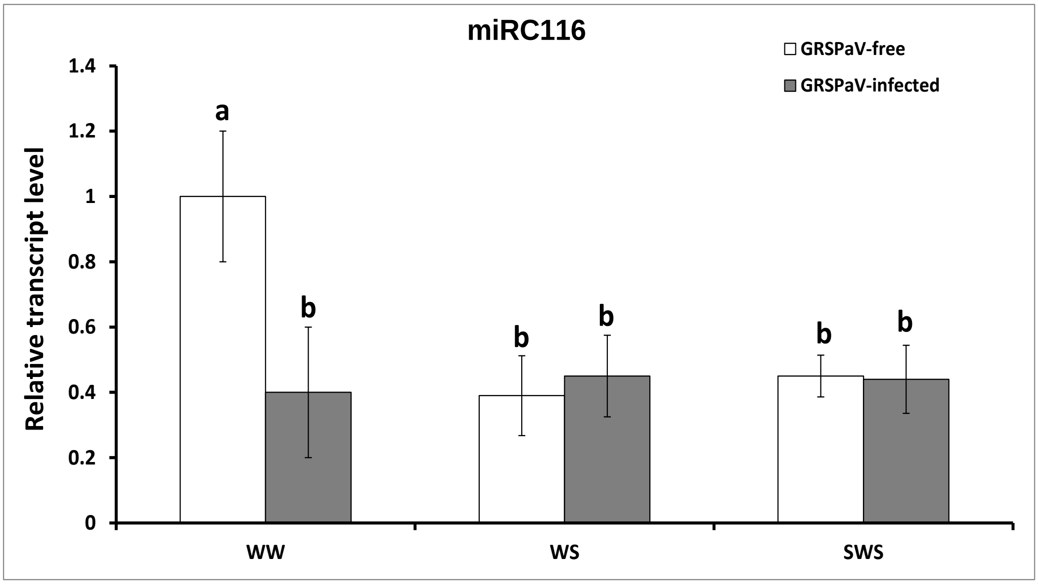

miRC116
GRSPaV-free
GRSPaV-infected
Relative transcript level
1.4
1.2
1
0.8
0.6
0.4
0.2
0
a
b
b
b
b
b
WW
WS
SWS

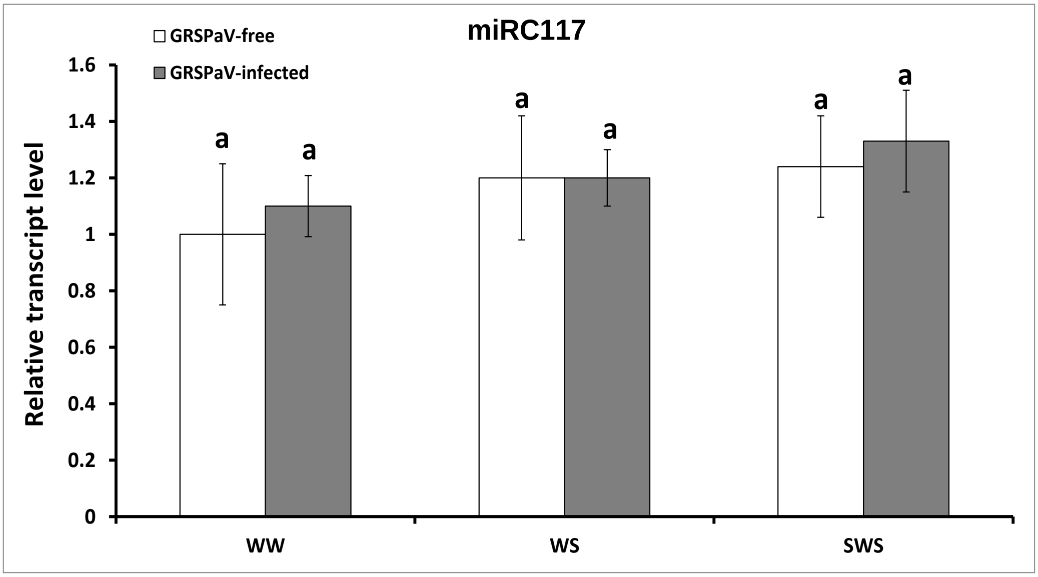

miRC117
GRSPaV-free
GRSPaV-infected
Relative transcript level
1.6
1.4
1.2
1
0.8
0.6
0.4
0.2
0
a
a
a
a
a
a
WW
WS
SWS


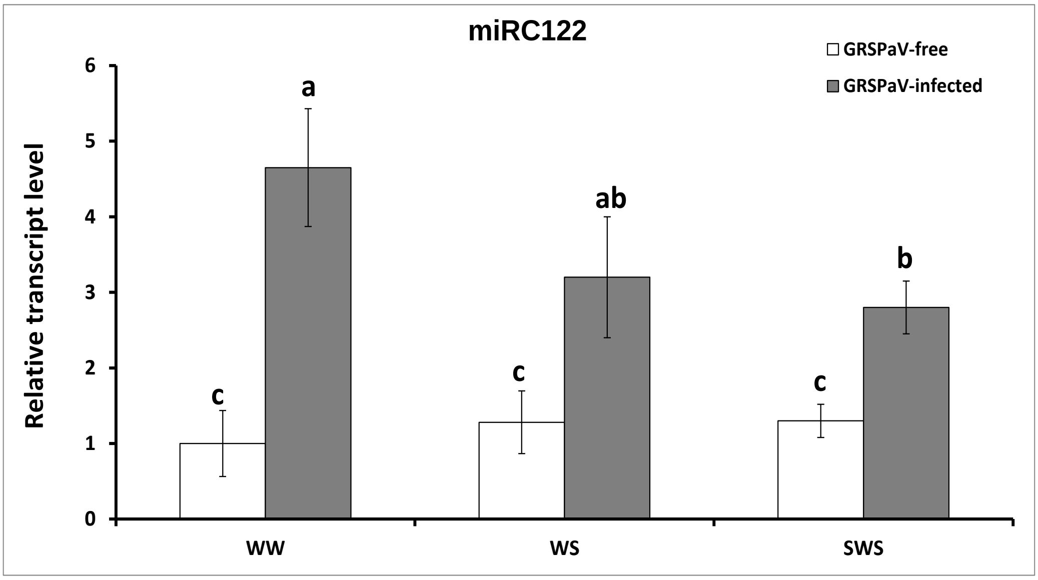

miRC122
GRSPaV-free
GRSPaV-infected
Relative transcript level
6
5
4
3
2
1
0
c
a
c
ab
c
b
WW
WS
SWS


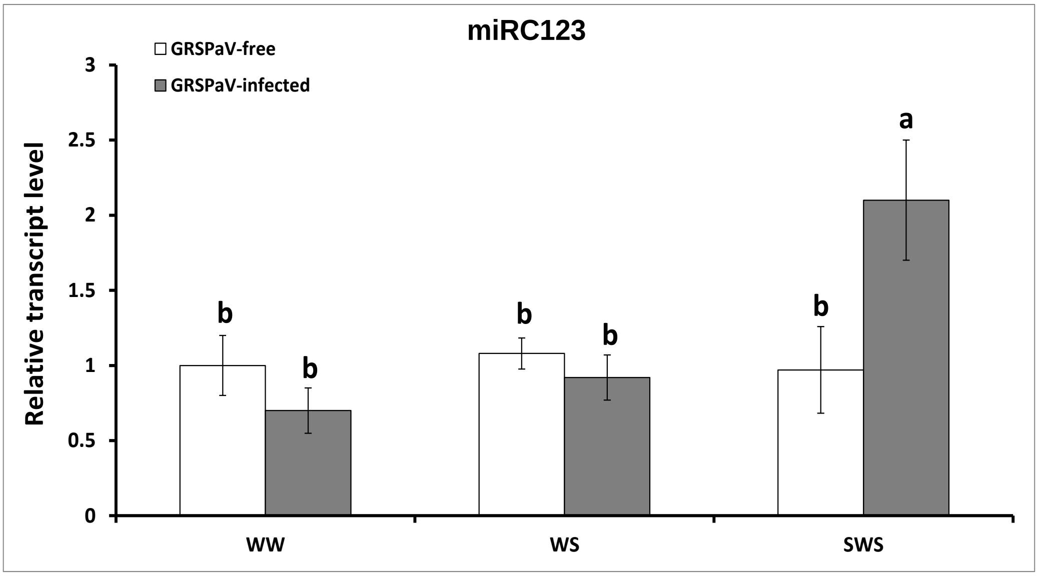

miRC123
GRSPaV-free
GRSPaV-infected
Relative transcript level
3
2.5
2
1.5
1
0.5
0
b
b
b
b
b
a
WW
WS
SWS

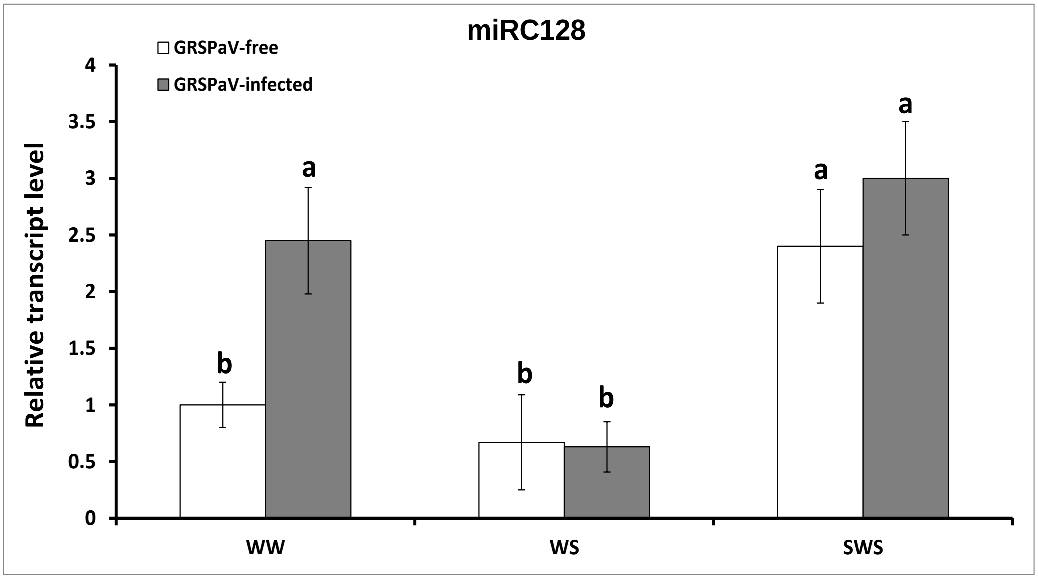
miRC128
GRSPaV-free
GRSPaV-infected
Relative transcript level
4
3.5
3
2.5
2
1.5
1
0.5
0
a
b
a
b
b
a
WW
WS
SWS

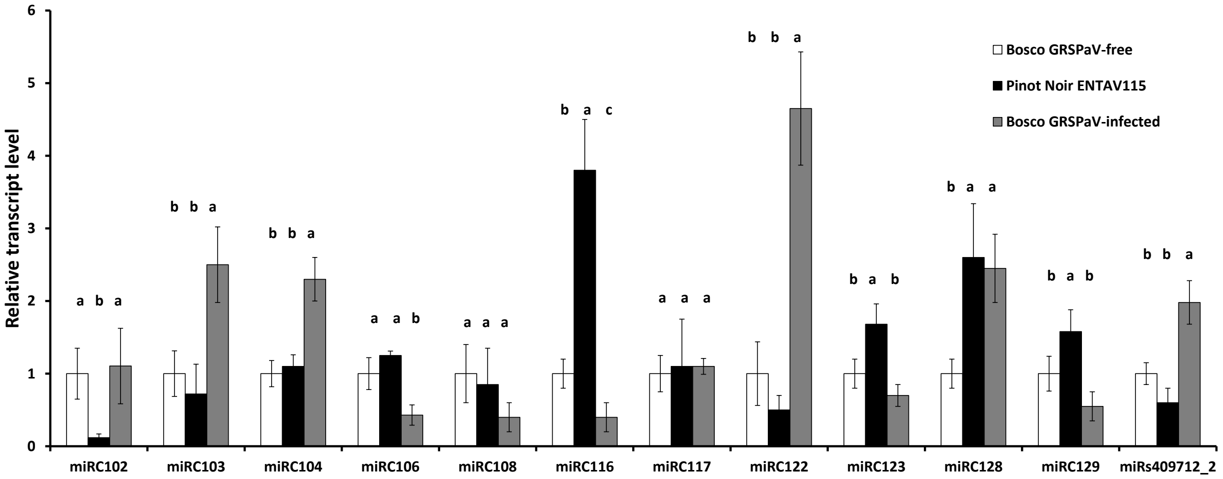


**Supplementary Figure S8.** Relative expression levels of all novel miRNA candidates in *Grapevine rupestris stem pitting-associated* (GRSPaV)-free, GRSPaV-infected 'Bosco' leaves and in 'Pinot noir' ENTAV115 leaves as determined by qRT-PCR. Samples were collected under well watered (WW) conditions. qRT-PCR signals were normalised to U6 and 5.8 levels. Data are presented as mean ± standard deviation of three biological replicates; different letters denote significant differences at $p \leq 0.05$.

**Supplementary Figure S9. T-plots for targets of grapevine-specific microRNAs (miRNAs).**

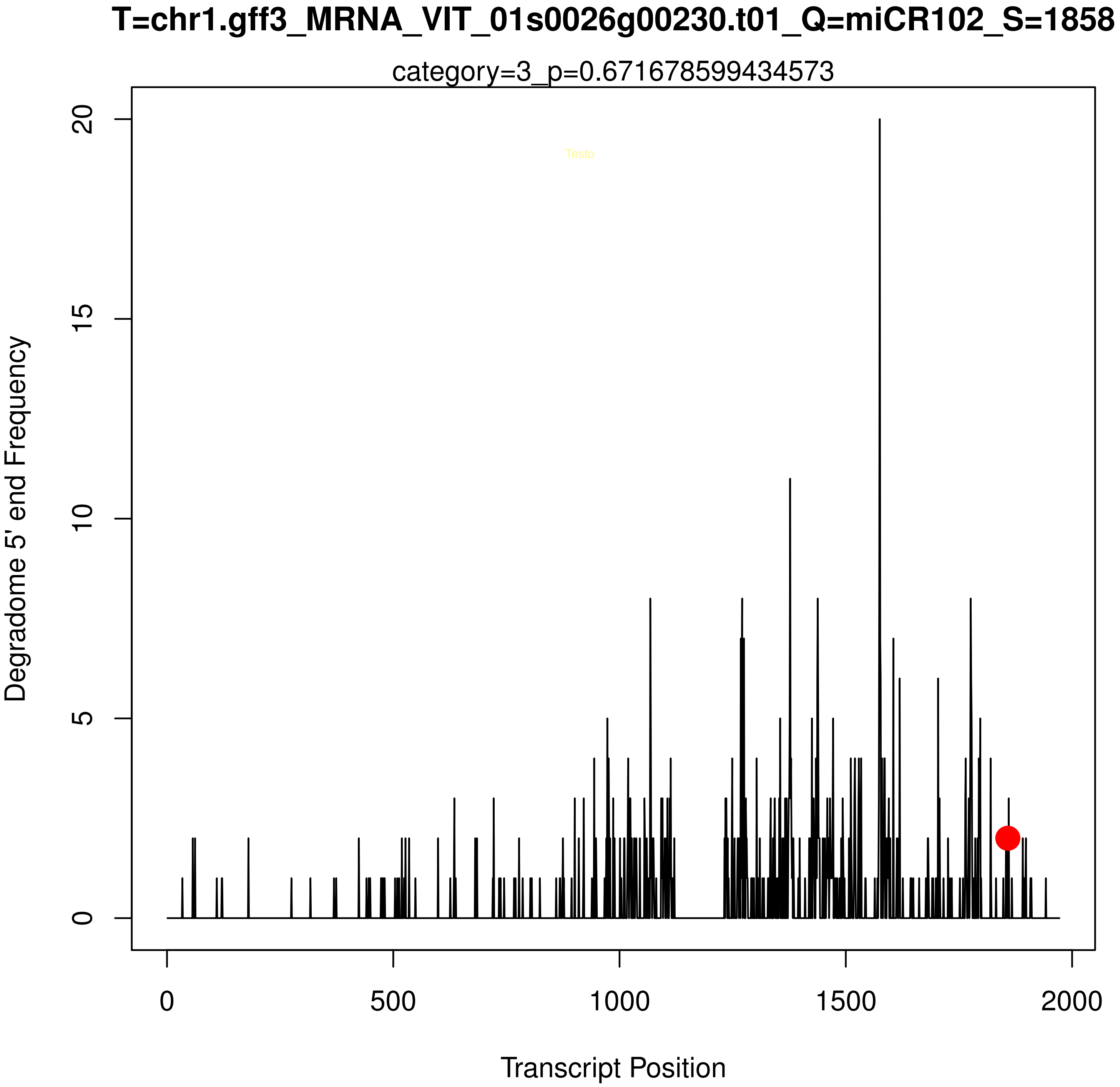

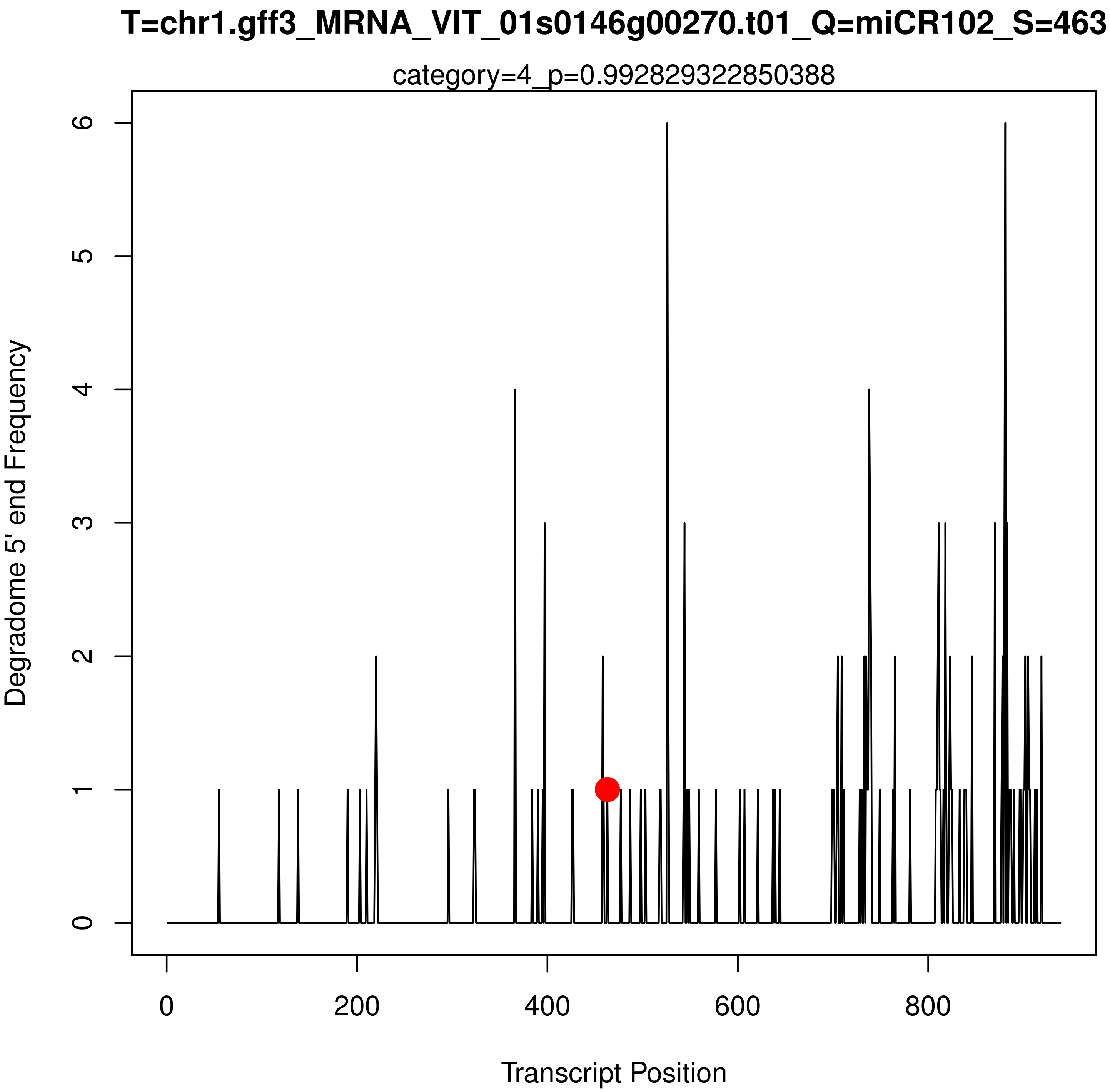
T=chr1.gff3_MRNA_VIT_01s0146g00270.t01_Q=miCR102_S=463
category=4_p=0.992829322850388
Degradome 5' end Frequency
Transcript Position
0
1
2
3
4
5
6
0
200
400
600
800

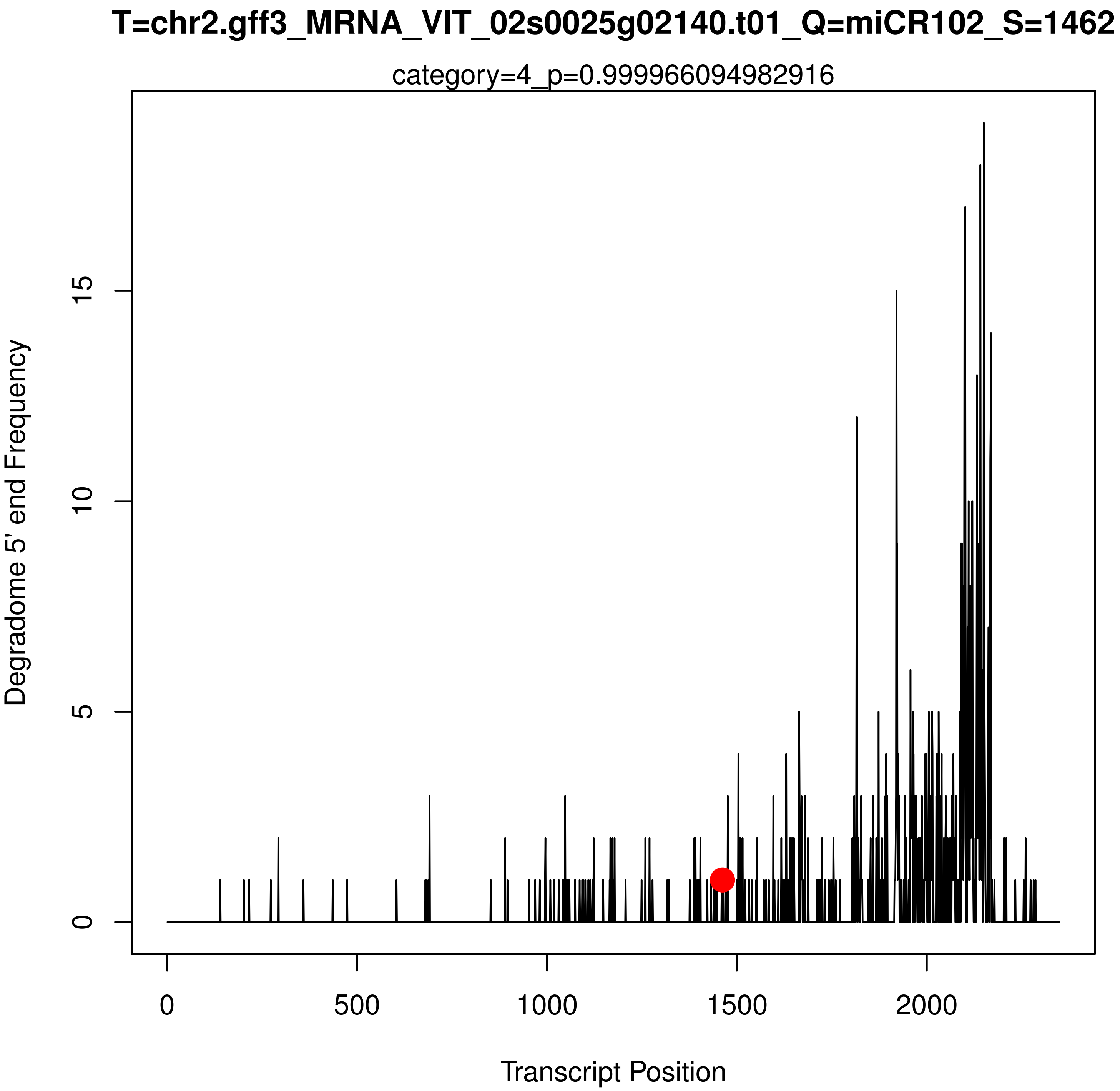
T=chr2.gff3_MRNA_VIT_02s0025g02140.t01_Q=miCR102_S=1462
category=4_p=0.999966094982916
Degradome 5' end Frequency
0
5
10
15
0
500
1000
1500
2000
Transcript Position

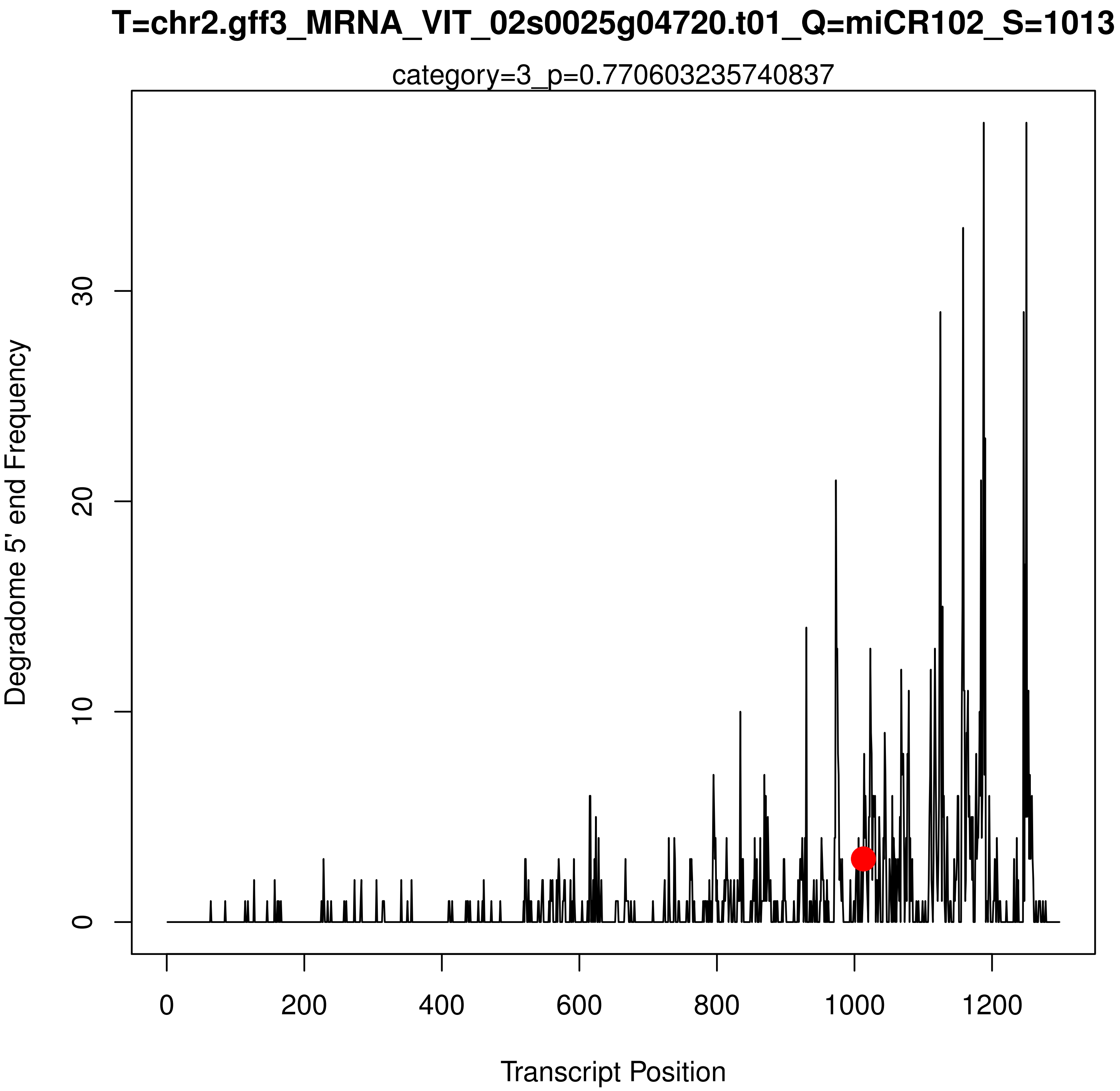

T=chr2.gff3_MRNA_VIT_02s0025g04720.t01_Q=miCR102_S=1013
category=3_p=0.770603235740837
Degradome 5' end Frequency
0
10
20
30
0
200
400
600
800
1000
1200
Transcript Position

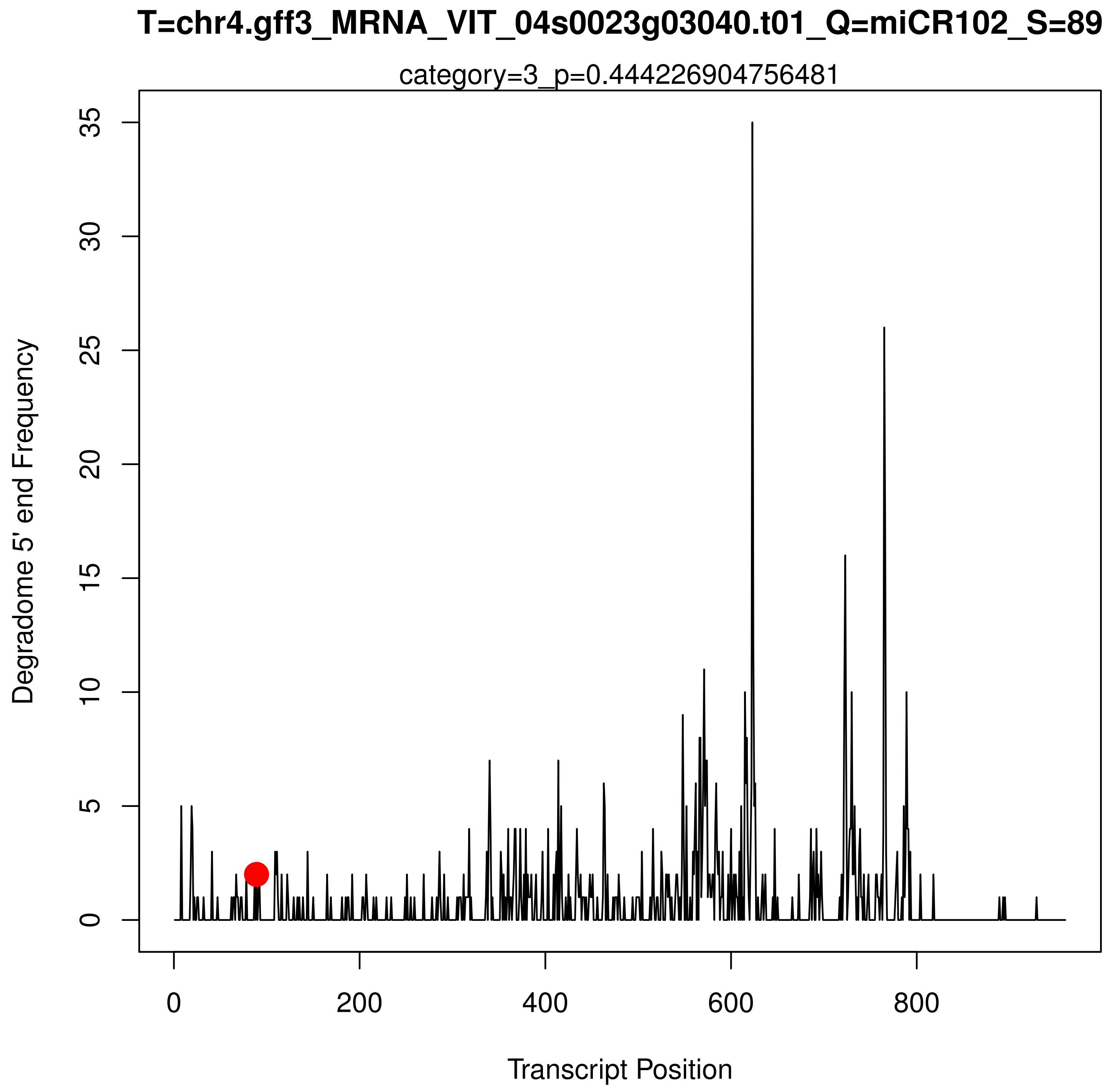
T=chr4.gff3_MRNA_VIT_04s0023g03040.t01_Q=miCR102_S=89
category=3_p=0.444226904756481
Degradome 5' end Frequency
Transcript Position
0
5
10
15
20
25
30
35
0
200
400
600
800

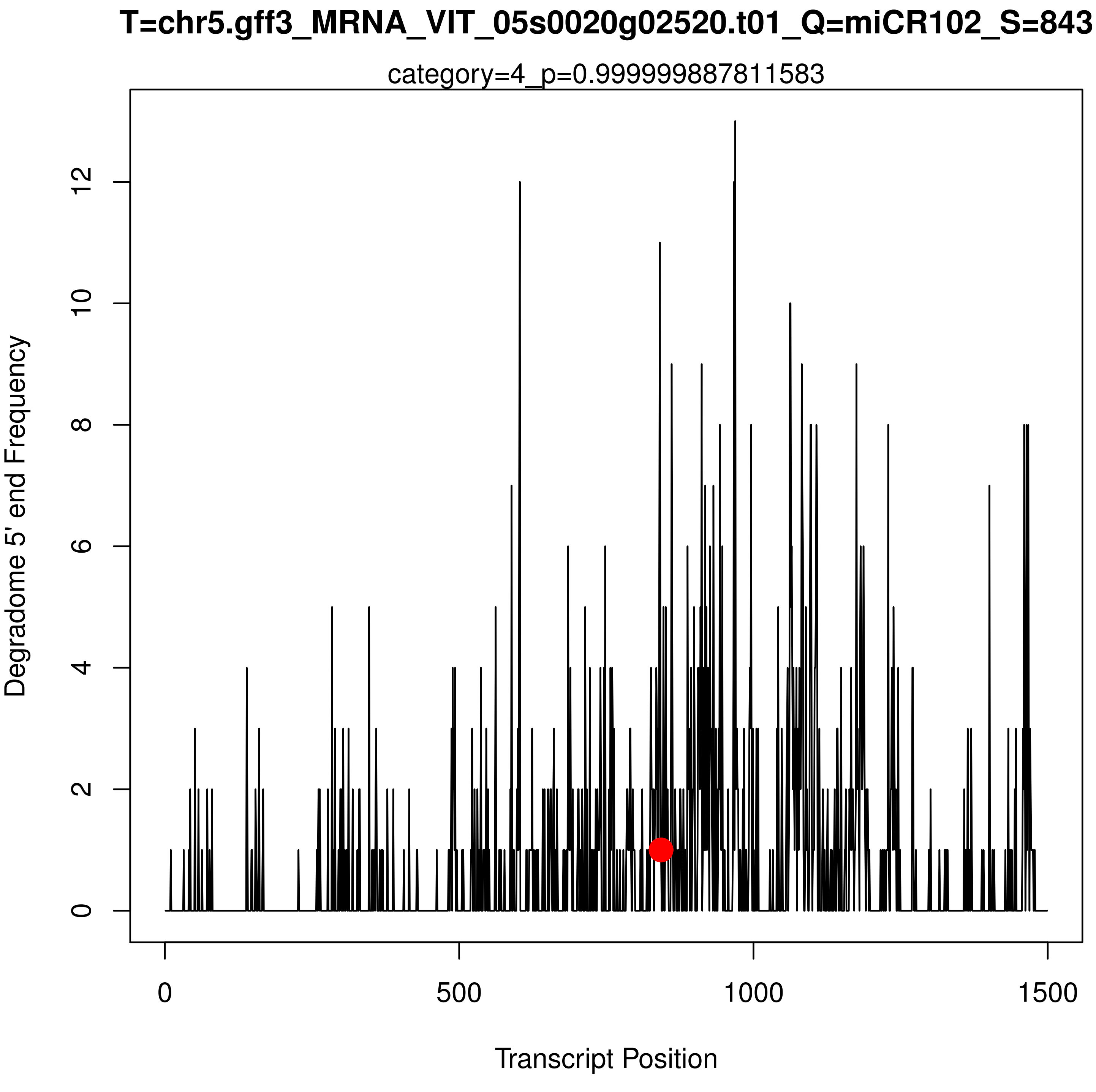
T=chr5.gff3_MRNA_VIT_05s0020g02520.t01_Q=miCR102_S=843
category=4_p=0.999999887811583
Degradome 5' end Frequency
Transcript Position
0
2
4
6
8
10
12
0
500
1000
1500

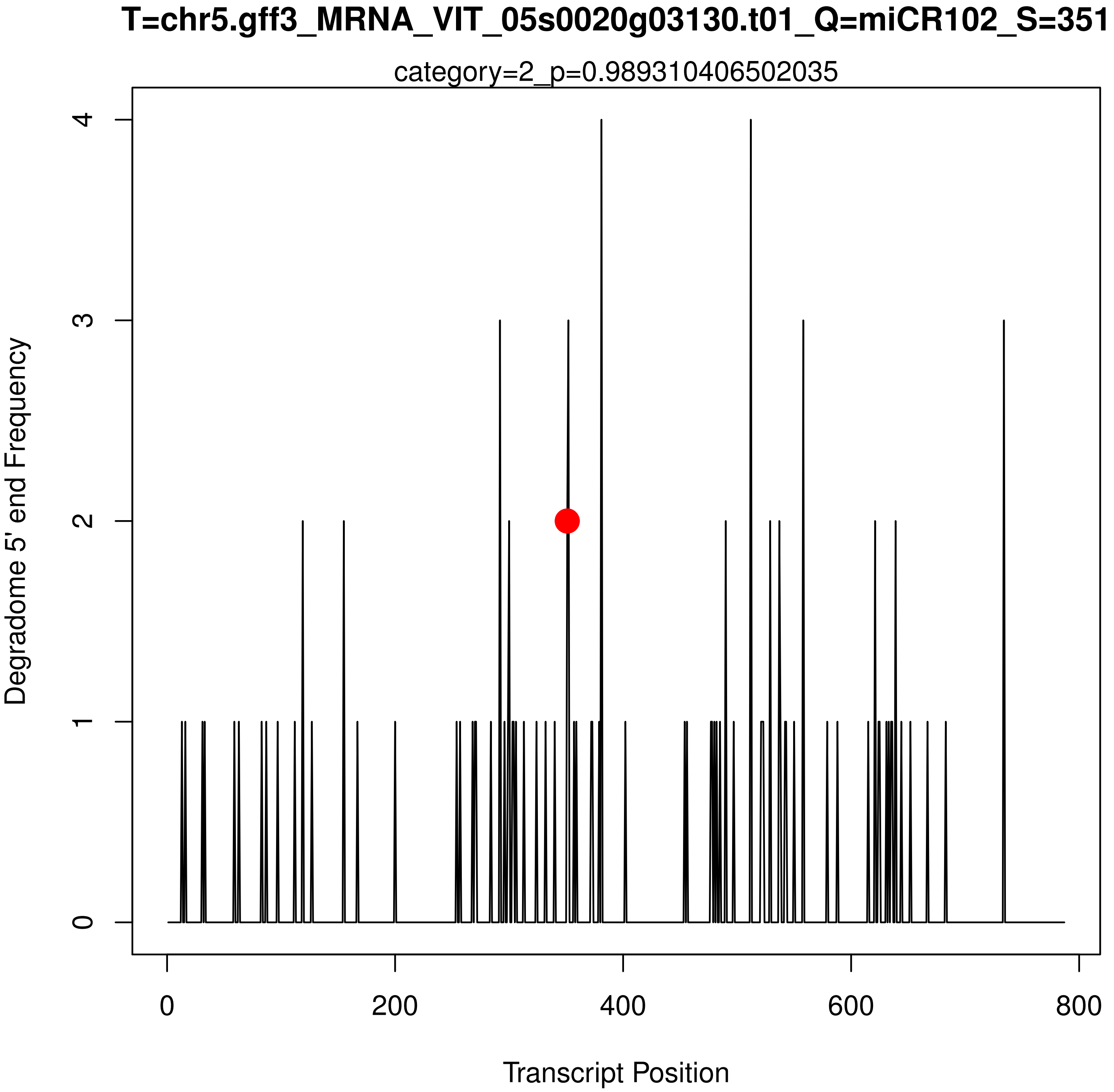

T=chr5.gff3_MRNA_VIT_05s0020g03130.t01_Q=miCR102_S=351
category=2_p=0.989310406502035
Degradome 5' end Frequency
0
1
2
3
4
0
200
400
600
800
Transcript Position

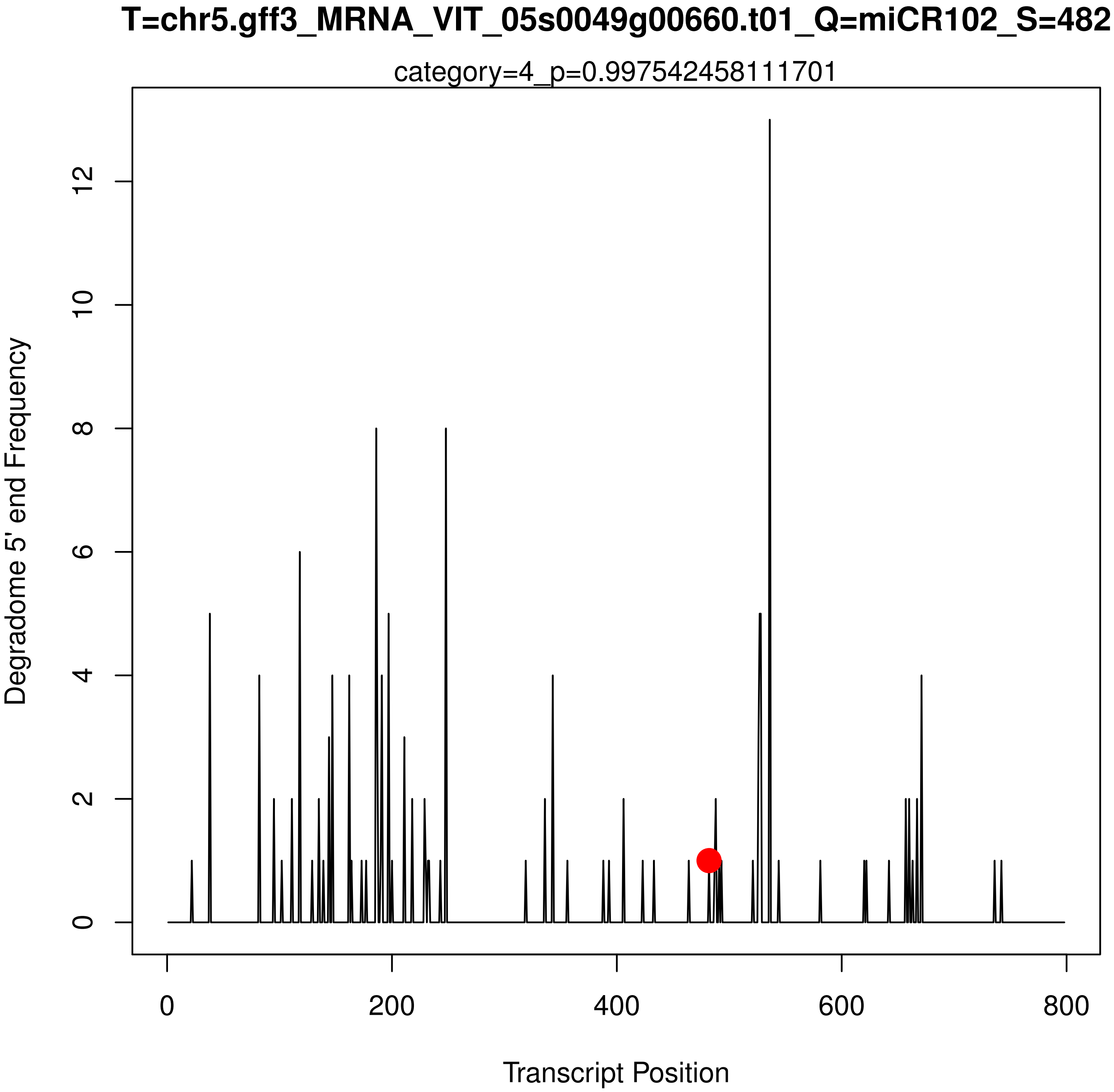

T=chr5.gff3_MRNA_VIT_05s0049g00660.t01_Q=miCR102_S=482
category=4_p=0.997542458111701
Degradome 5' end Frequency
0
2
4
6
8
10
12
0
200
400
600
800
Transcript Position

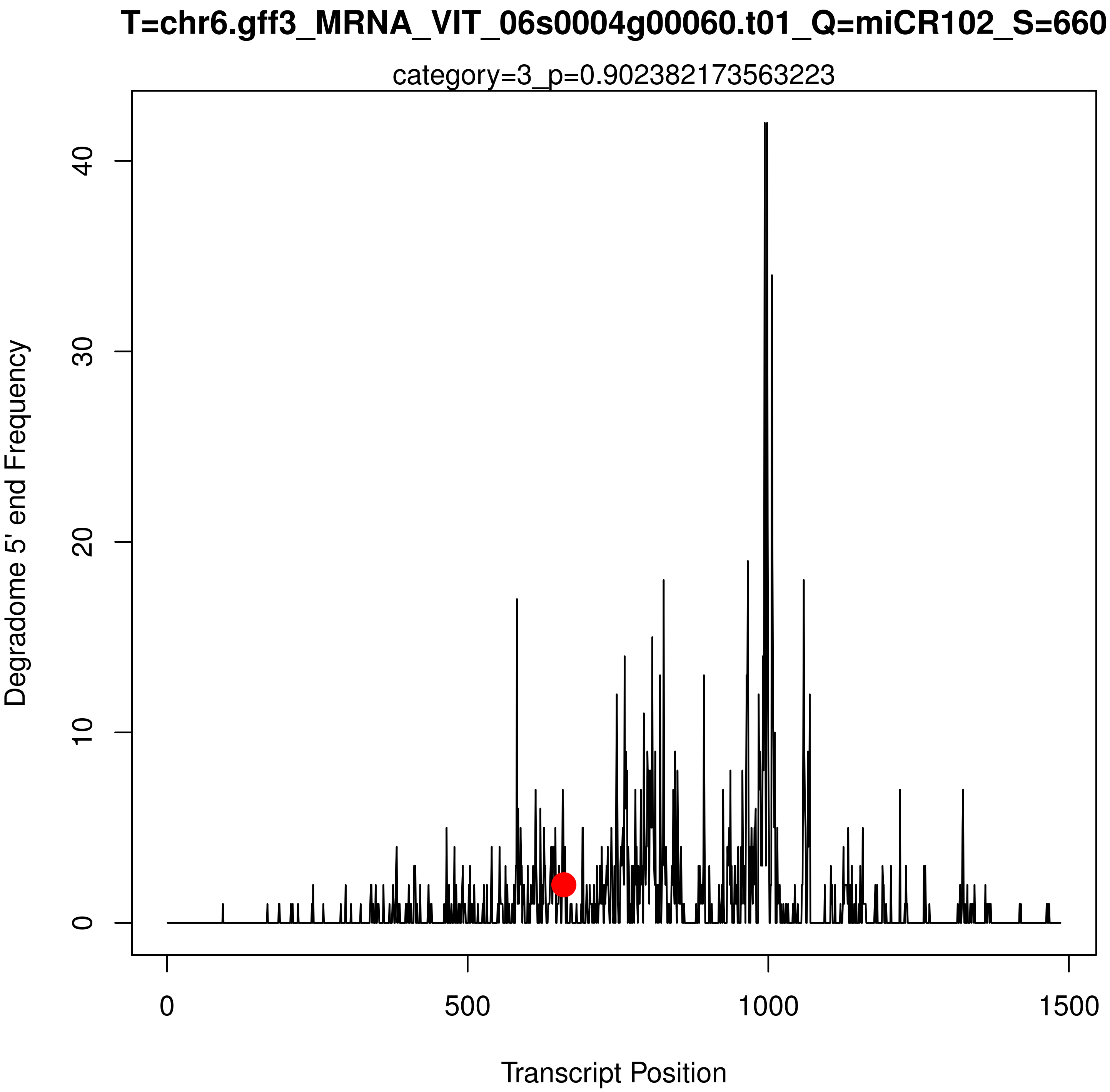
T=chr6.gff3_MRNA_VIT_06s0004g00060.t01_Q=miCR102_S=660
category=3_p=0.902382173563223
Degradome 5' end Frequency
0
10
20
30
40
0
500
1000
1500
Transcript Position

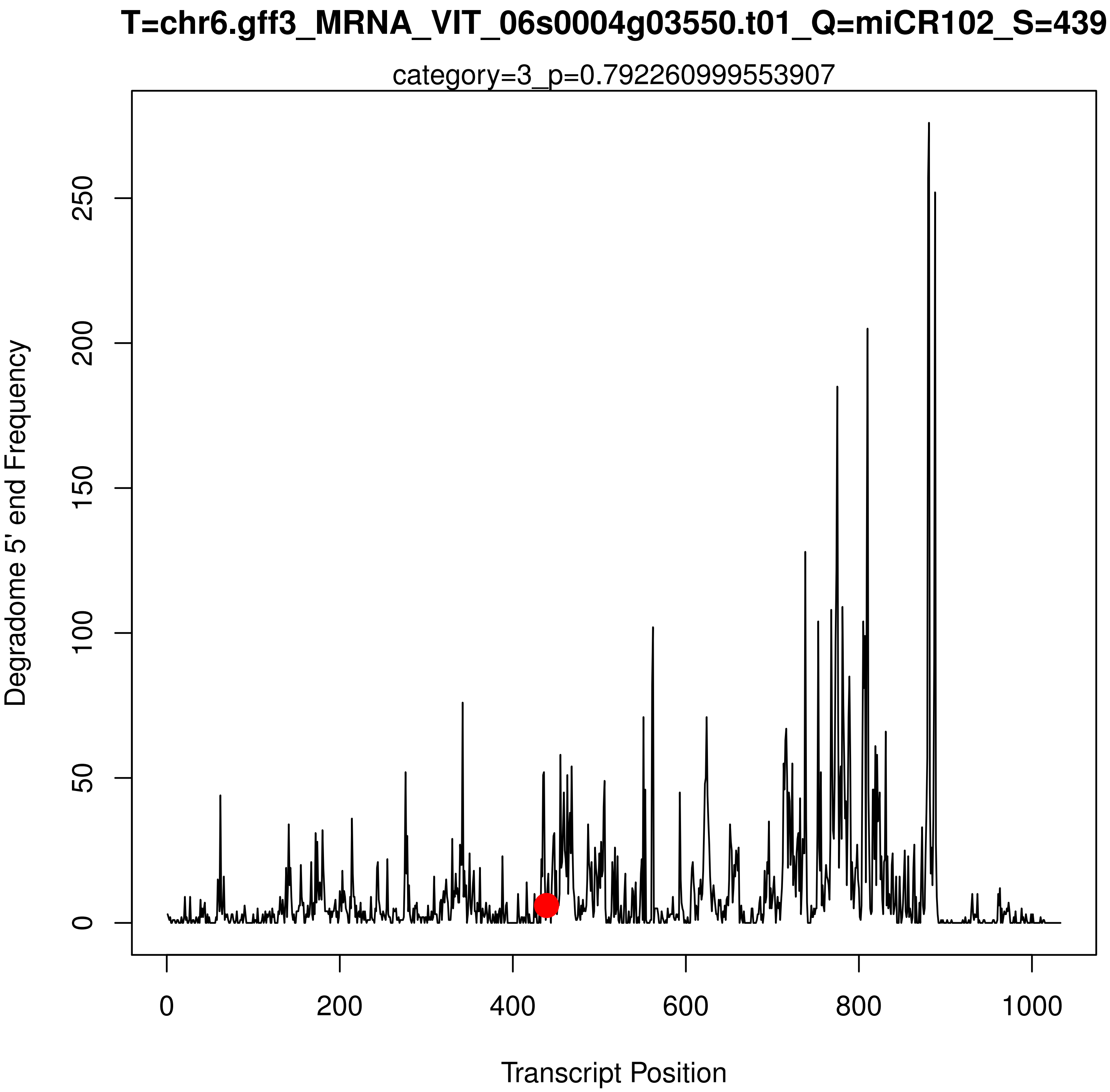
T=chr6.gff3_MRNA_VIT_06s0004g03550.t01_Q=miCR102_S=439
category=3_p=0.792260999553907
Degradome 5' end Frequency
0
50
100
150
200
250
0
200
400
600
800
1000
Transcript Position

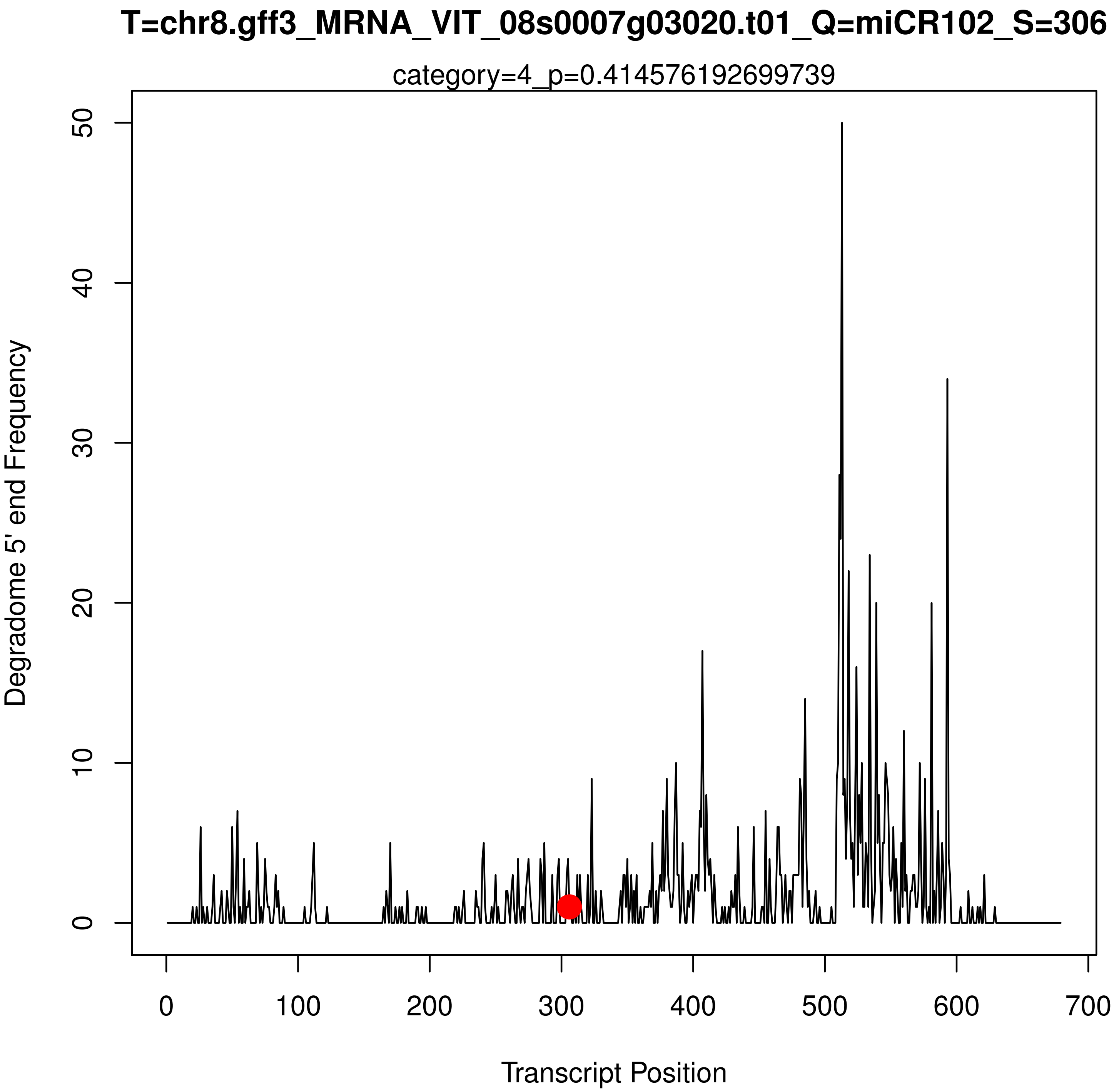
T=chr8.gff3_MRNA_VIT_08s0007g03020.t01_Q=miCR102_S=306
category=4_p=0.414576192699739
Degradome 5' end Frequency
0
10
20
30
40
50
0
100
200
300
400
500
600
700
Transcript Position

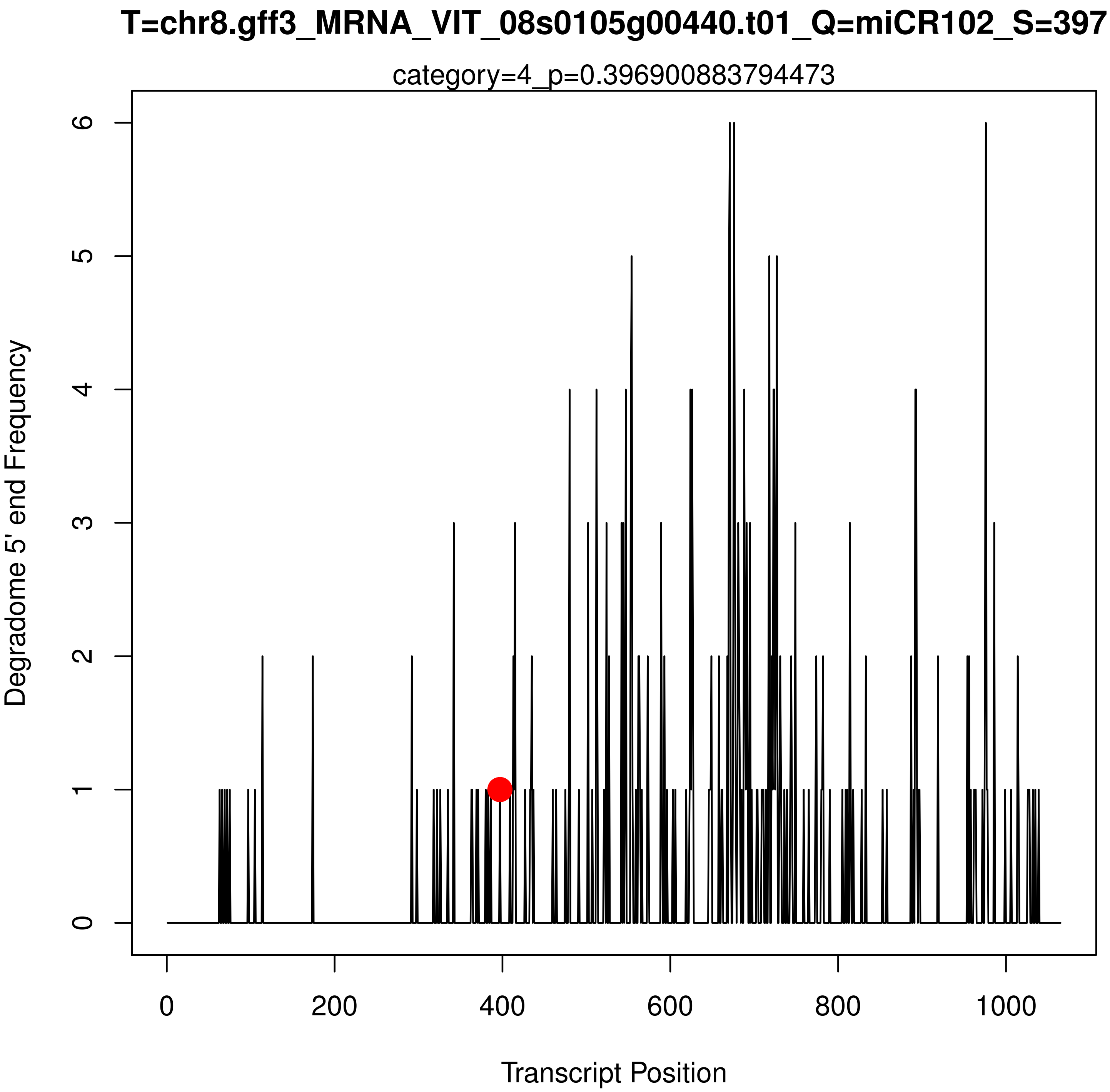

T=chr8.gff3_MRNA_VIT_08s0105g00440.t01_Q=miCR102_S=397
category=4_p=0.396900883794473
Degradome 5' end Frequency
0
1
2
3
4
5
6
0
200
400
600
800
1000
Transcript Position

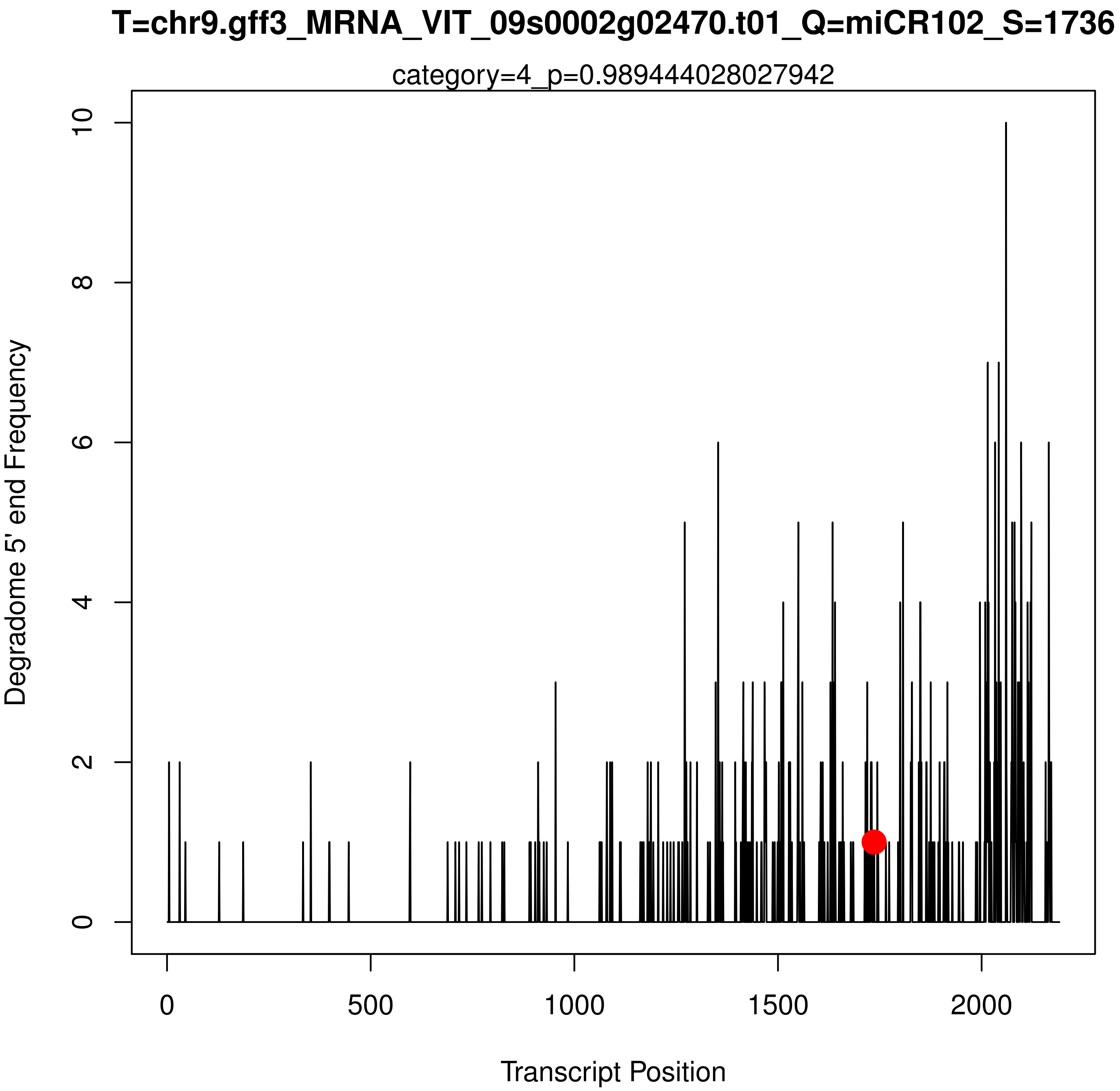
T=chr9.gff3_MRNA_VIT_09s0002g02470.t01_Q=miCR102_S=1736
category=4_p=0.989444028027942
Degradome 5' end Frequency
0
2
4
6
8
10
0
500
1000
1500
2000
Transcript Position

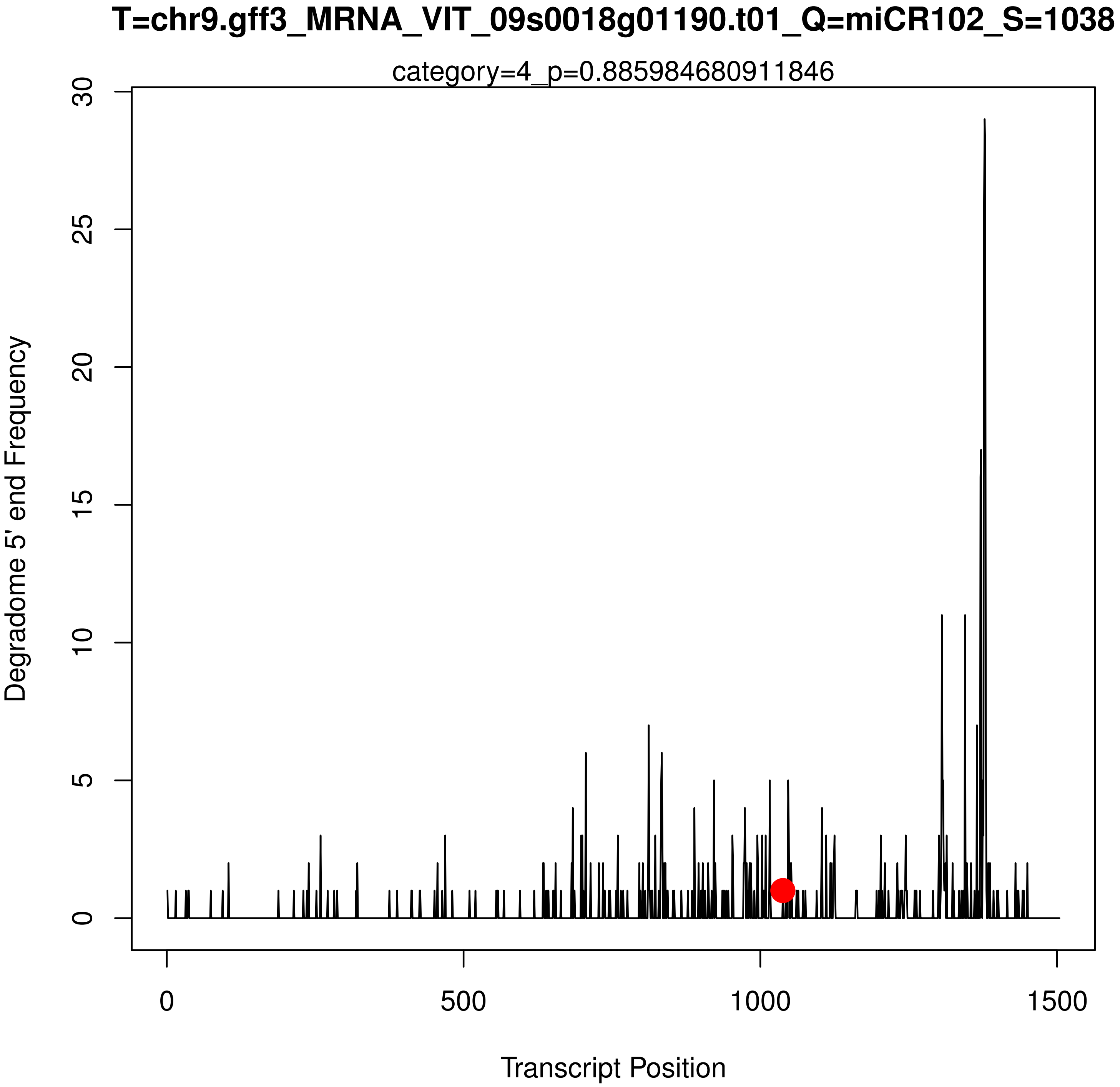

T=chr9.gff3_MRNA_VIT_09s0018g01190.t01_Q=miCR102_S=1038
category=4_p=0.885984680911846
Degradome 5' end Frequency
Transcript Position
0
5
10
15
20
25
30
0
500
1000
1500

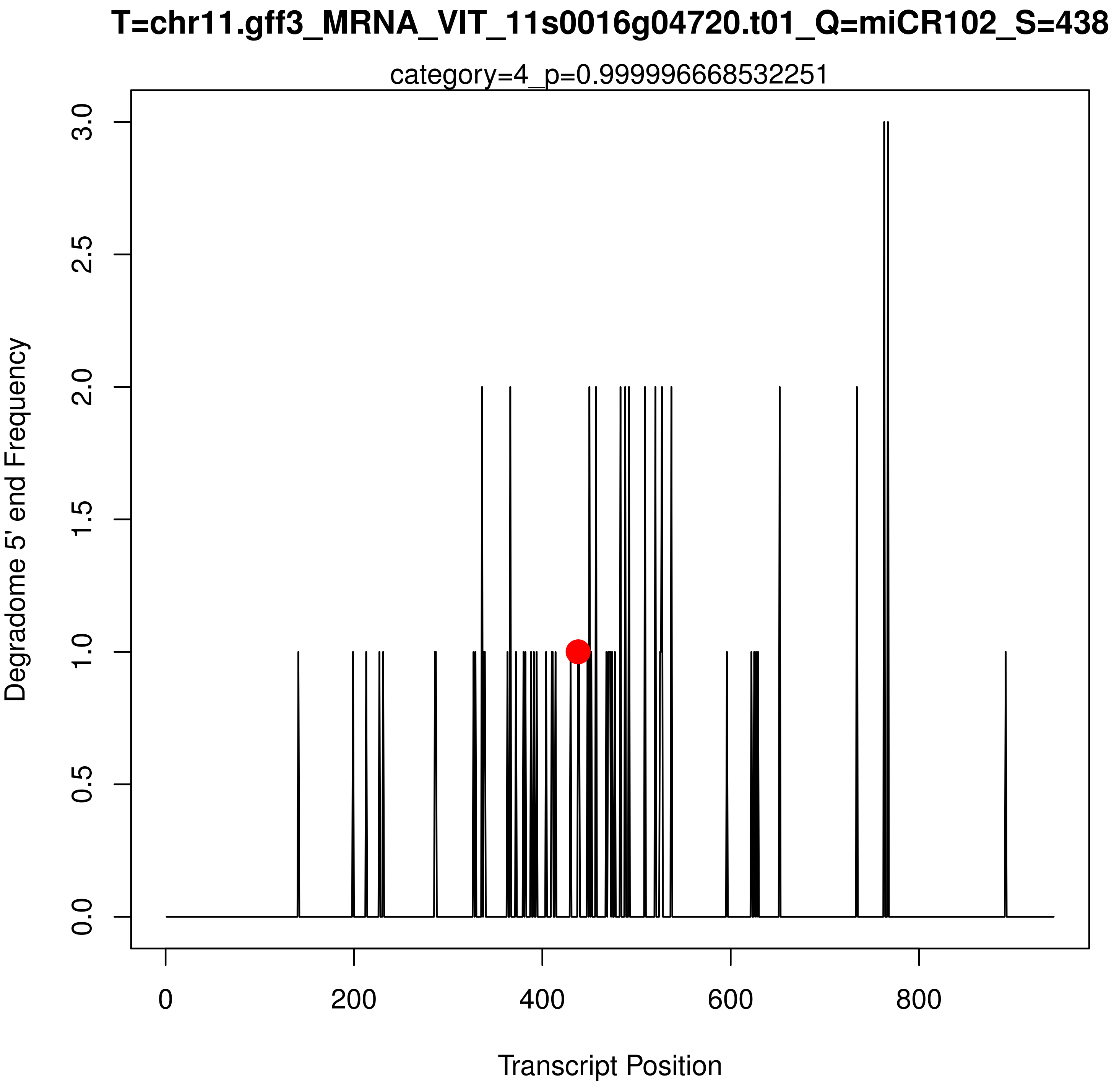
T=chr11.gff3_MRNA_VIT_11s0016g04720.t01_Q=miCR102_S=438
category=4_p=0.999996668532251
Degradome 5' end Frequency
0.0
0.5
1.0
1.5
2.0
2.5
3.0
0
200
400
600
800
Transcript Position

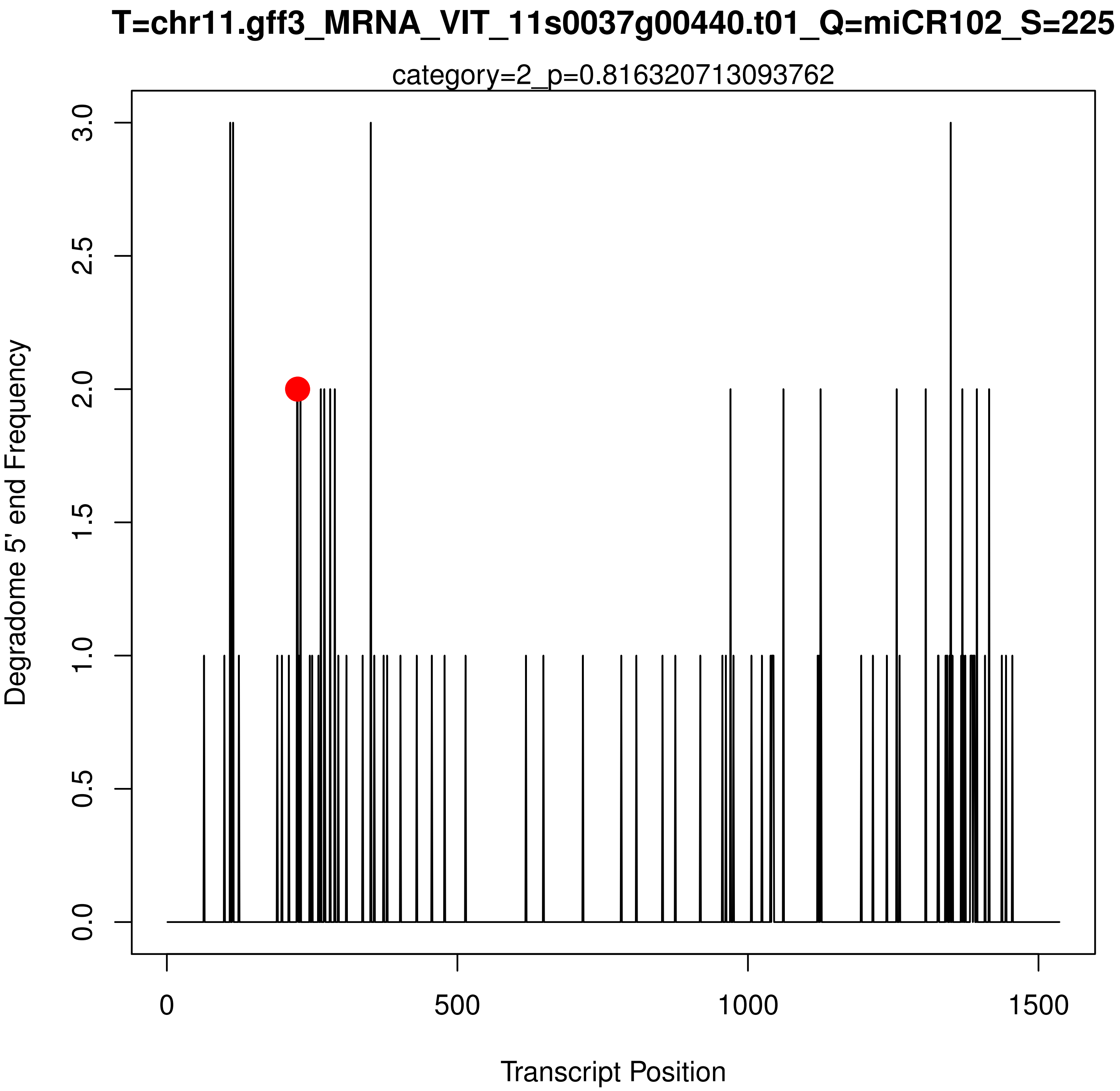

T=chr11.gff3_MRNA_VIT_11s0037g00440.t01_Q=miCR102_S=225
category=2_p=0.816320713093762
Degradome 5' end Frequency
Transcript Position
0.0
0.5
1.0
1.5
2.0
2.5
3.0
0
500
1000
1500

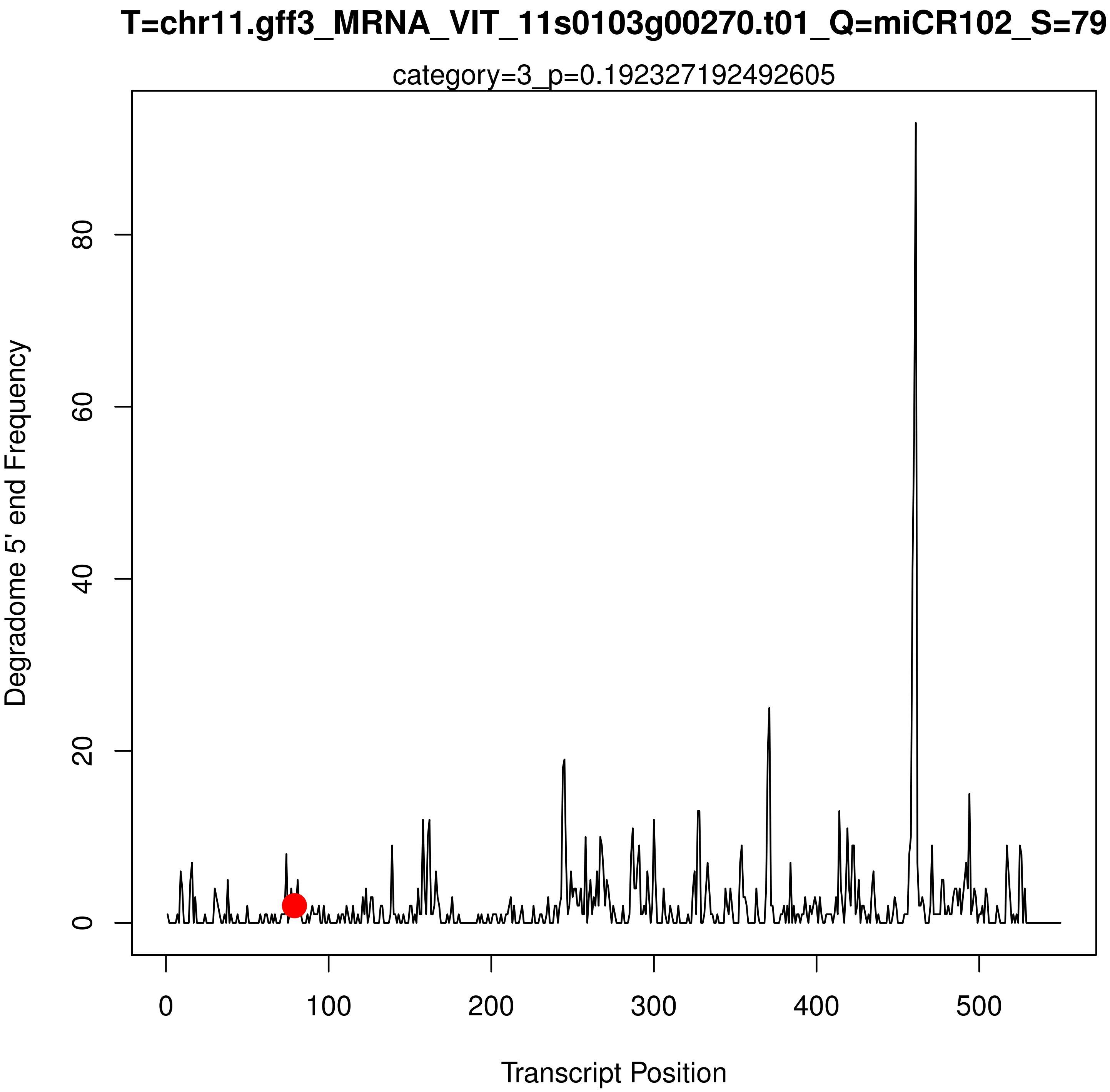
T=chr11.gff3_MRNA_VIT_11s0103g00270.t01_Q=miCR102_S=79
category=3_p=0.192327192492605
Degradome 5' end Frequency
0
20
40
60
80
0
100
200
300
400
500
Transcript Position

# T=chr12.gff3_MRNA_VIT_12s0028g02170.t01_Q=miCR102_S=820

category=2_p=0.989466766454178

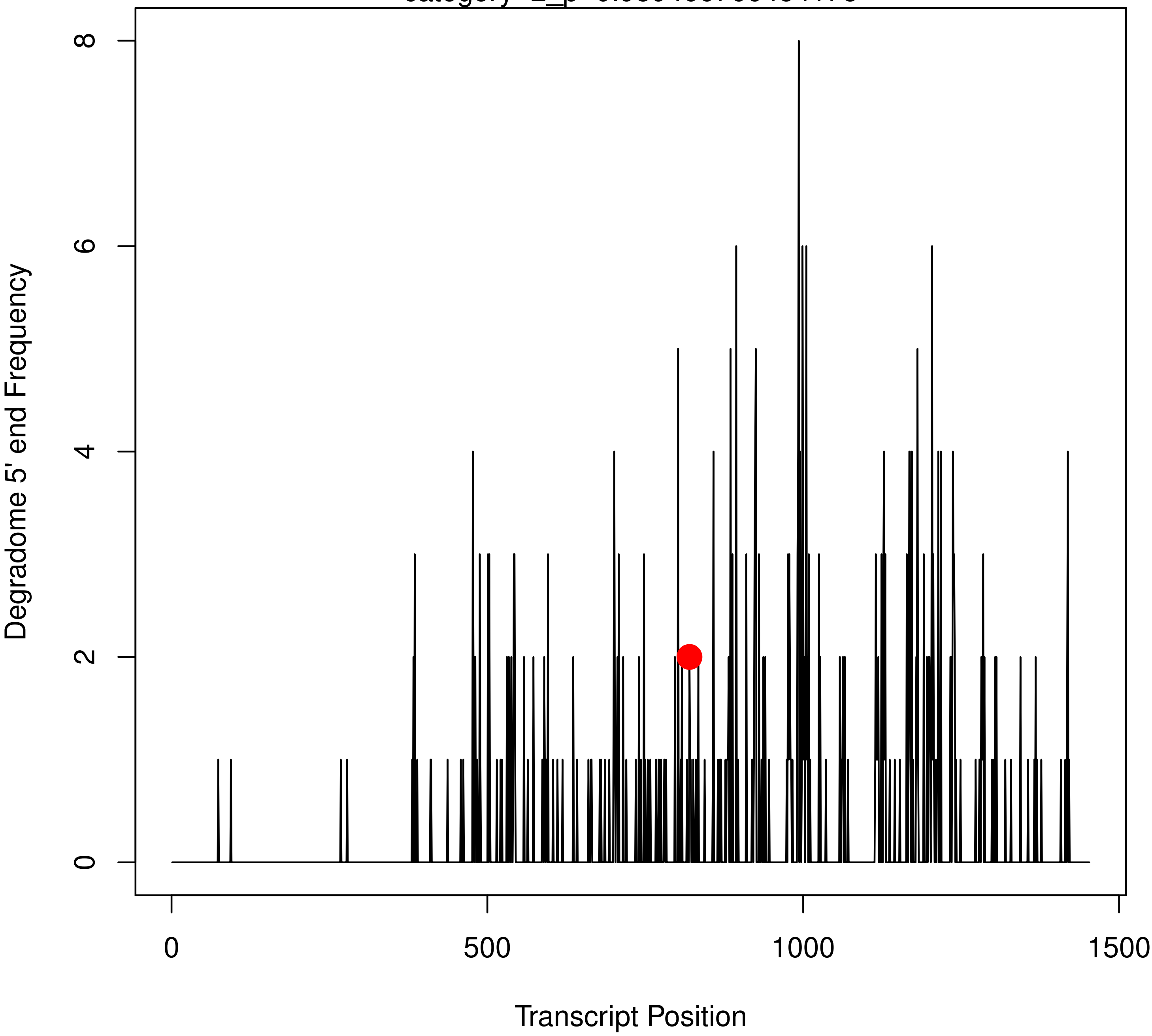

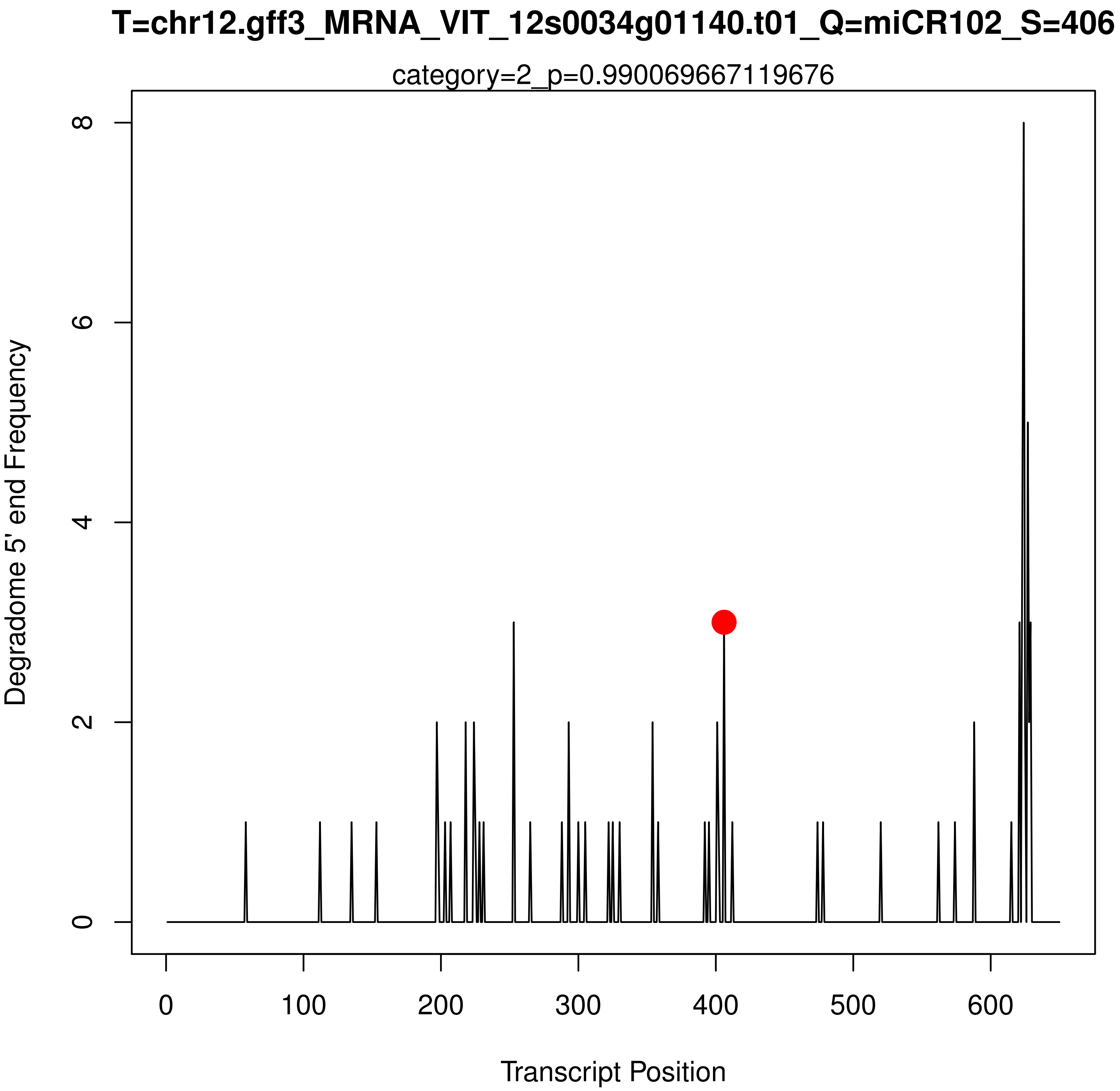
T=chr12.gff3_MRNA_VIT_12s0034g01140.t01_Q=miCR102_S=406
category=2_p=0.990069667119676
Degradome 5' end Frequency
0
2
4
6
8
0
100
200
300
400
500
600
Transcript Position

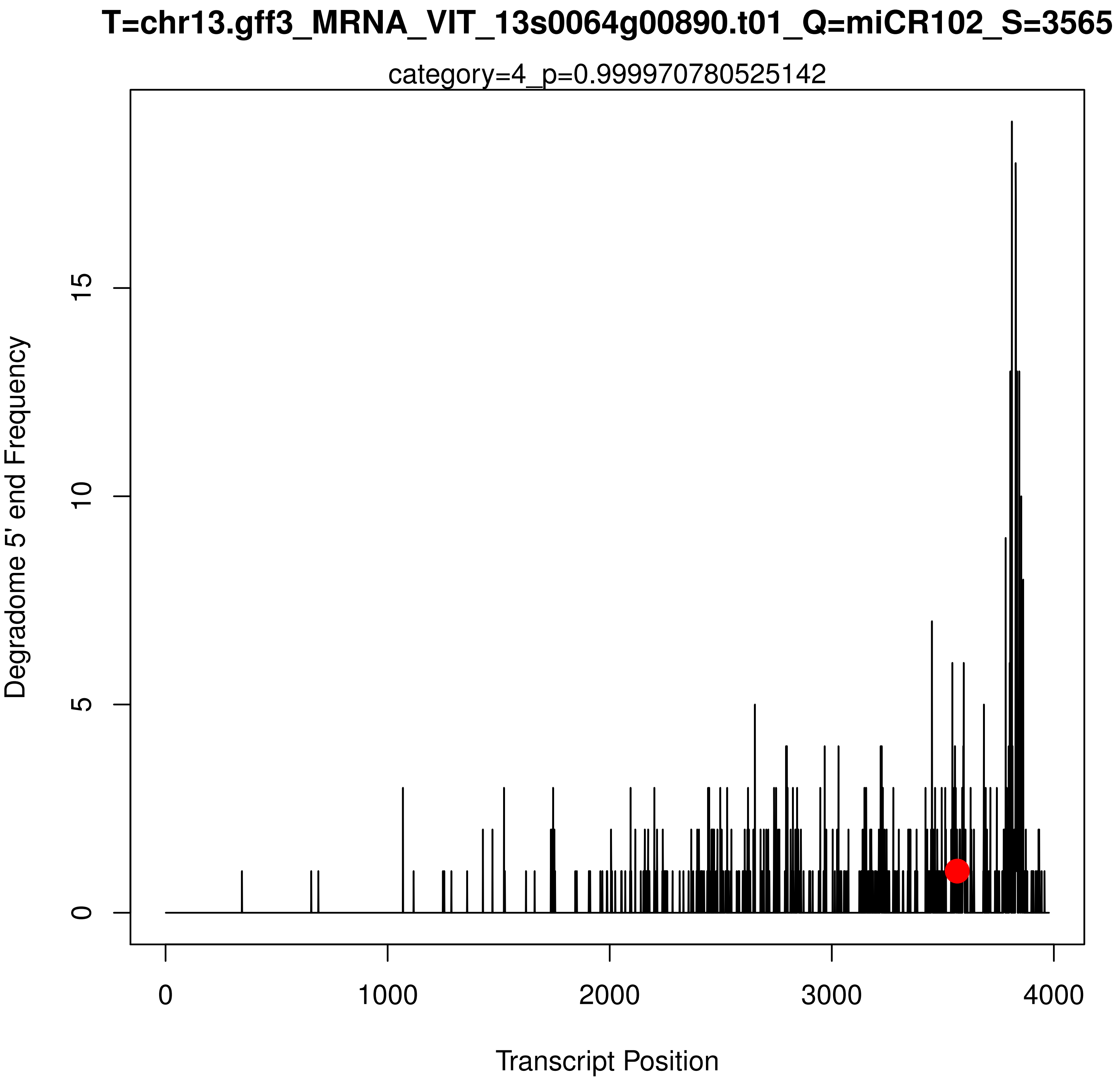
T=chr13.gff3_MRNA_VIT_13s0064g00890.t01_Q=miCR102_S=3565
category=4_p=0.999970780525142
Degradome 5' end Frequency
0
5
10
15
0
1000
2000
3000
4000
Transcript Position

**T=chr13.gff3_MRNA_VIT_13s0067g01070.t01_Q=miCR102_S=2016**

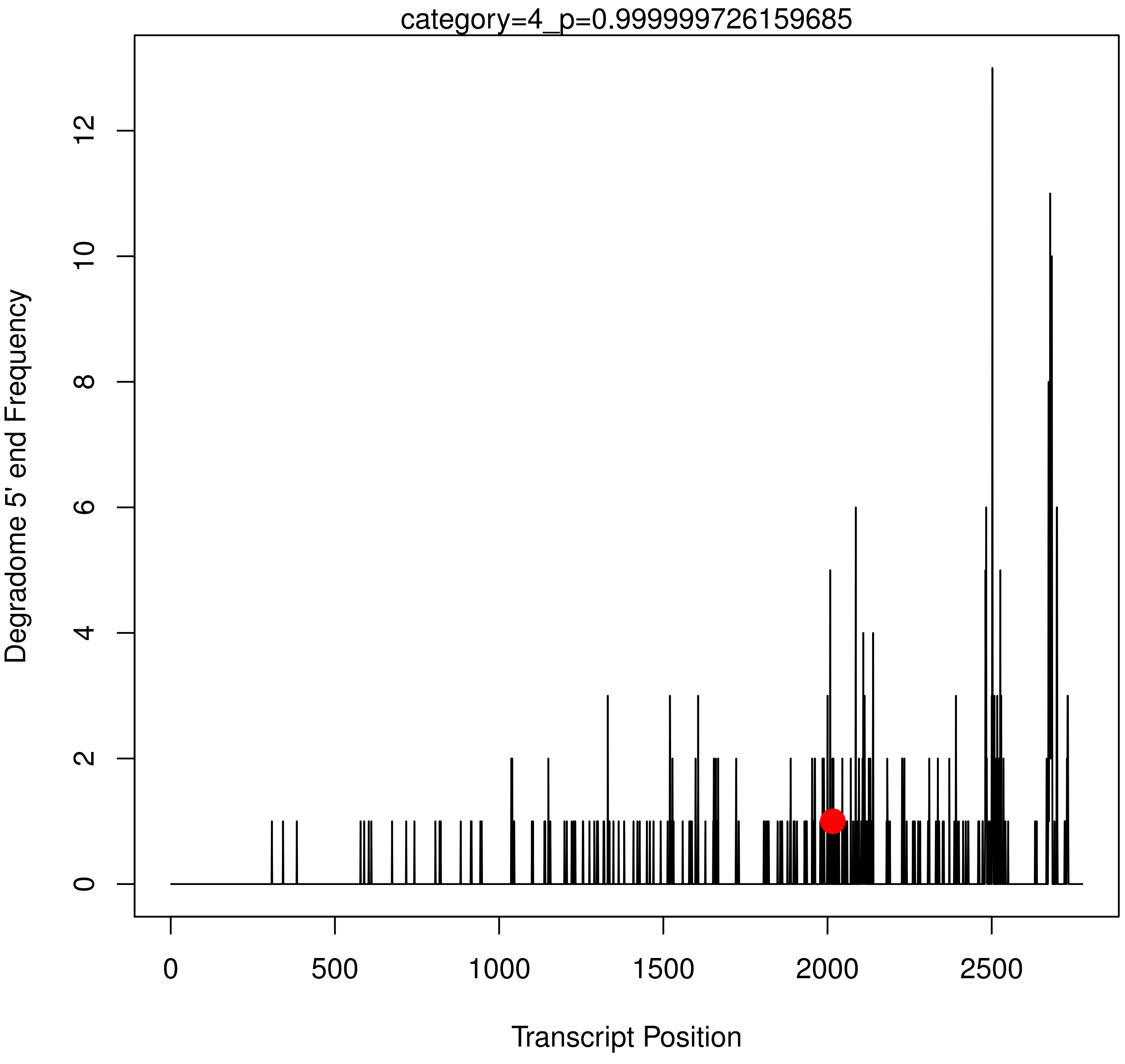

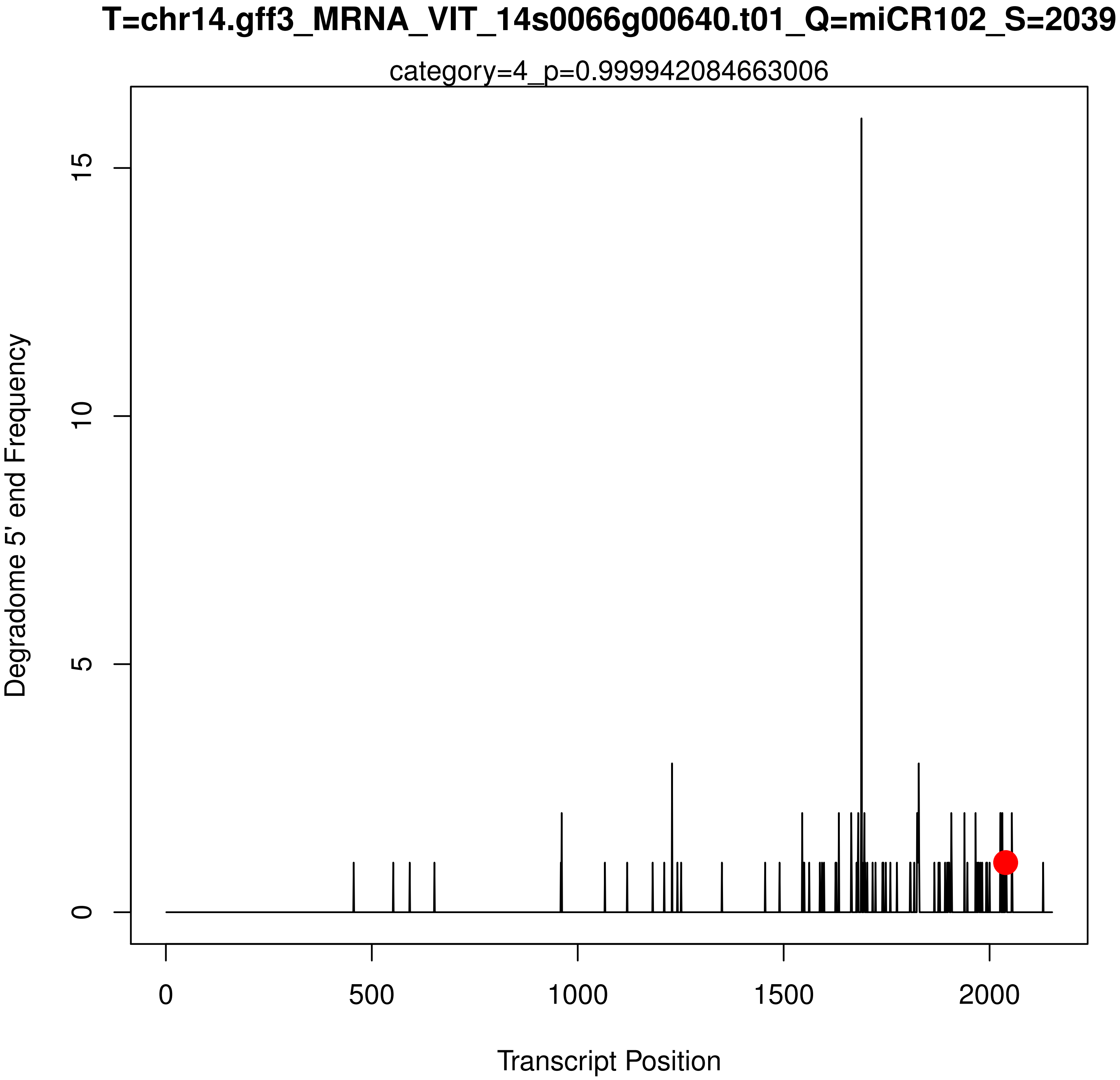

T=chr14.gff3_MRNA_VIT_14s0066g00640.t01_Q=miCR102_S=2039
category=4_p=0.999942084663006
Degradome 5' end Frequency
0
5
10
15
0
500
1000
1500
2000
Transcript Position

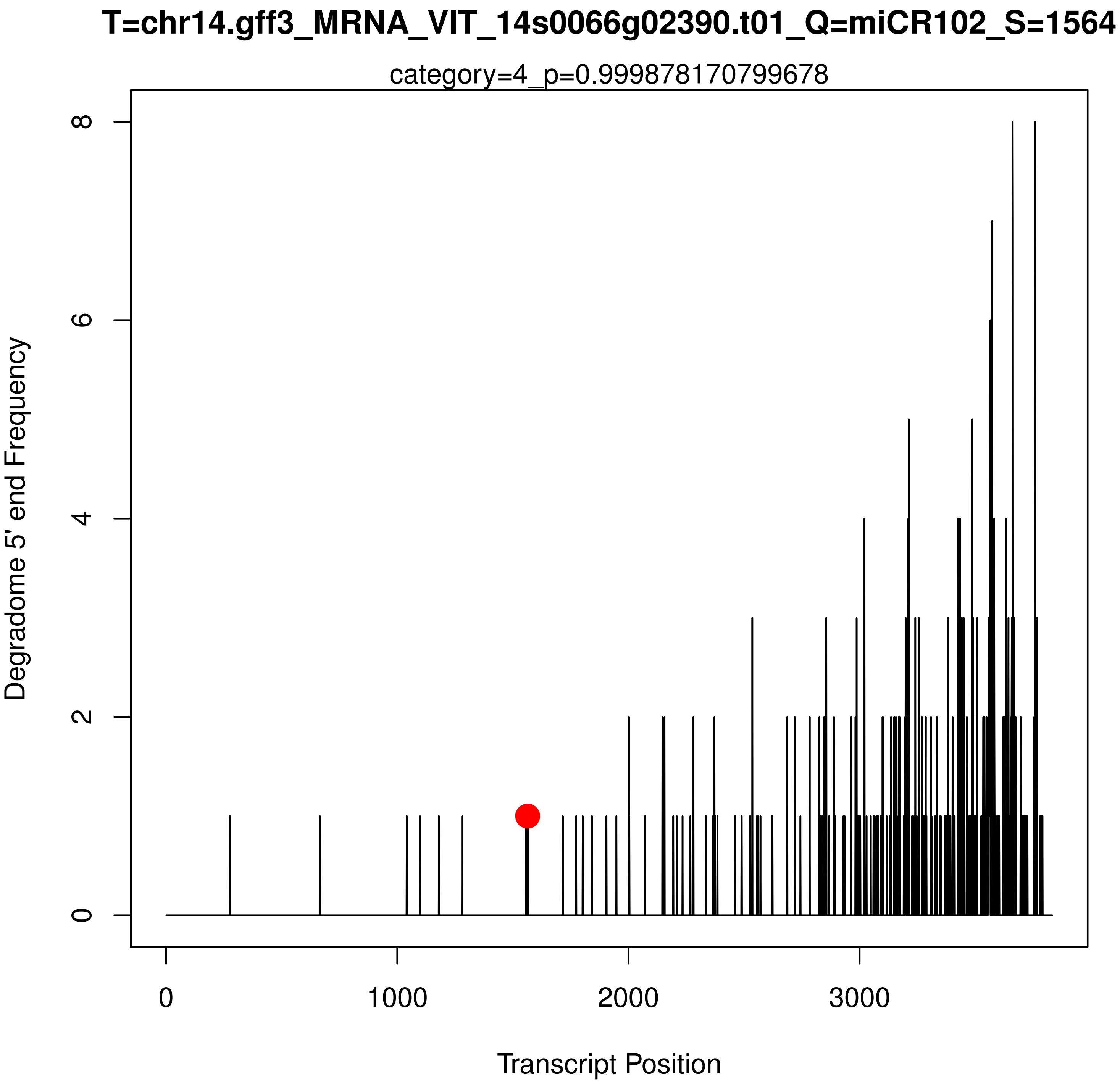
T=chr14.gff3_MRNA_VIT_14s0066g02390.t01_Q=miCR102_S=1564
category=4_p=0.999878170799678
Degradome 5' end Frequency
0
2
4
6
8
0
1000
2000
3000
Transcript Position

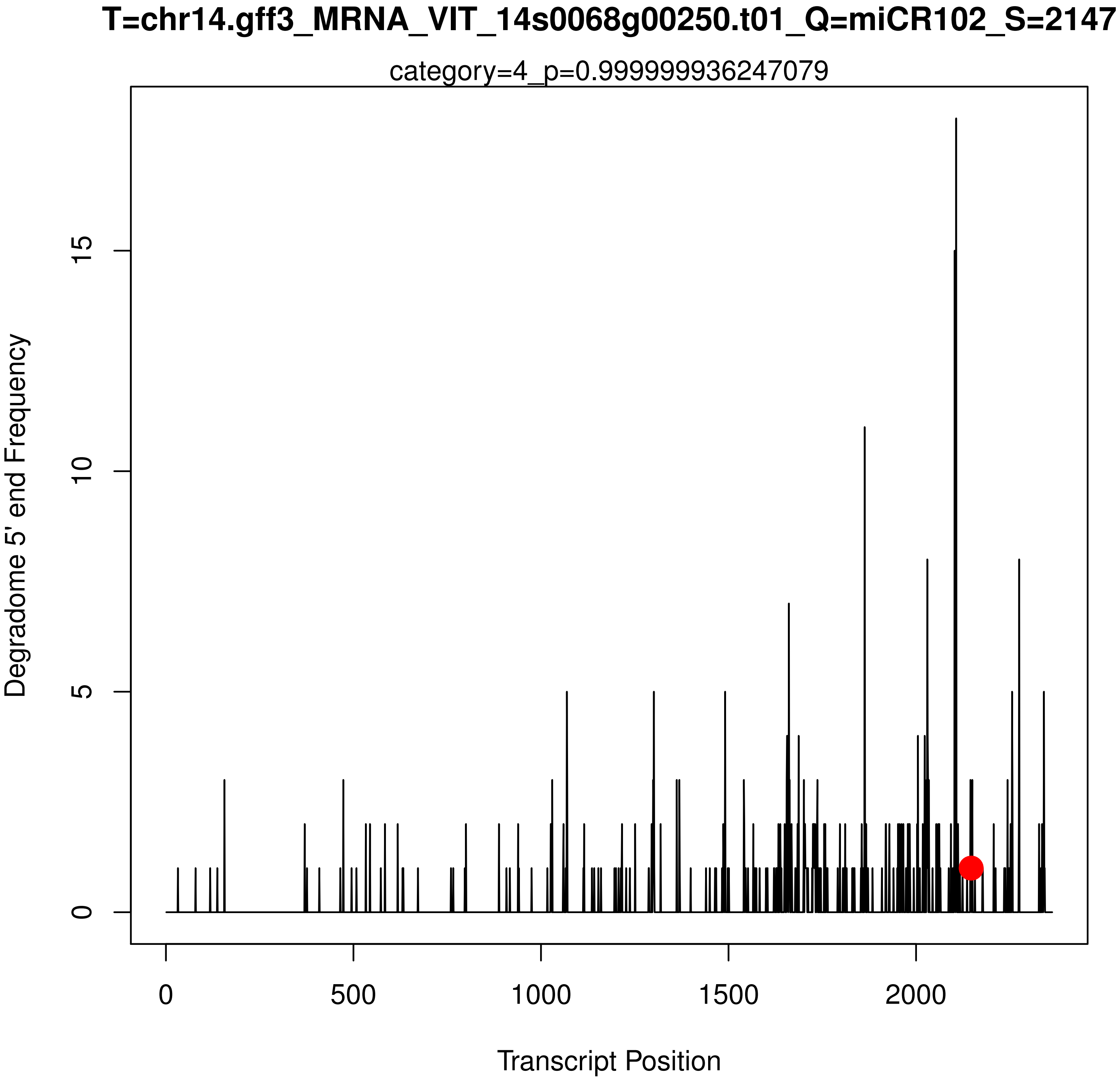
T=chr14.gff3_MRNA_VIT_14s0068g00250.t01_Q=miCR102_S=2147
category=4_p=0.999999936247079
Degradome 5' end Frequency
Transcript Position
0
5
10
15
0
500
1000
1500
2000

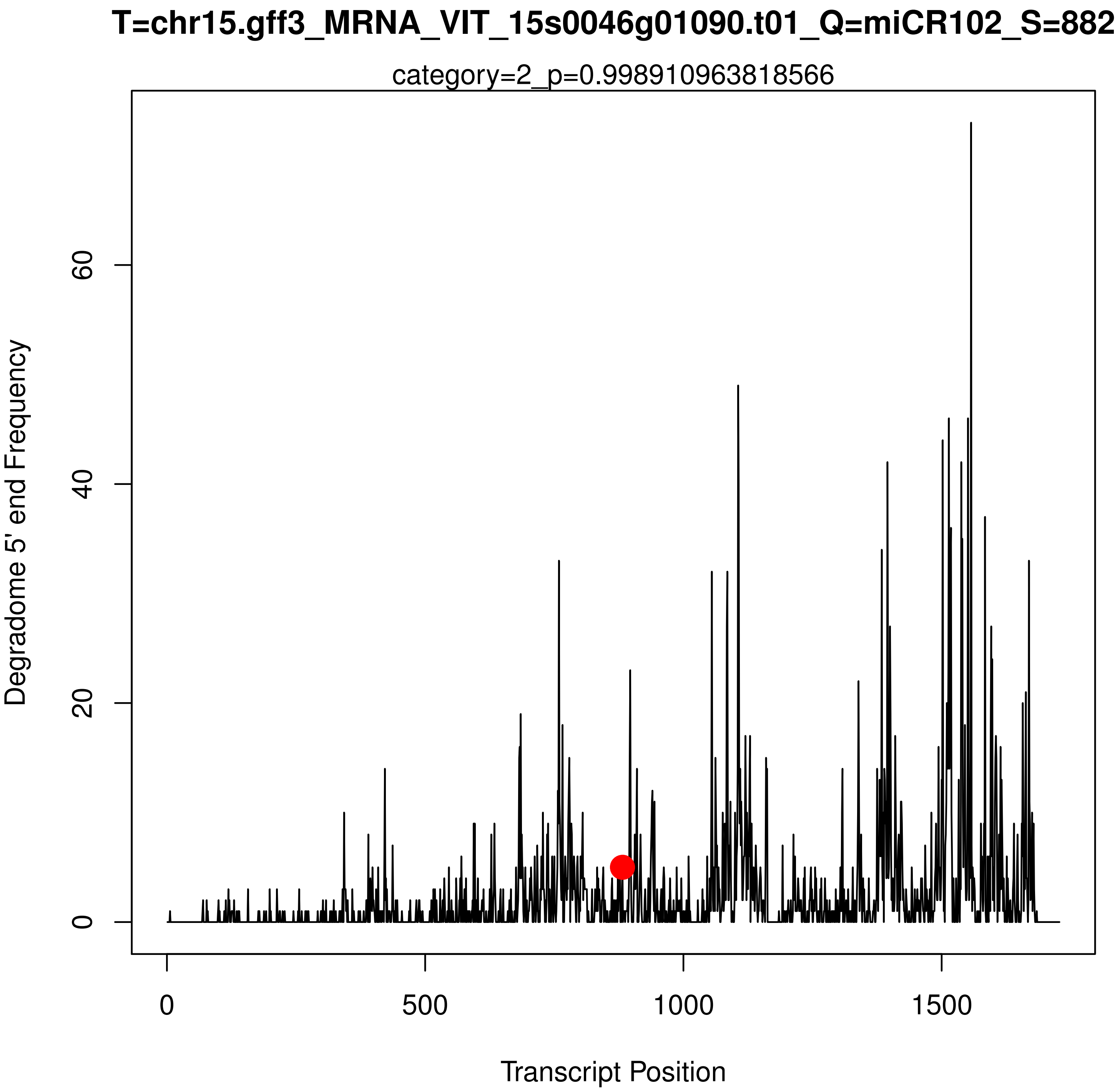
T=chr15.gff3_MRNA_VIT_15s0046g01090.t01_Q=miCR102_S=882
category=2_p=0.998910963818566
Degradome 5' end Frequency
Transcript Position
0
20
40
60
0
500
1000
1500

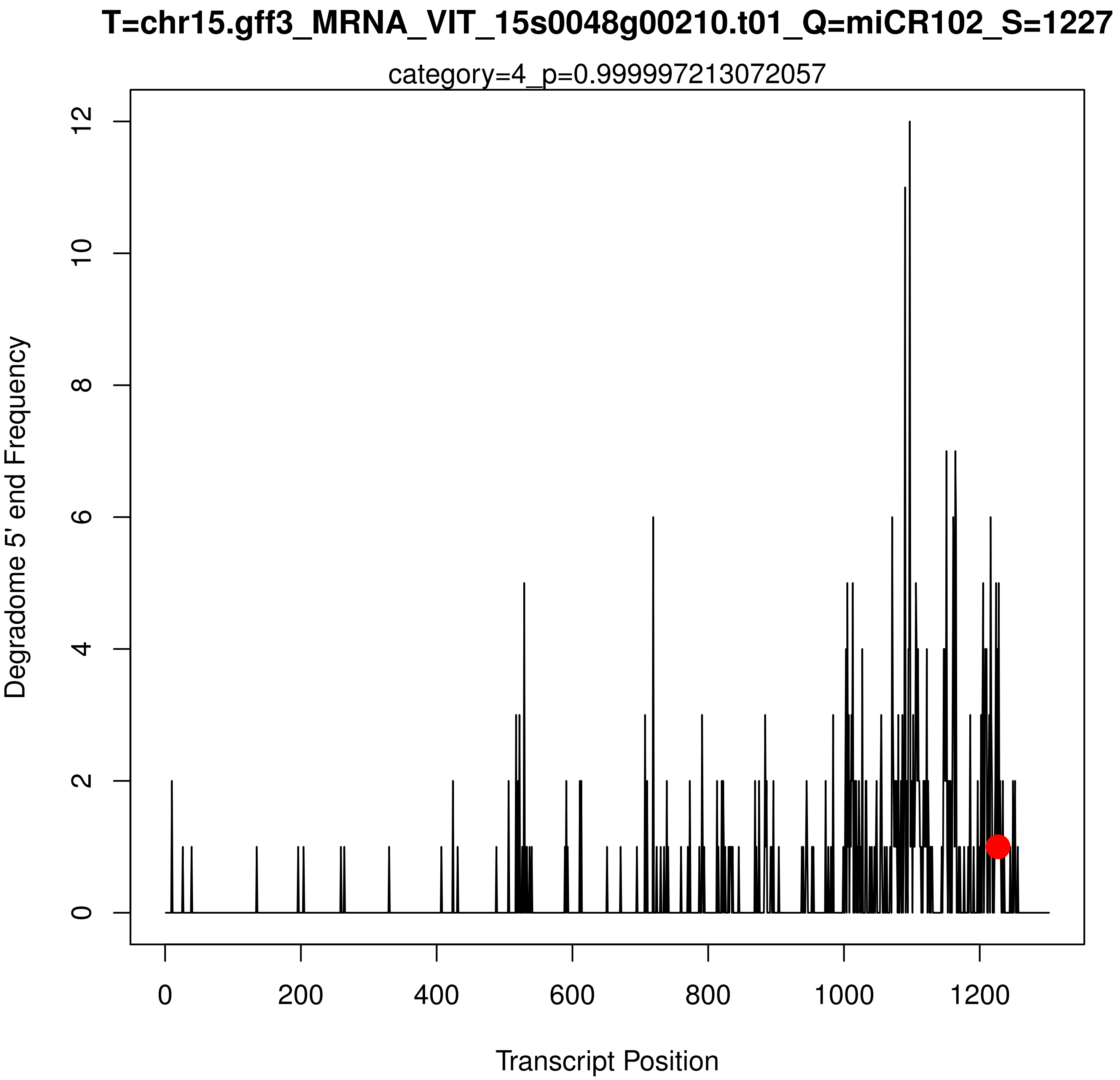
T=chr15.gff3_MRNA_VIT_15s0048g00210.t01_Q=miCR102_S=1227
category=4_p=0.999997213072057
Degradome 5' end Frequency
Transcript Position
0
2
4
6
8
10
12
0
200
400
600
800
1000
1200

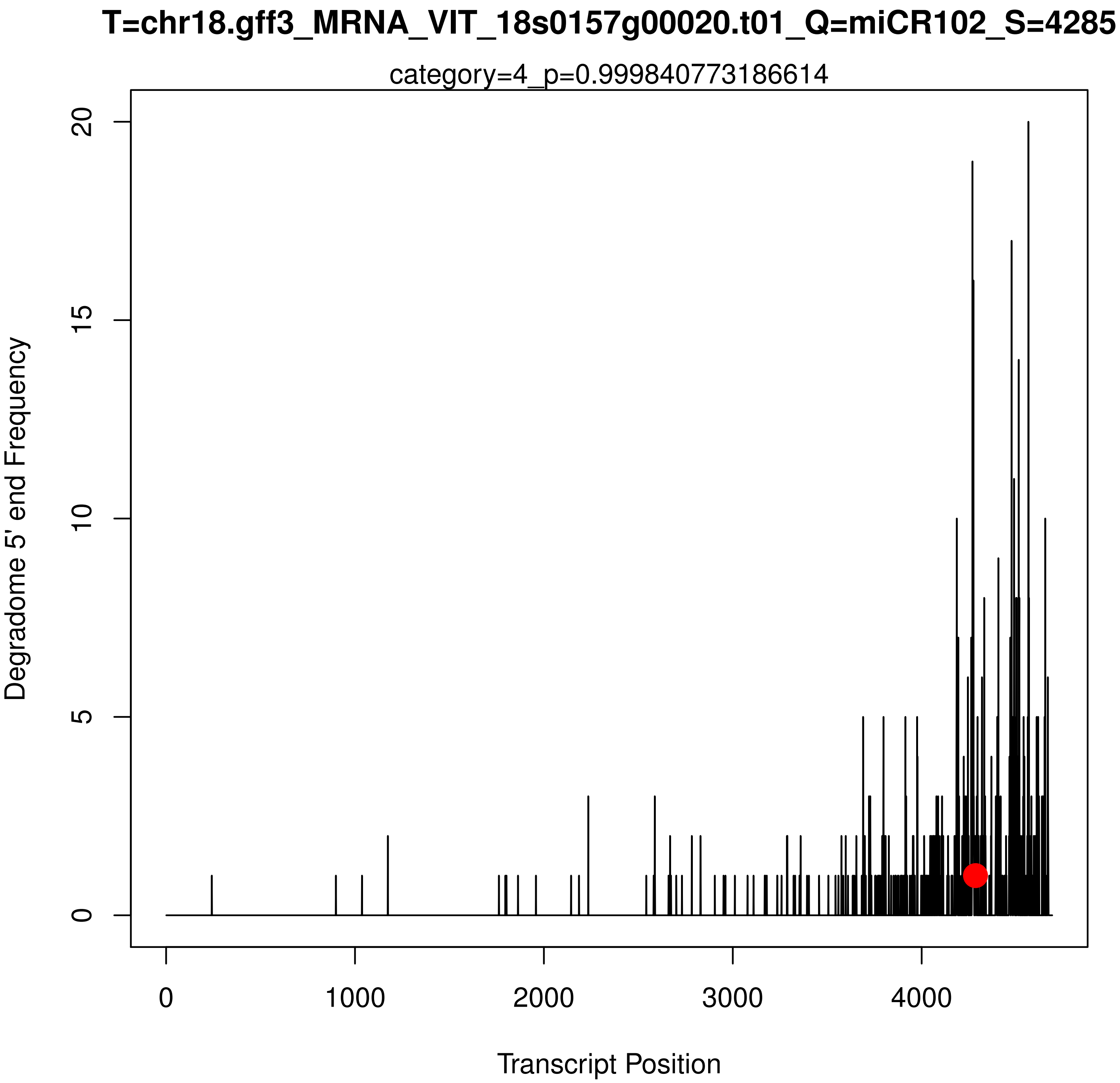
T=chr18.gff3_MRNA_VIT_18s0157g00020.t01_Q=miCR102_S=4285
category=4_p=0.999840773186614
Degradome 5' end Frequency
0
5
10
15
20
0
1000
2000
3000
4000
Transcript Position

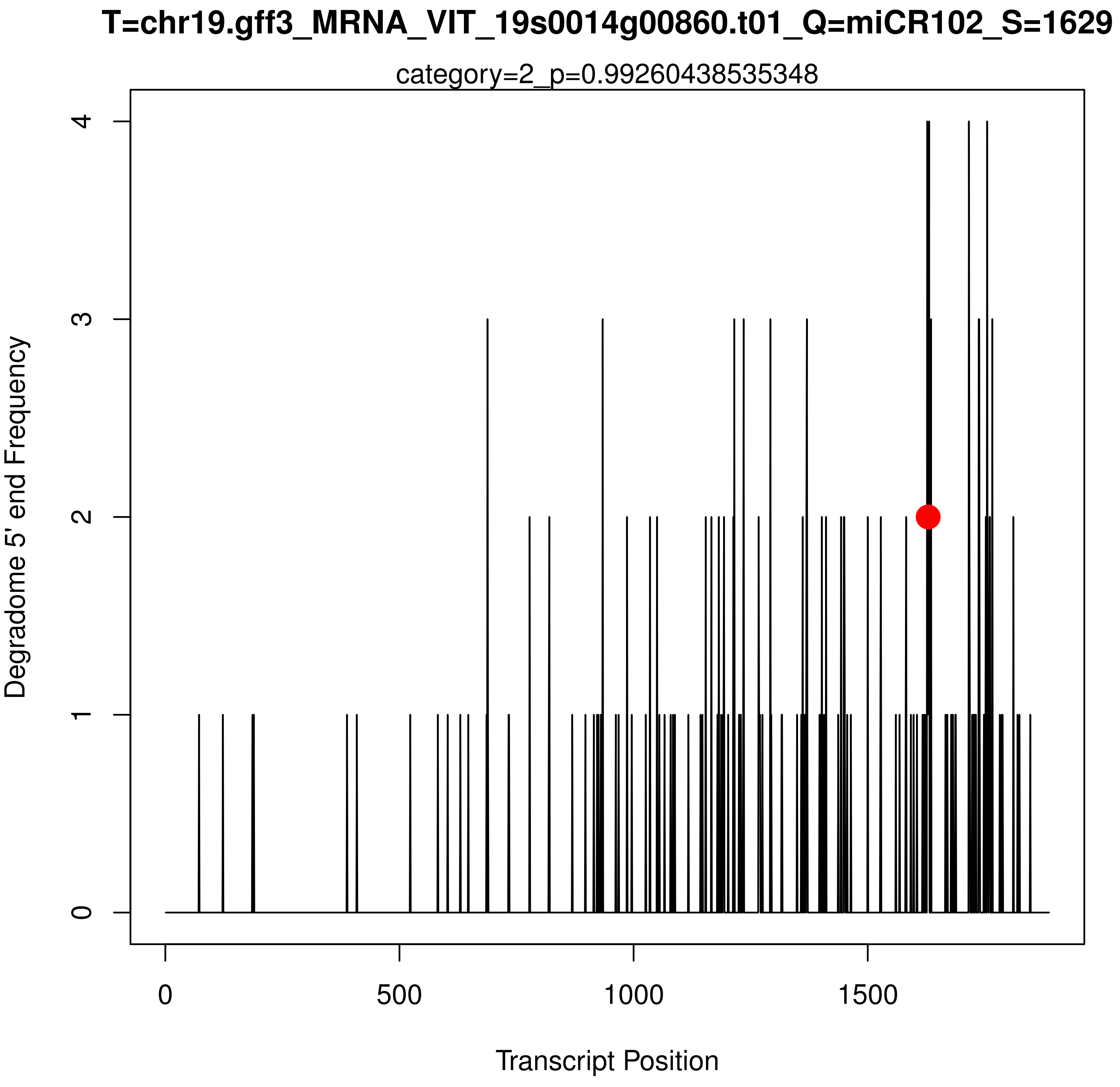

T=chr19.gff3_MRNA_VIT_19s0014g00860.t01_Q=miCR102_S=1629
category=2_p=0.99260438535348
Degradome 5' end Frequency
0
1
2
3
4
0
500
1000
1500
Transcript Position

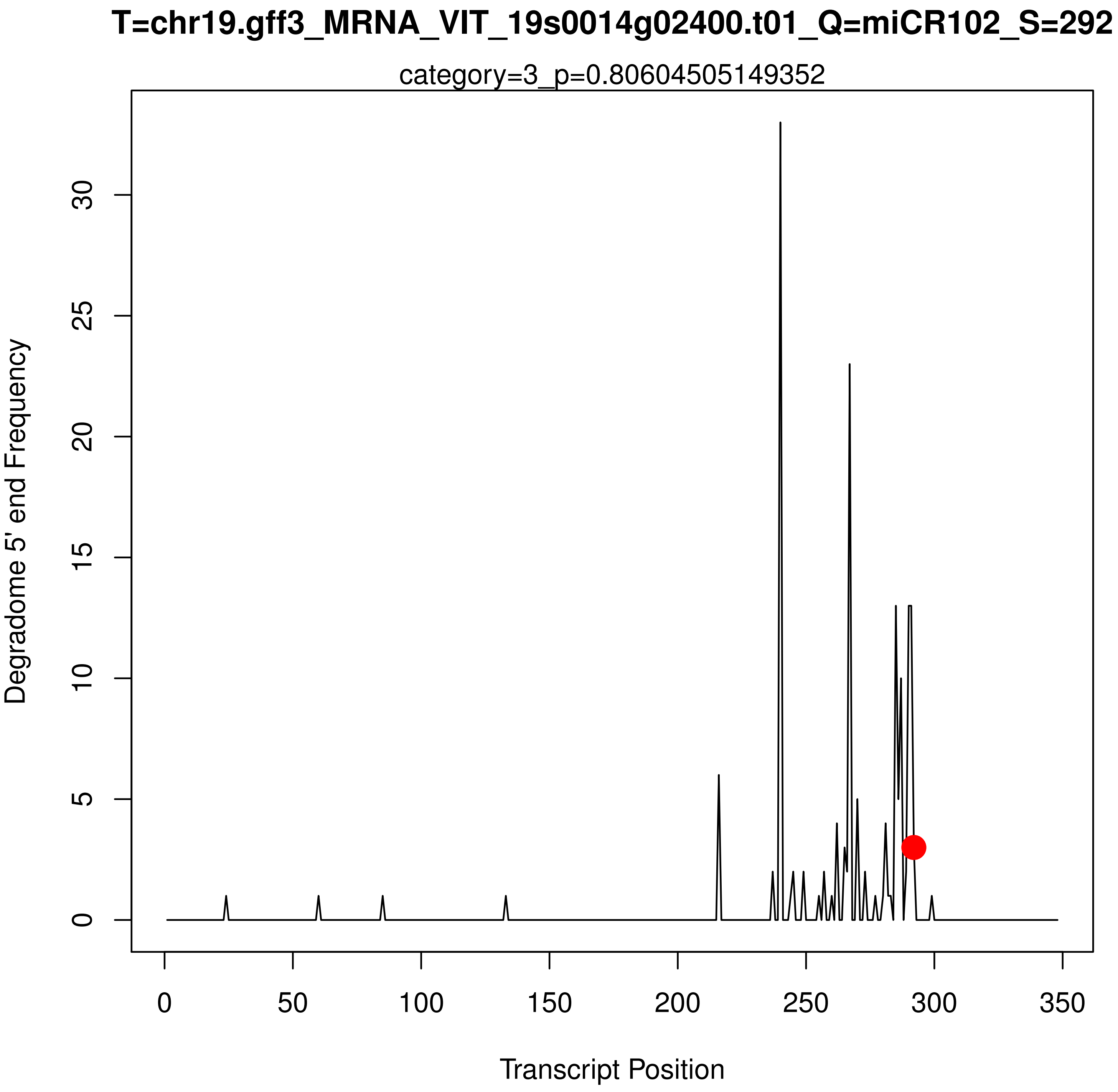
T=chr19.gff3_MRNA_VIT_19s0014g02400.t01_Q=miCR102_S=292
category=3_p=0.80604505149352
Degradome 5' end Frequency
Transcript Position
0
5
10
15
20
25
30
0
50
100
150
200
250
300
350

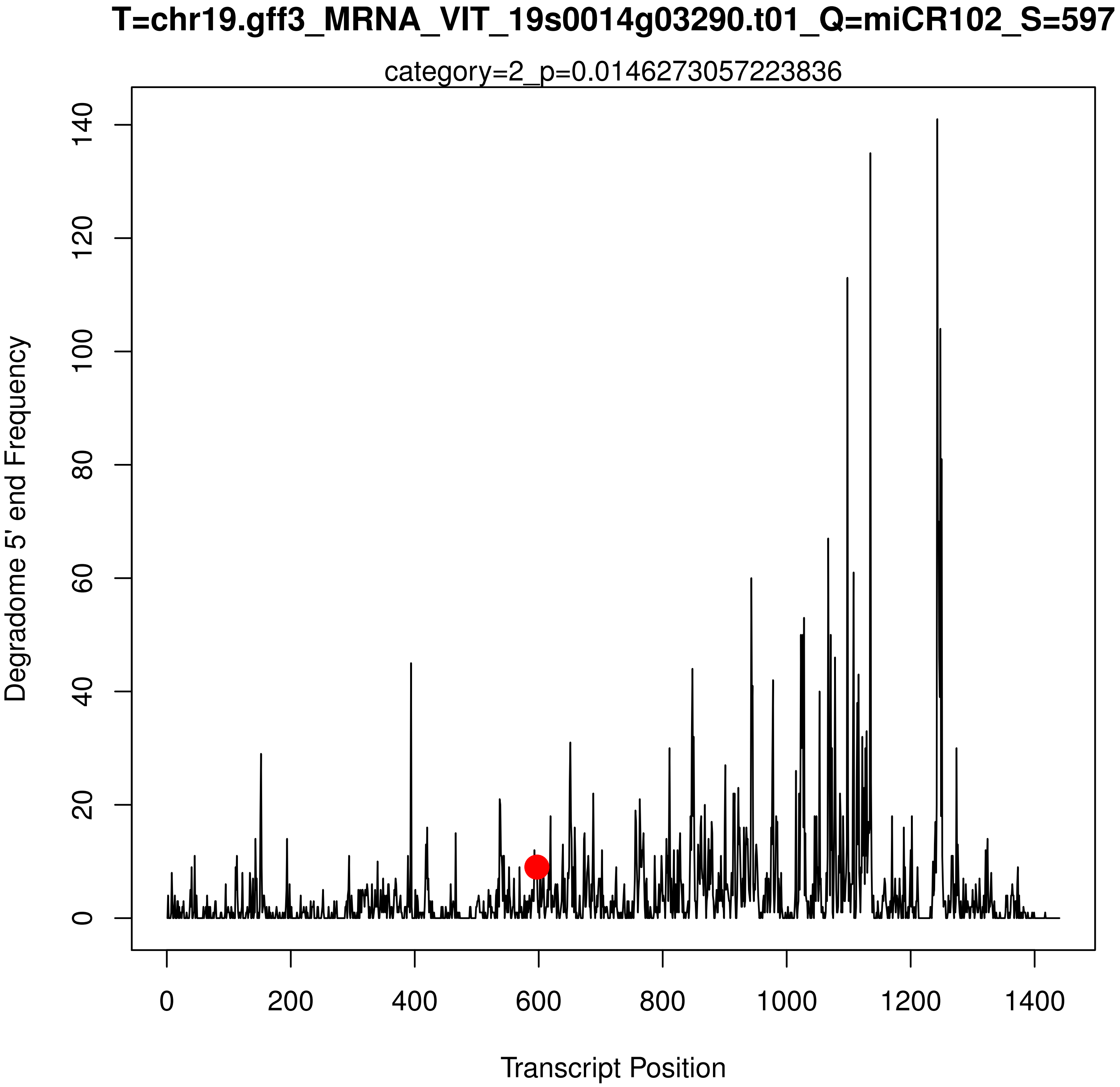

T=chr19.gff3_MRNA_VIT_19s0014g03290.t01_Q=miCR102_S=597
category=2_p=0.0146273057223836
Degradome 5' end Frequency
0
20
40
60
80
100
120
140
0
200
400
600
800
1000
1200
1400
Transcript Position

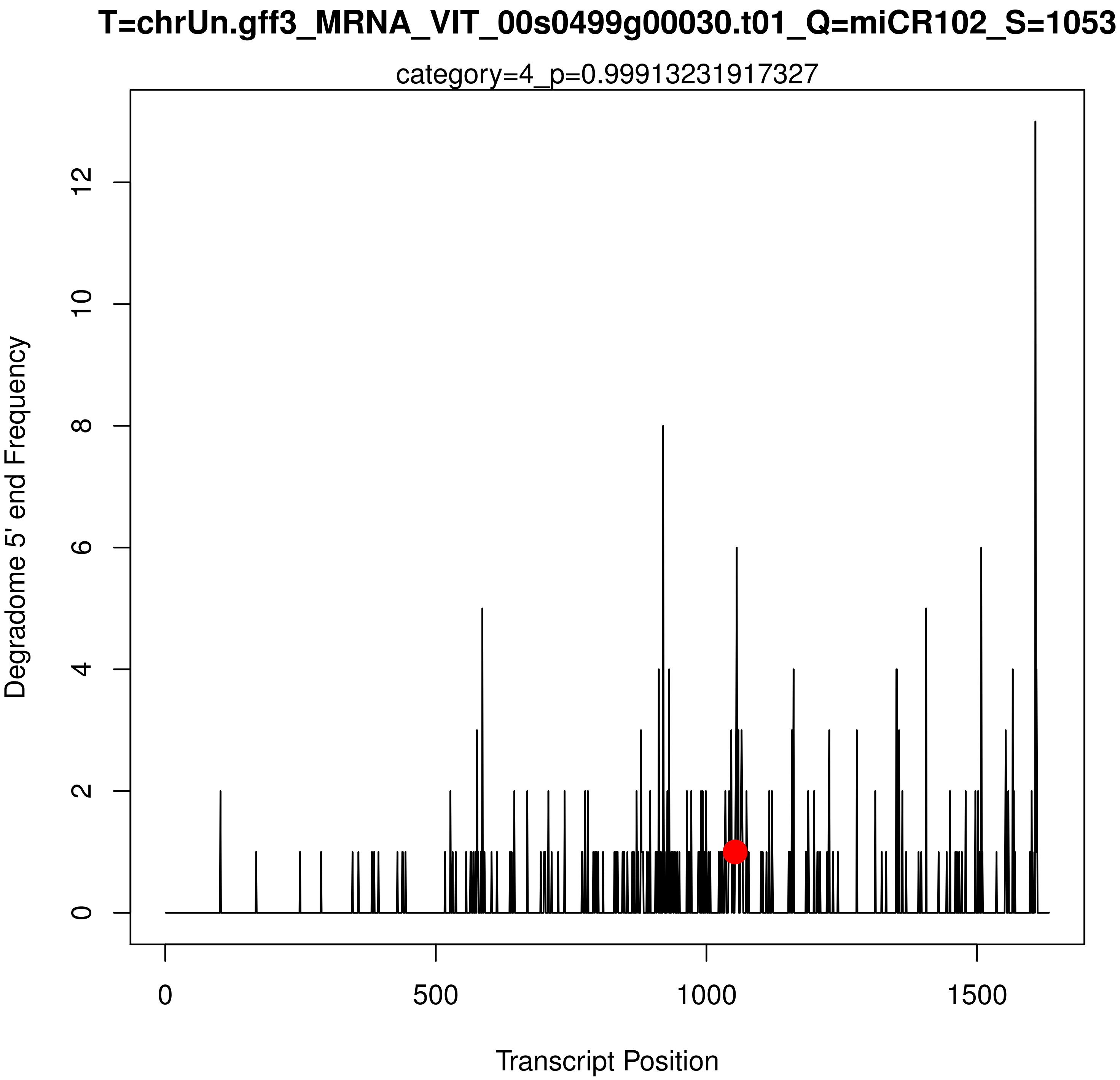

T=chrUn.gff3_MRNA_VIT_00s0499g00030.t01_Q=miCR102_S=1053
category=4_p=0.99913231917327
Degradome 5' end Frequency
Transcript Position
0
2
4
6
8
10
12
0
500
1000
1500

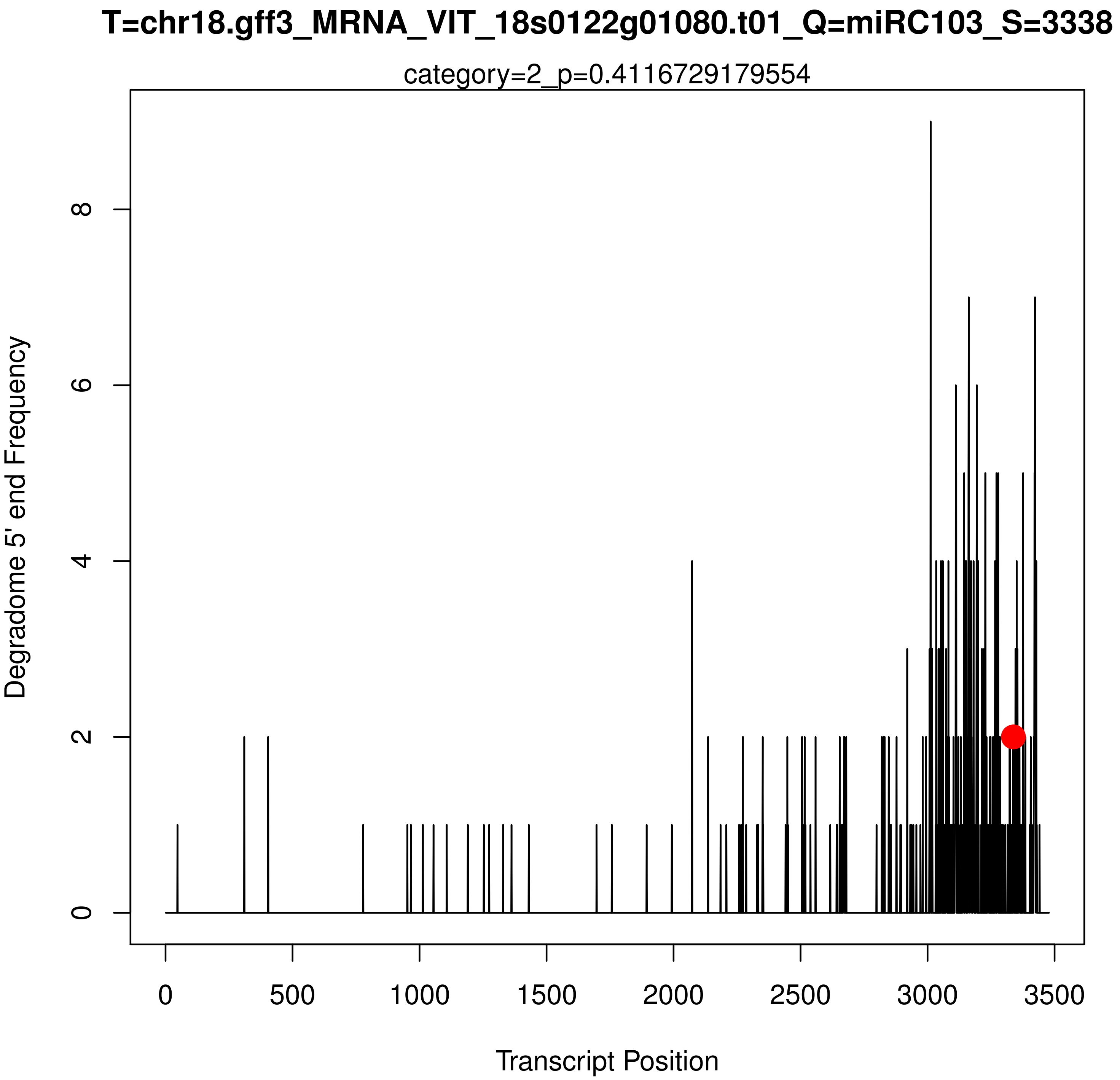

T=chr18.gff3_MRNA_VIT_18s0122g01080.t01_Q=miRC103_S=3338
category=2_p=0.4116729179554
Degradome 5' end Frequency
0
2
4
6
8
0
500
1000
1500
2000
2500
3000
3500
Transcript Position

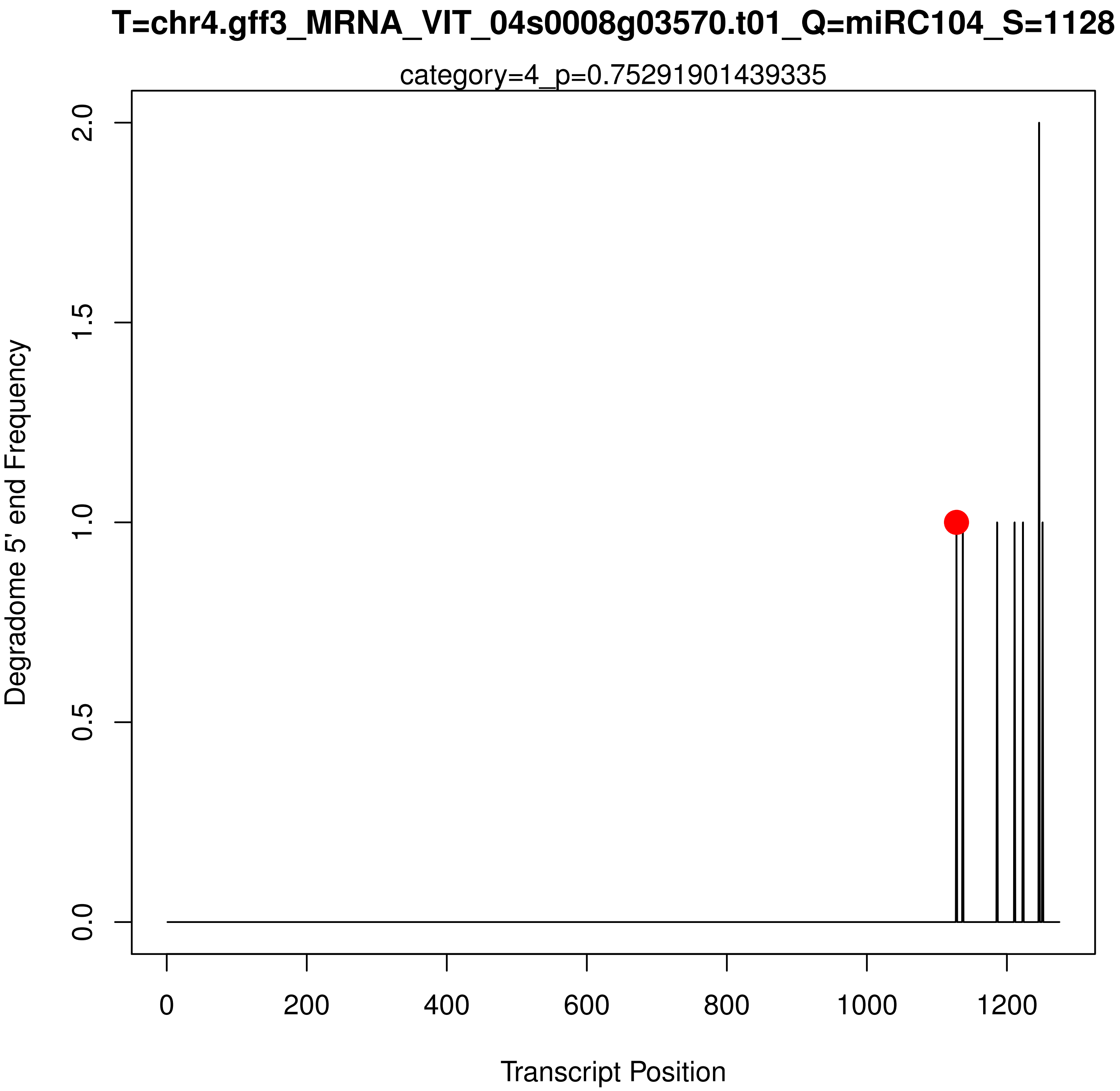
T=chr4.gff3_MRNA_VIT_04s0008g03570.t01_Q=miRC104_S=1128
category=4_p=0.75291901439335
Degradome 5' end Frequency
0.0
0.5
1.0
1.5
2.0
0
200
400
600
800
1000
1200
Transcript Position

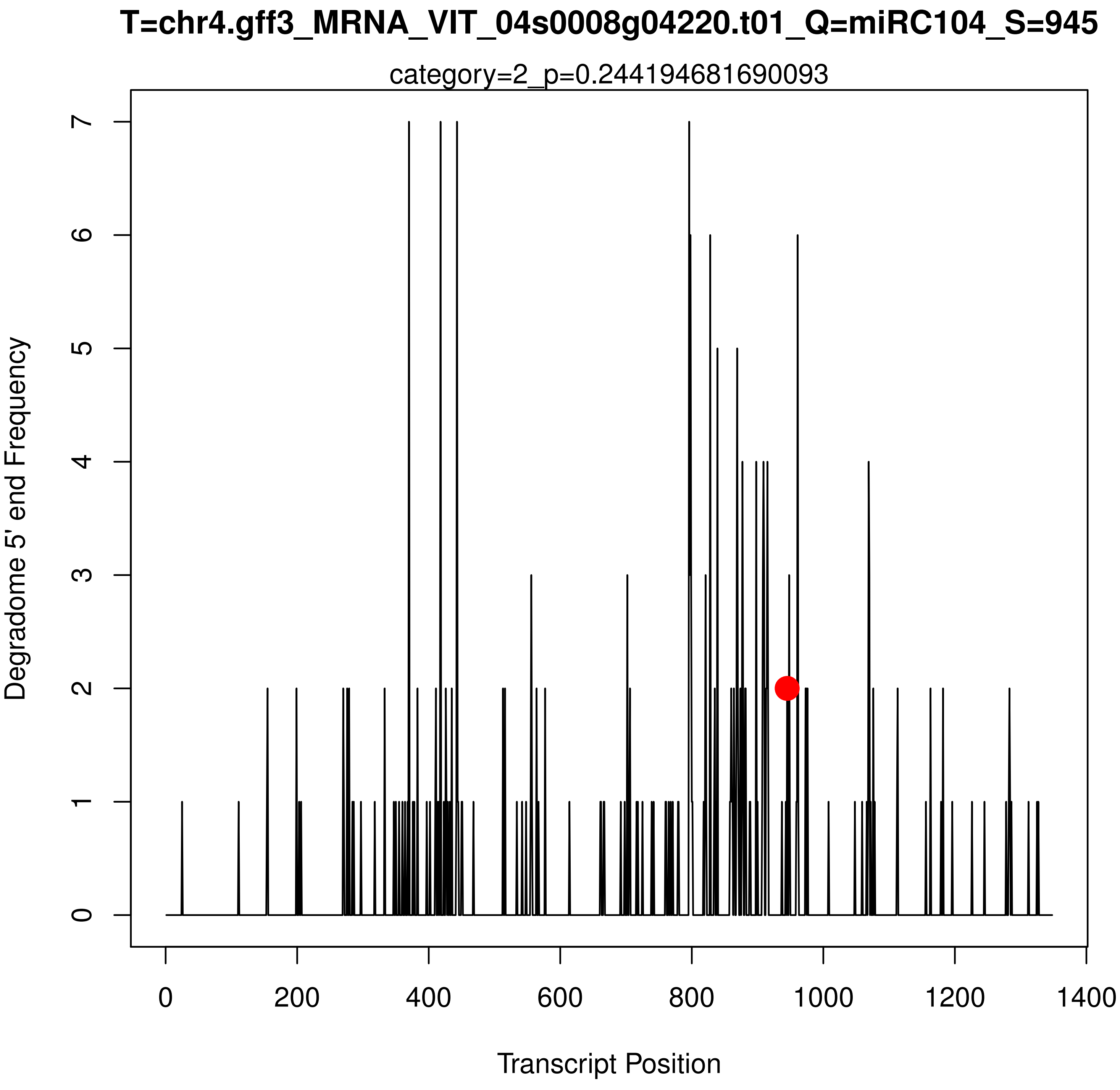
T=chr4.gff3_MRNA_VIT_04s0008g04220.t01_Q=miRC104_S=945
category=2_p=0.244194681690093
Degradome 5' end Frequency
0
1
2
3
4
5
6
7
0
200
400
600
800
1000
1200
1400
Transcript Position

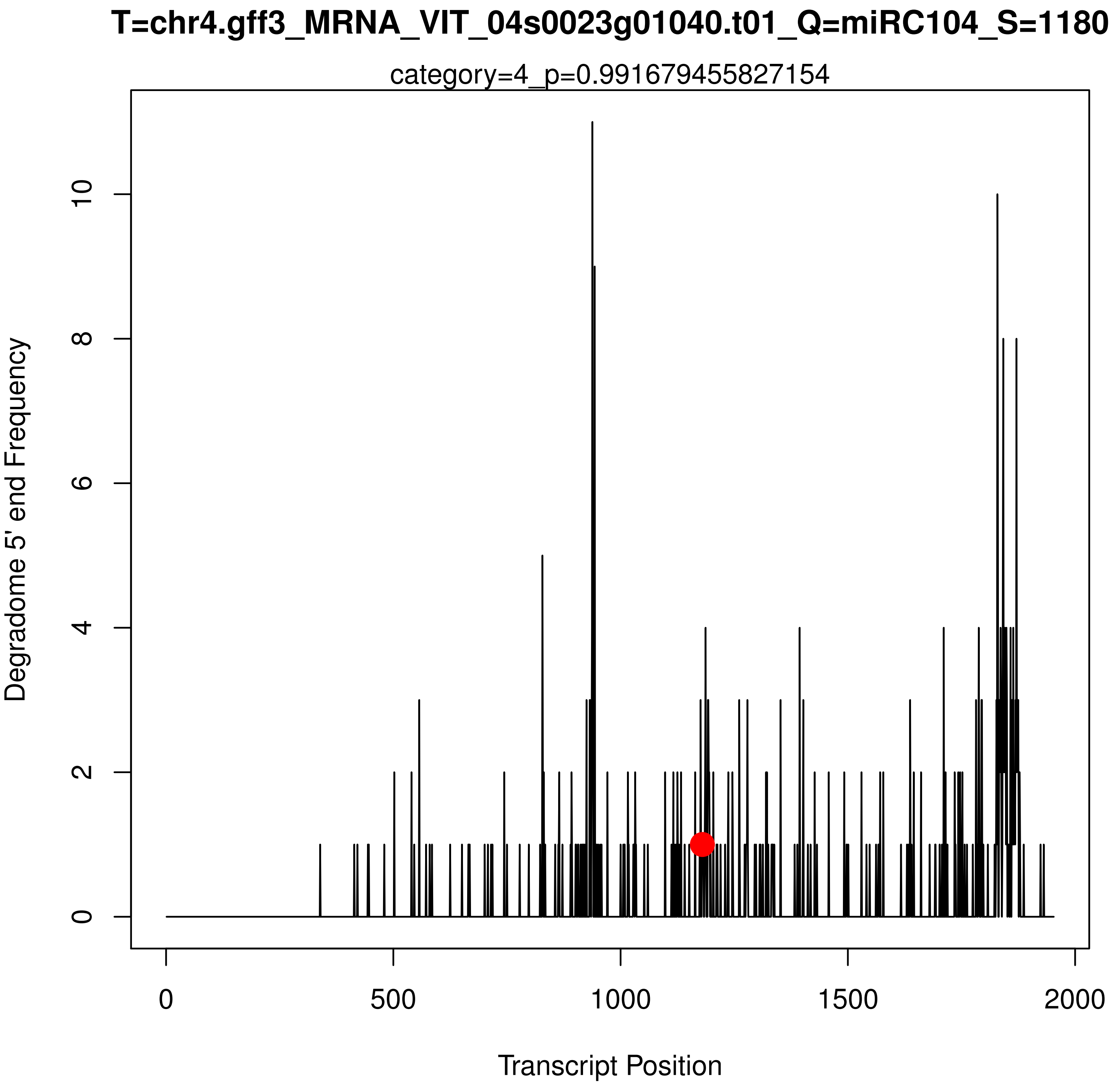
T=chr4.gff3_MRNA_VIT_04s0023g01040.t01_Q=miRC104_S=1180
category=4_p=0.991679455827154
Degradome 5' end Frequency
0
2
4
6
8
10
0
500
1000
1500
2000
Transcript Position

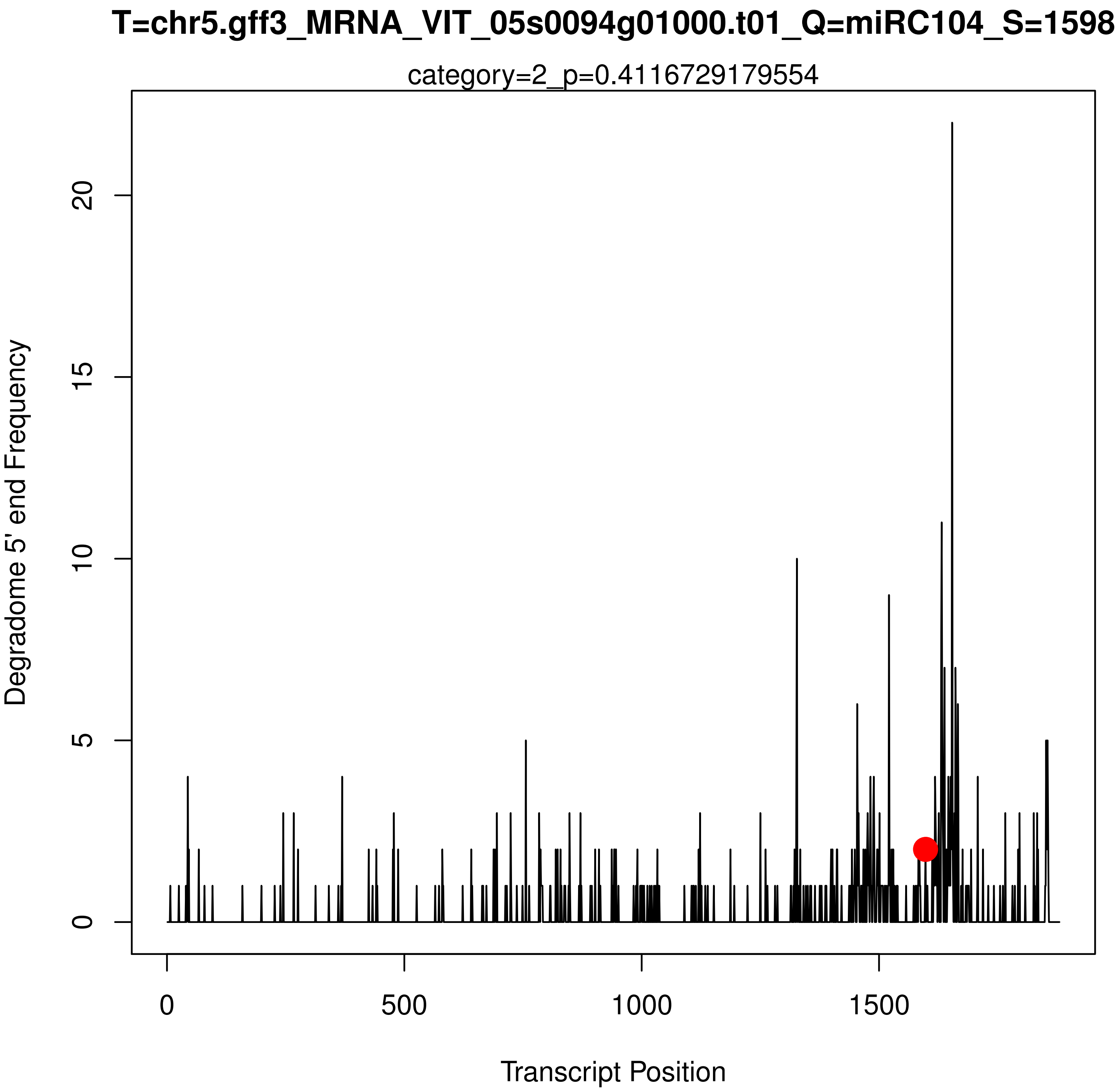

T=chr5.gff3_MRNA_VIT_05s0094g01000.t01_Q=miRC104_S=1598
category=2_p=0.4116729179554
Degradome 5' end Frequency
Transcript Position
0
5
10
15
20
0
500
1000
1500

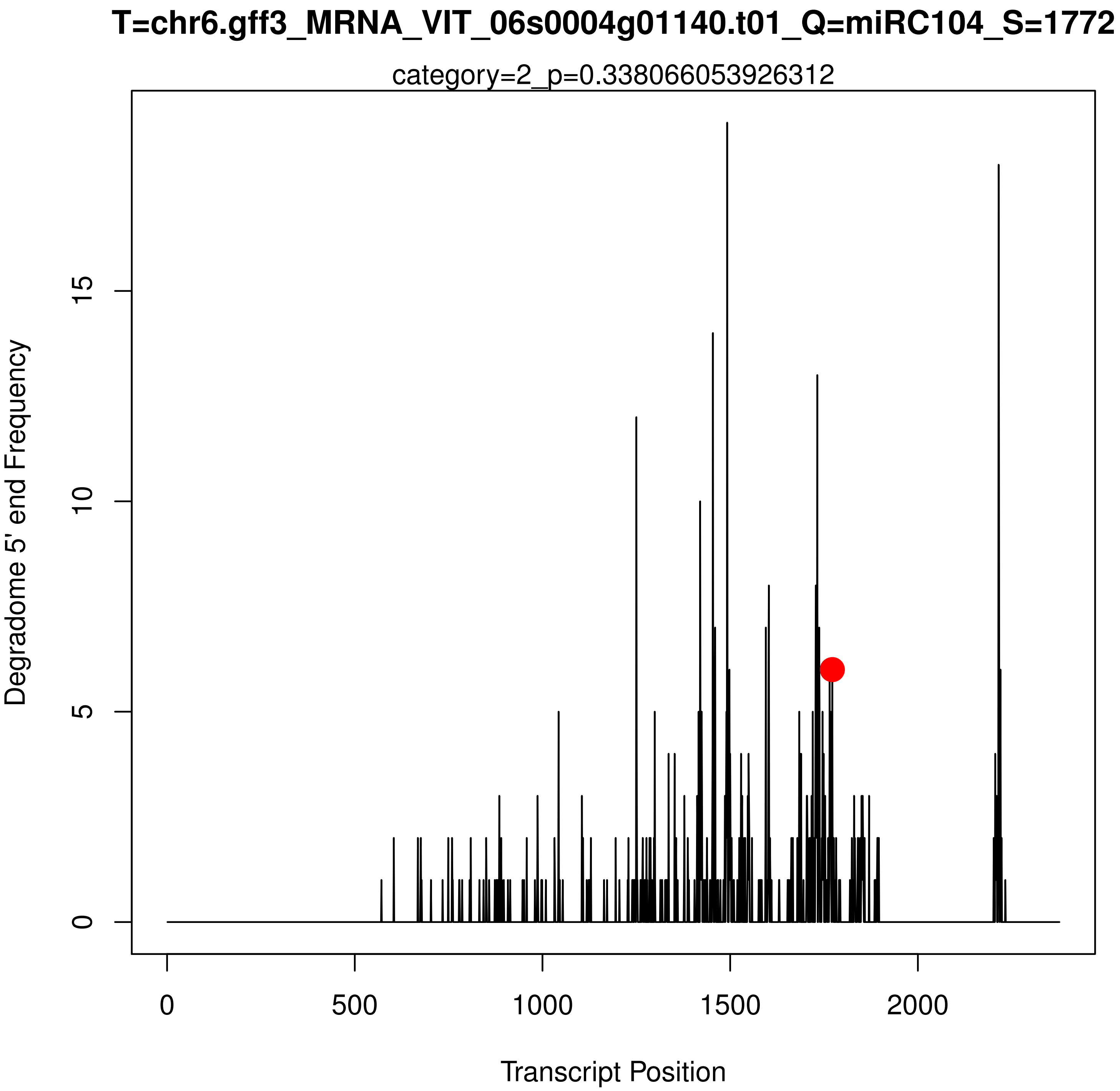

T=chr6.gff3_MRNA_VIT_06s0004g01140.t01_Q=miRC104_S=1772
category=2_p=0.338066053926312
Degradome 5' end Frequency
0
5
10
15
0
500
1000
1500
2000
Transcript Position

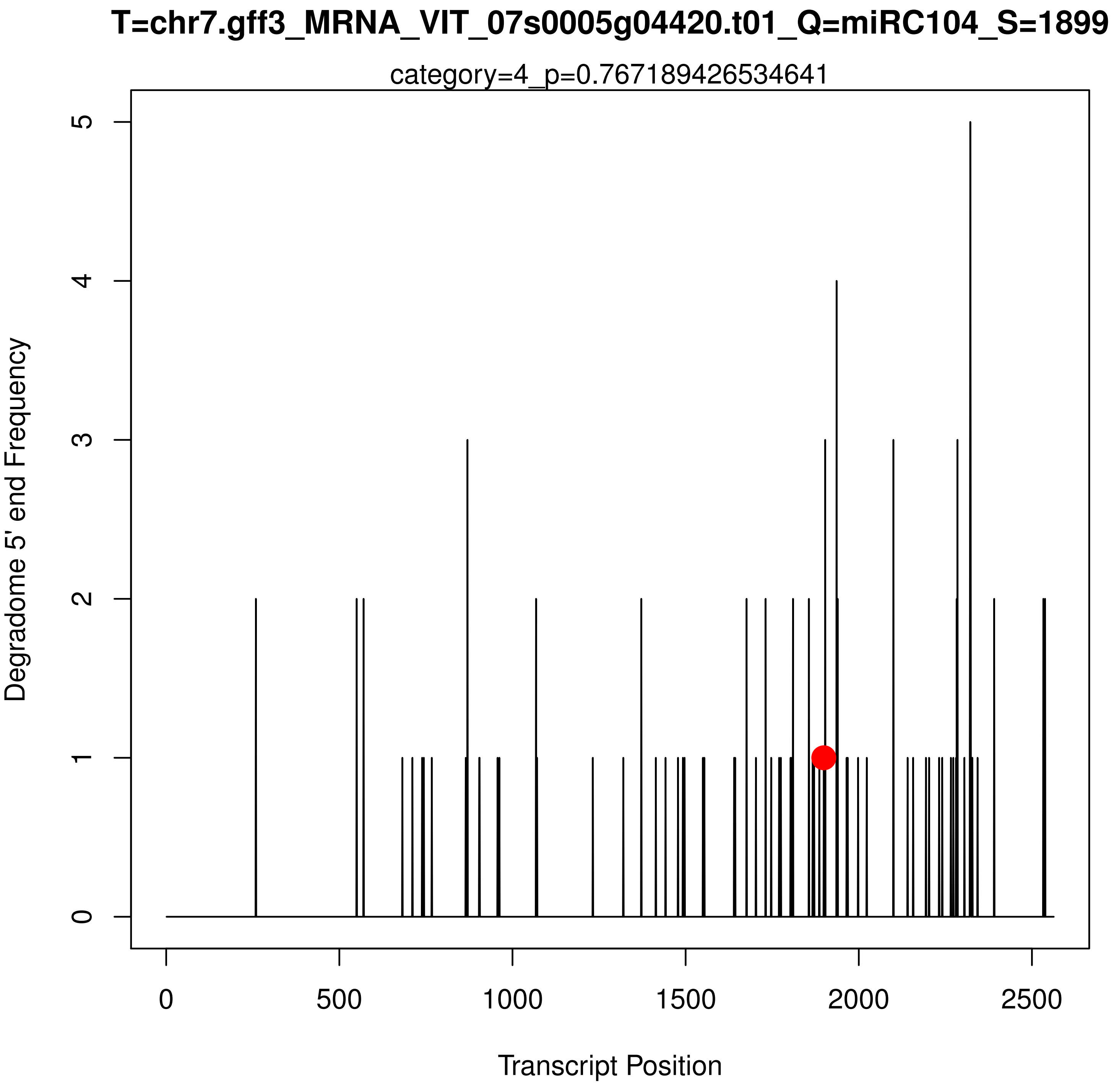
T=chr7.gff3_MRNA_VIT_07s0005g04420.t01_Q=miRC104_S=1899
category=4_p=0.767189426534641
Degradome 5' end Frequency
Transcript Position
0
1
2
3
4
5
0
500
1000
1500
2000
2500

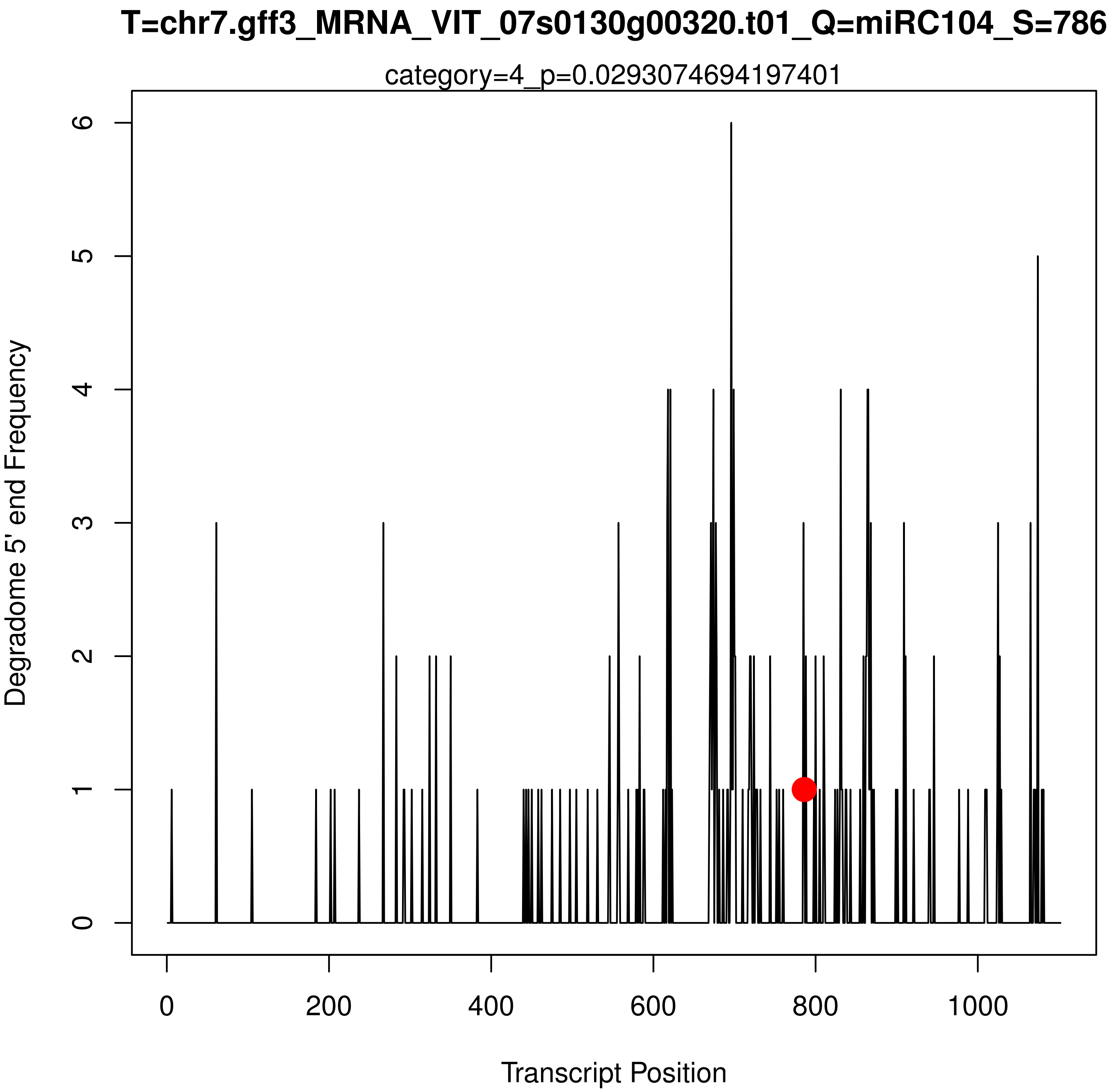
T=chr7.gff3_MRNA_VIT_07s0130g00320.t01_Q=miRC104_S=786
category=4_p=0.0293074694197401
Degradome 5' end Frequency
0
1
2
3
4
5
6
0
200
400
600
800
1000
Transcript Position

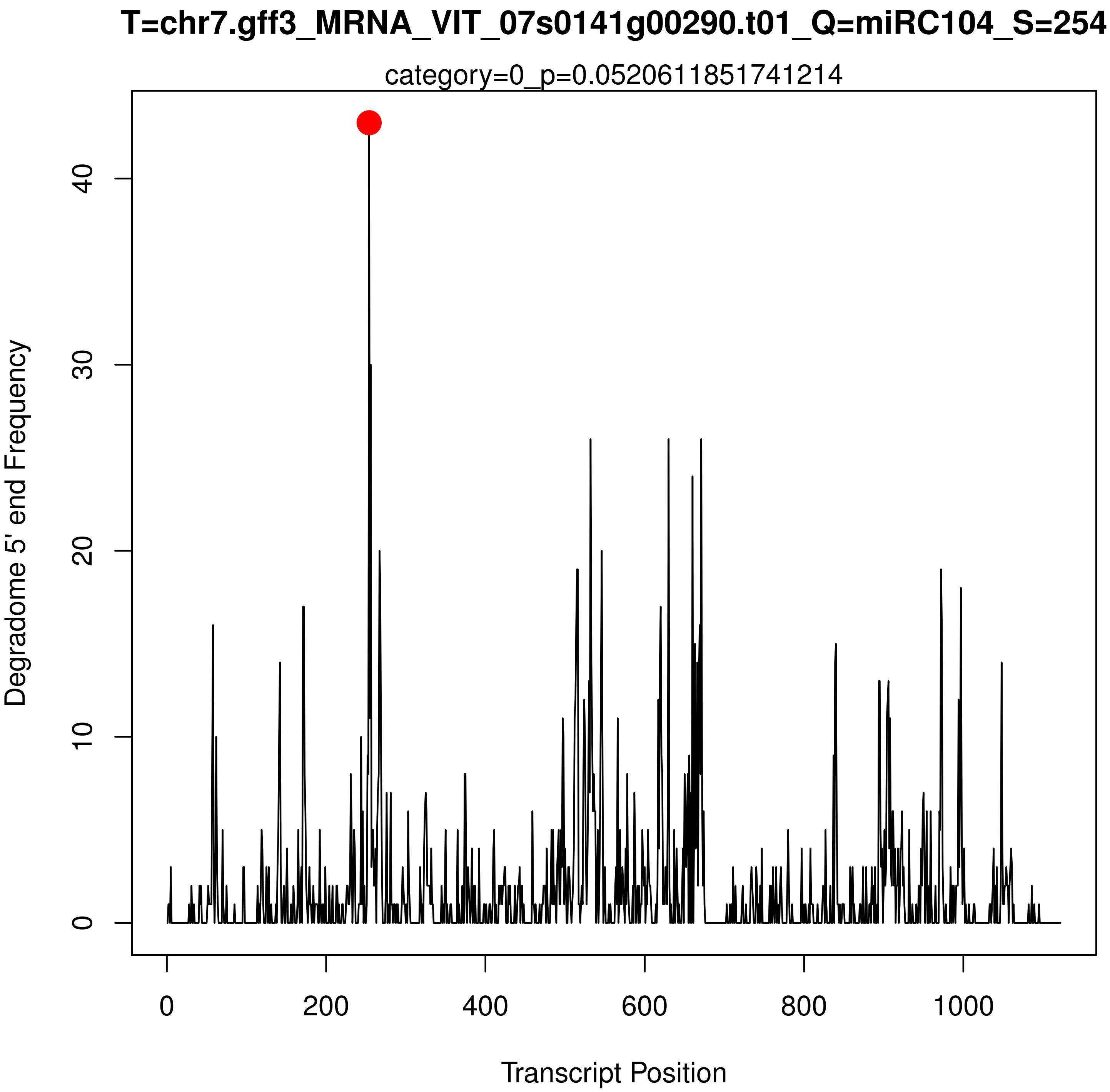

T=chr7.gff3_MRNA_VIT_07s0141g00290.t01_Q=miRC104_S=254
category=0_p=0.0520611851741214
Degradome 5' end Frequency
0
10
20
30
40
0
200
400
600
800
1000
Transcript Position

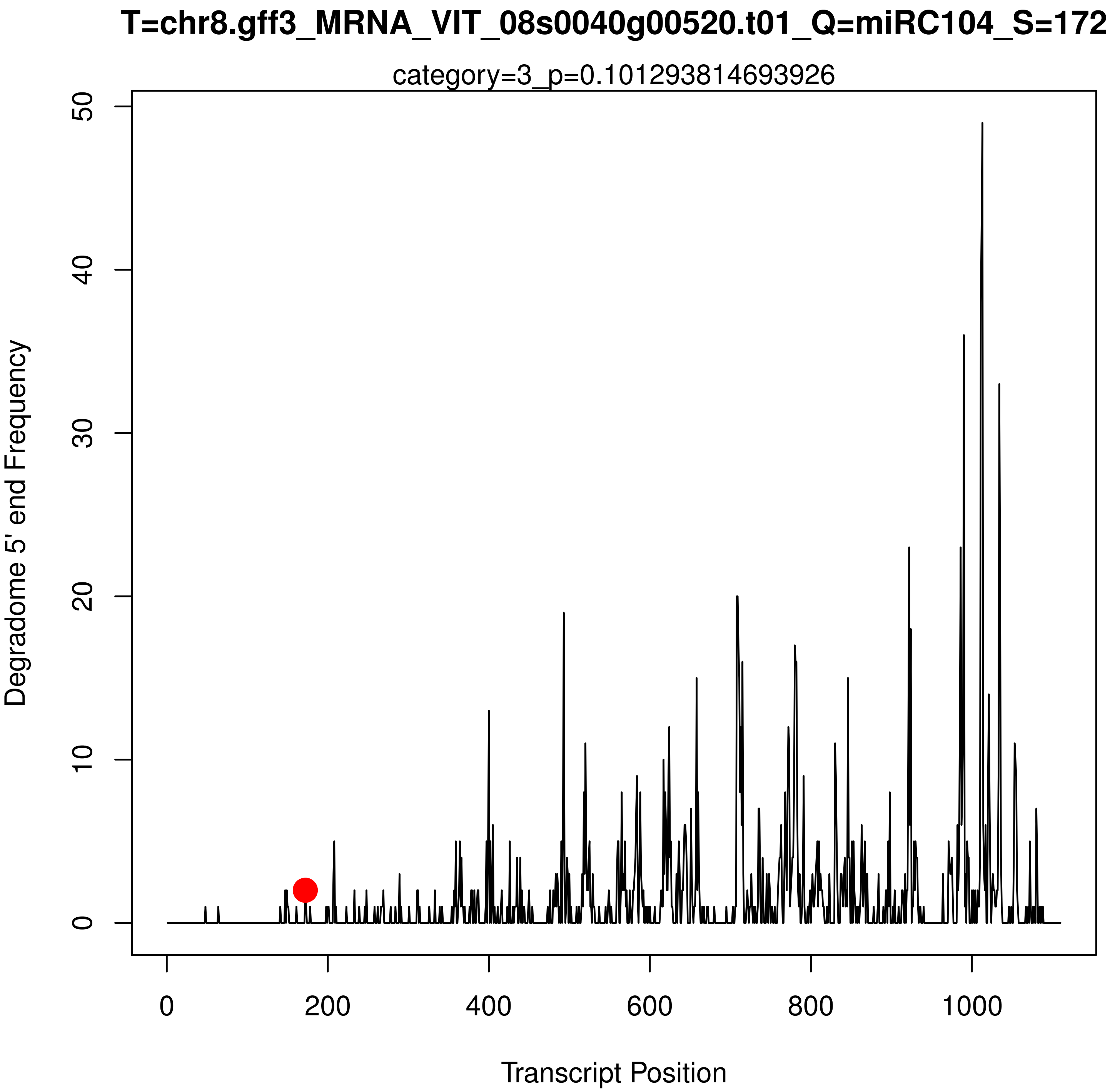
T=chr8.gff3_MRNA_VIT_08s0040g00520.t01_Q=miRC104_S=172
category=3_p=0.101293814693926
Degradome 5' end Frequency
Transcript Position
0
10
20
30
40
50
0
200
400
600
800
1000

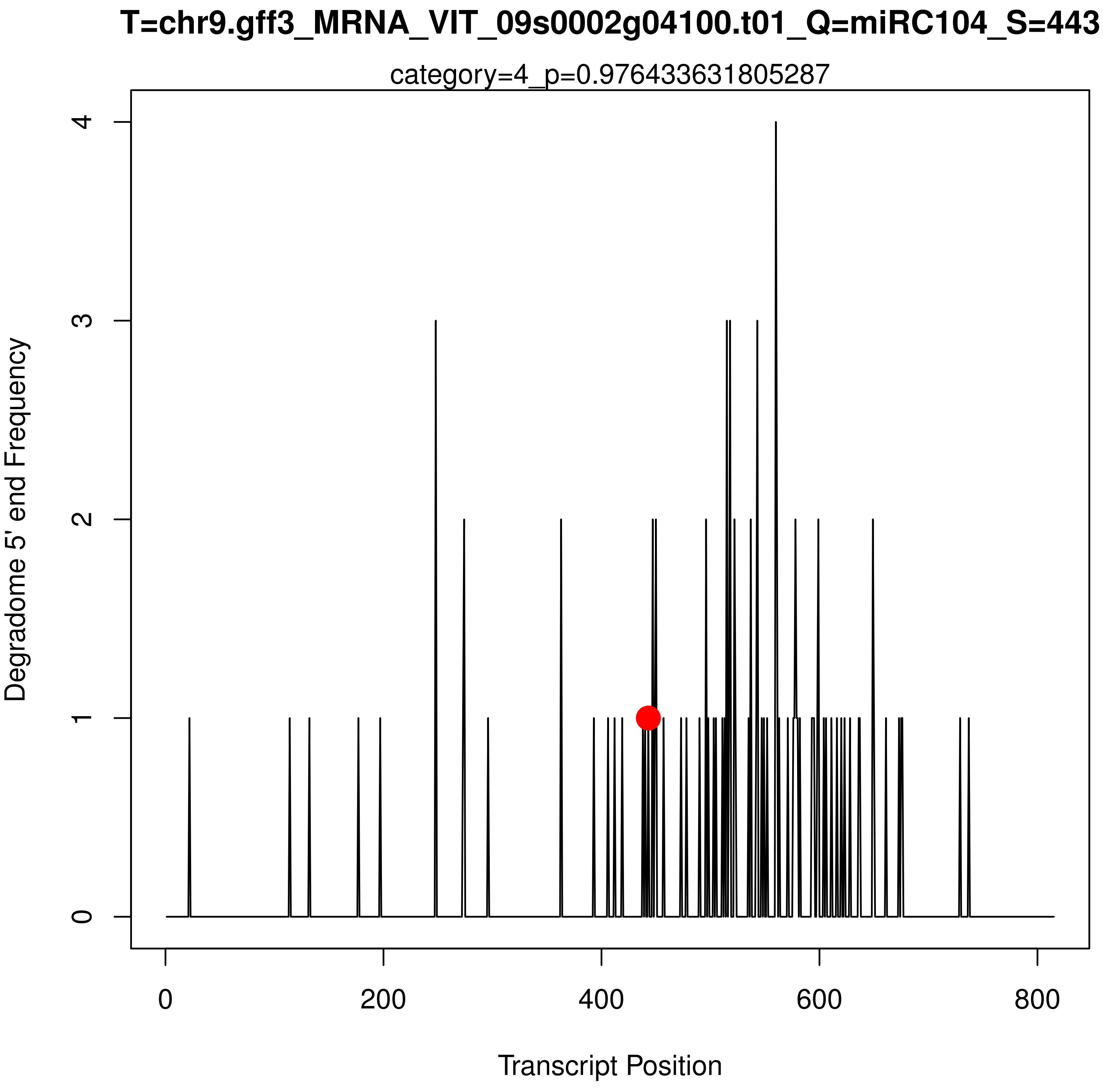
T=chr9.gff3_MRNA_VIT_09s0002g04100.t01_Q=miRC104_S=443
category=4_p=0.976433631805287
Degradome 5' end Frequency
Transcript Position
0
1
2
3
4
0
200
400
600
800

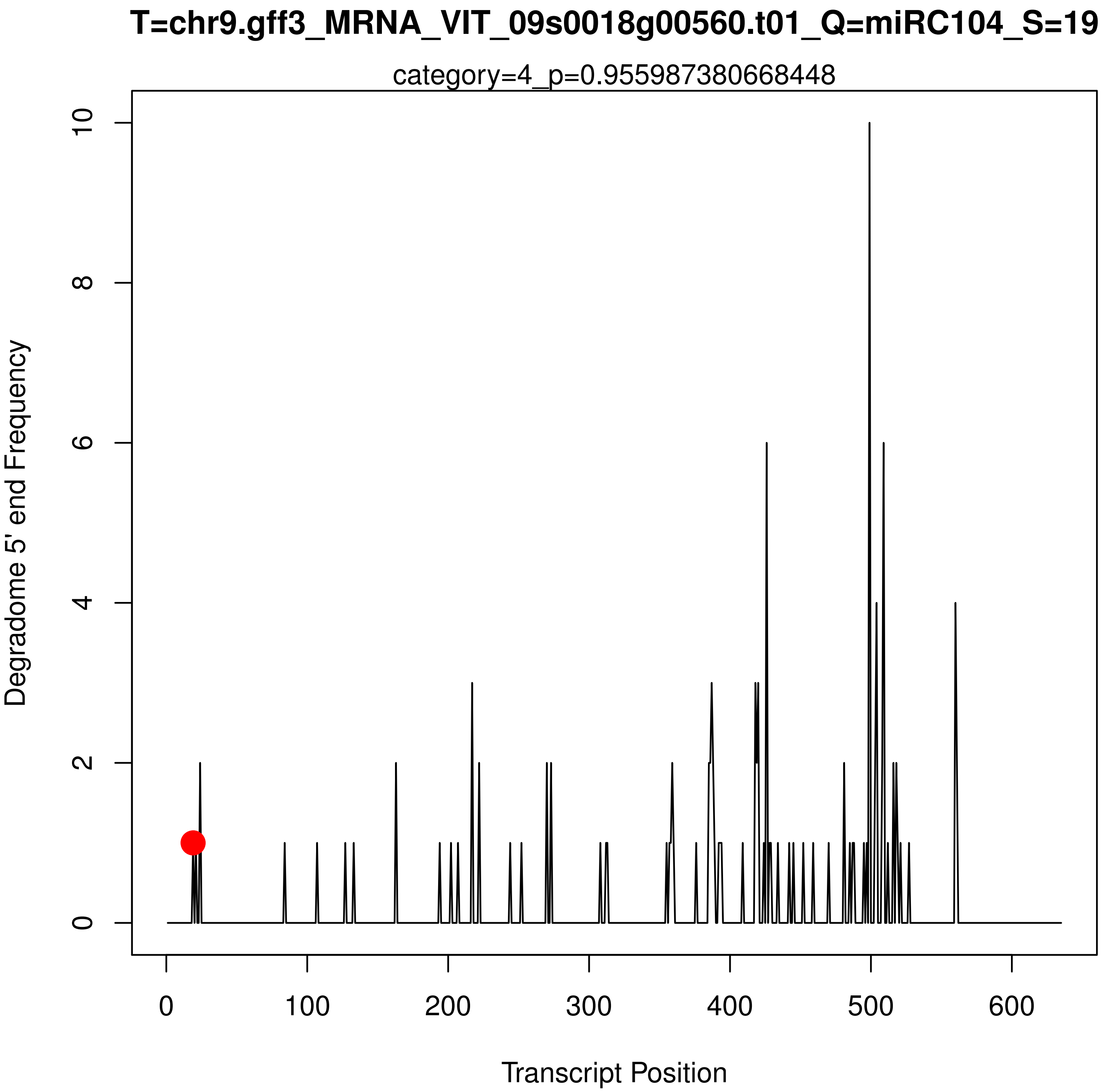
T=chr9.gff3_MRNA_VIT_09s0018g00560.t01_Q=miRC104_S=19
category=4_p=0.955987380668448
Degradome 5' end Frequency
0
2
4
6
8
10
0
100
200
300
400
500
600
Transcript Position

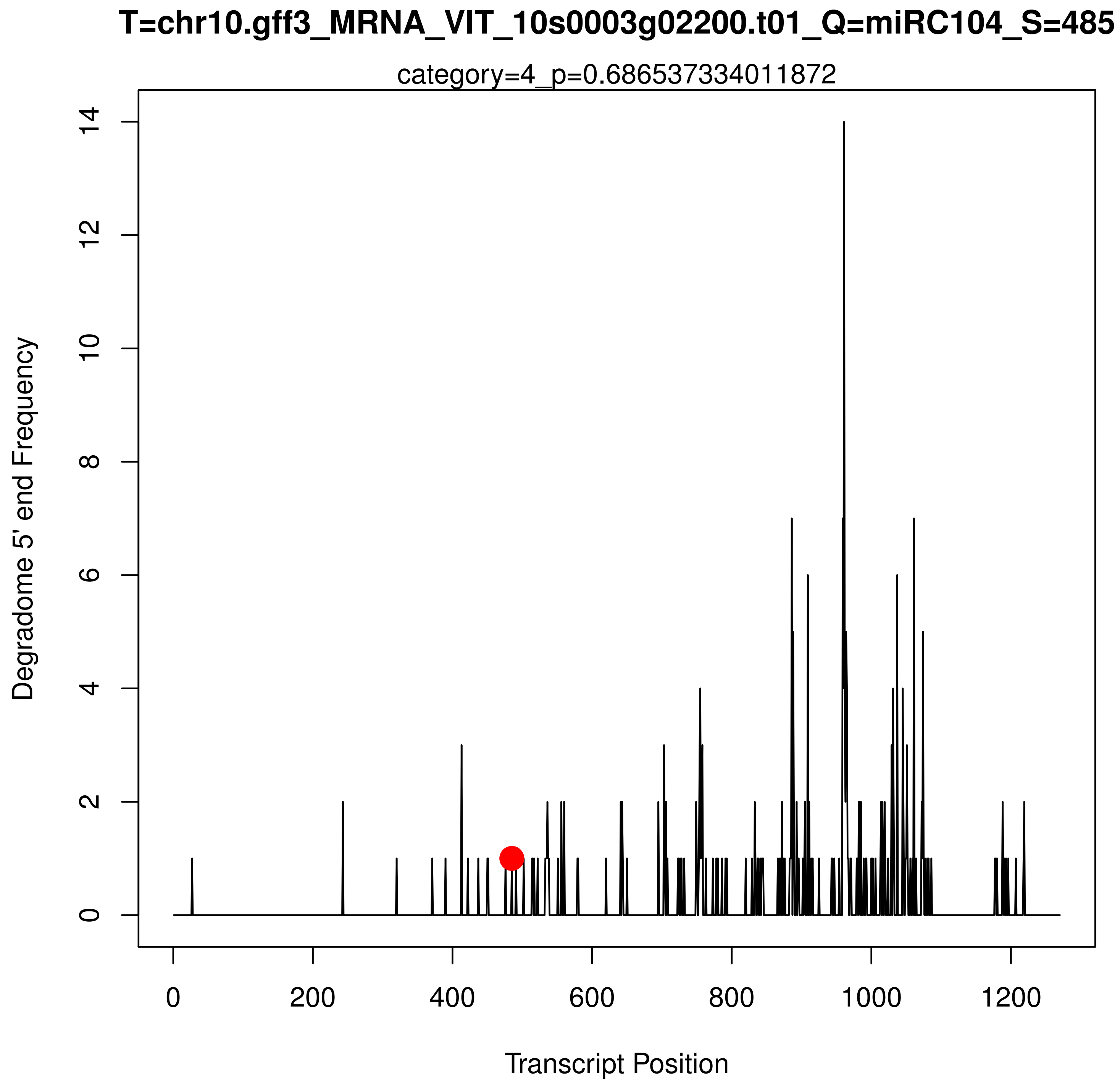
T=chr10.gff3_MRNA_VIT_10s0003g02200.t01_Q=miRC104_S=485
category=4_p=0.686537334011872
Degradome 5' end Frequency
Transcript Position
0
2
4
6
8
10
12
14
0
200
400
600
800
1000
1200

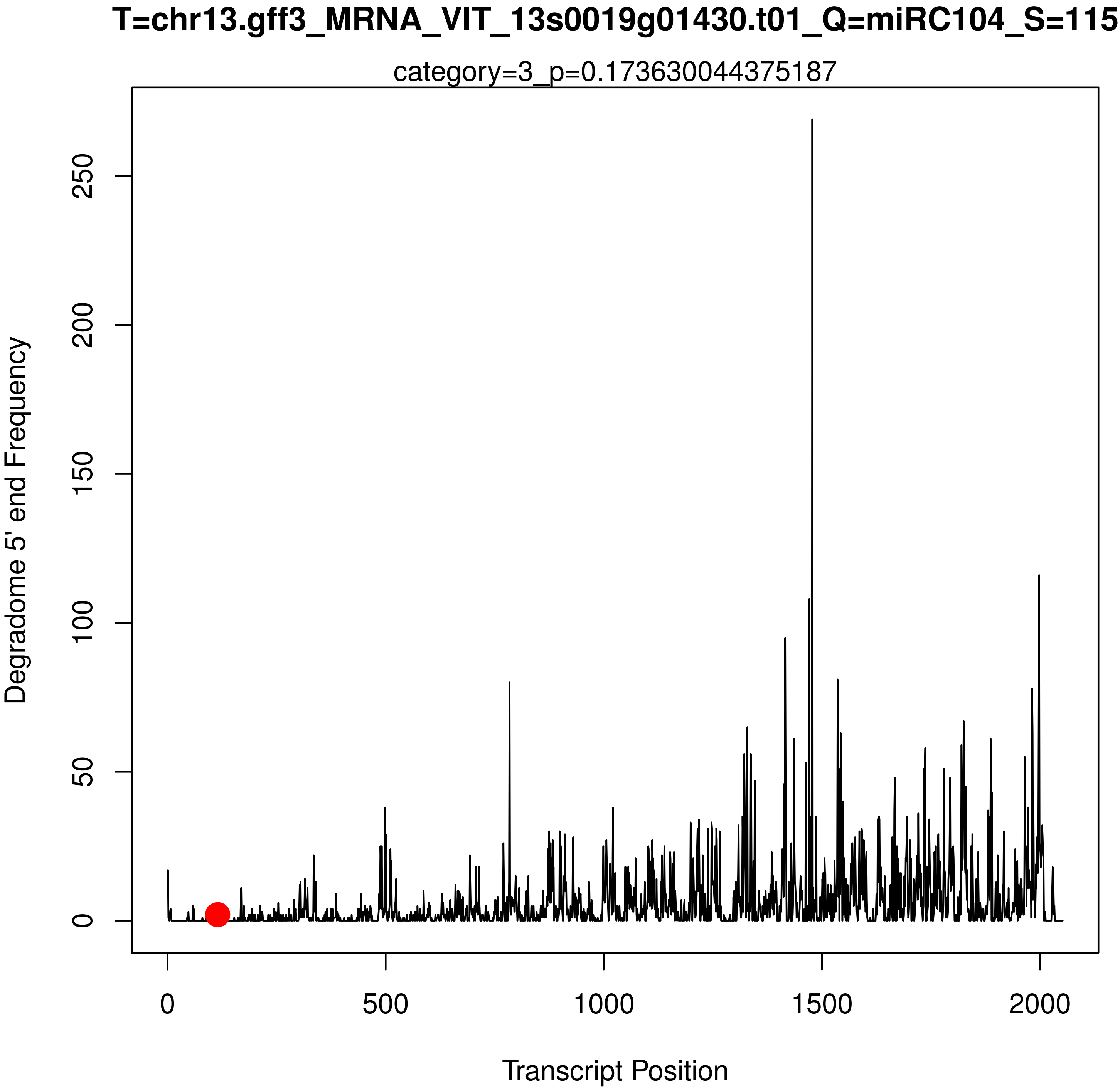

T=chr13.gff3_MRNA_VIT_13s0019g01430.t01_Q=miRC104_S=115
category=3_p=0.173630044375187
Degradome 5' end Frequency
0
50
100
150
200
250
0
500
1000
1500
2000
Transcript Position

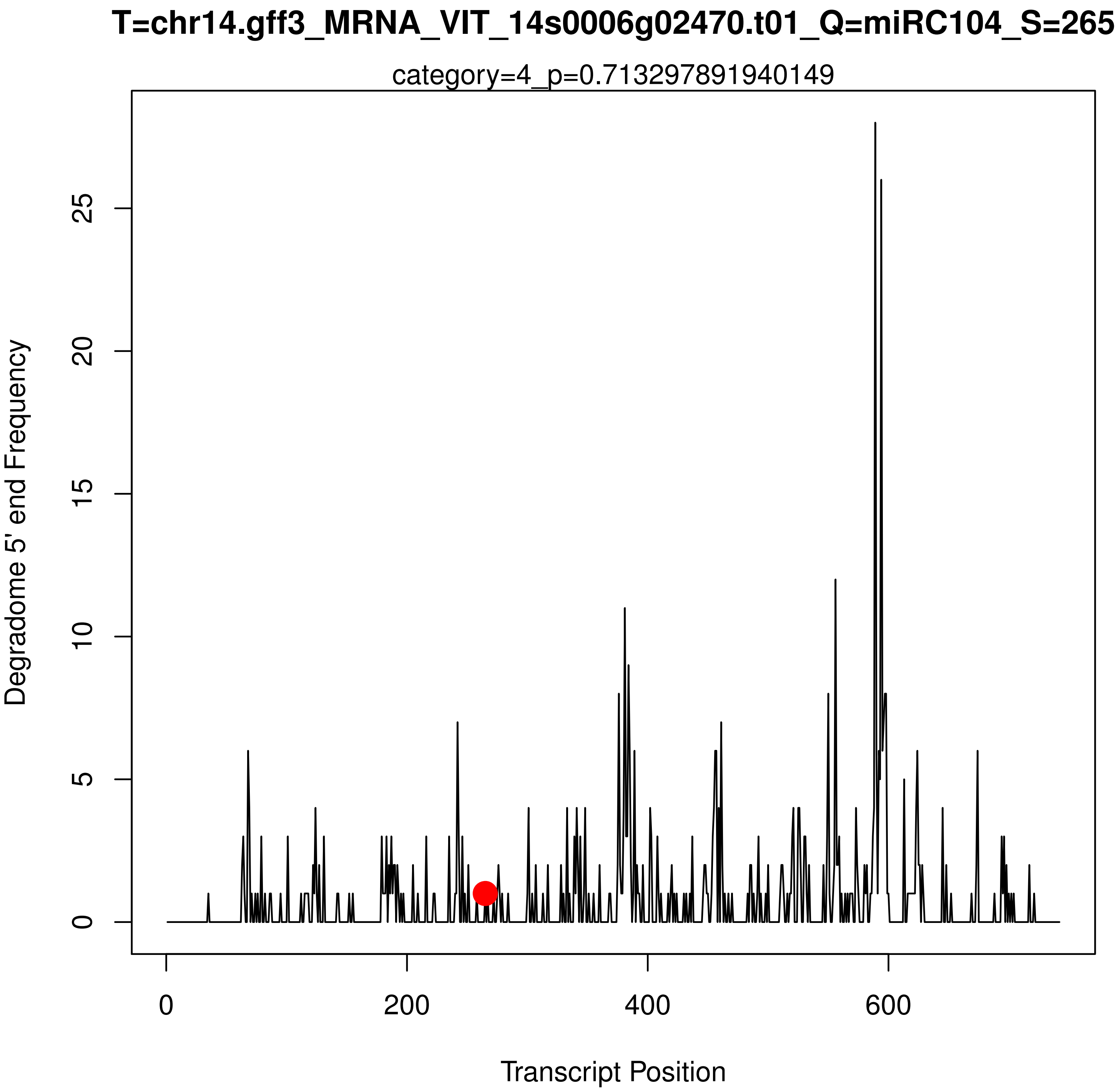
T=chr14.gff3_MRNA_VIT_14s0006g02470.t01_Q=miRC104_S=265
category=4_p=0.713297891940149
Degradome 5' end Frequency
0
5
10
15
20
25
0
200
400
600
Transcript Position

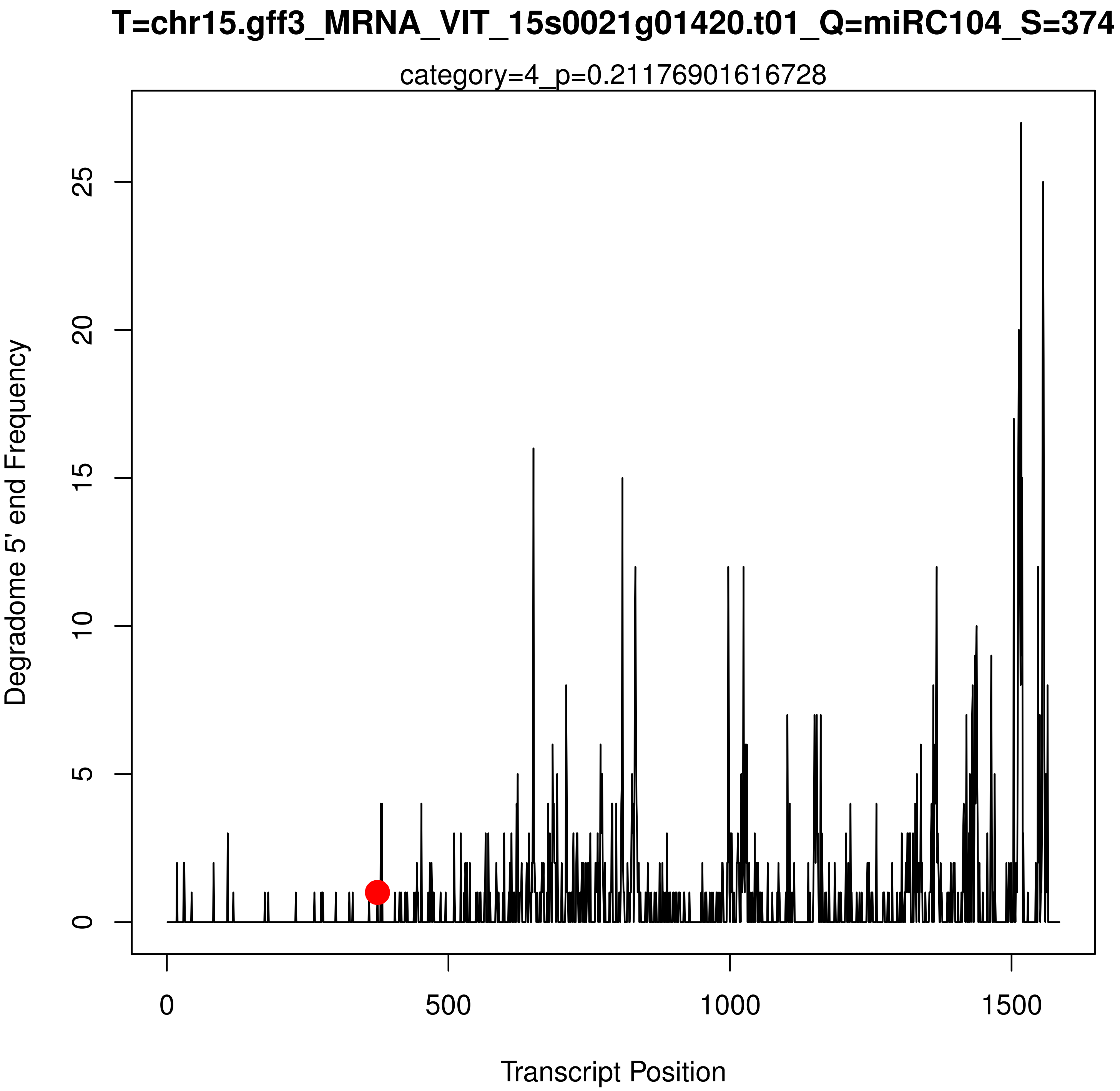

T=chr15.gff3_MRNA_VIT_15s0021g01420.t01_Q=miRC104_S=374
category=4_p=0.21176901616728
Degradome 5' end Frequency
Transcript Position
0
500
1000
1500
0
5
10
15
20
25

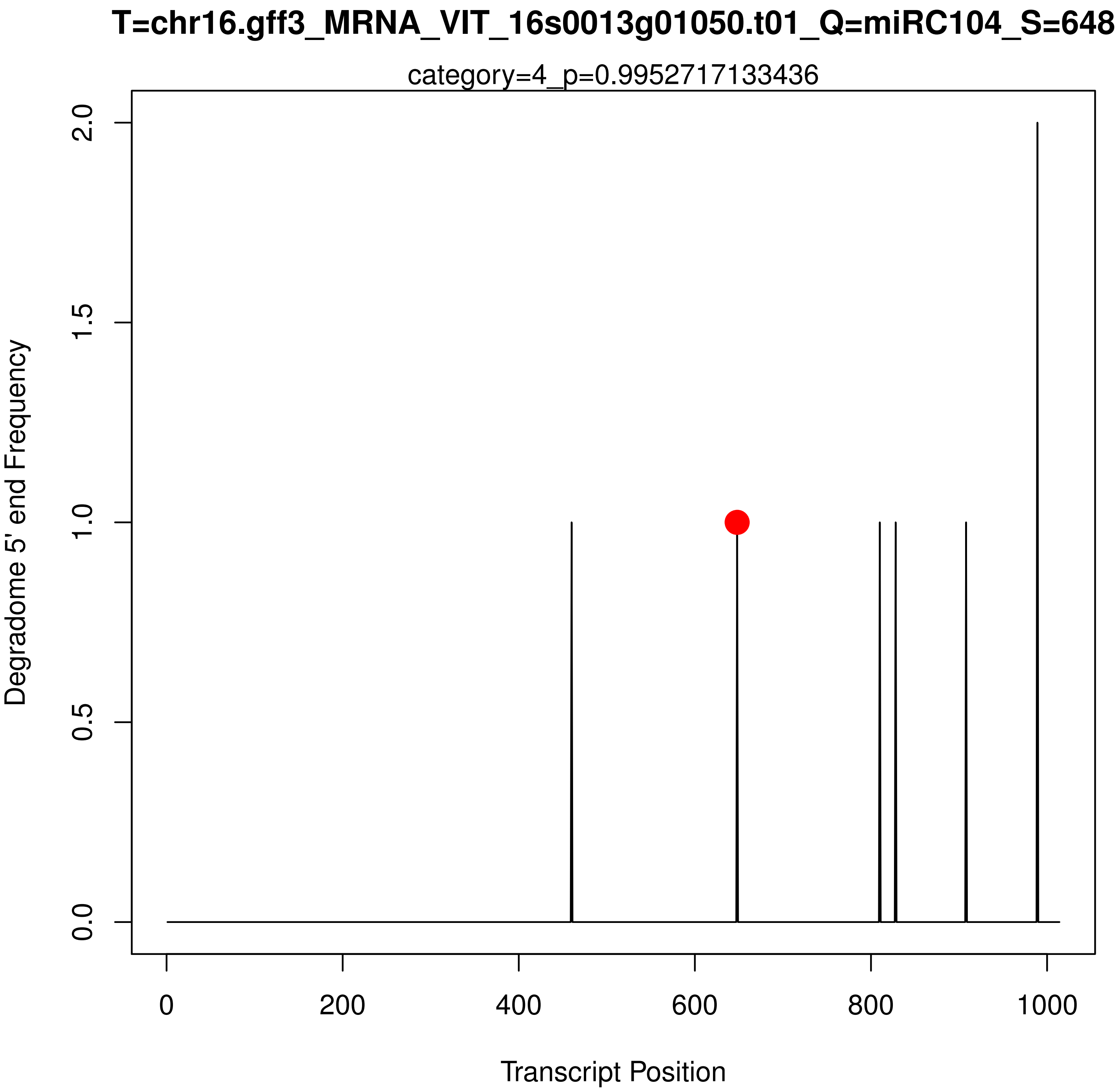
T=chr16.gff3_MRNA_VIT_16s0013g01050.t01_Q=miRC104_S=648
category=4_p=0.9952717133436
Degradome 5' end Frequency
0.0
0.5
1.0
1.5
2.0
0
200
400
600
800
1000
Transcript Position

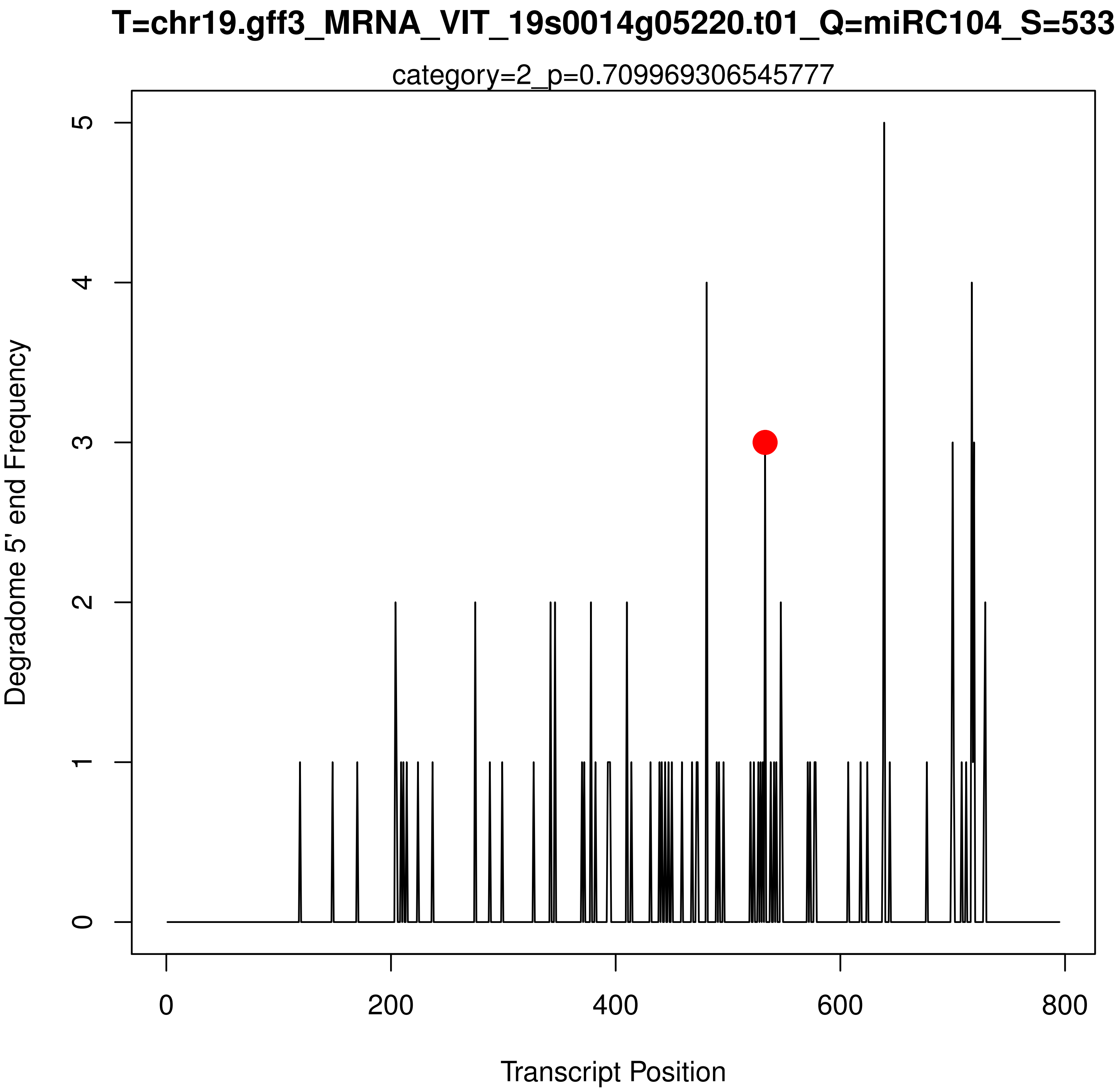

T=chr19.gff3_MRNA_VIT_19s0014g05220.t01_Q=miRC104_S=533
category=2_p=0.709969306545777
Degradome 5' end Frequency
0
1
2
3
4
5
0
200
400
600
800
Transcript Position

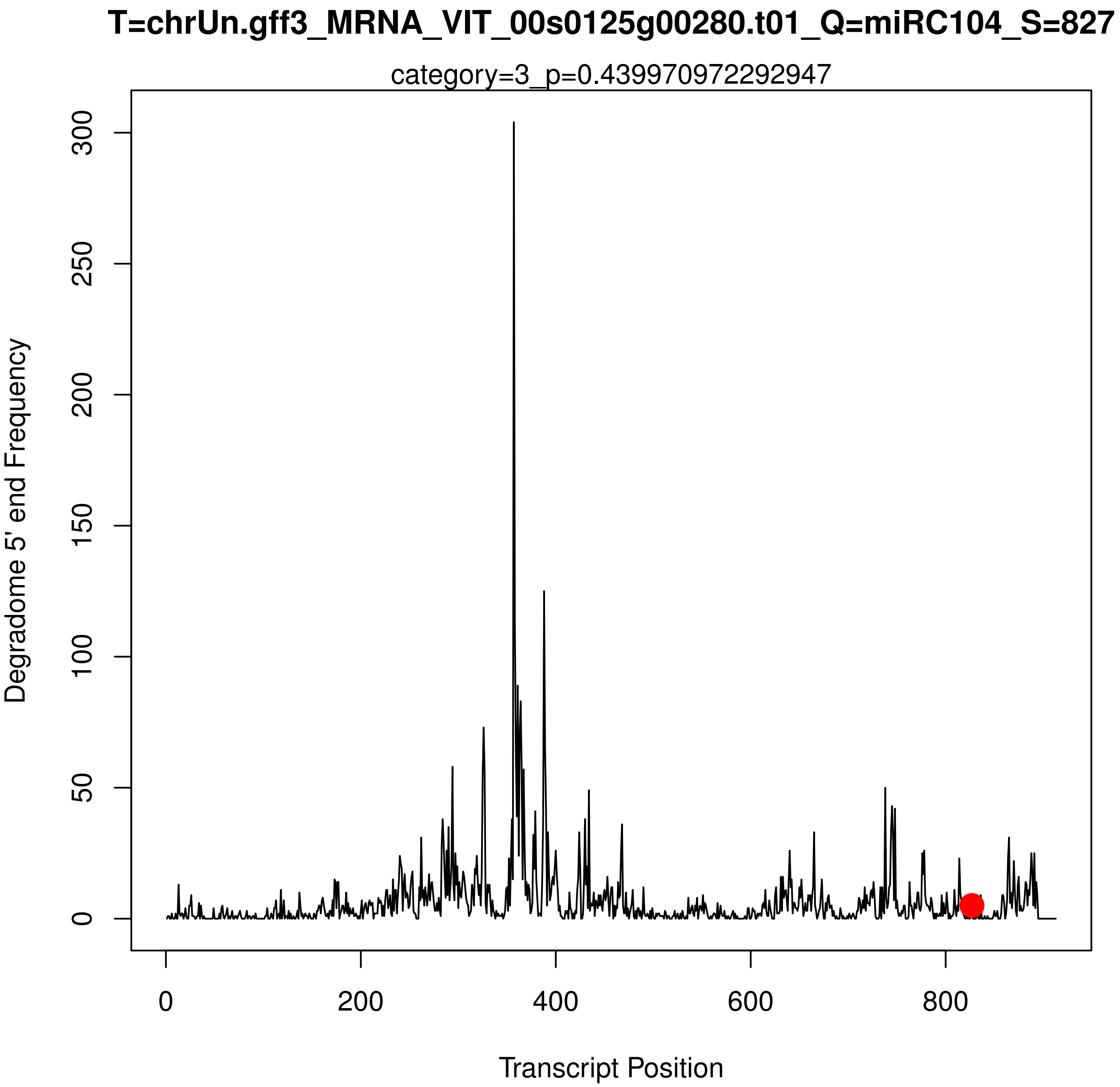
T=chrUn.gff3_MRNA_VIT_00s0125g00280.t01_Q=miRC104_S=827
category=3_p=0.439970972292947
Degradome 5' end Frequency
0
50
100
150
200
250
300
0
200
400
600
800
Transcript Position

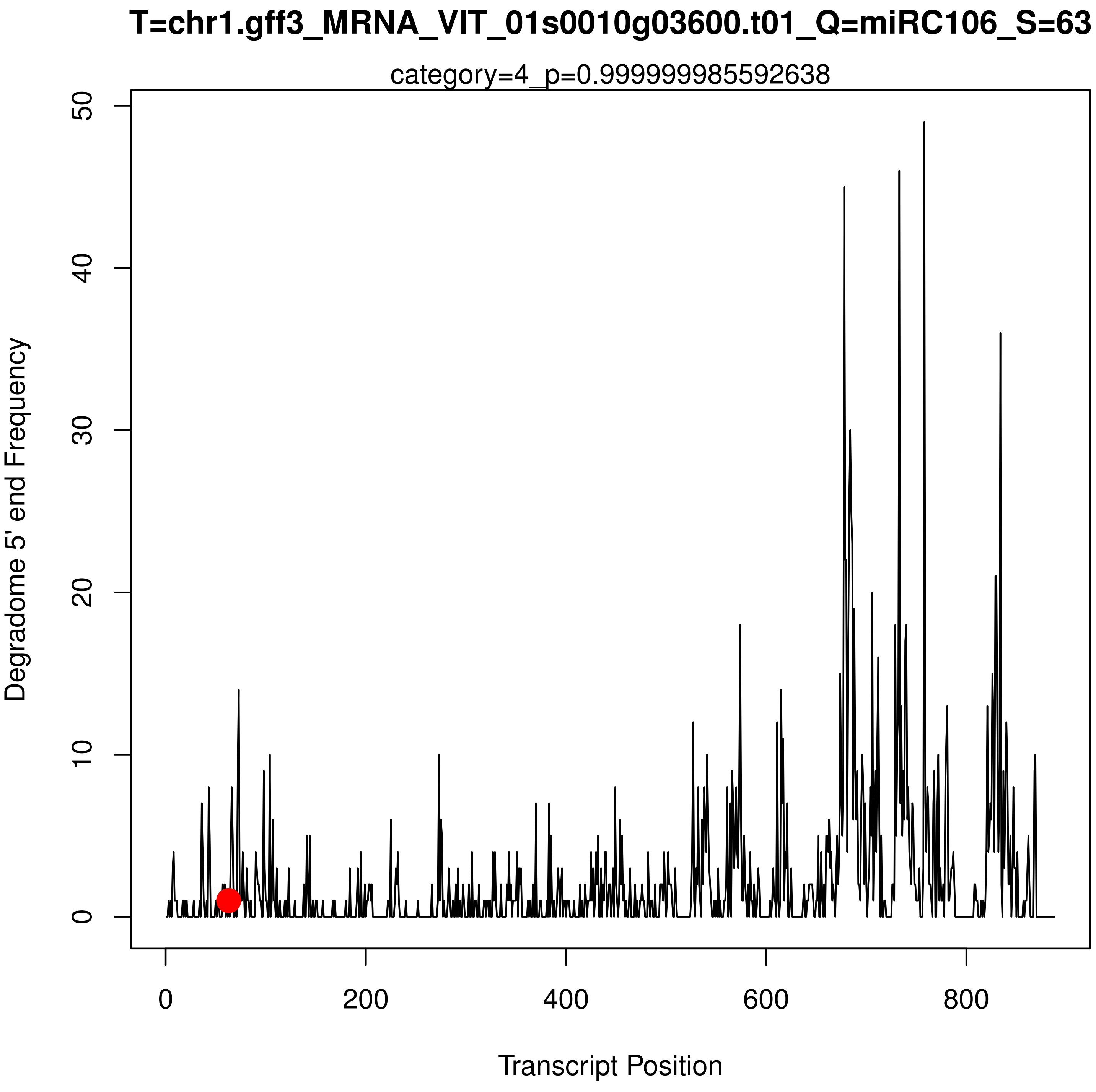
T=chr1.gff3_MRNA_VIT_01s0010g03600.t01_Q=miRC106_S=63
category=4_p=0.999999985592638
Degradome 5' end Frequency
0
10
20
30
40
50
0
200
400
600
800
Transcript Position

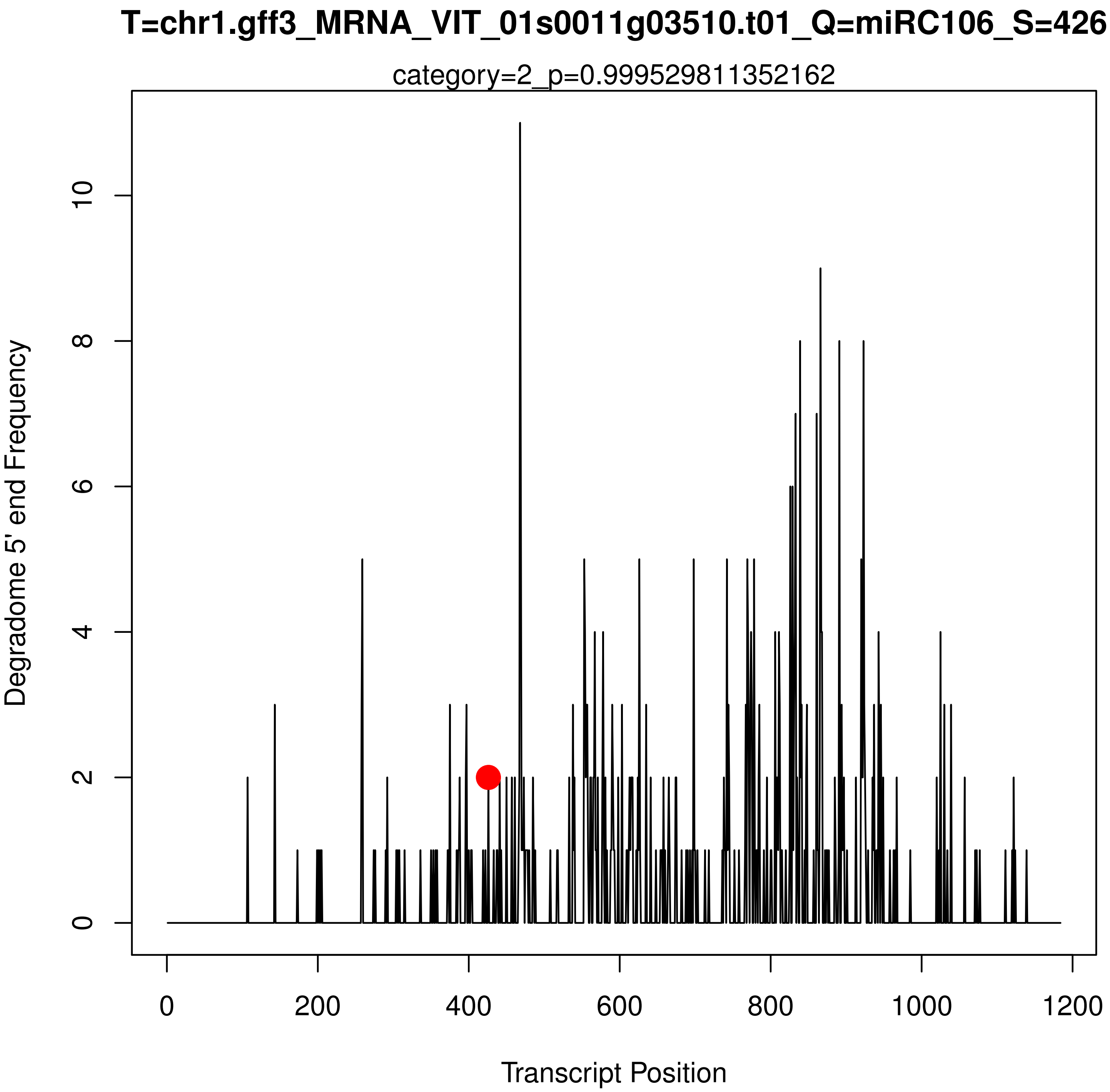

T=chr1.gff3_MRNA_VIT_01s0011g03510.t01_Q=miRC106_S=426
category=2_p=0.999529811352162
Degradome 5' end Frequency
0
2
4
6
8
10
0
200
400
600
800
1000
1200
Transcript Position

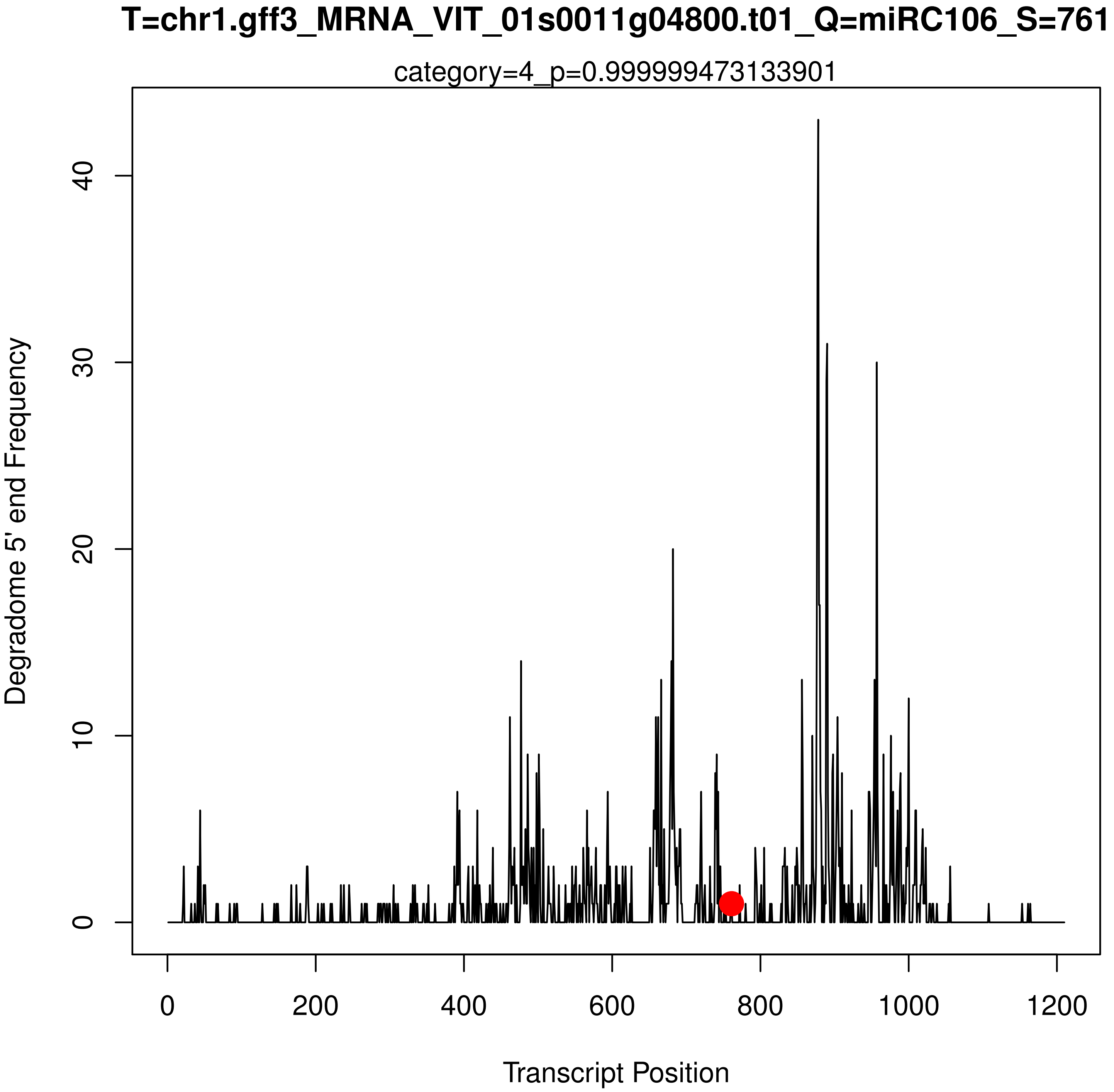
T=chr1.gff3_MRNA_VIT_01s0011g04800.t01_Q=miRC106_S=761
category=4_p=0.999999473133901
Degradome 5' end Frequency
0
10
20
30
40
0
200
400
600
800
1000
1200
Transcript Position

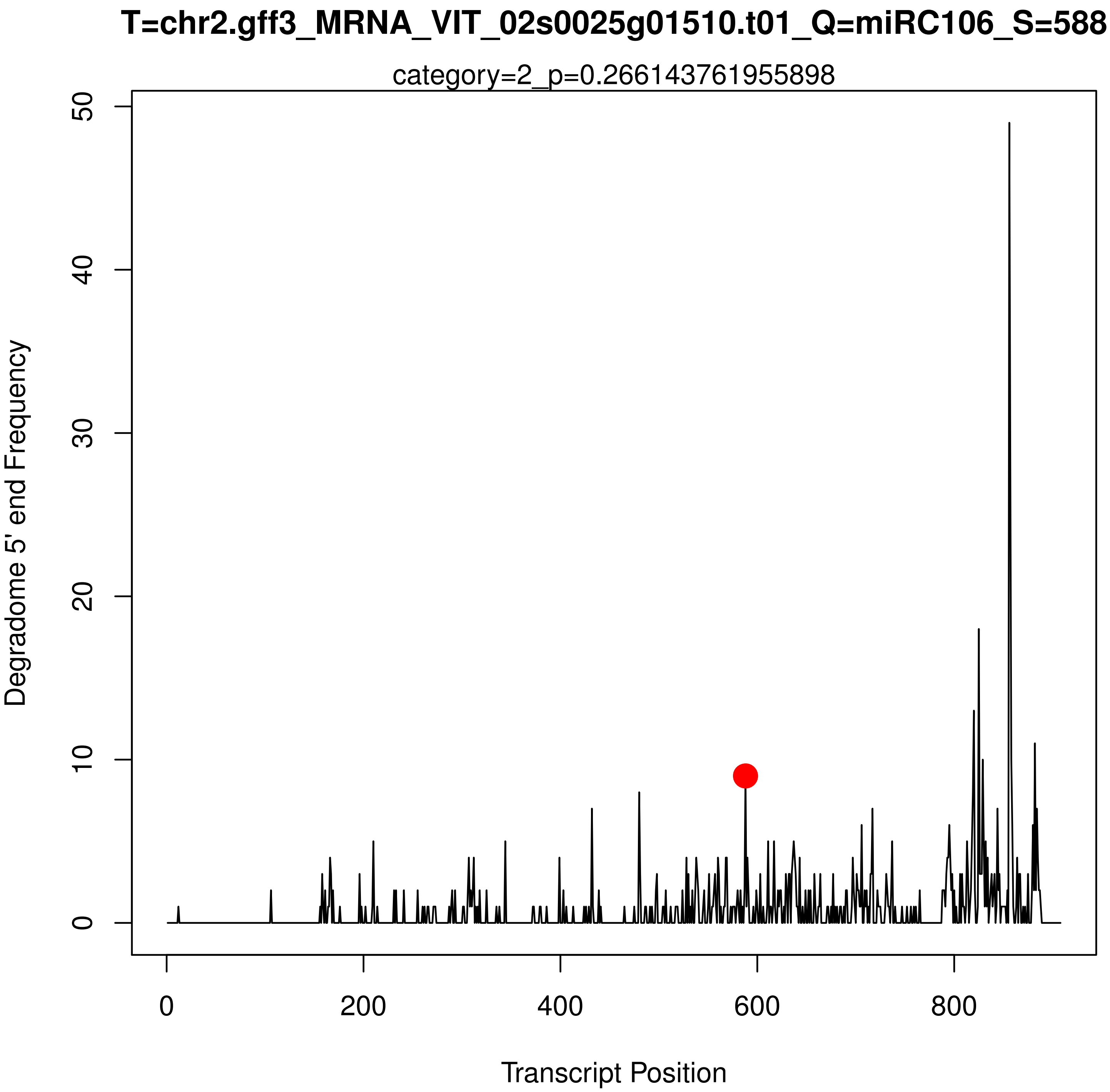
T=chr2.gff3_MRNA_VIT_02s0025g01510.t01_Q=miRC106_S=588
category=2_p=0.266143761955898
Degradome 5' end Frequency
Transcript Position
0
10
20
30
40
50
0
200
400
600
800

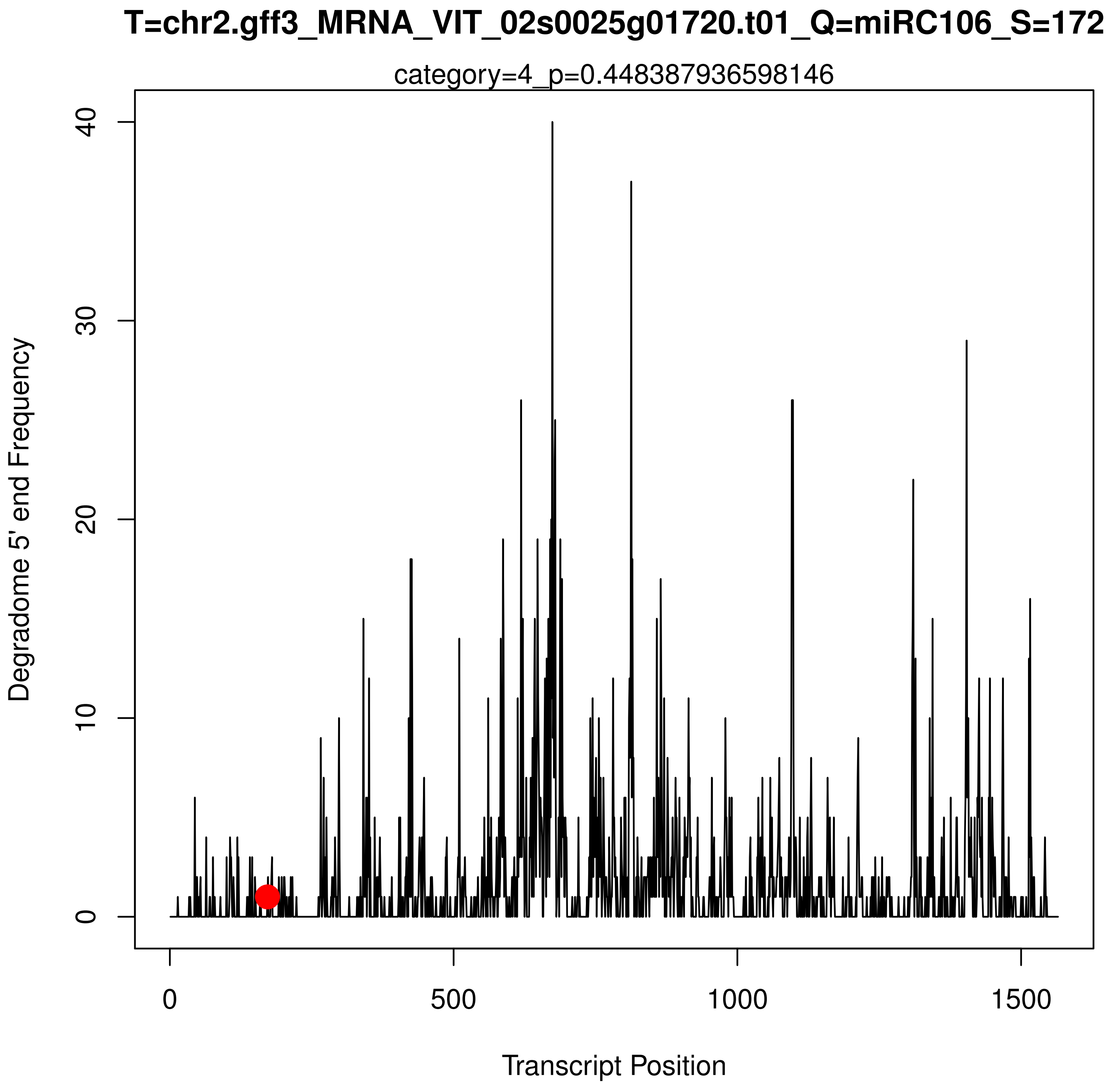
T=chr2.gff3_MRNA_VIT_02s0025g01720.t01_Q=miRC106_S=172
category=4_p=0.448387936598146
Degradome 5' end Frequency
0
10
20
30
40
0
500
1000
1500
Transcript Position

**T=chr2.gff3_MRNA_VIT_02s0109g00380.t01_Q=miRC106_S=606**

category=4_p=0.999999993735712

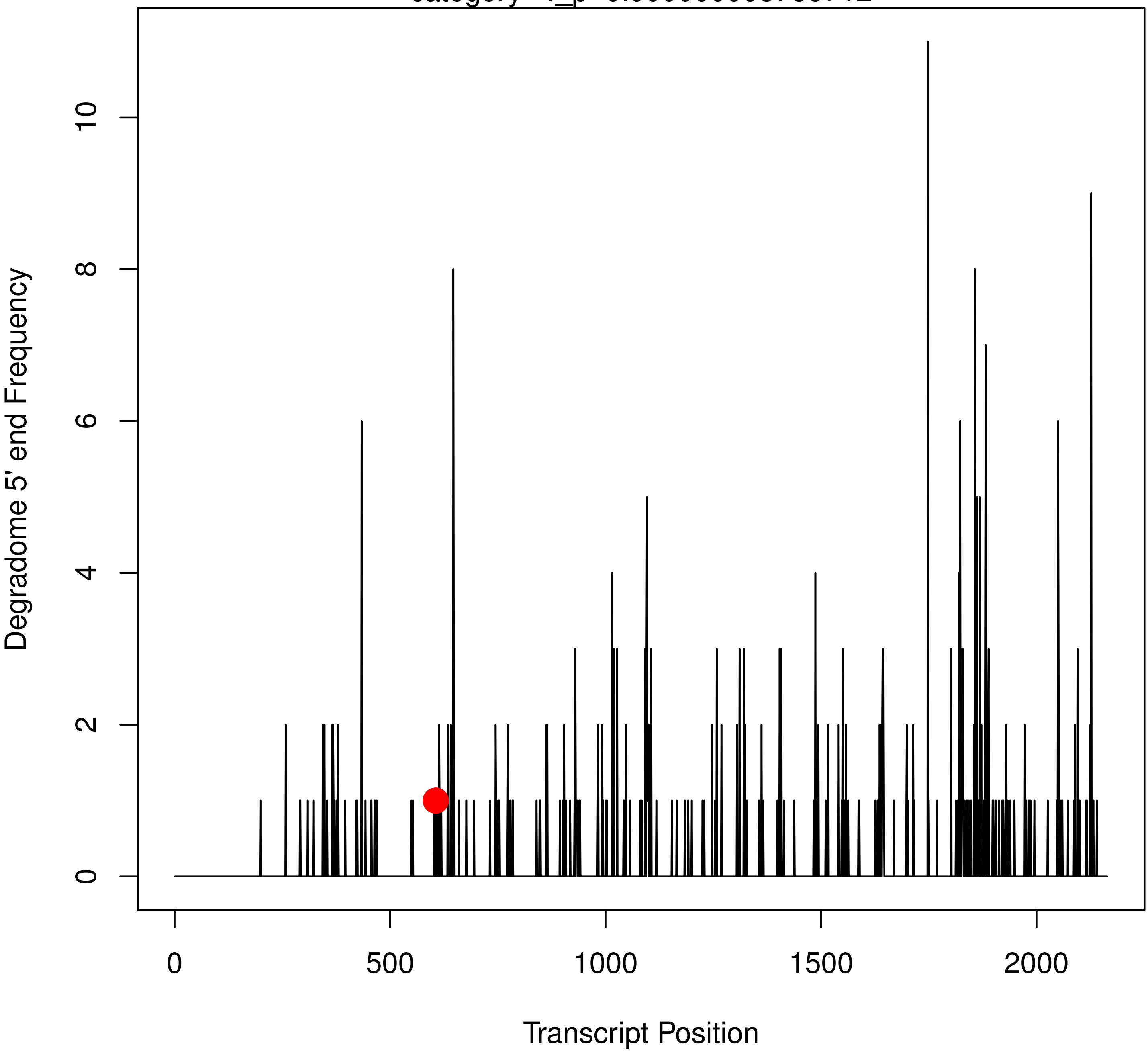

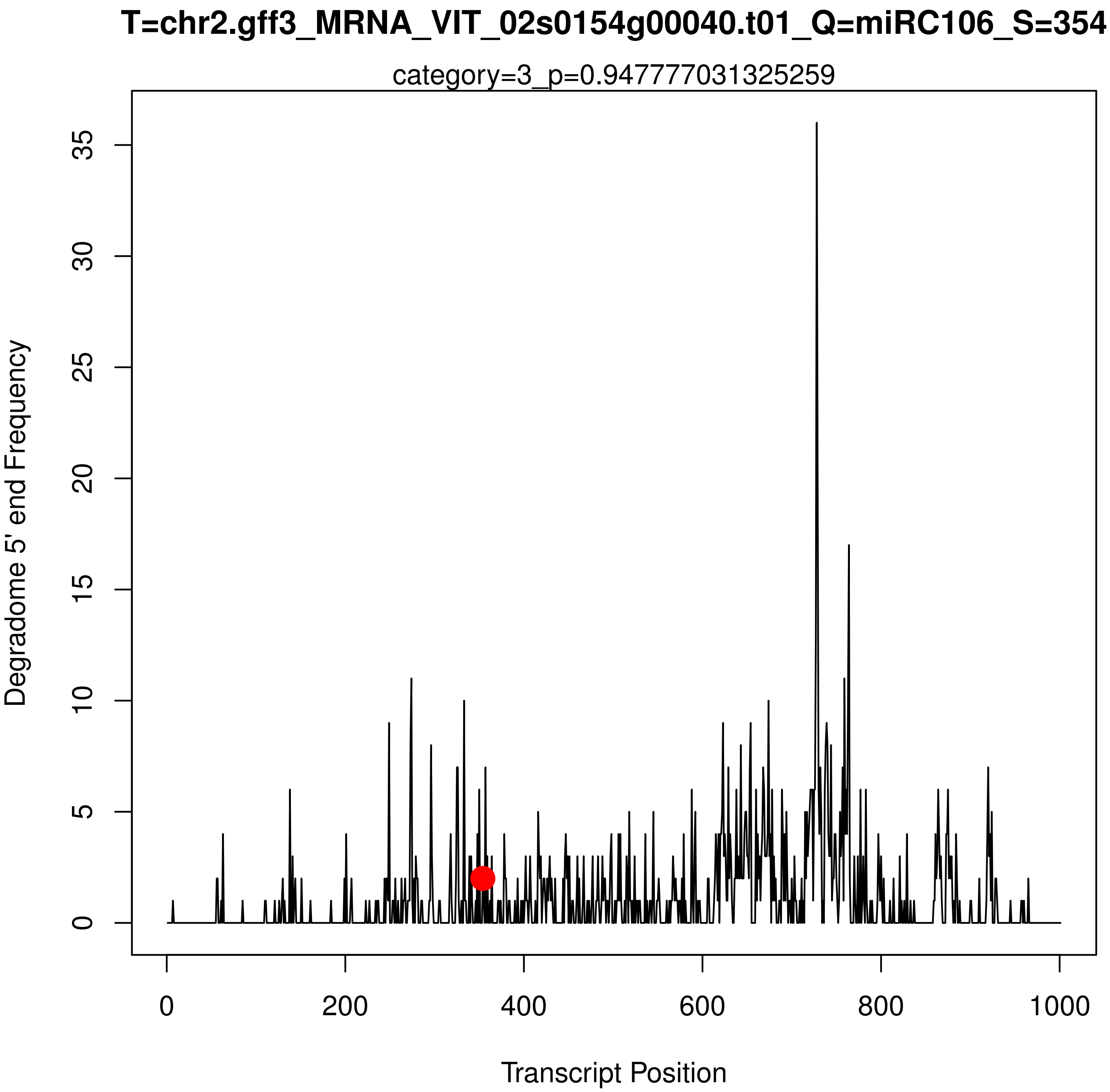
T=chr2.gff3_MRNA_VIT_02s0154g00040.t01_Q=miRC106_S=354
category=3_p=0.947777031325259
Degradome 5' end Frequency
Transcript Position
0
5
10
15
20
25
30
35
0
200
400
600
800
1000

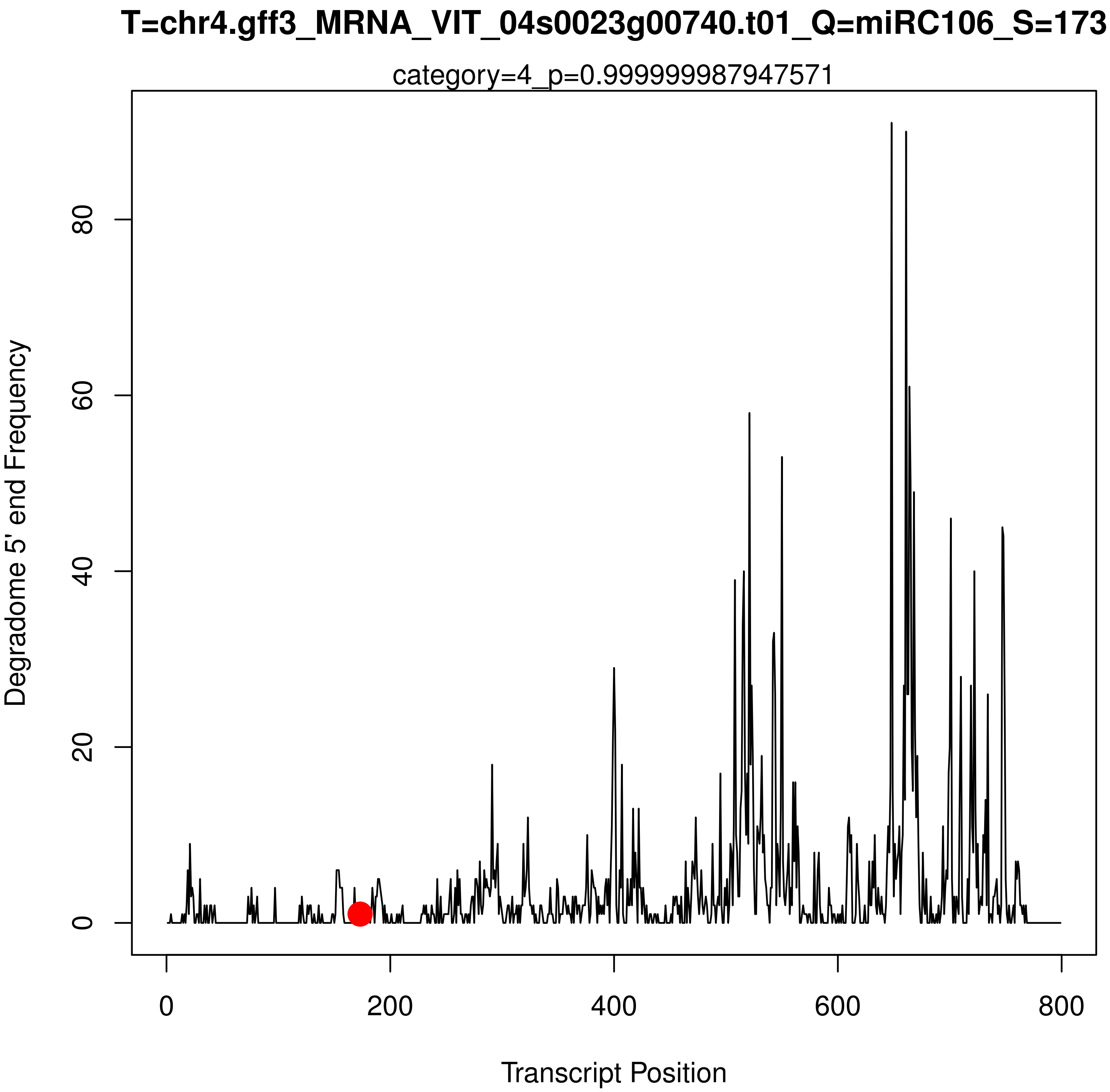
T=chr4.gff3_MRNA_VIT_04s0023g00740.t01_Q=miRC106_S=173
category=4_p=0.999999987947571
Degradome 5' end Frequency
0
20
40
60
80
0
200
400
600
800
Transcript Position

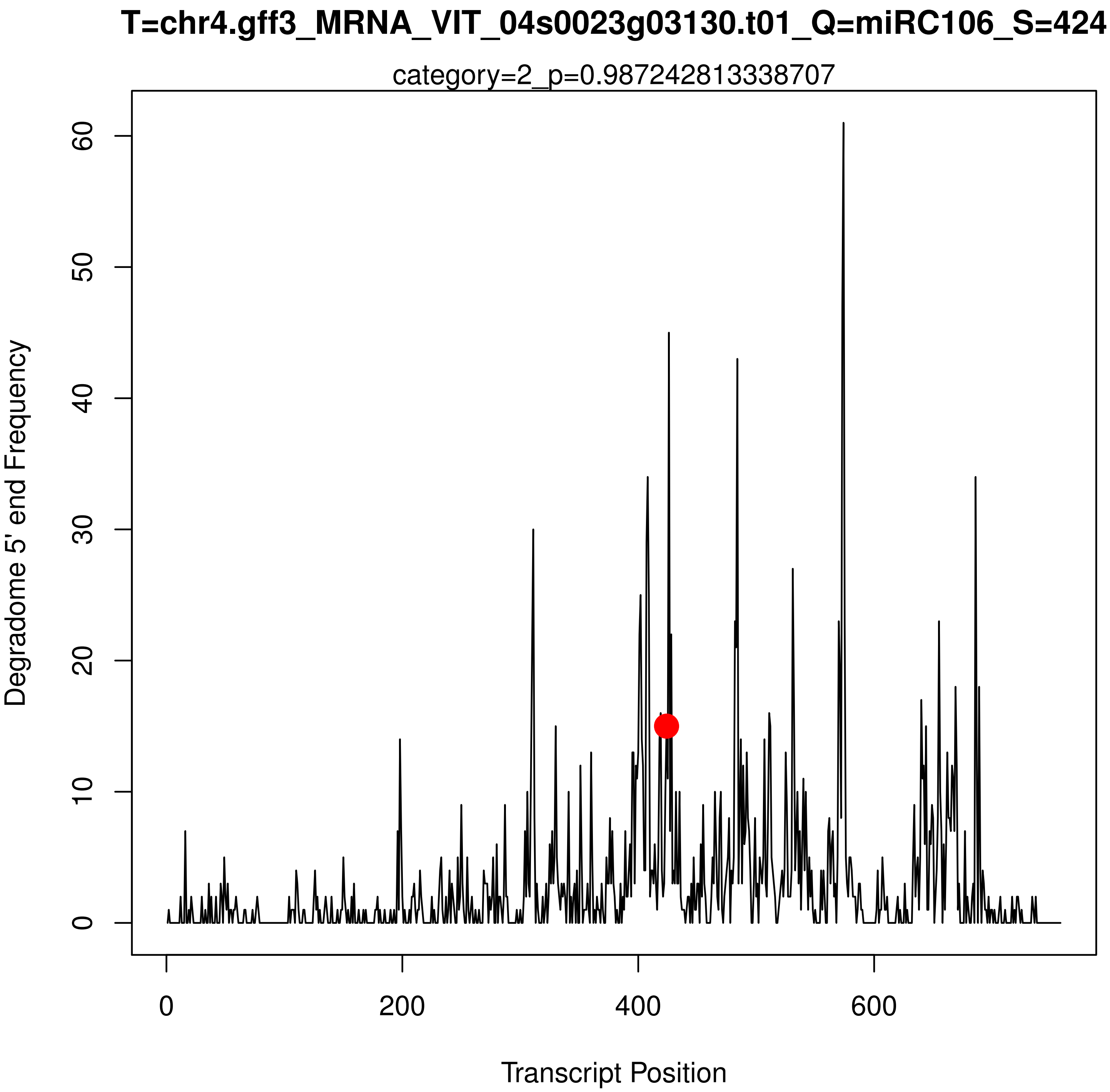
T=chr4.gff3_MRNA_VIT_04s0023g03130.t01_Q=miRC106_S=424
category=2_p=0.987242813338707
Degradome 5' end Frequency
Transcript Position
0
10
20
30
40
50
60
0
200
400
600

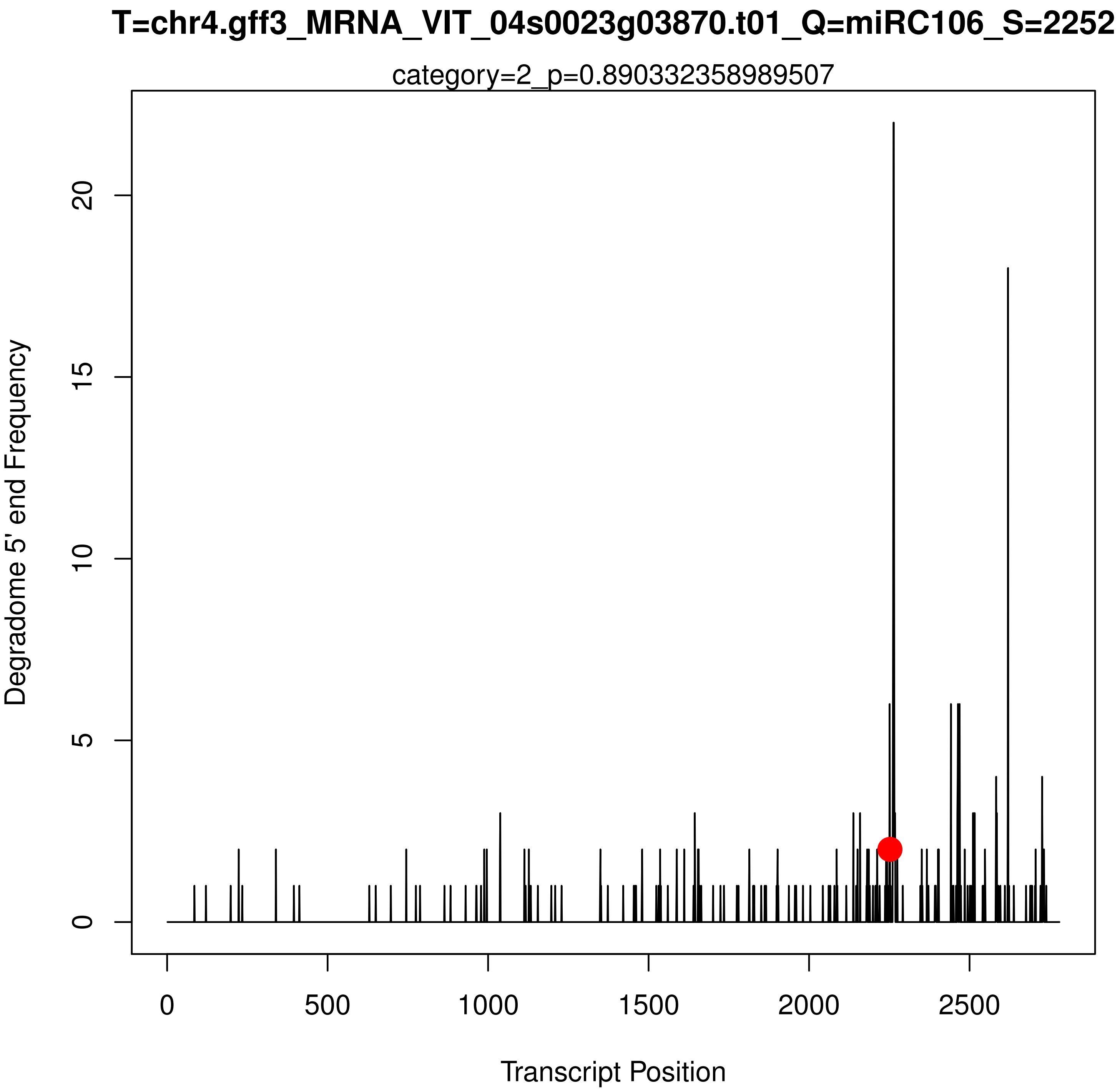
T=chr4.gff3_MRNA_VIT_04s0023g03870.t01_Q=miRC106_S=2252
category=2_p=0.890332358989507
Degradome 5' end Frequency
0
5
10
15
20
0
500
1000
1500
2000
2500
Transcript Position

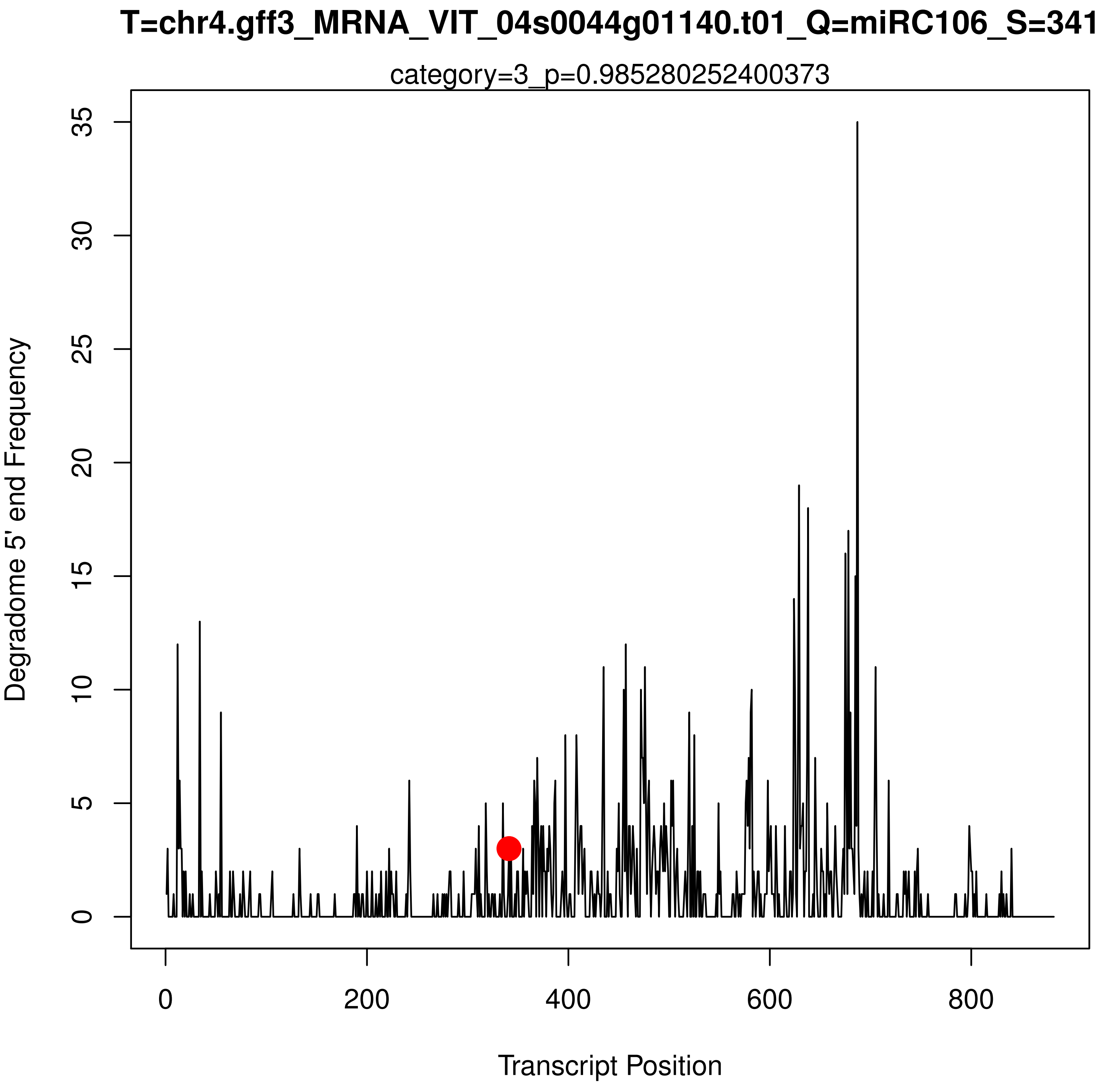

T=chr4.gff3_MRNA_VIT_04s0044g01140.t01_Q=miRC106_S=341
category=3_p=0.985280252400373
Degradome 5' end Frequency
0
5
10
15
20
25
30
35
0
200
400
600
800
Transcript Position

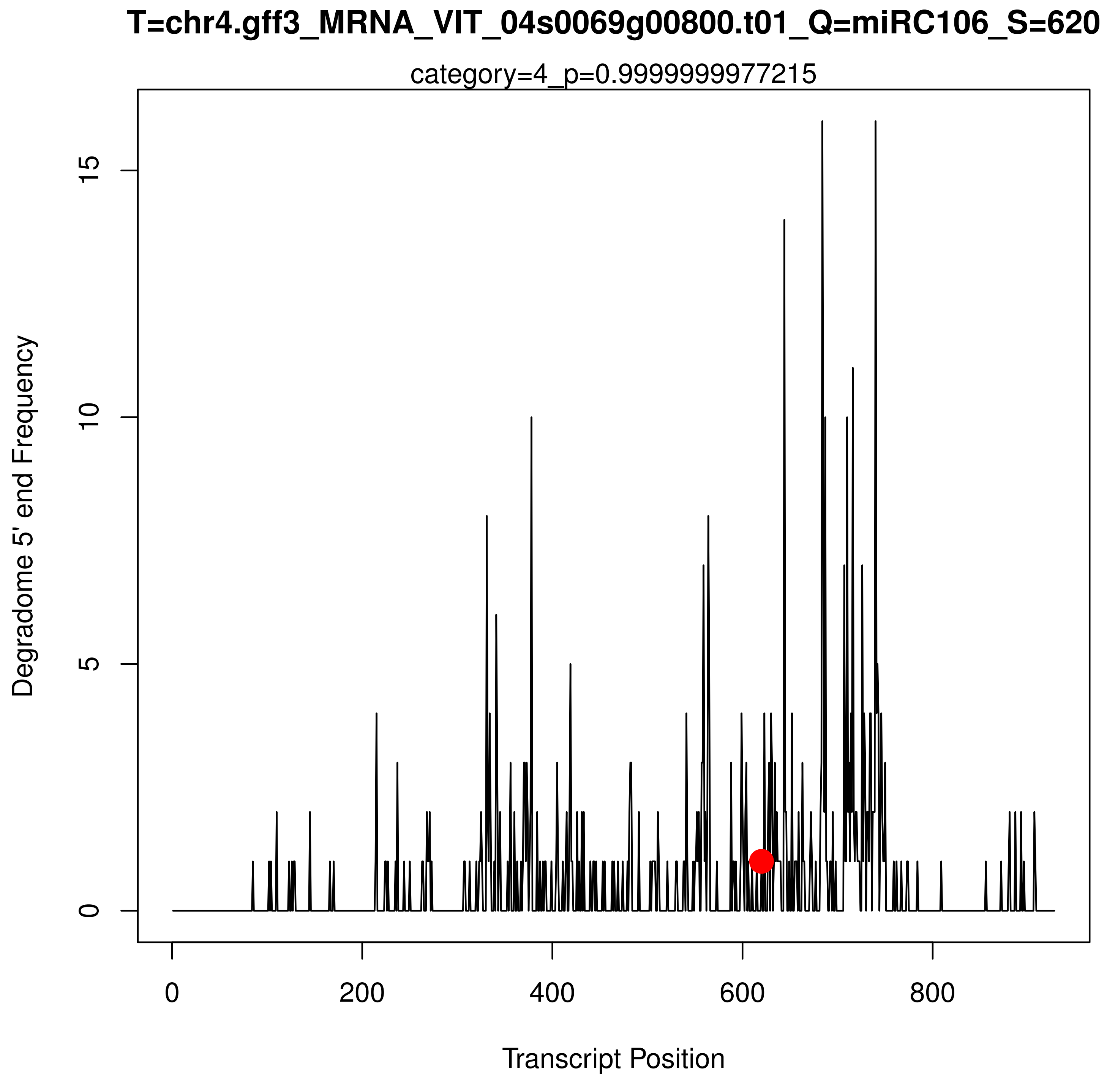

T=chr4.gff3_MRNA_VIT_04s0069g00800.t01_Q=miRC106_S=620
category=4_p=0.9999999977215
Degradome 5' end Frequency
Transcript Position
0
5
10
15
0
200
400
600
800

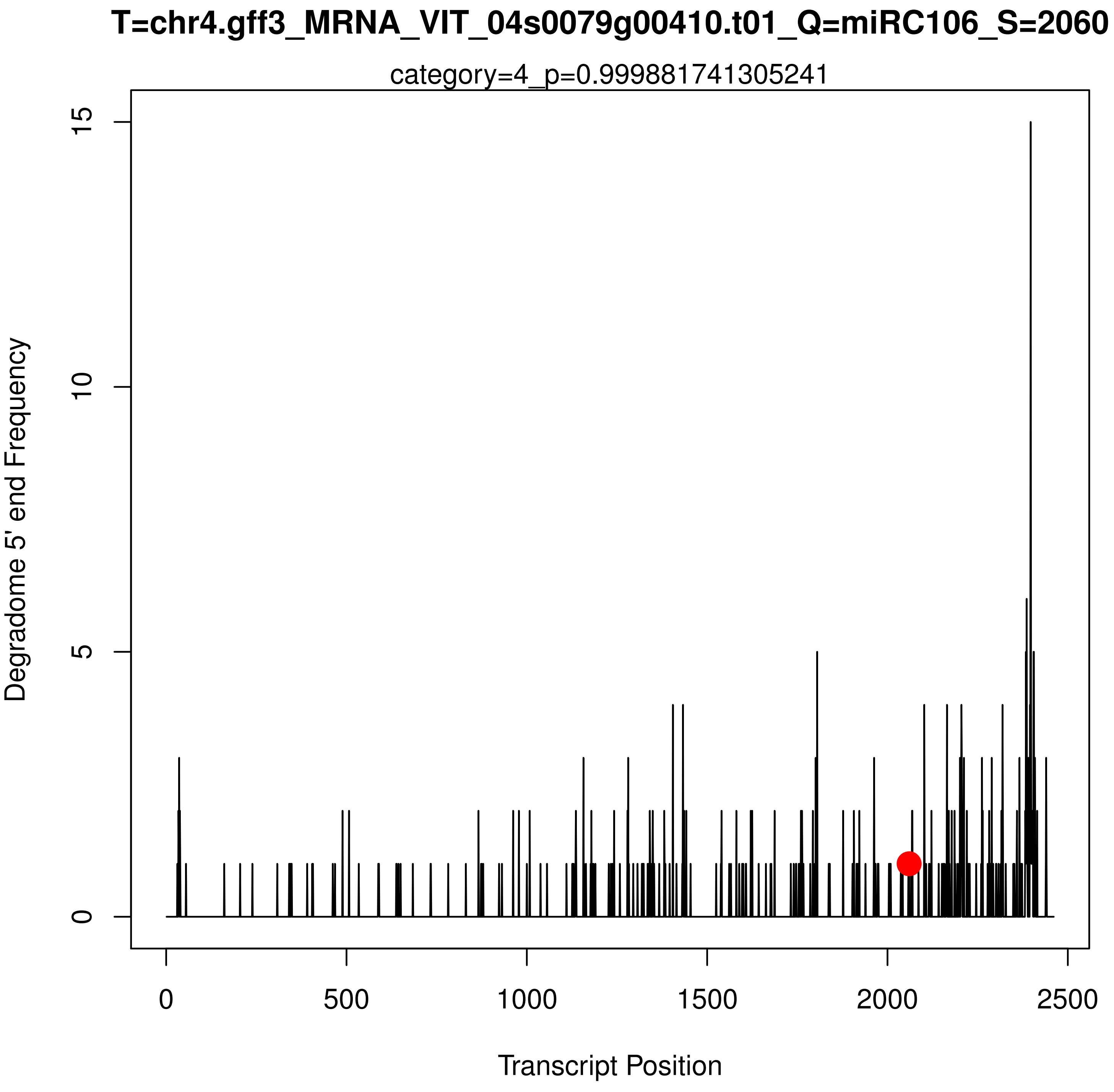
T=chr4.gff3_MRNA_VIT_04s0079g00410.t01_Q=miRC106_S=2060
category=4_p=0.999881741305241
Degradome 5' end Frequency
0
5
10
15
0
500
1000
1500
2000
2500
Transcript Position

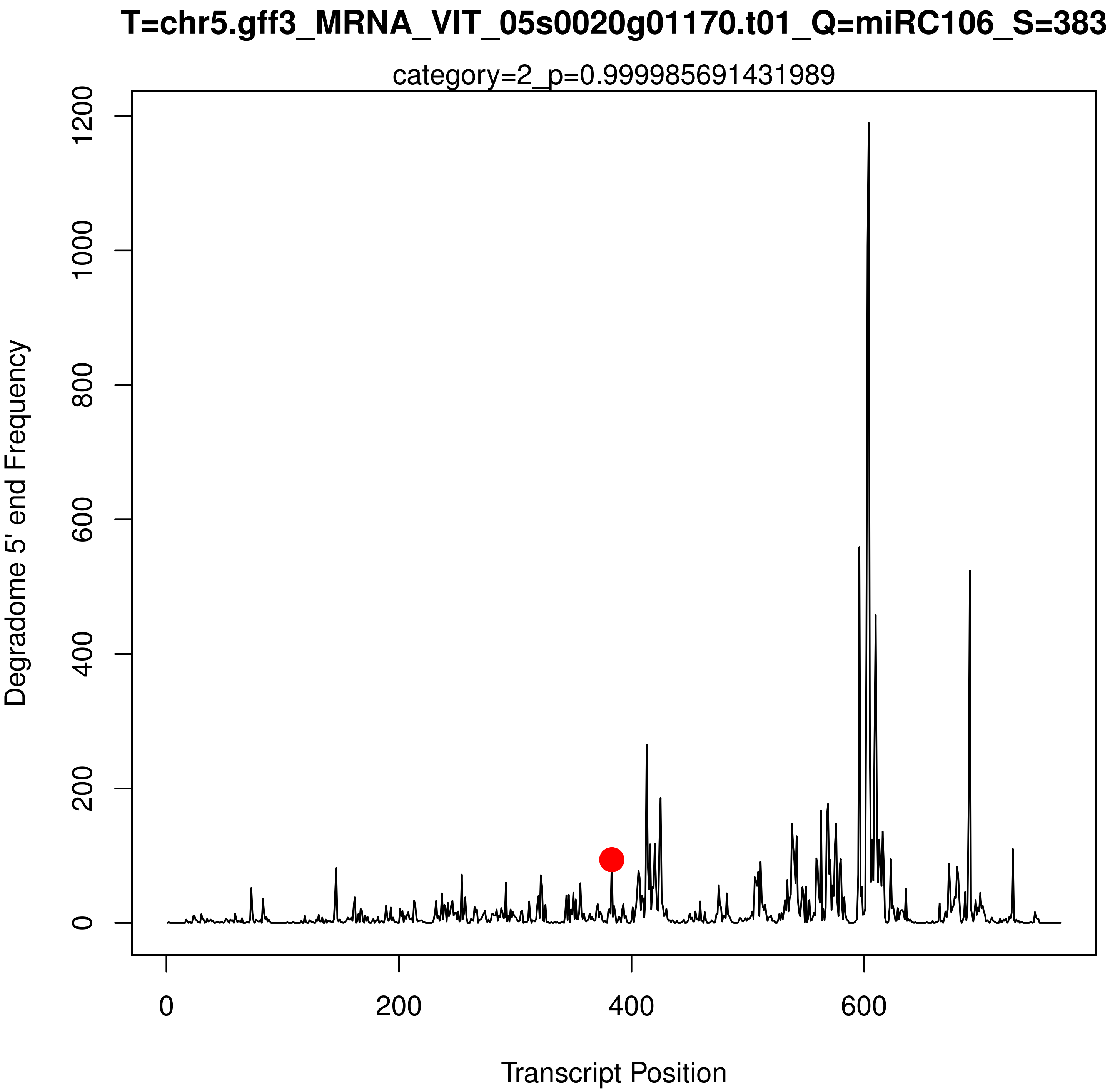
T=chr5.gff3_MRNA_VIT_05s0020g01170.t01_Q=miRC106_S=383
category=2_p=0.999985691431989
Degradome 5' end Frequency
Transcript Position
0
200
400
600
800
1000
1200

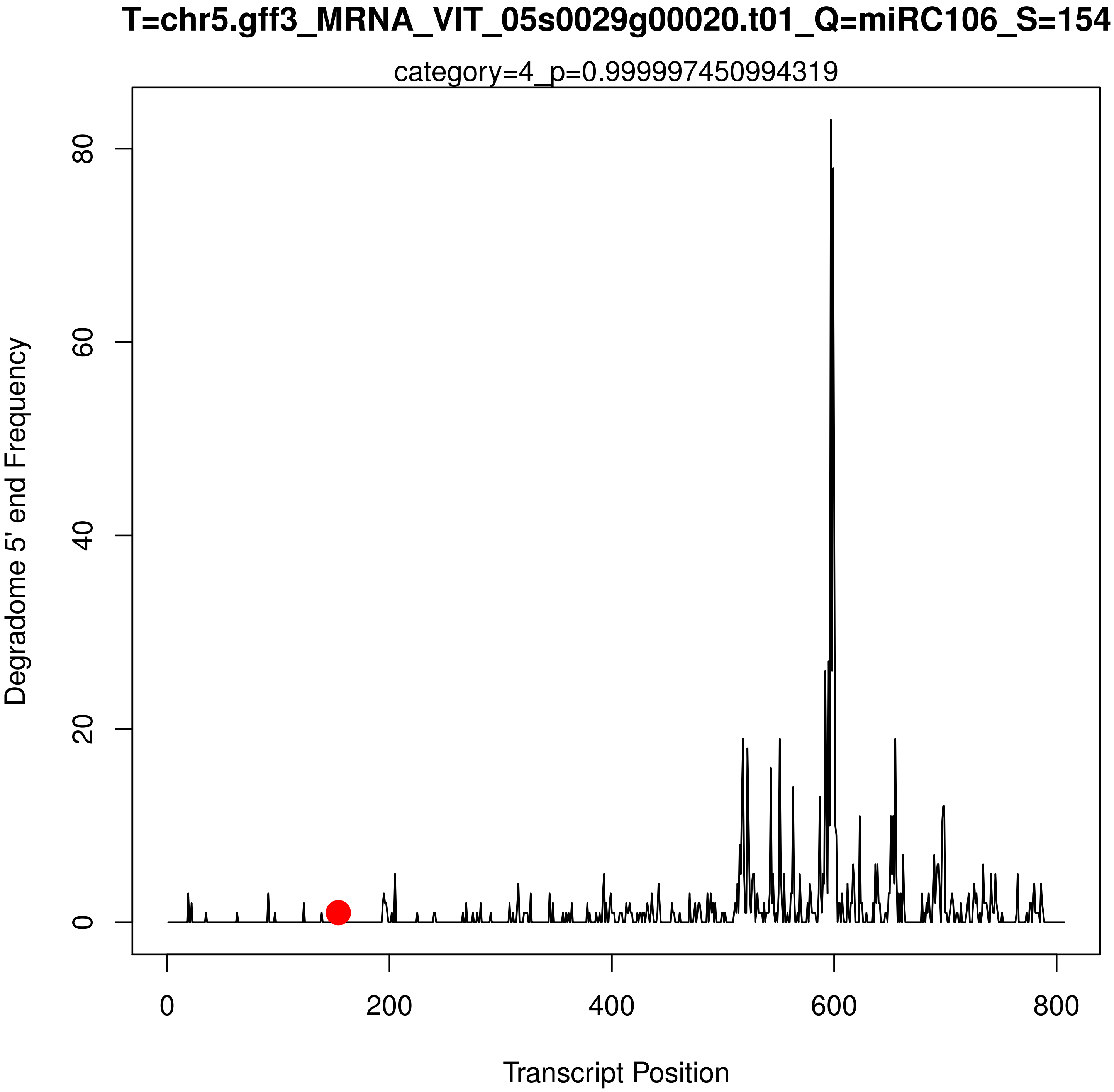
T=chr5.gff3_MRNA_VIT_05s0029g00020.t01_Q=miRC106_S=154
category=4_p=0.999997450994319
Degradome 5' end Frequency
0
20
40
60
80
0
200
400
600
800
Transcript Position

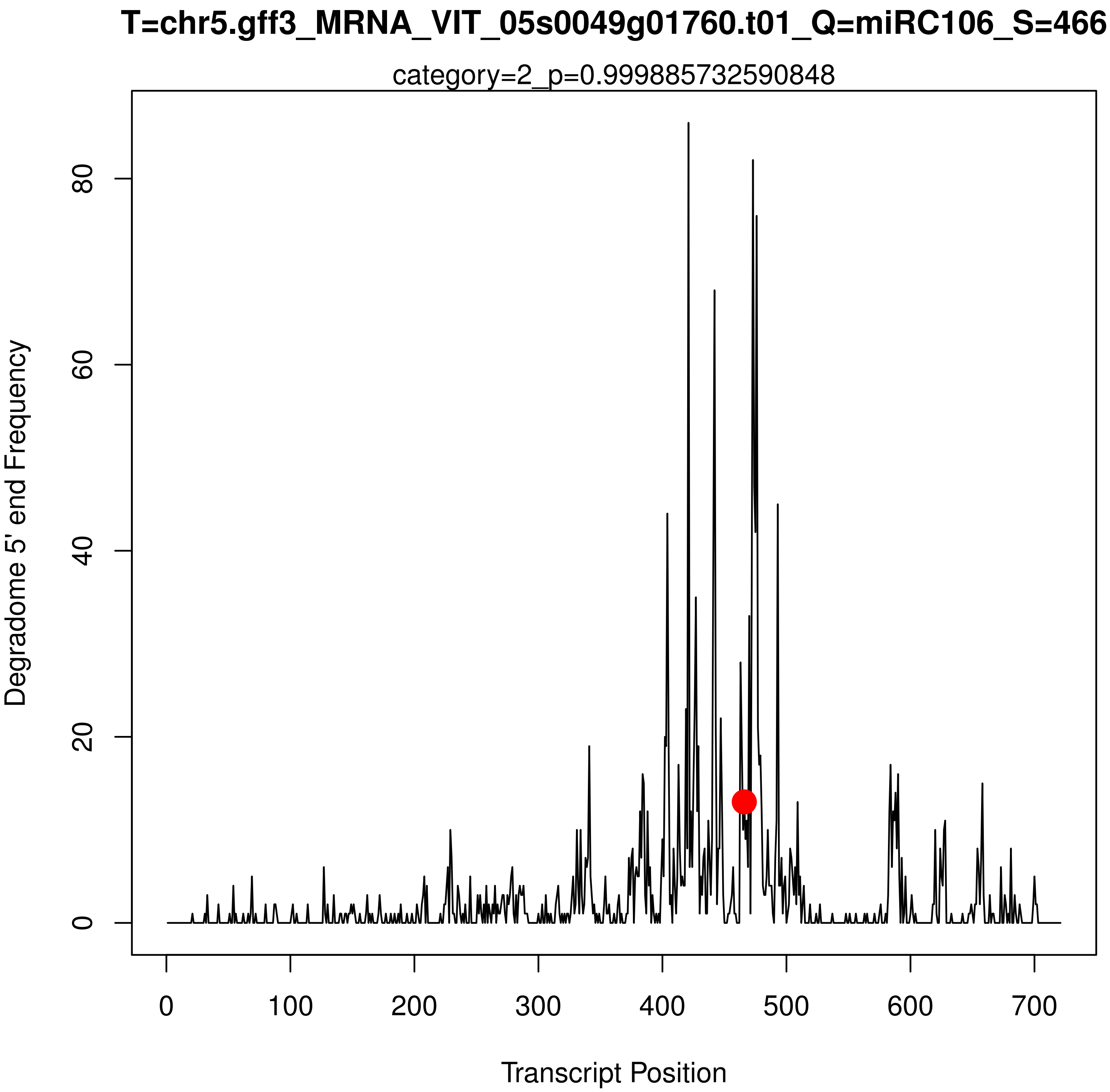
T=chr5.gff3_MRNA_VIT_05s0049g01760.t01_Q=miRC106_S=466
category=2_p=0.999885732590848
Degradome 5' end Frequency
0
20
40
60
80
0
100
200
300
400
500
600
700
Transcript Position

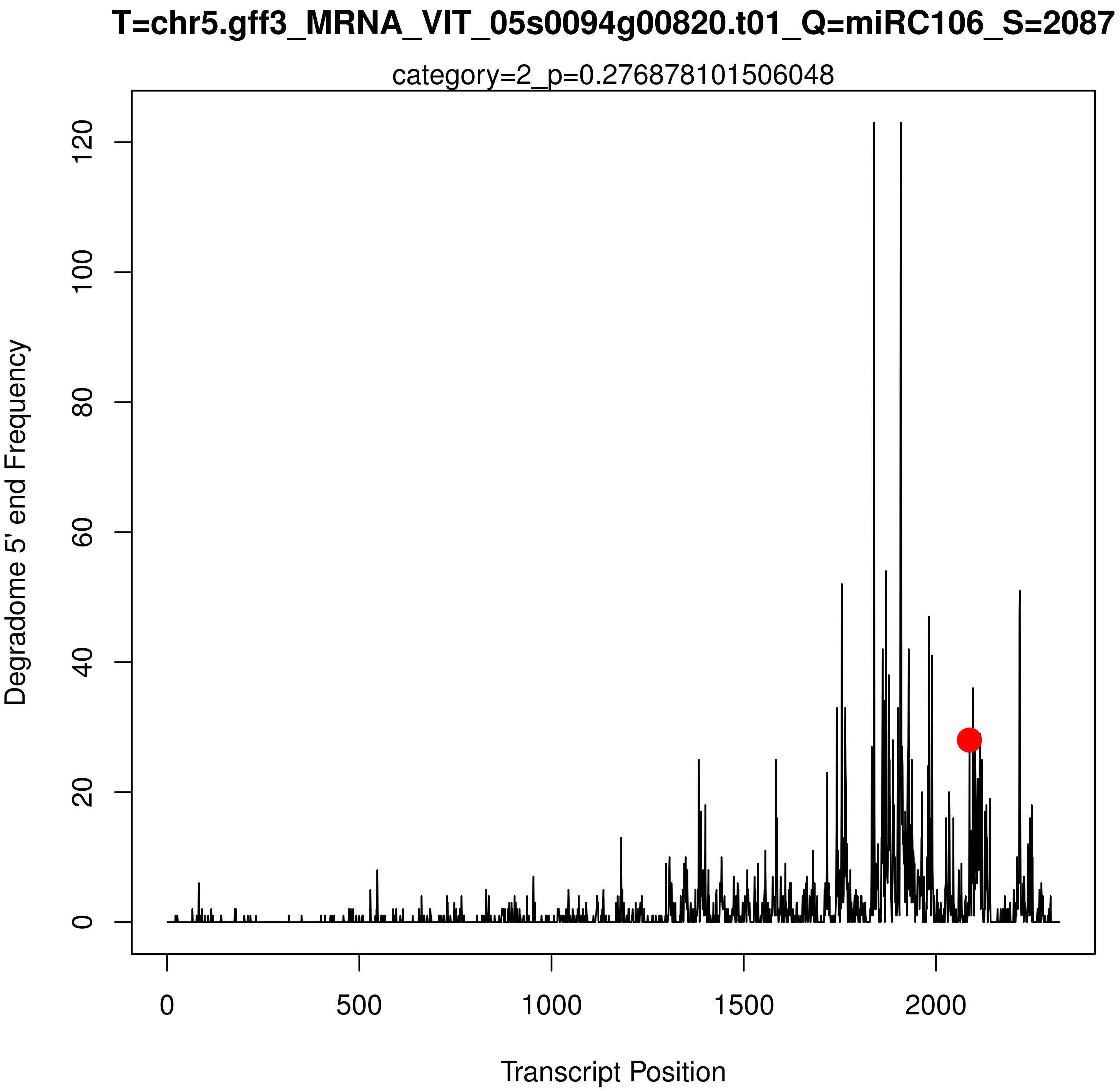
T=chr5.gff3_MRNA_VIT_05s0094g00820.t01_Q=miRC106_S=2087
category=2_p=0.276878101506048
Degradome 5' end Frequency
0
20
40
60
80
100
120
0
500
1000
1500
2000
Transcript Position

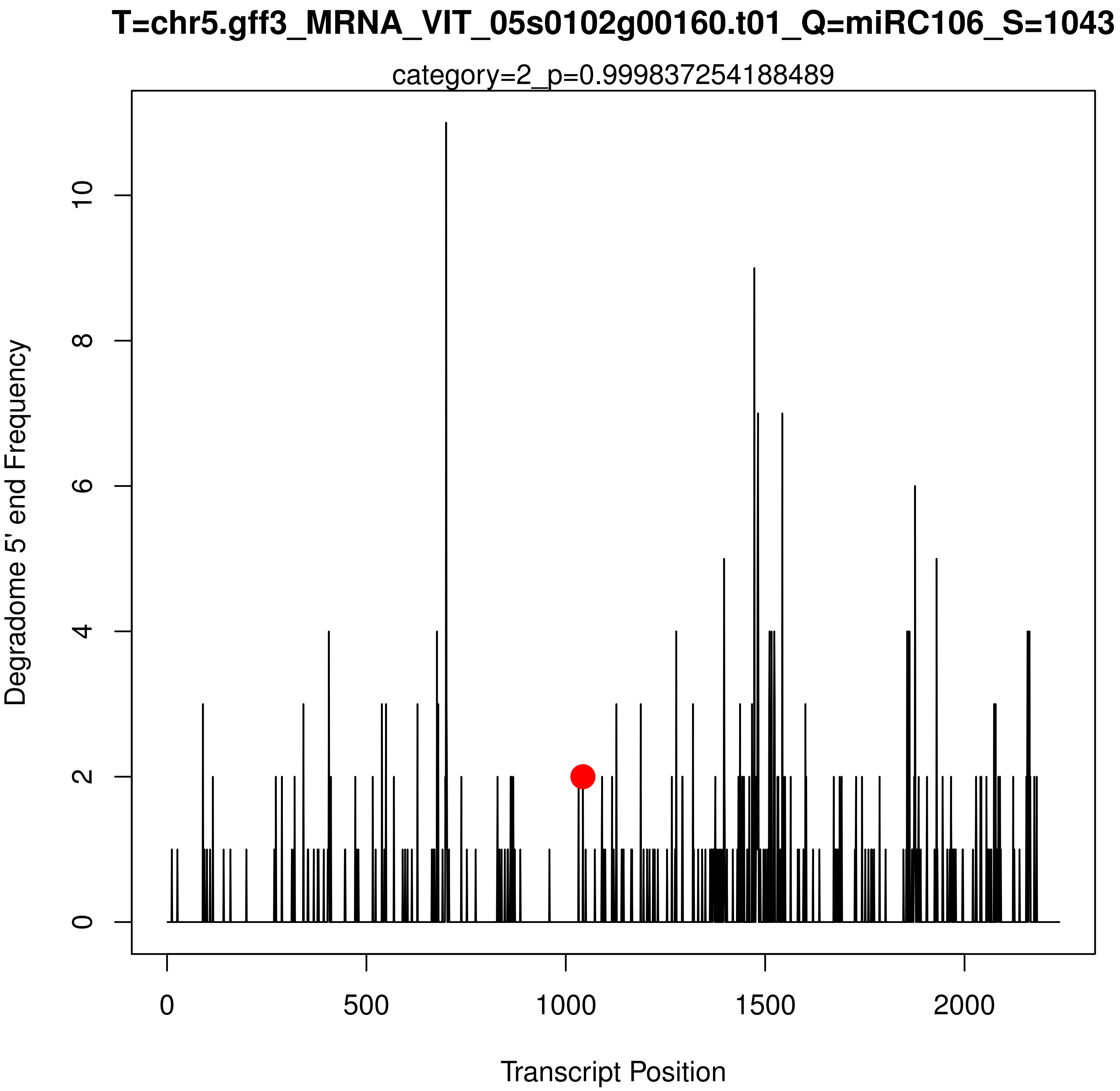

T=chr5.gff3_MRNA_VIT_05s0102g00160.t01_Q=miRC106_S=1043
category=2_p=0.999837254188489
Degradome 5' end Frequency
0
2
4
6
8
10
0
500
1000
1500
2000
Transcript Position

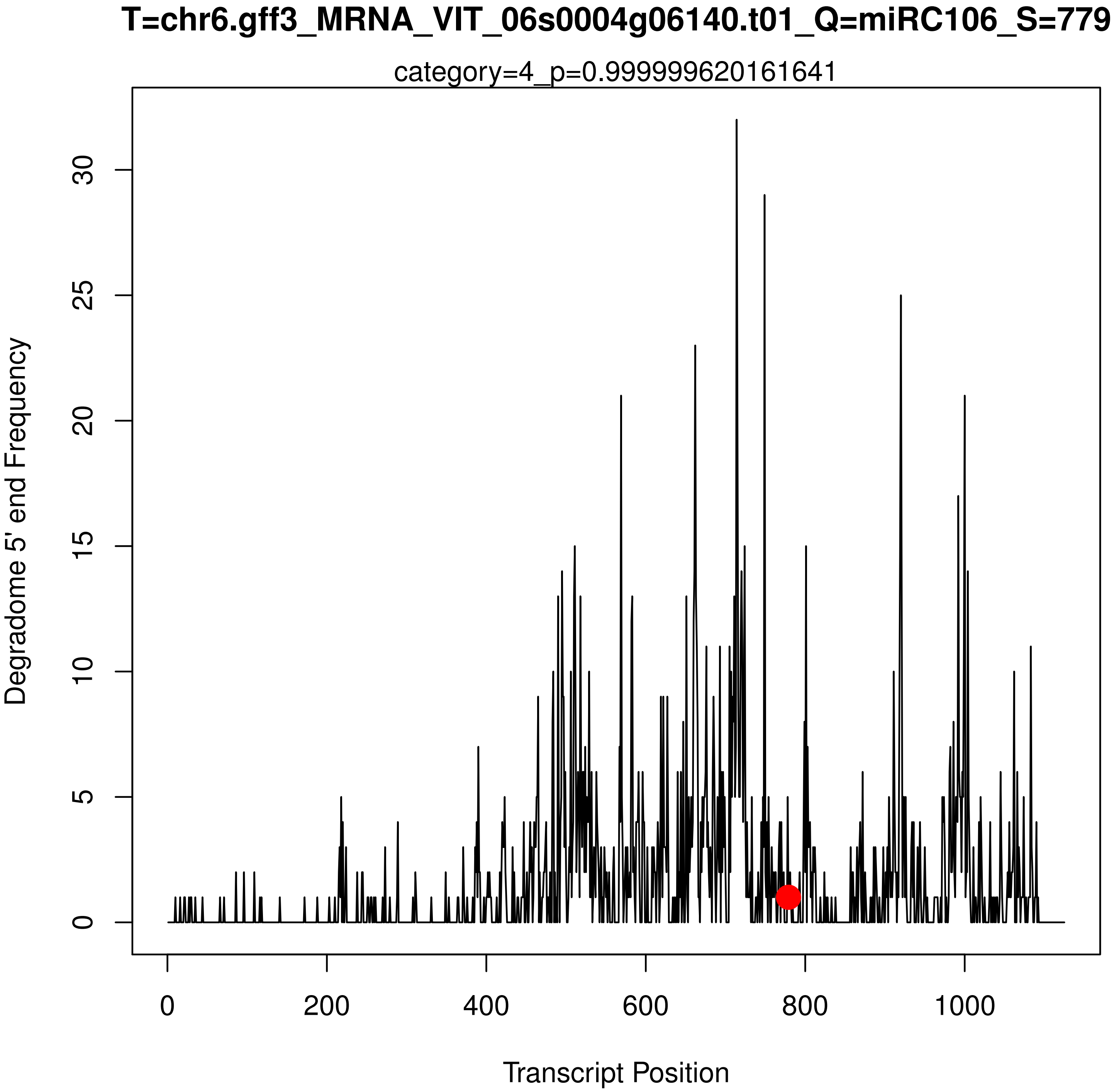
T=chr6.gff3_MRNA_VIT_06s0004g06140.t01_Q=miRC106_S=779
category=4_p=0.999999620161641
Degradome 5' end Frequency
0
5
10
15
20
25
30
0
200
400
600
800
1000
Transcript Position

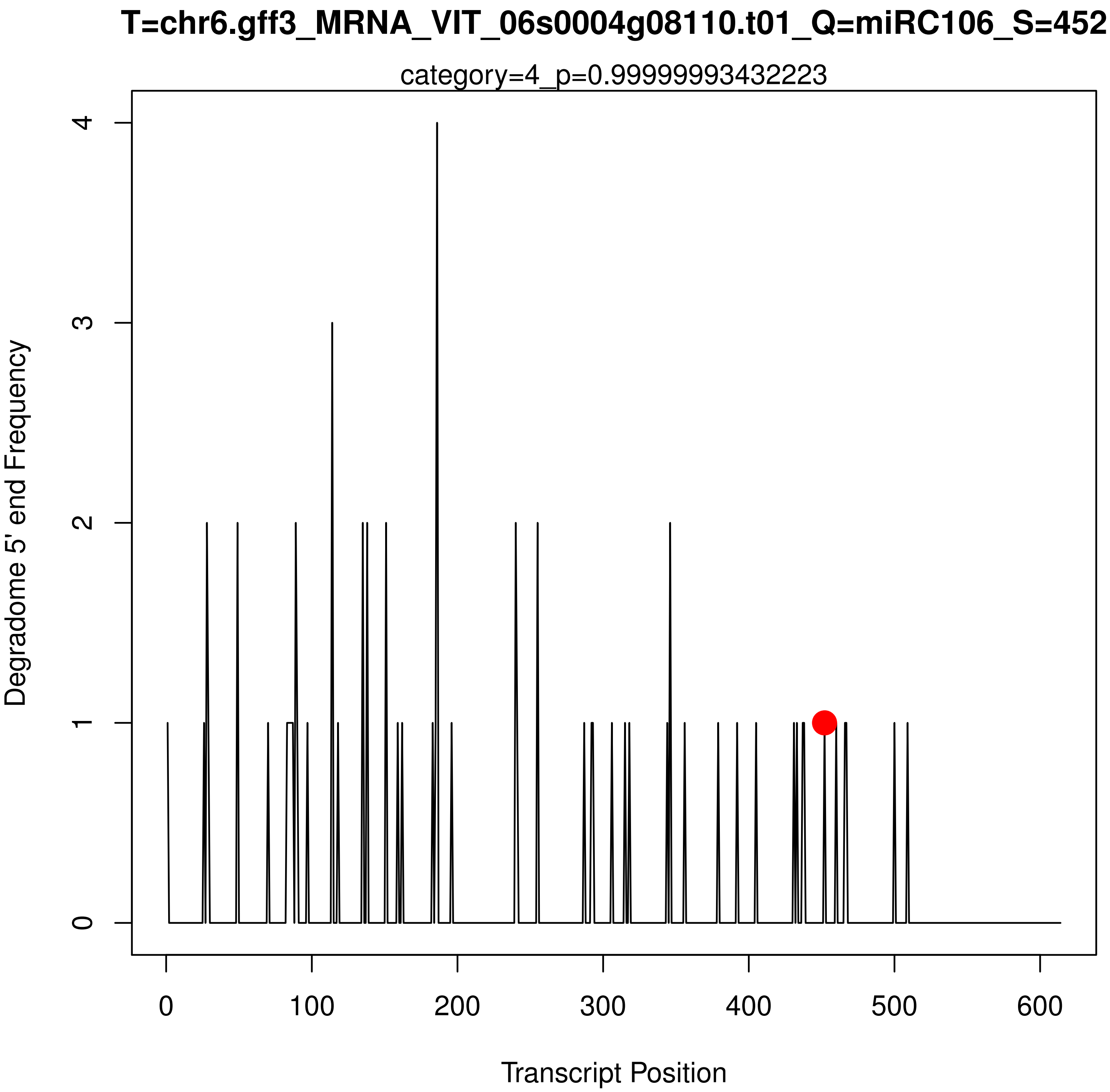
T=chr6.gff3_MRNA_VIT_06s0004g08110.t01_Q=miRC106_S=452
category=4_p=0.99999993432223
Degradome 5' end Frequency
0
1
2
3
4
0
100
200
300
400
500
600
Transcript Position

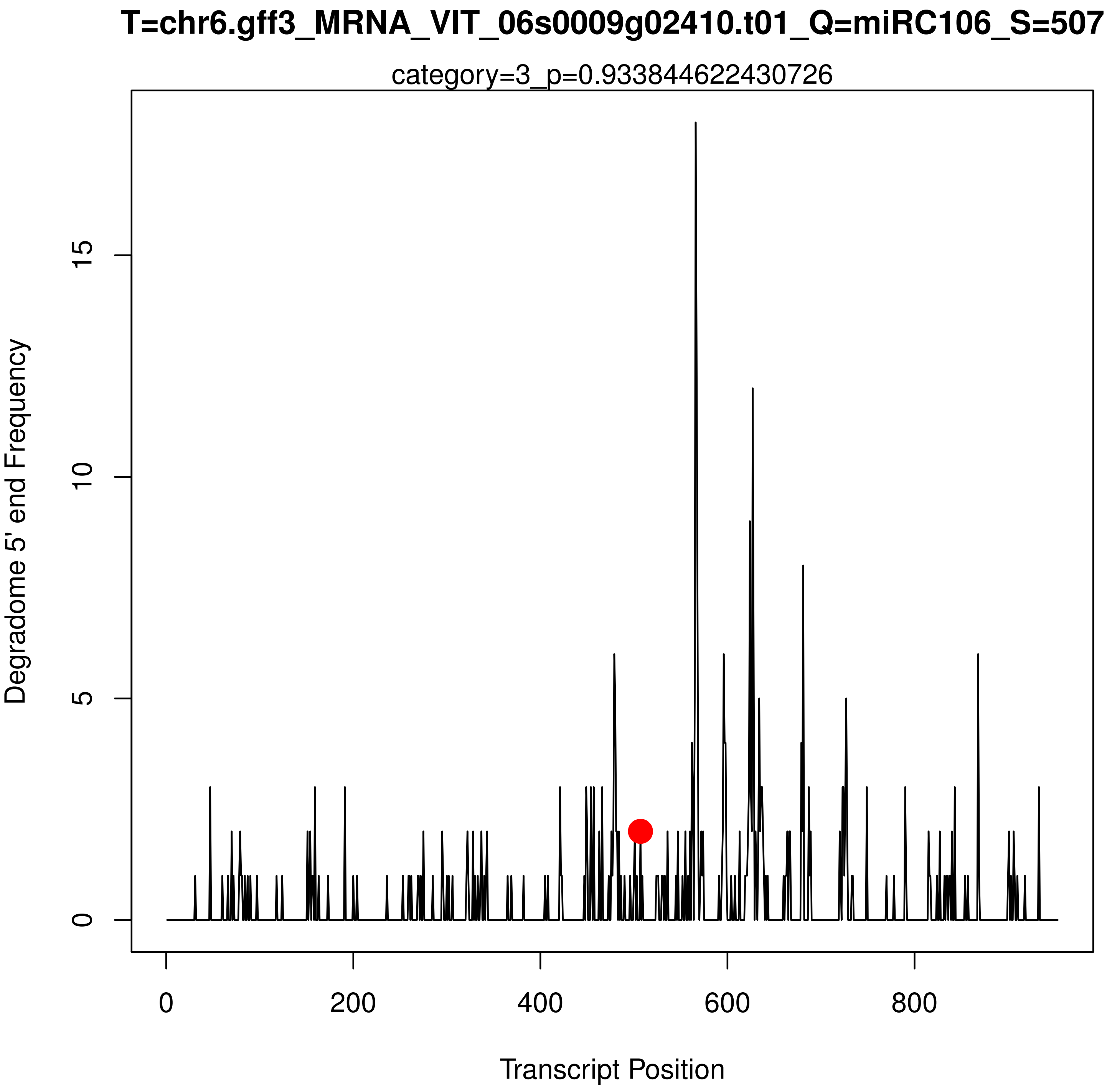
T=chr6.gff3_MRNA_VIT_06s0009g02410.t01_Q=miRC106_S=507
category=3_p=0.933844622430726
Degradome 5' end Frequency
0
5
10
15
0
200
400
600
800
Transcript Position

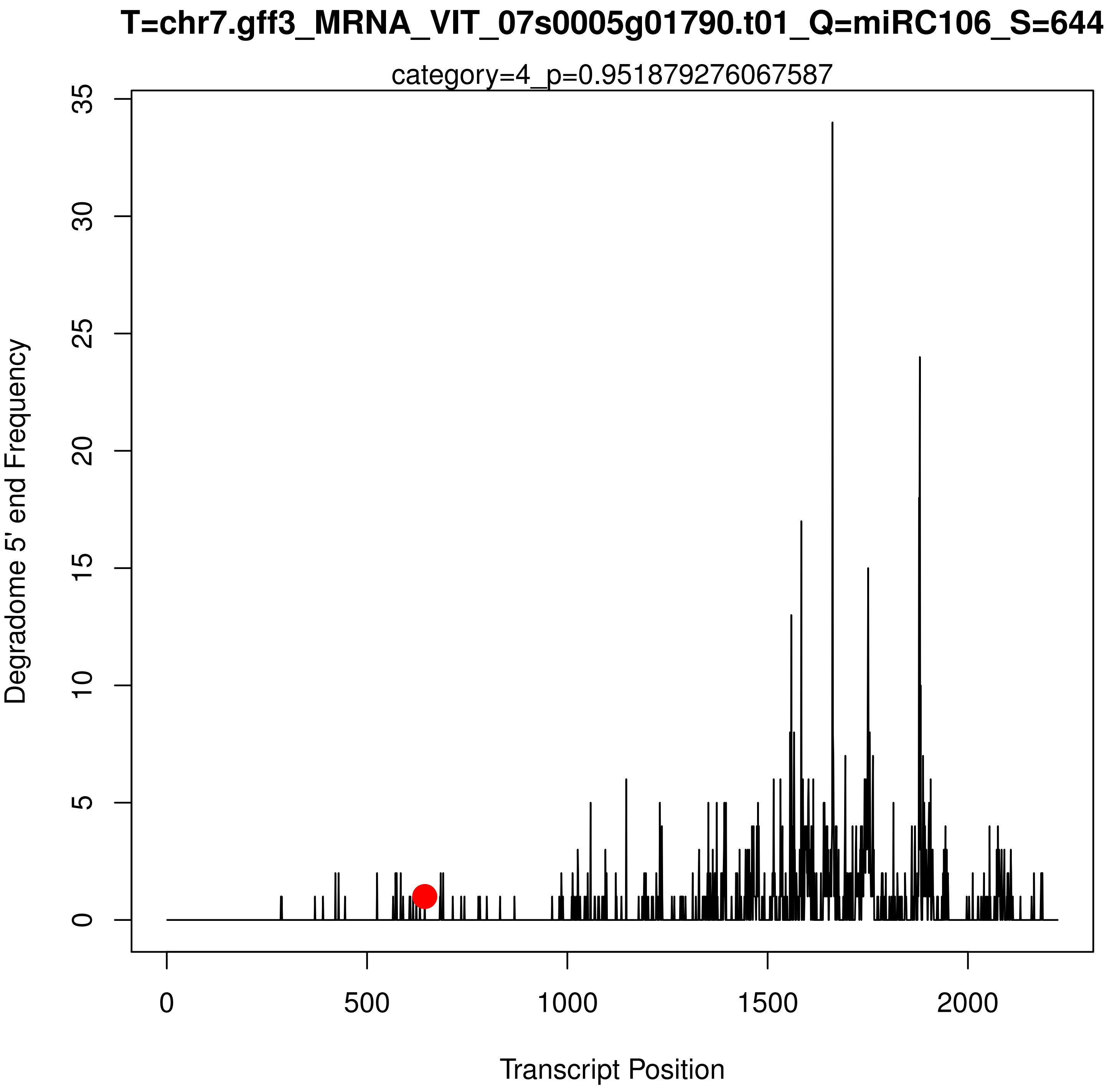

T=chr7.gff3_MRNA_VIT_07s0005g01790.t01_Q=miRC106_S=644
category=4_p=0.951879276067587
Degradome 5' end Frequency
0
5
10
15
20
25
30
35
0
500
1000
1500
2000
Transcript Position

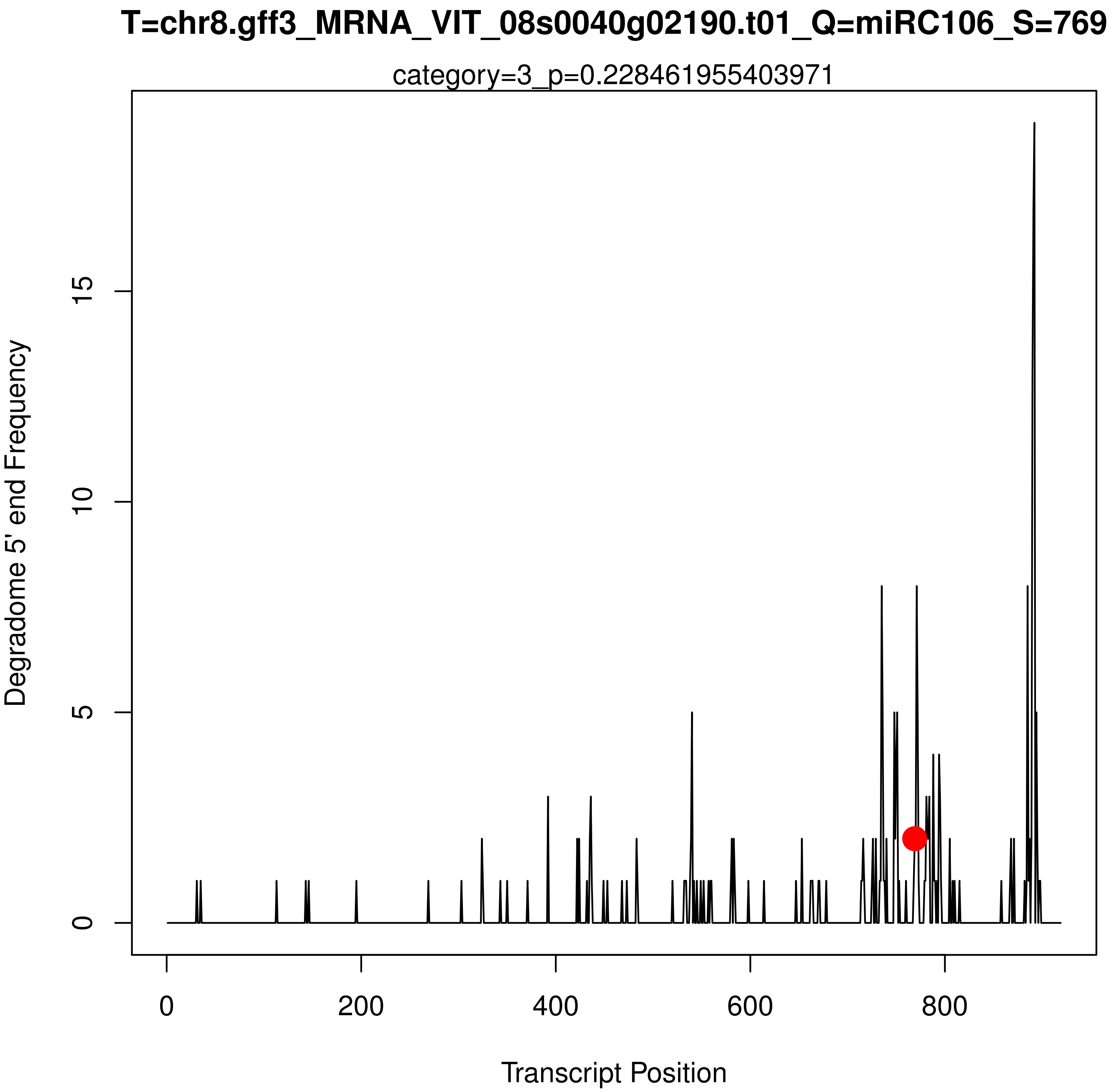
T=chr8.gff3_MRNA_VIT_08s0040g02190.t01_Q=miRC106_S=769
category=3_p=0.228461955403971
Degradome 5' end Frequency
0
5
10
15
0
200
400
600
800
Transcript Position

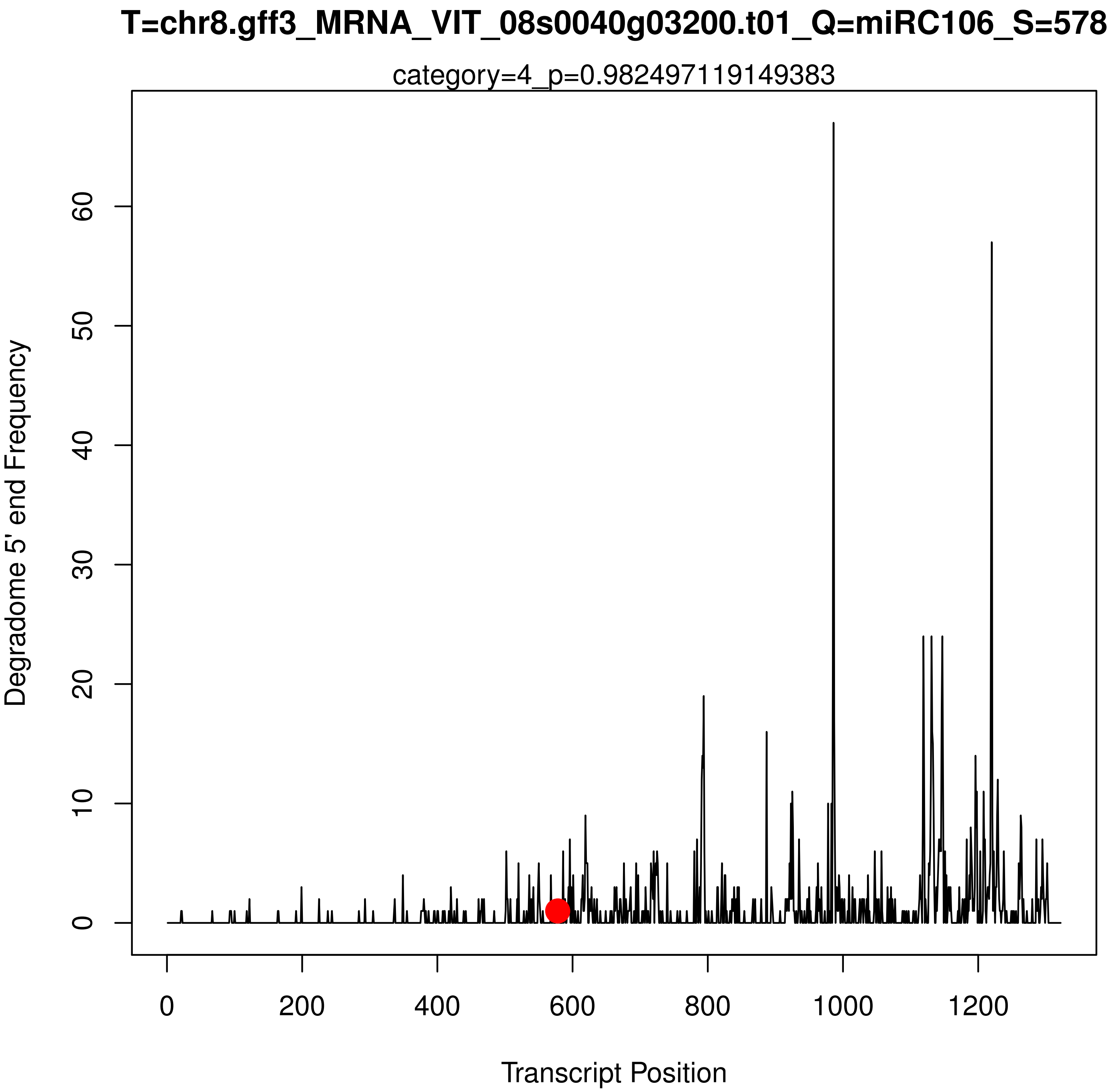
T=chr8.gff3_MRNA_VIT_08s0040g03200.t01_Q=miRC106_S=578
category=4_p=0.982497119149383
Degradome 5' end Frequency
0
10
20
30
40
50
60
0
200
400
600
800
1000
1200
Transcript Position

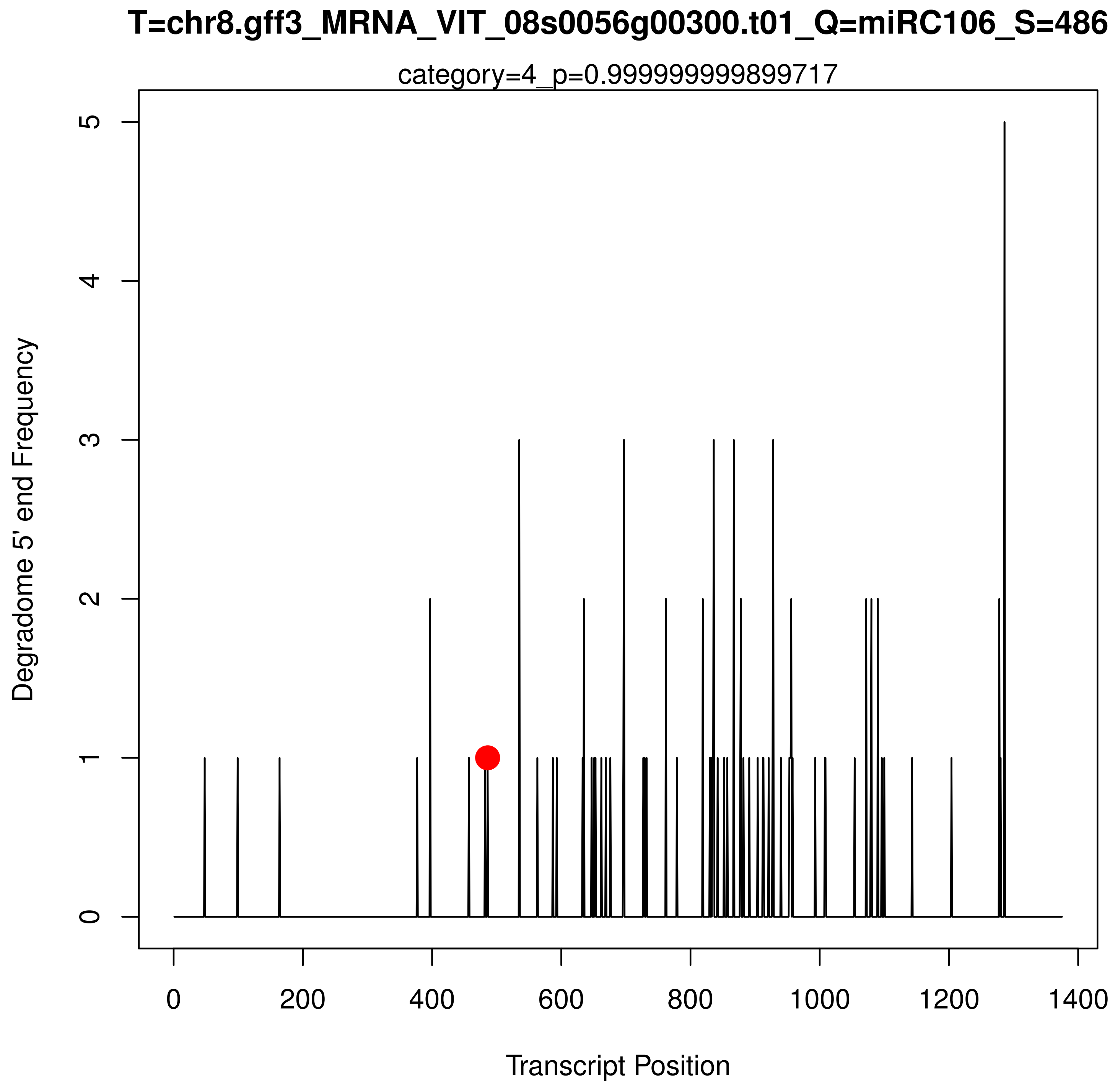
T=chr8.gff3_MRNA_VIT_08s0056g00300.t01_Q=miRC106_S=486
category=4_p=0.999999999899717
Degradome 5' end Frequency
0
1
2
3
4
5
0
200
400
600
800
1000
1200
1400
Transcript Position

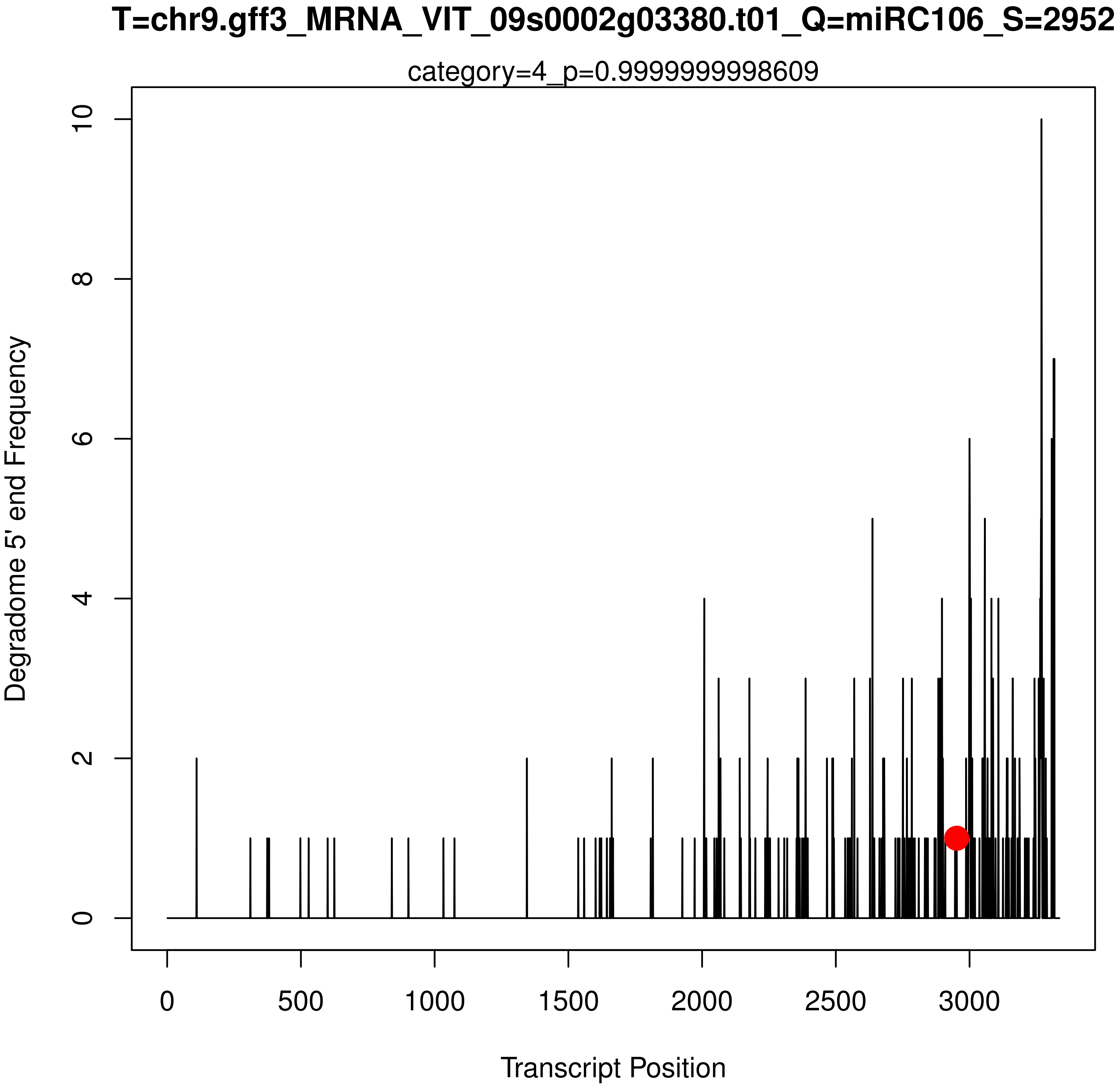

T=chr9.gff3_MRNA_VIT_09s0002g03380.t01_Q=miRC106_S=2952
category=4_p=0.9999999998609
Degradome 5' end Frequency
0
2
4
6
8
10
0
500
1000
1500
2000
2500
3000
Transcript Position

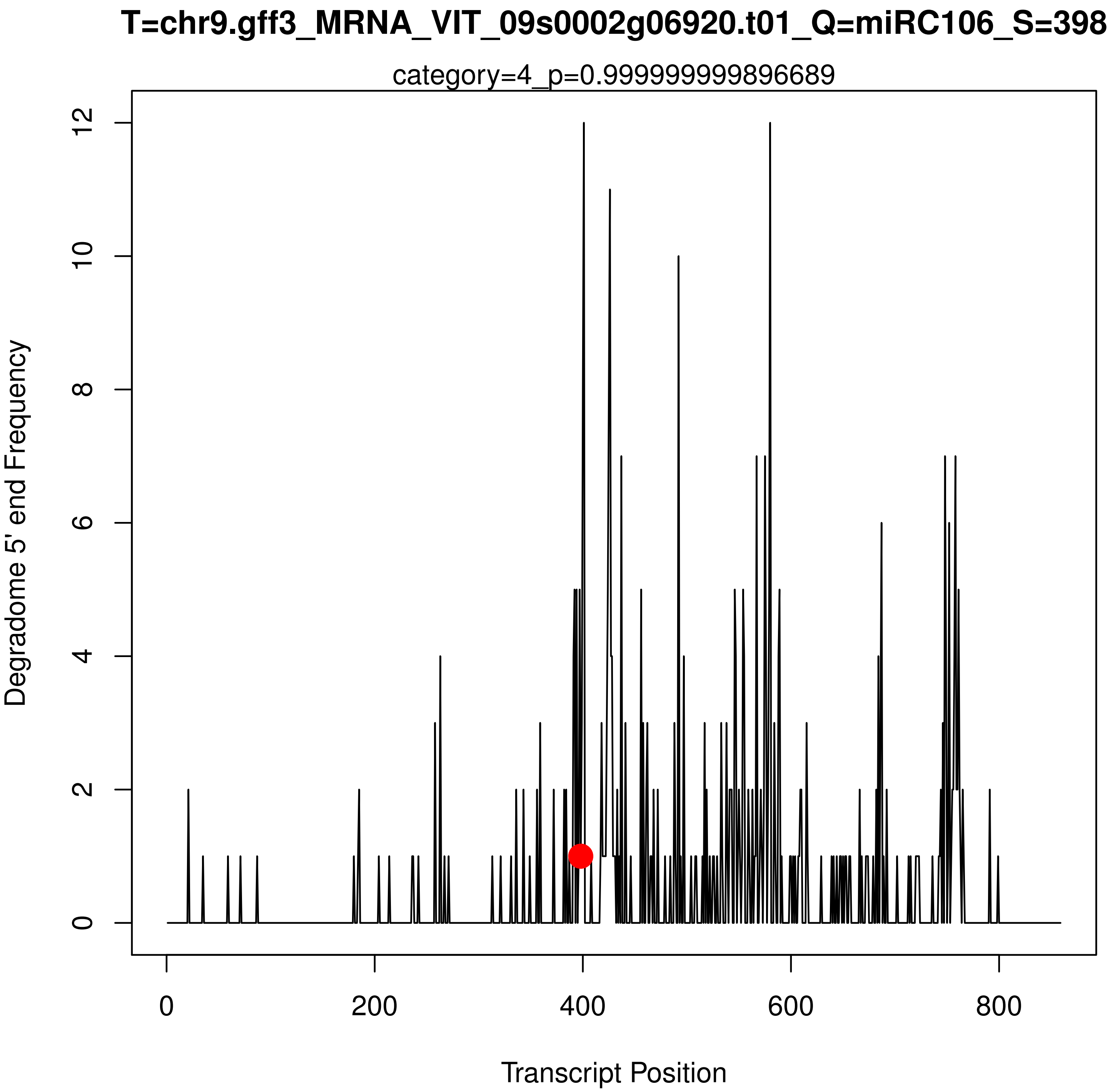

T=chr9.gff3_MRNA_VIT_09s0002g06920.t01_Q=miRC106_S=398
category=4_p=0.999999999896689
Degradome 5' end Frequency
Transcript Position
0
2
4
6
8
10
12
0
200
400
600
800

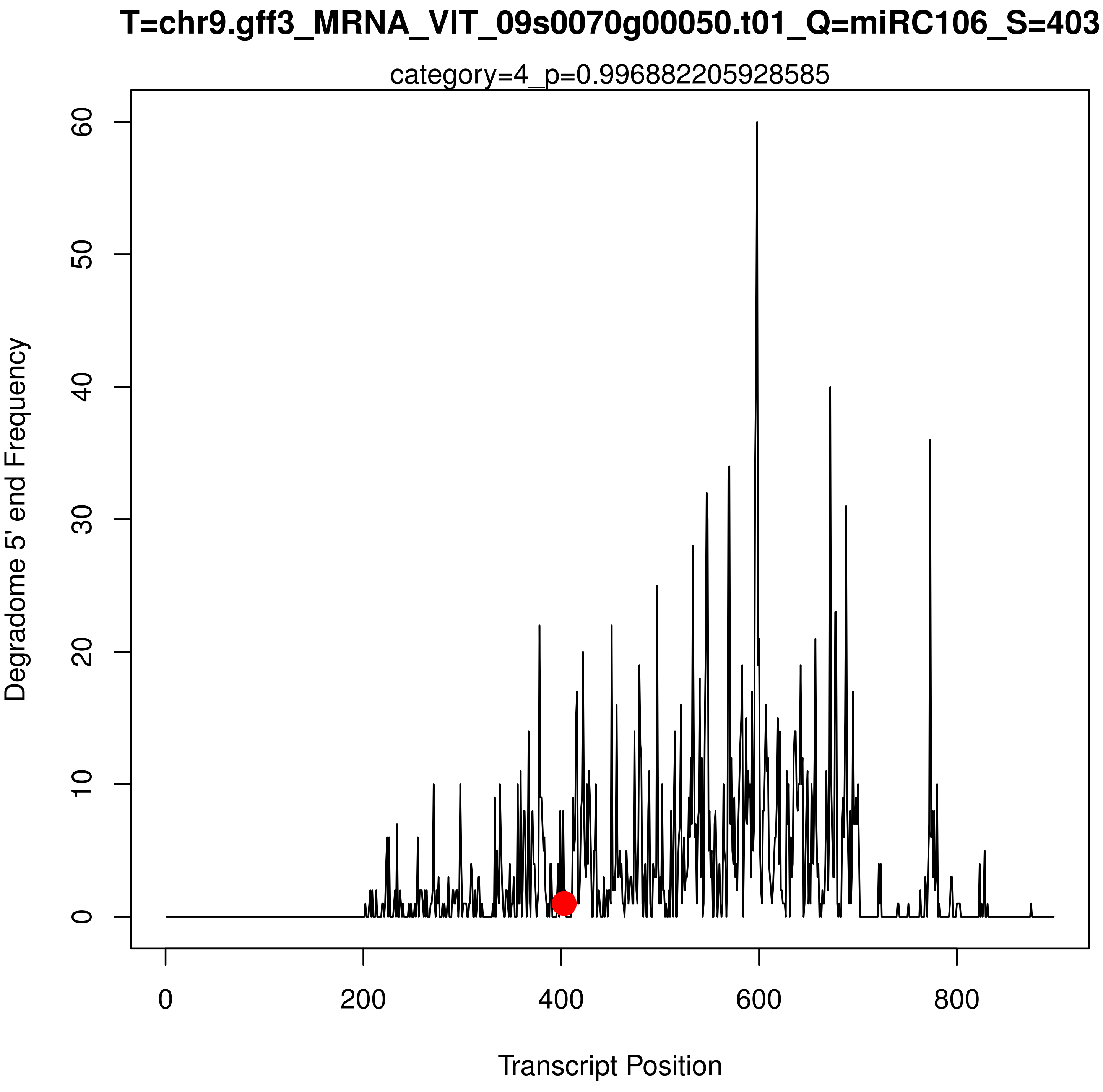
T=chr9.gff3_MRNA_VIT_09s0070g00050.t01_Q=miRC106_S=403
category=4_p=0.996882205928585
Degradome 5' end Frequency
0
10
20
30
40
50
60
0
200
400
600
800
Transcript Position

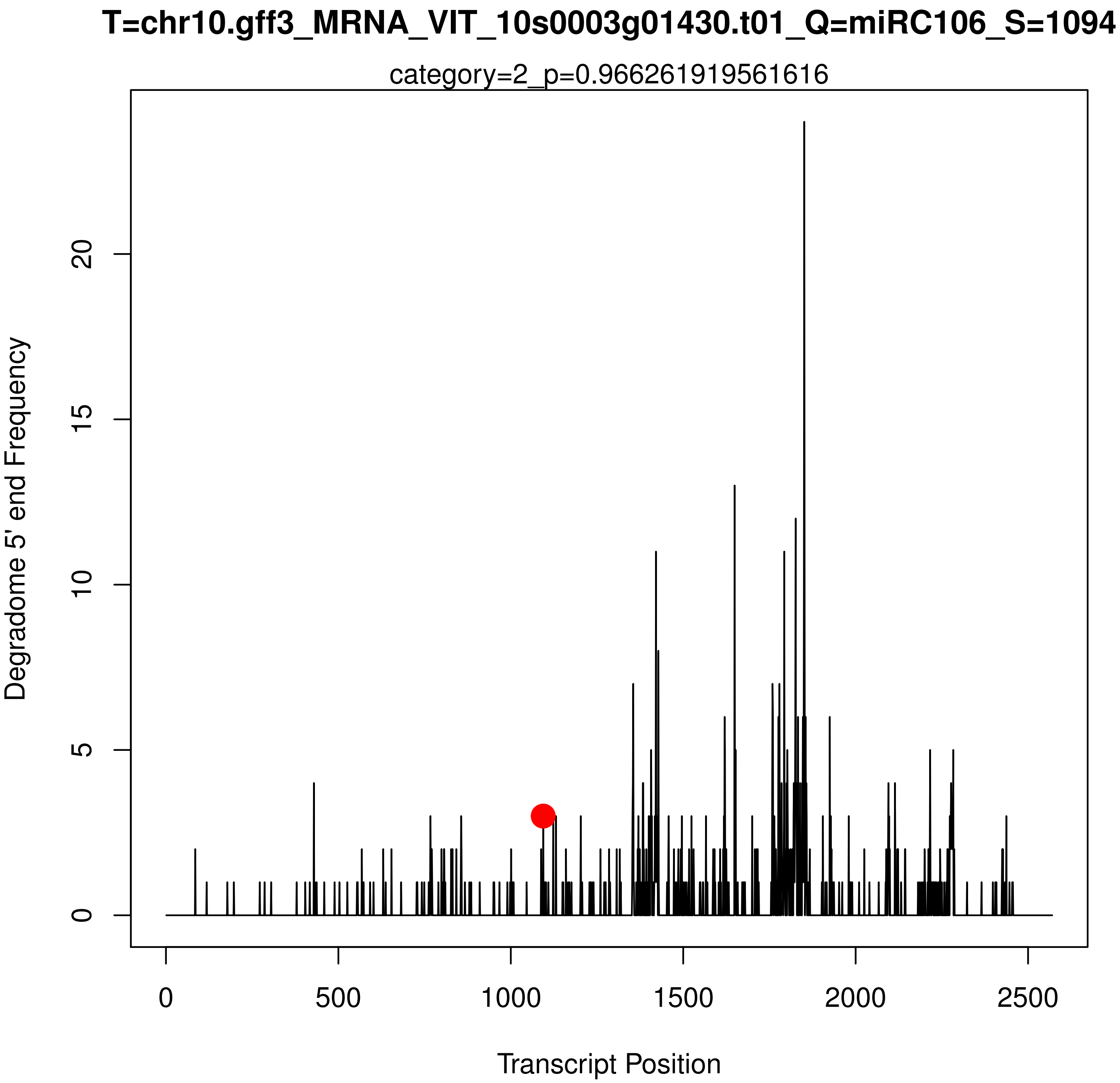

T=chr10.gff3_MRNA_VIT_10s0003g01430.t01_Q=miRC106_S=1094
category=2_p=0.966261919561616
Degradome 5' end Frequency
0
5
10
15
20
0
500
1000
1500
2000
2500
Transcript Position

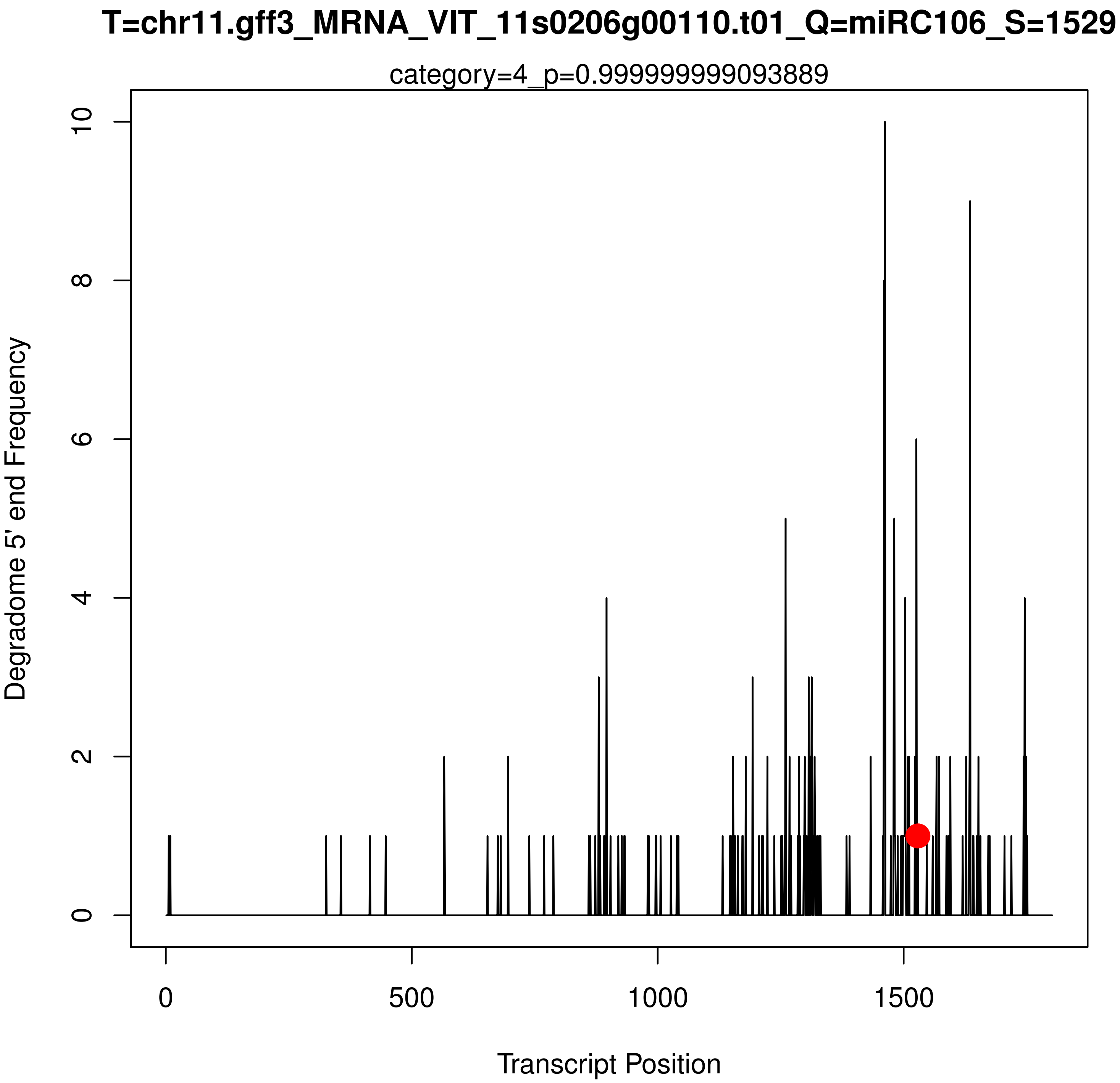

T=chr11.gff3_MRNA_VIT_11s0206g00110.t01_Q=miRC106_S=1529
category=4_p=0.999999999093889
Degradome 5' end Frequency
Transcript Position
0
2
4
6
8
10
0
500
1000
1500

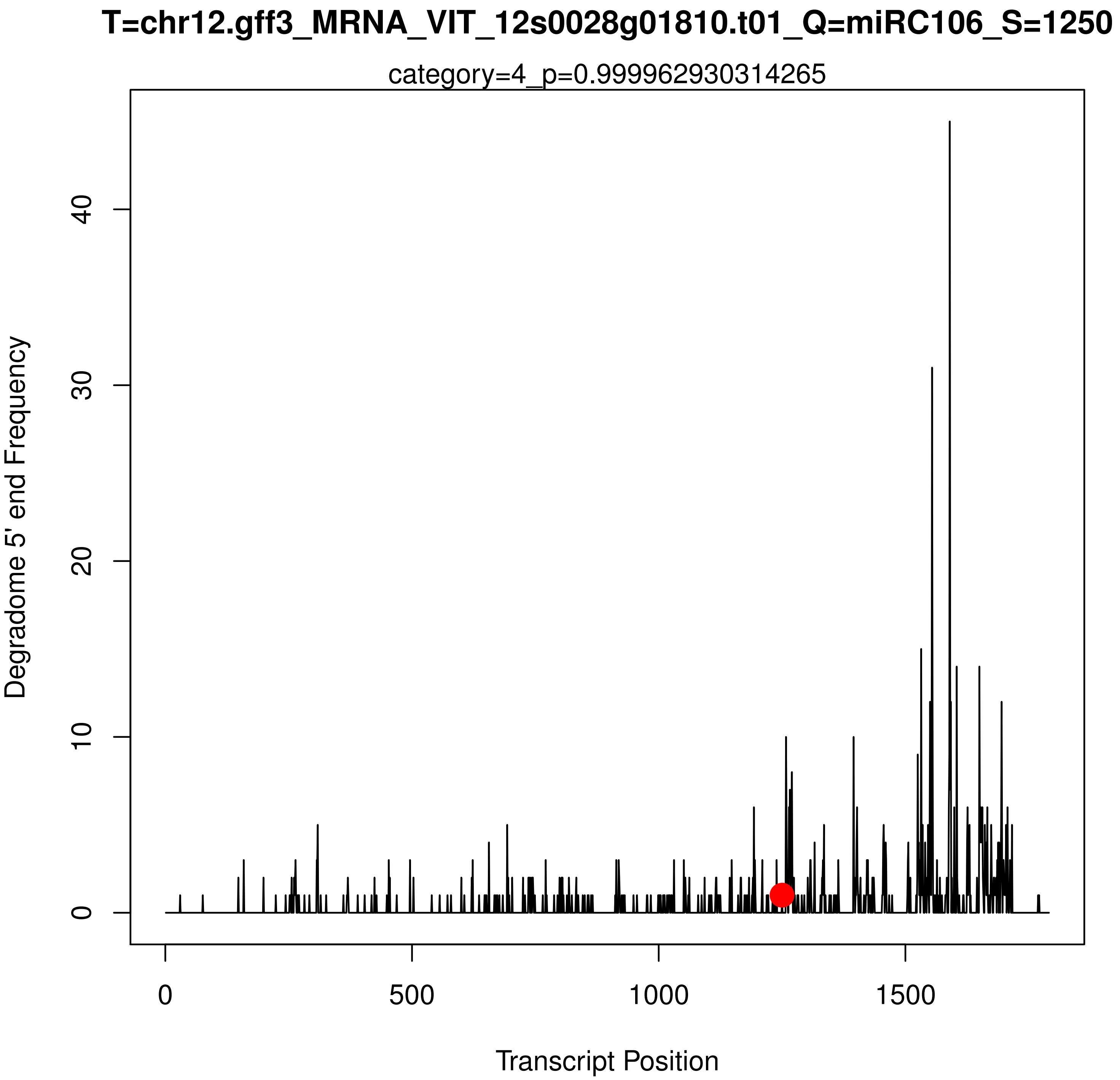
T=chr12.gff3_MRNA_VIT_12s0028g01810.t01_Q=miRC106_S=1250
category=4_p=0.999962930314265
Degradome 5' end Frequency
0
10
20
30
40
0
500
1000
1500
Transcript Position

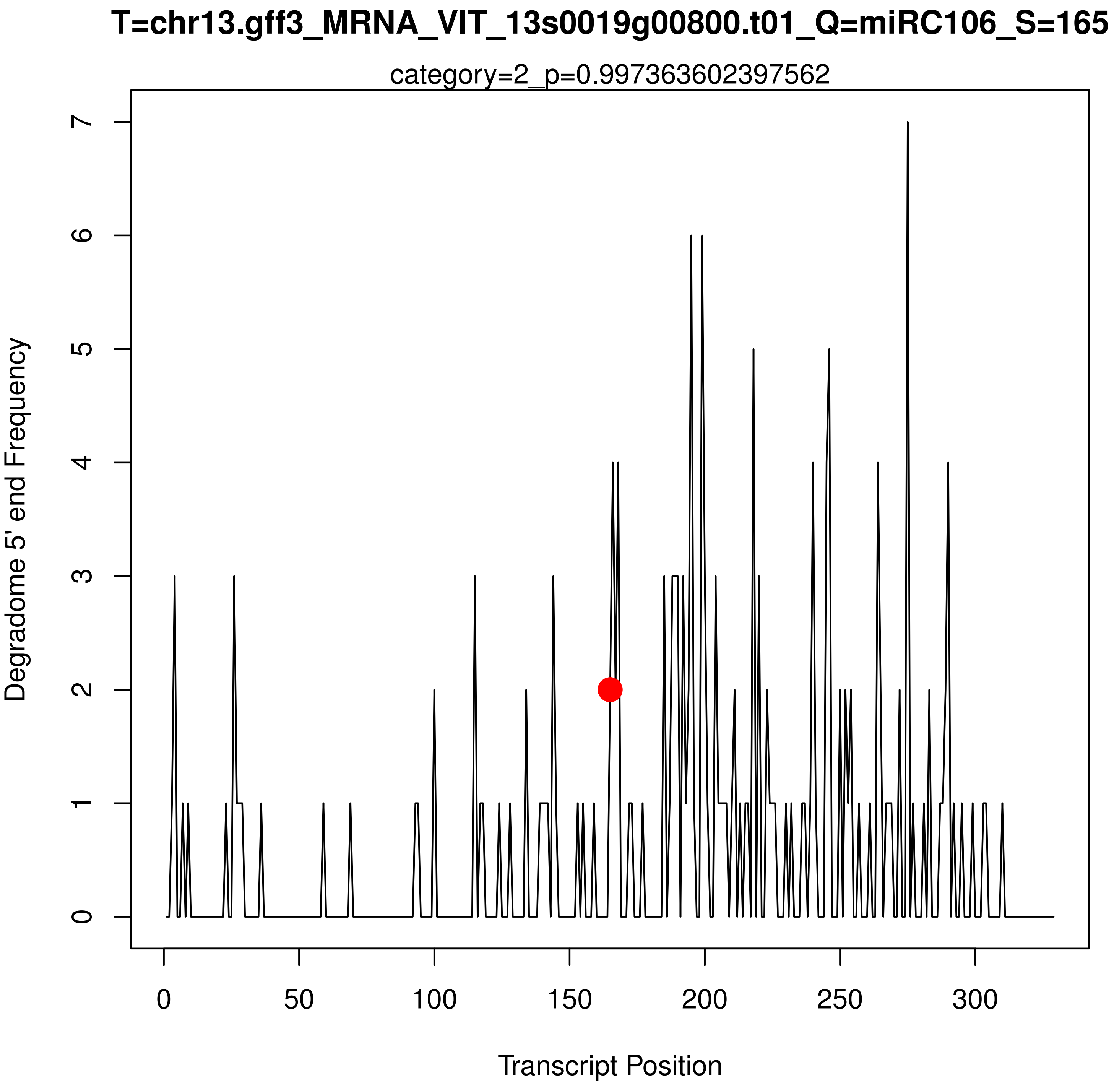

T=chr13.gff3_MRNA_VIT_13s0019g00800.t01_Q=miRC106_S=165
category=2_p=0.997363602397562
Degradome 5' end Frequency
Transcript Position
0
1
2
3
4
5
6
7
0
50
100
150
200
250
300

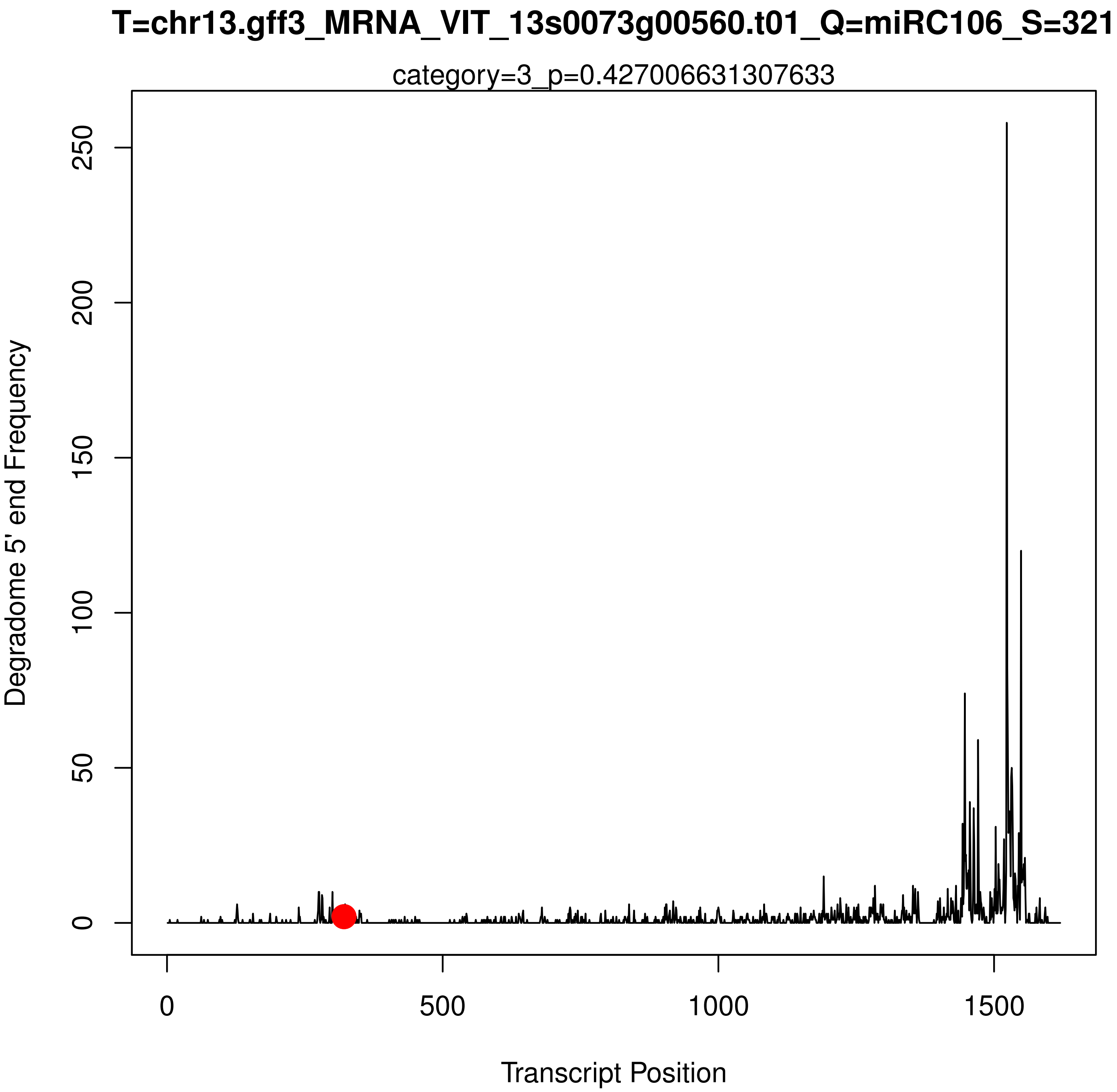
T=chr13.gff3_MRNA_VIT_13s0073g00560.t01_Q=miRC106_S=321
category=3_p=0.427006631307633
Degradome 5' end Frequency
0
50
100
150
200
250
0
500
1000
1500
Transcript Position

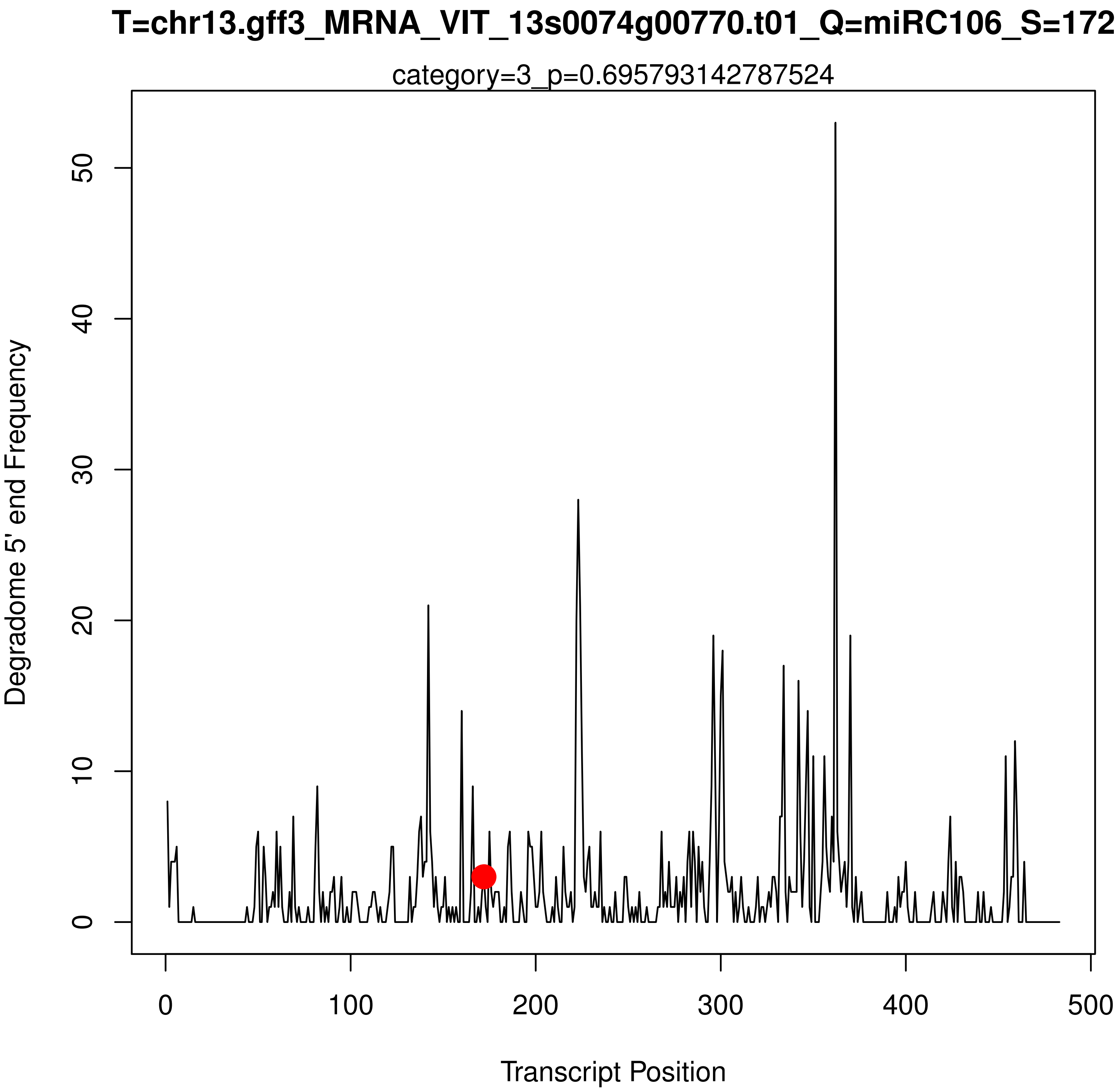
T=chr13.gff3_MRNA_VIT_13s0074g00770.t01_Q=miRC106_S=172
category=3_p=0.695793142787524
Degradome 5' end Frequency
0
10
20
30
40
50
0
100
200
300
400
500
Transcript Position

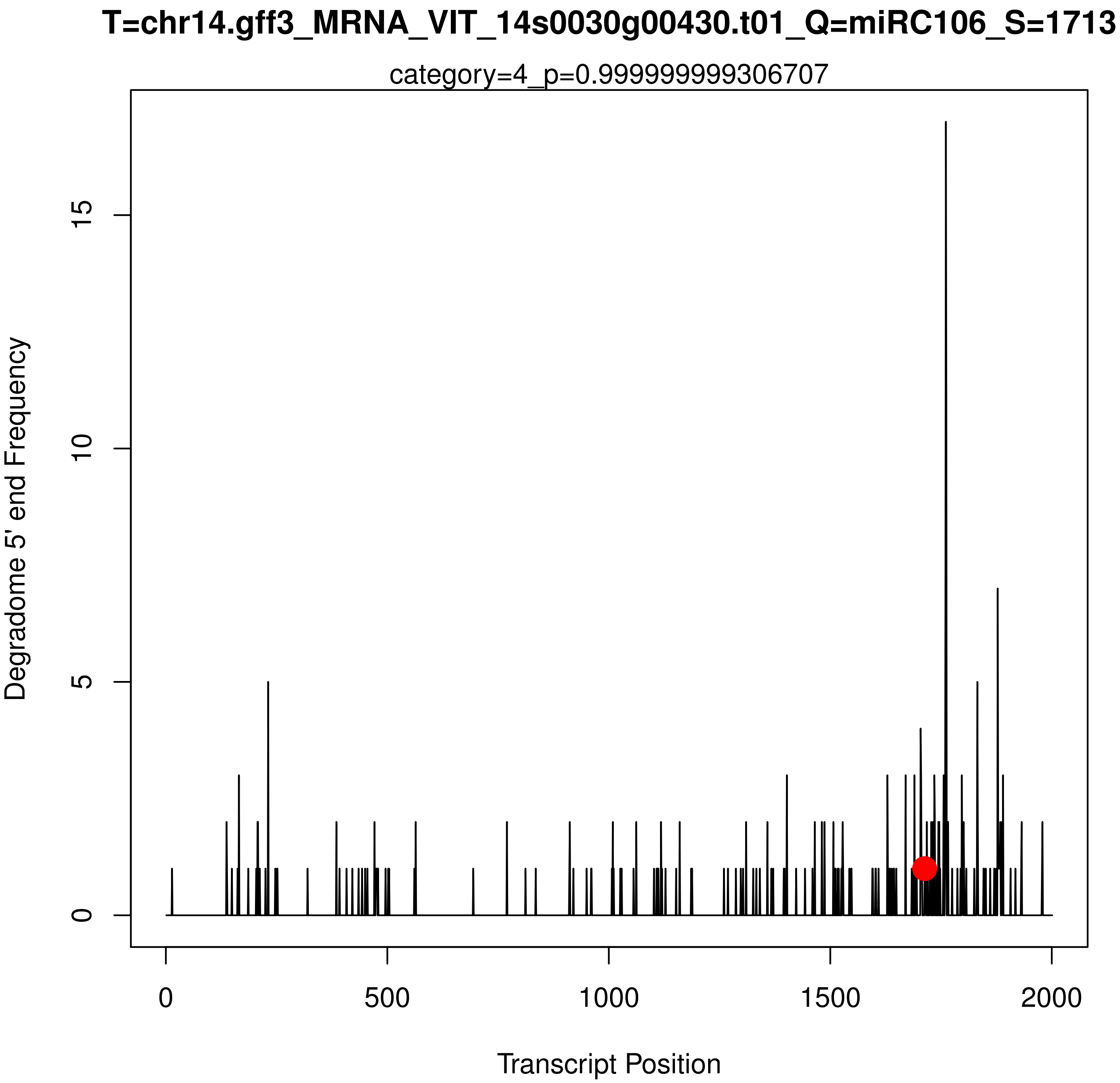

T=chr14.gff3_MRNA_VIT_14s0030g00430.t01_Q=miRC106_S=1713
category=4_p=0.999999999306707
Degradome 5' end Frequency
0
5
10
15
0
500
1000
1500
2000
Transcript Position

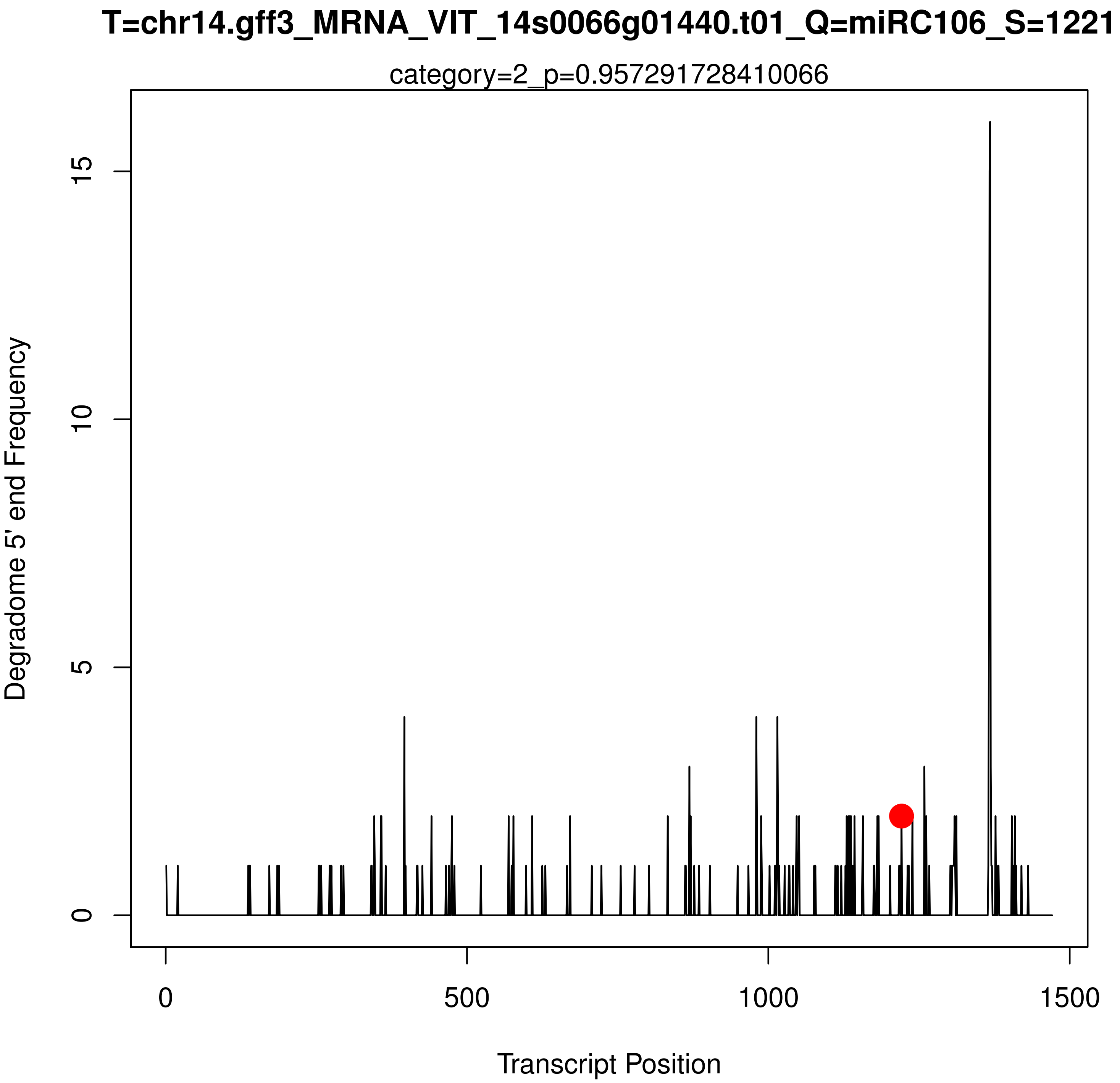
T=chr14.gff3_MRNA_VIT_14s0066g01440.t01_Q=miRC106_S=1221
category=2_p=0.957291728410066
Degradome 5' end Frequency
0
5
10
15
0
500
1000
1500
Transcript Position

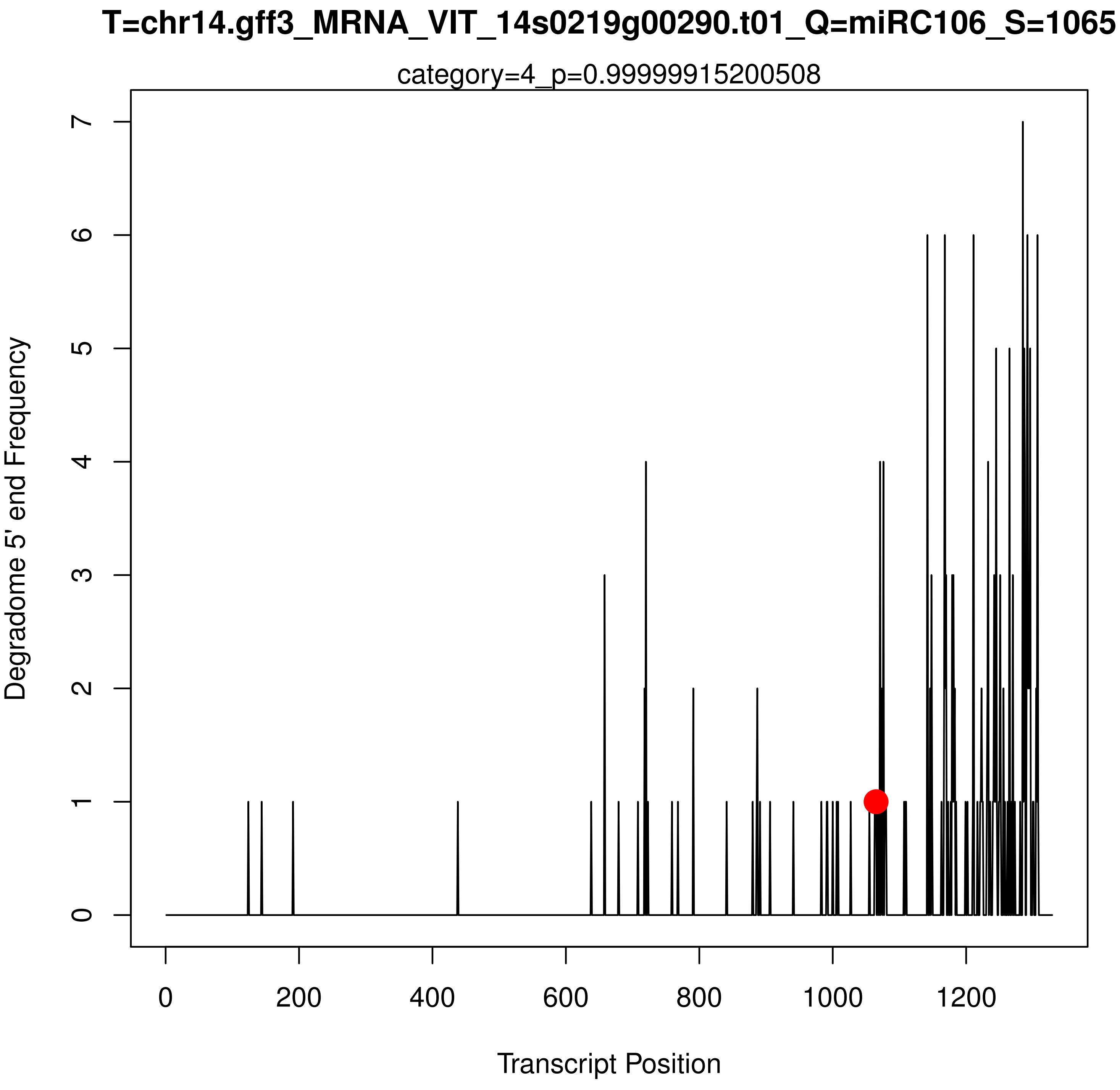
T=chr14.gff3_MRNA_VIT_14s0219g00290.t01_Q=miRC106_S=1065
category=4_p=0.99999915200508
Degradome 5' end Frequency
Transcript Position
0
1
2
3
4
5
6
7
0
200
400
600
800
1000
1200

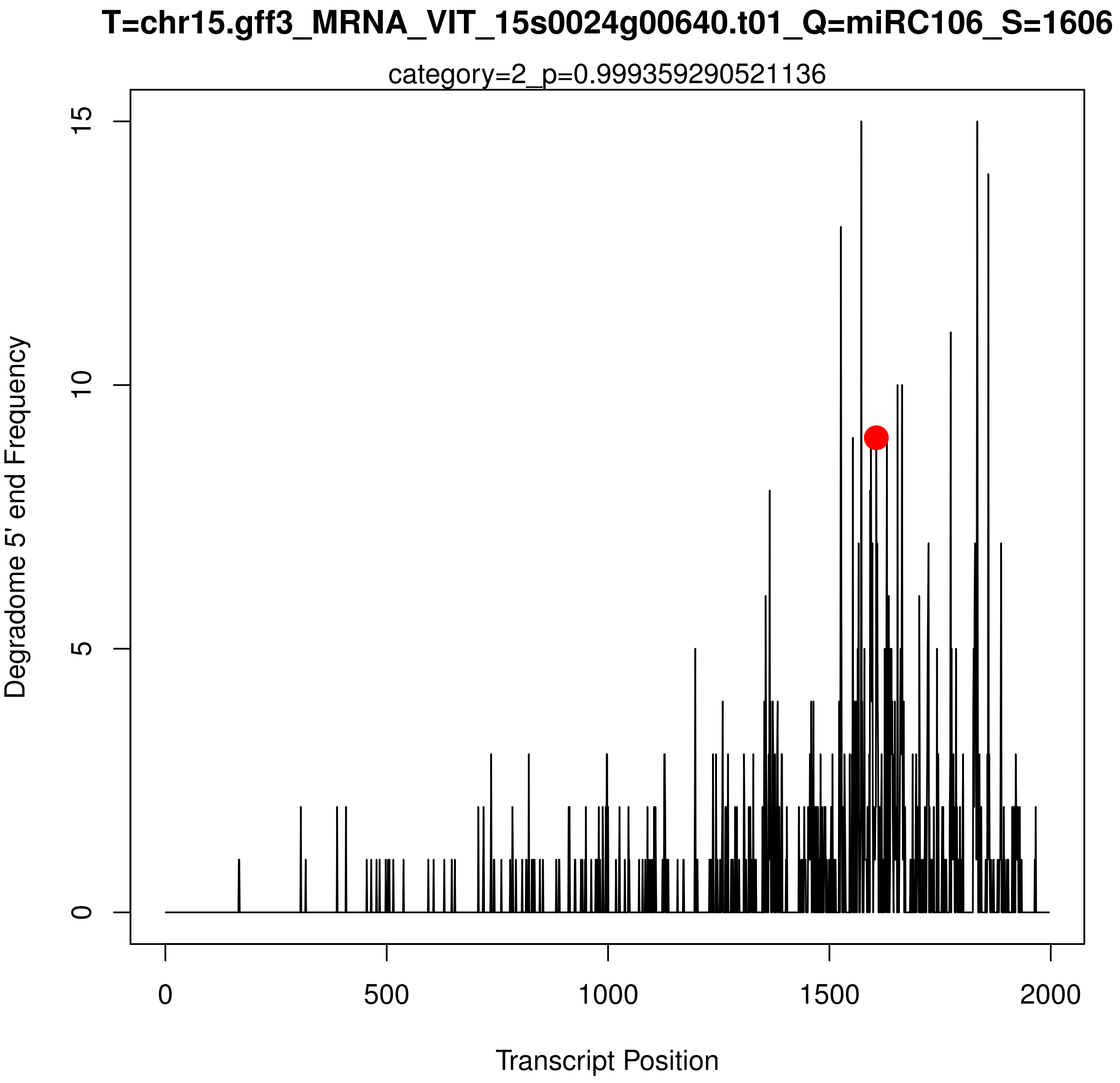

T=chr15.gff3_MRNA_VIT_15s0024g00640.t01_Q=miRC106_S=1606
category=2_p=0.999359290521136
Degradome 5' end Frequency
Transcript Position
0
5
10
15
0
500
1000
1500
2000

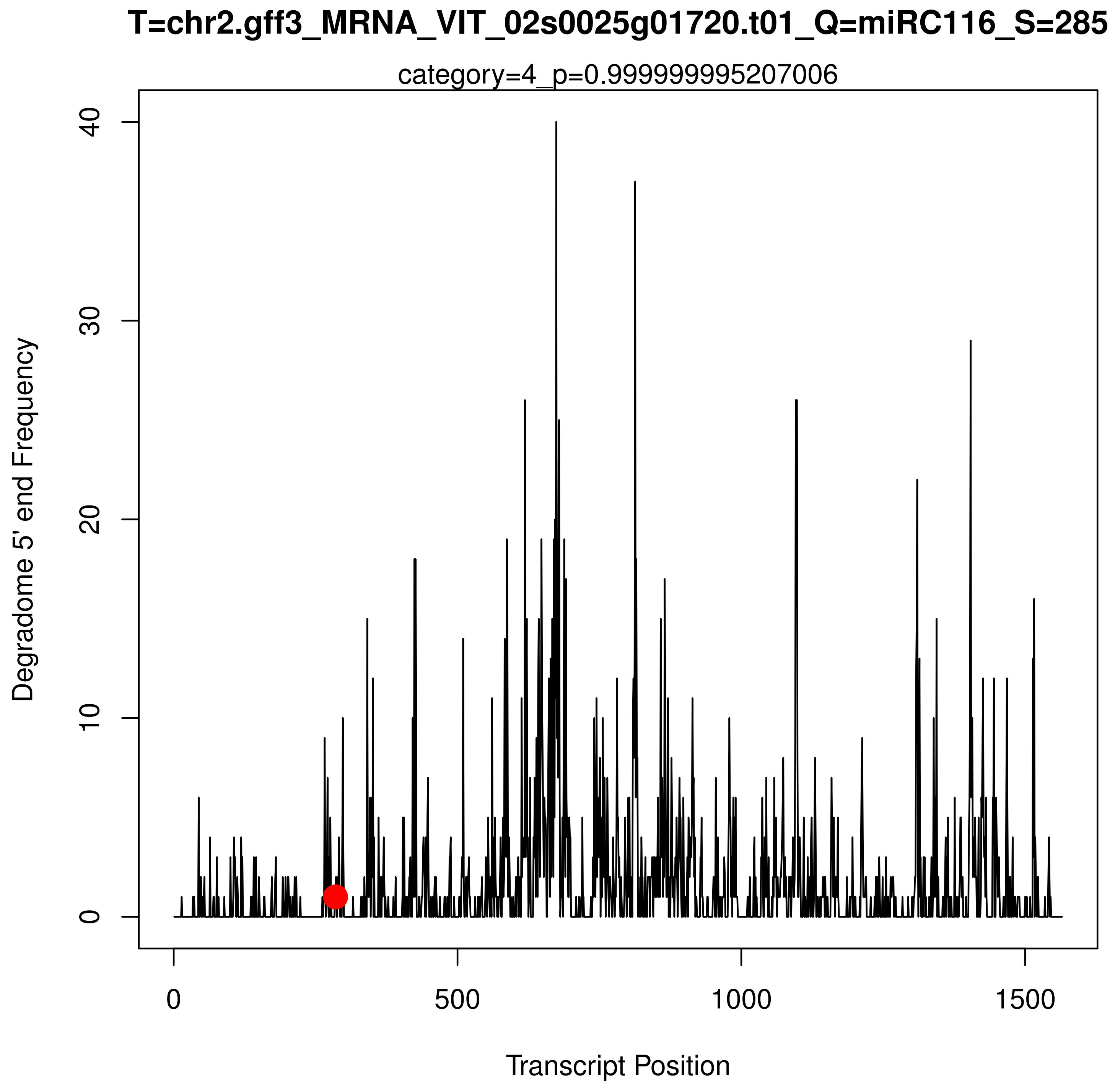
T=chr2.gff3_MRNA_VIT_02s0025g01720.t01_Q=miRC116_S=285
category=4_p=0.999999995207006
Degradome 5' end Frequency
0
10
20
30
40
0
500
1000
1500
Transcript Position

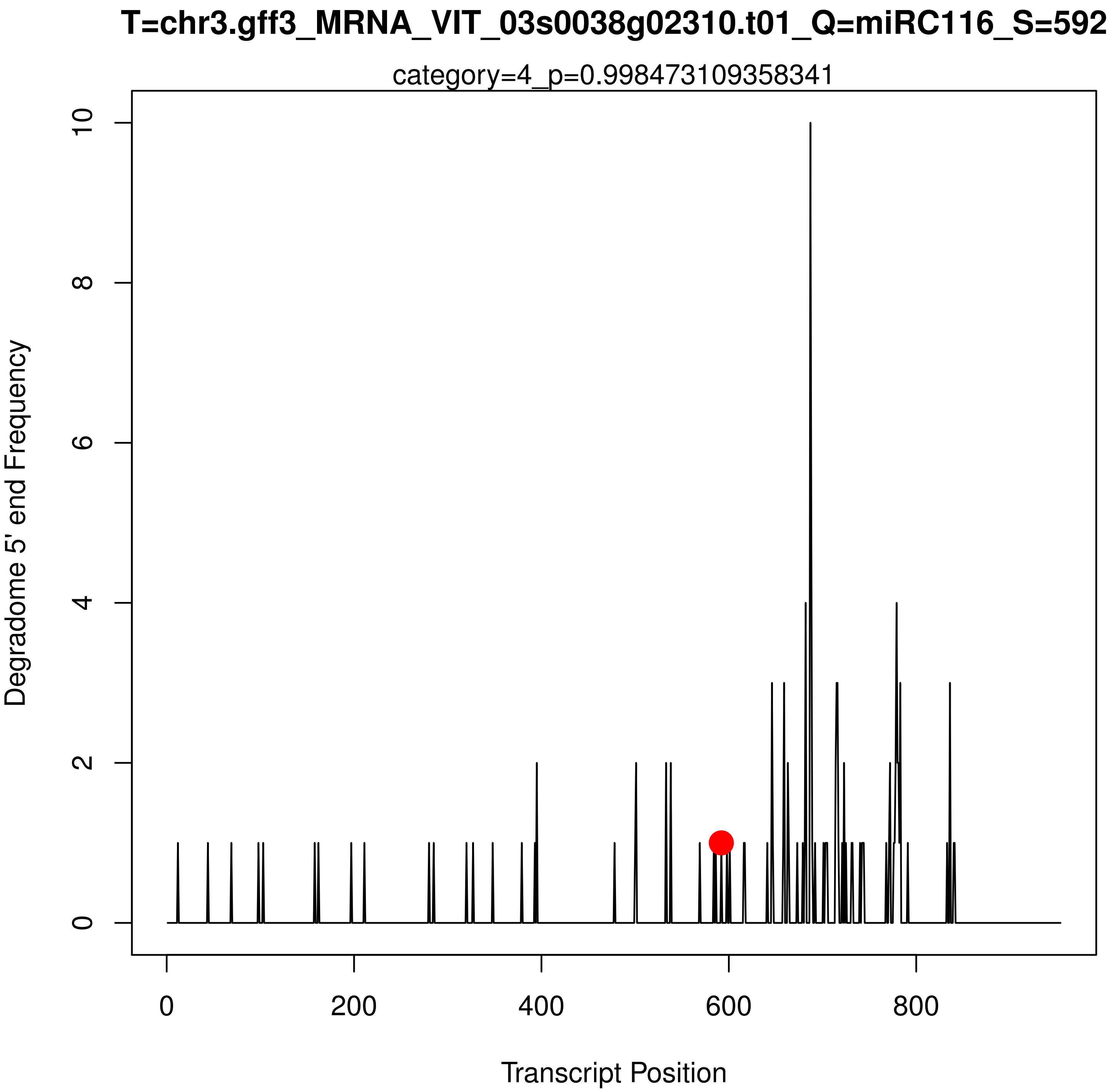
T=chr3.gff3_MRNA_VIT_03s0038g02310.t01_Q=miRC116_S=592
category=4_p=0.998473109358341
Degradome 5' end Frequency
0
2
4
6
8
10
0
200
400
600
800
Transcript Position

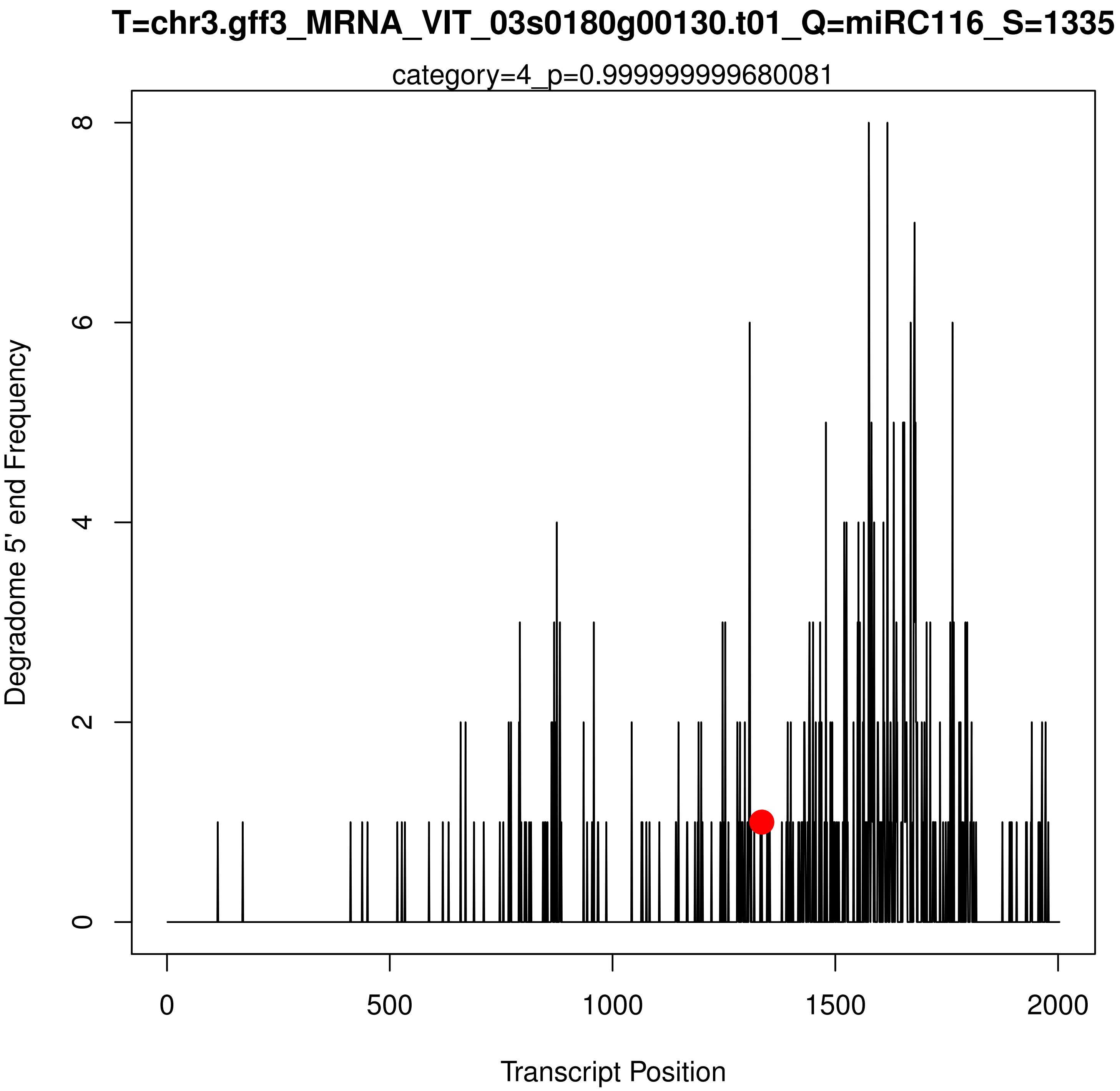
T=chr3.gff3_MRNA_VIT_03s0180g00130.t01_Q=miRC116_S=1335
category=4_p=0.999999999680081
Degradome 5' end Frequency
0
2
4
6
8
0
500
1000
1500
2000
Transcript Position

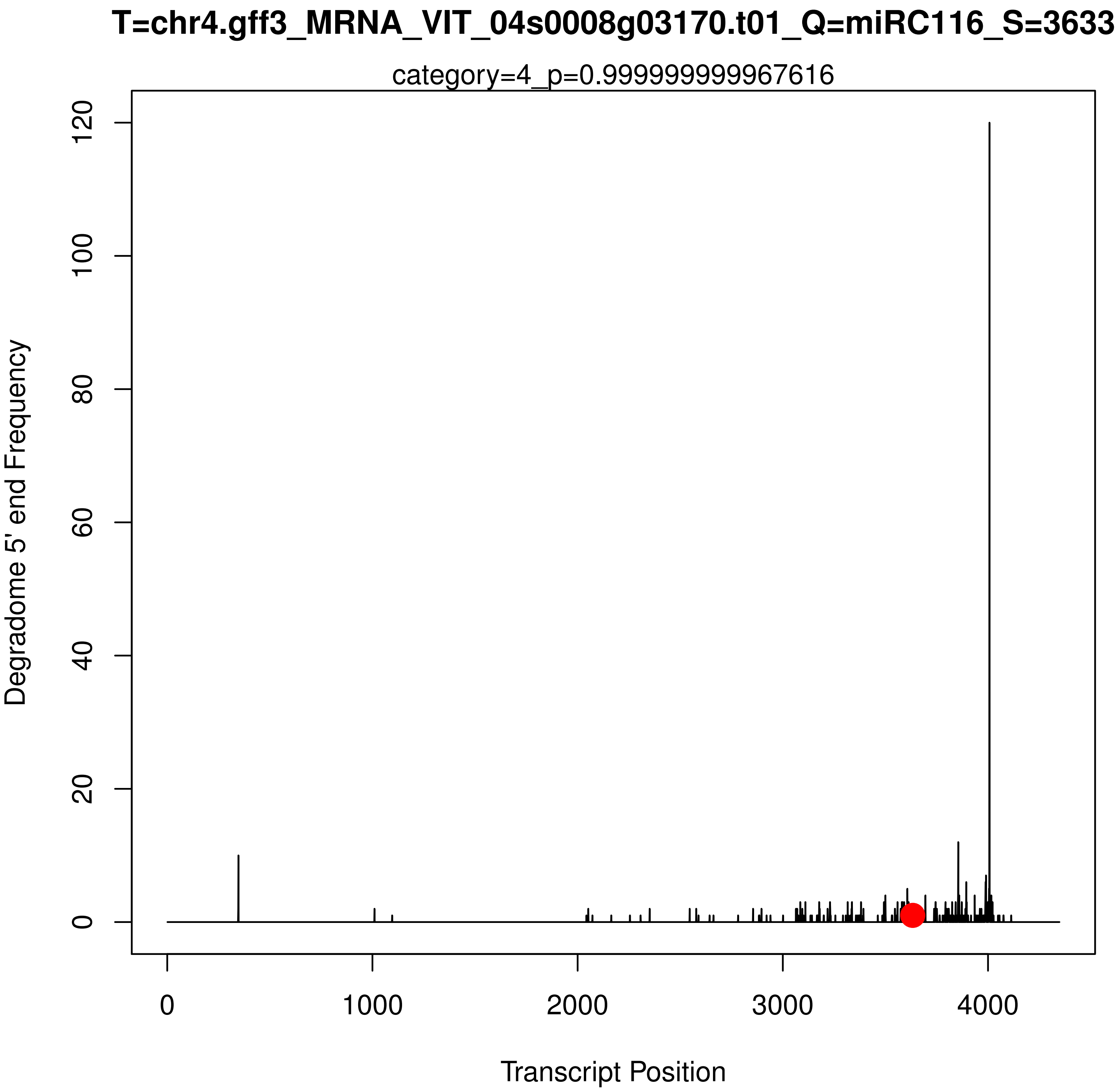

T=chr4.gff3_MRNA_VIT_04s0008g03170.t01_Q=miRC116_S=3633
category=4_p=0.999999999967616
Degradome 5' end Frequency
0
20
40
60
80
100
120
0
1000
2000
3000
4000
Transcript Position

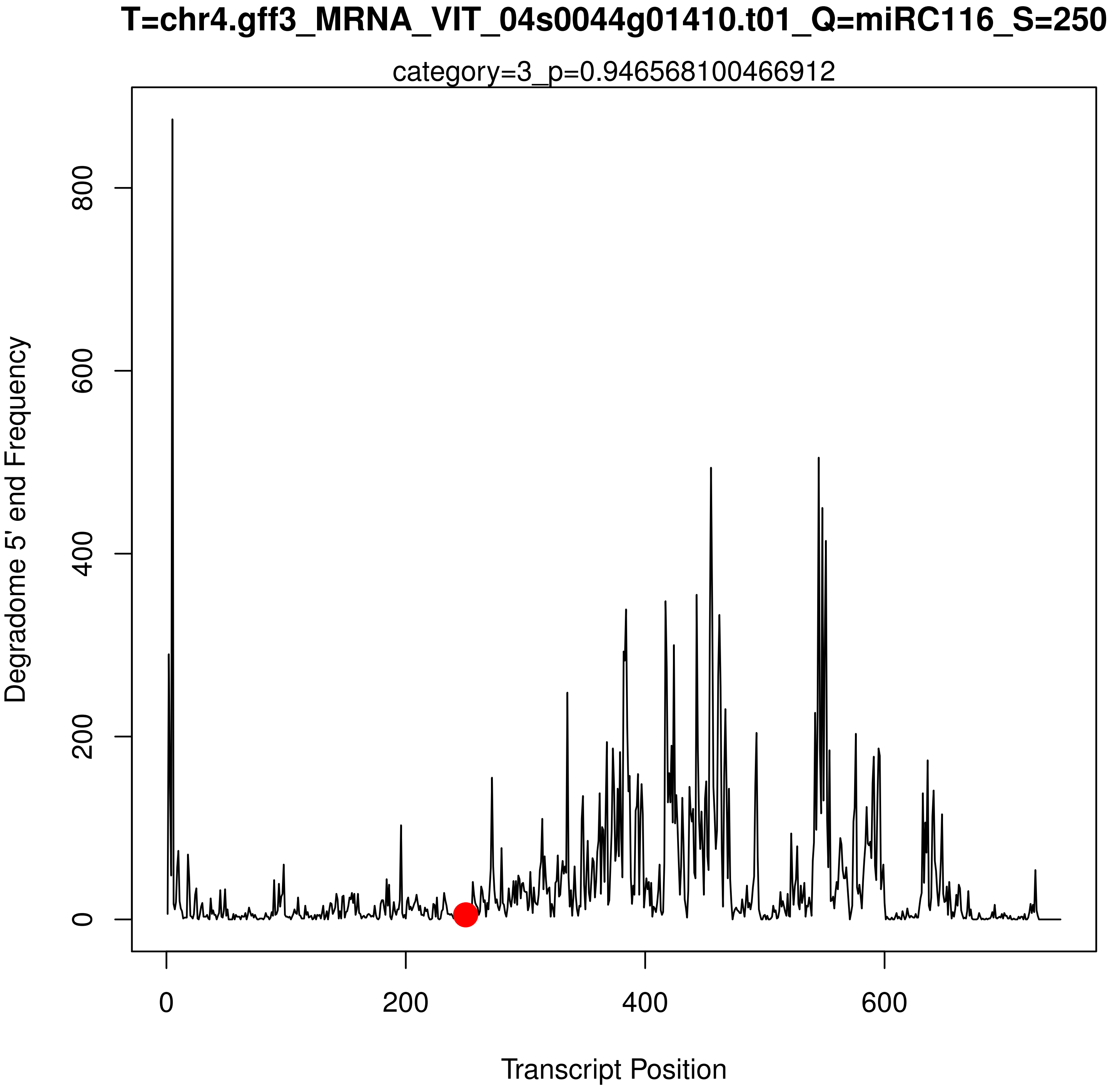

T=chr4.gff3_MRNA_VIT_04s0044g01410.t01_Q=miRC116_S=250
category=3_p=0.946568100466912
Degradome 5' end Frequency
0
200
400
600
800
0
200
400
600
Transcript Position

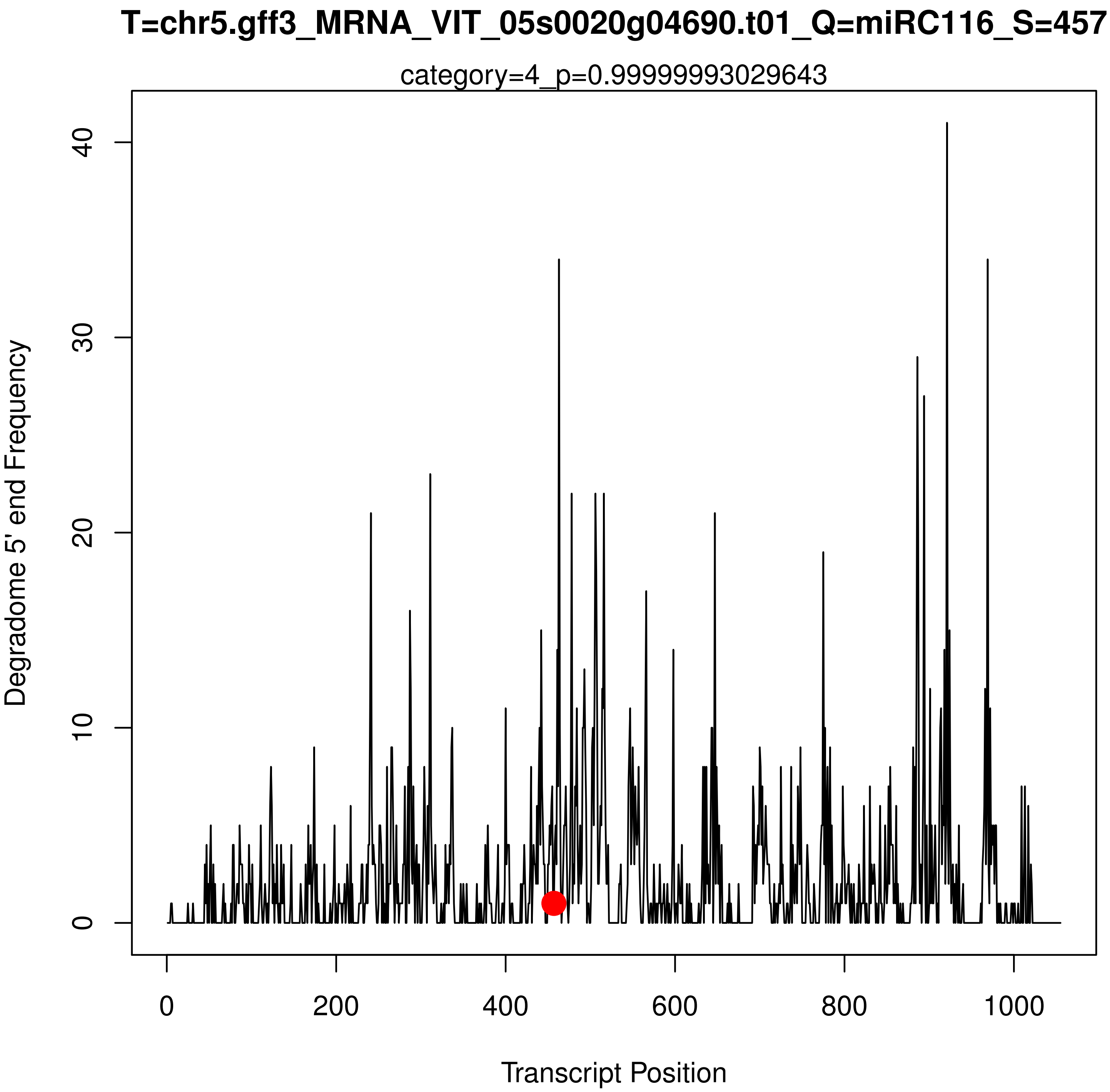
T=chr5.gff3_MRNA_VIT_05s0020g04690.t01_Q=miRC116_S=457
category=4_p=0.99999993029643
Degradome 5' end Frequency
Transcript Position
0
10
20
30
40
0
200
400
600
800
1000

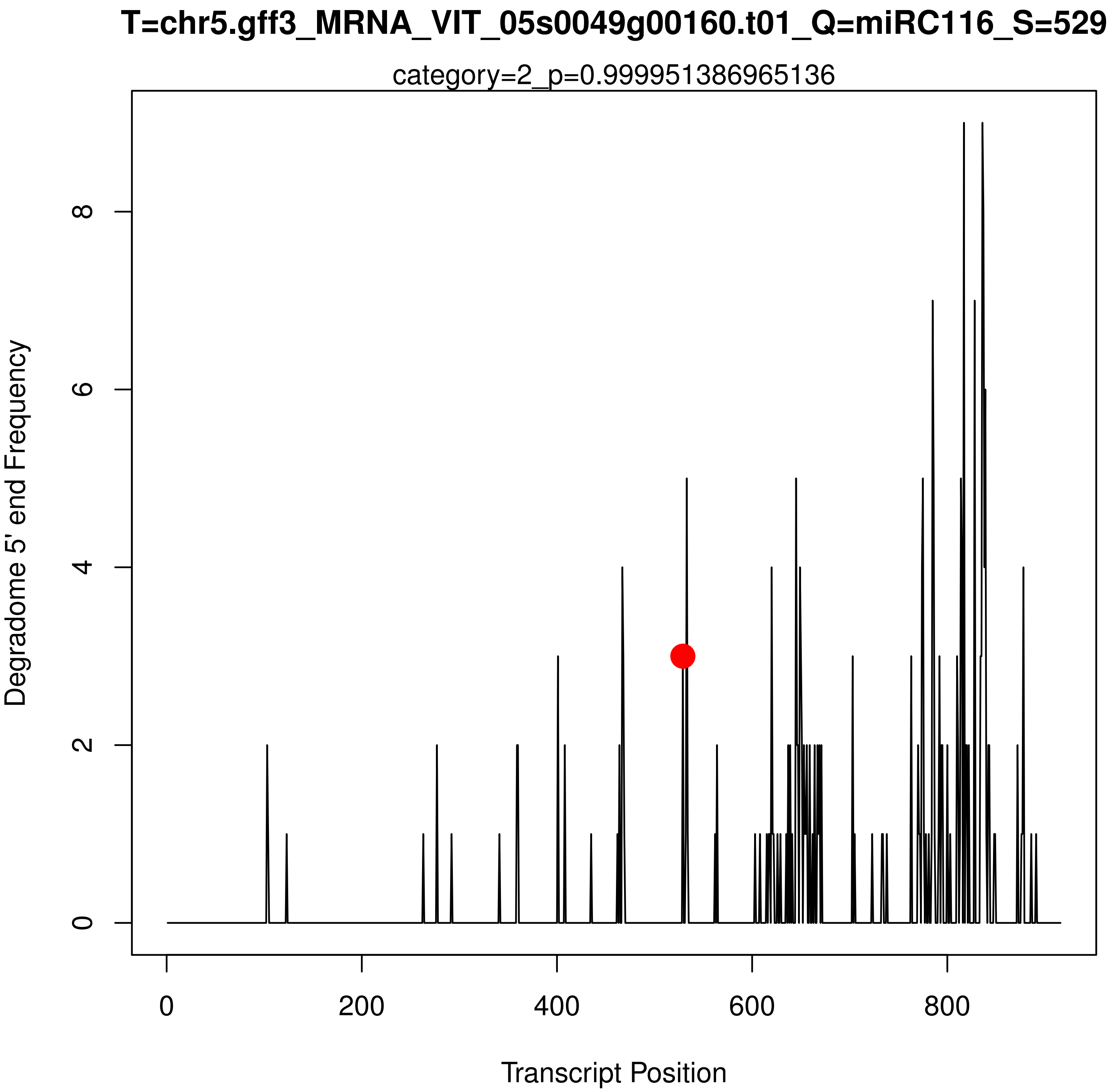

T=chr5.gff3_MRNA_VIT_05s0049g00160.t01_Q=miRC116_S=529
category=2_p=0.999951386965136
Degradome 5' end Frequency
Transcript Position
0
2
4
6
8
0
200
400
600
800

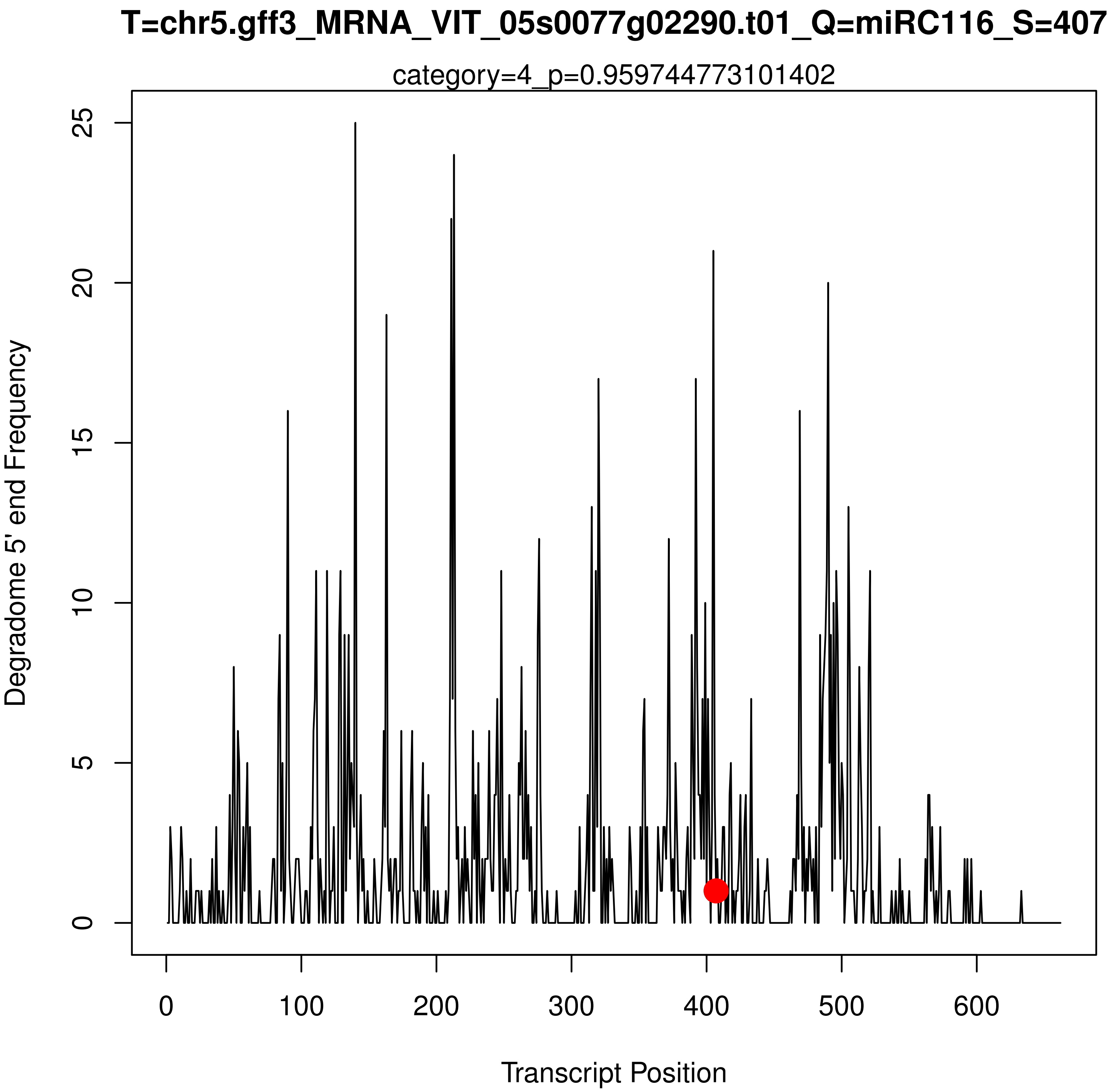
T=chr5.gff3_MRNA_VIT_05s0077g02290.t01_Q=miRC116_S=407
category=4_p=0.959744773101402
Degradome 5' end Frequency
0
5
10
15
20
25
0
100
200
300
400
500
600
Transcript Position

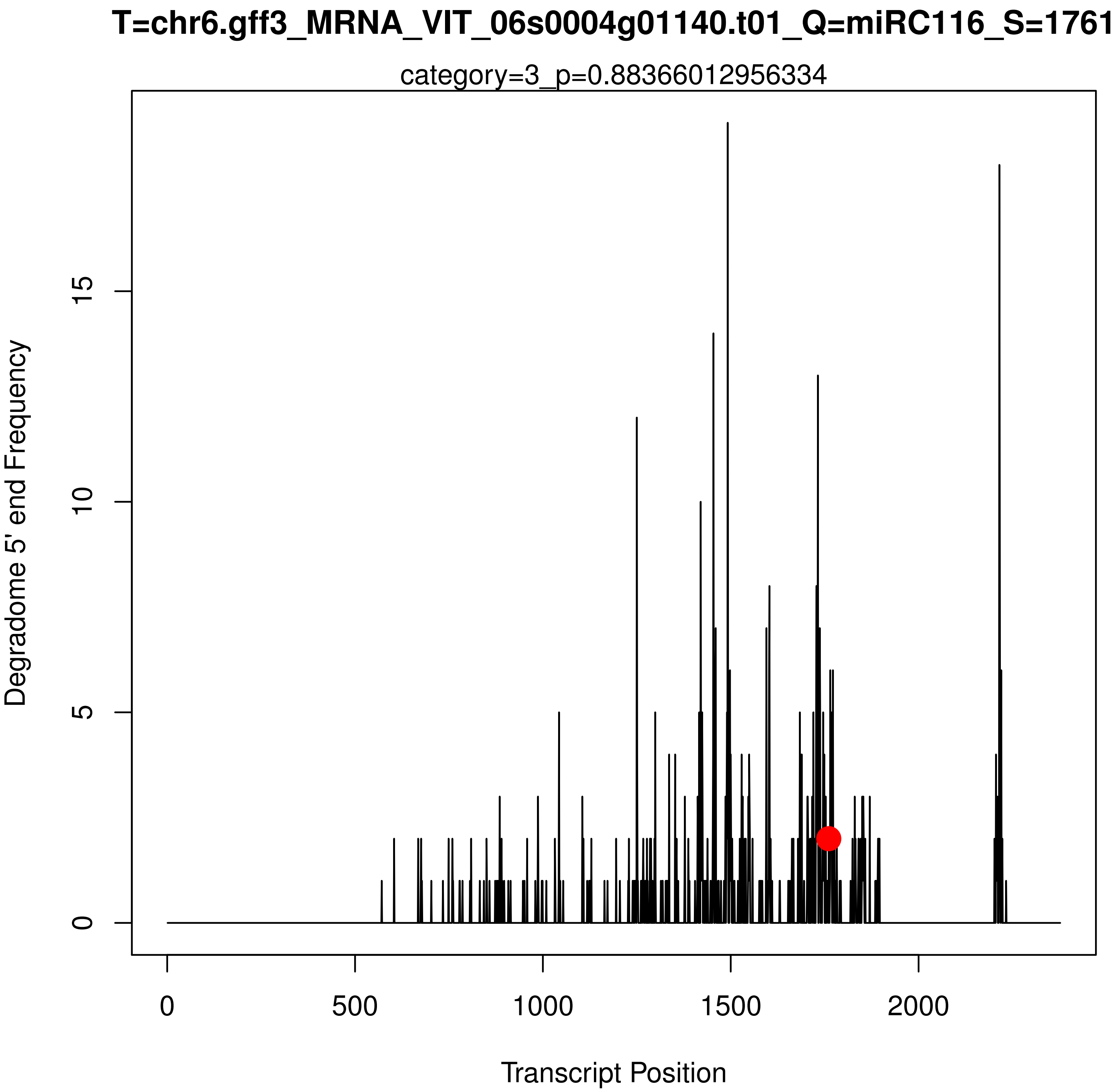
T=chr6.gff3_MRNA_VIT_06s0004g01140.t01_Q=miRC116_S=1761
category=3_p=0.88366012956334
Degradome 5' end Frequency
0
5
10
15
0
500
1000
1500
2000
Transcript Position

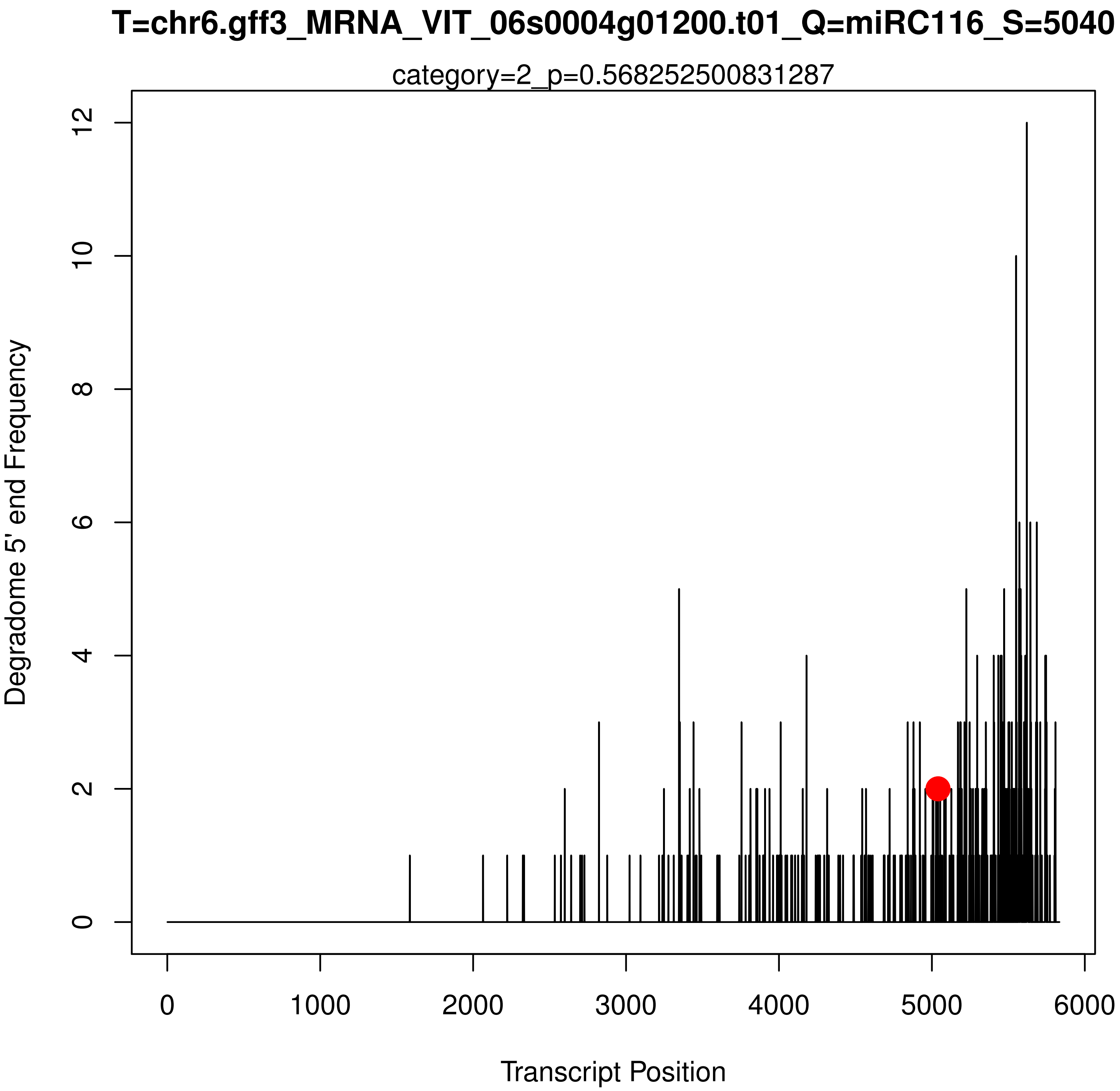

T=chr6.gff3_MRNA_VIT_06s0004g01200.t01_Q=miRC116_S=5040
category=2_p=0.568252500831287
Degradome 5' end Frequency
0
2
4
6
8
10
12
0
1000
2000
3000
4000
5000
6000
Transcript Position

T=chr7.gff3_MRNA_VIT_07s0031g00050.t01_Q=miRC116_S=153

category=4_p=0.996273017956983

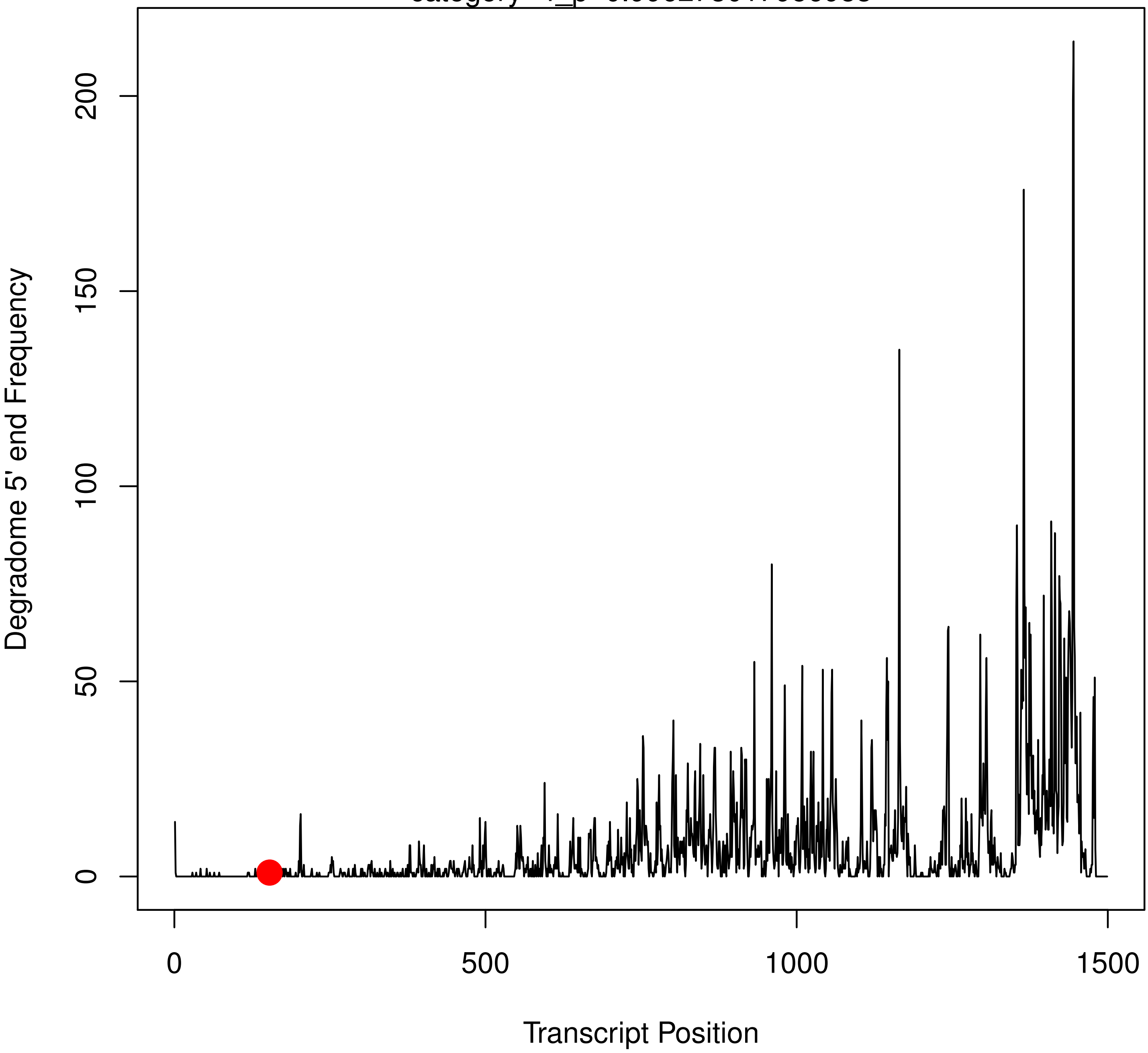

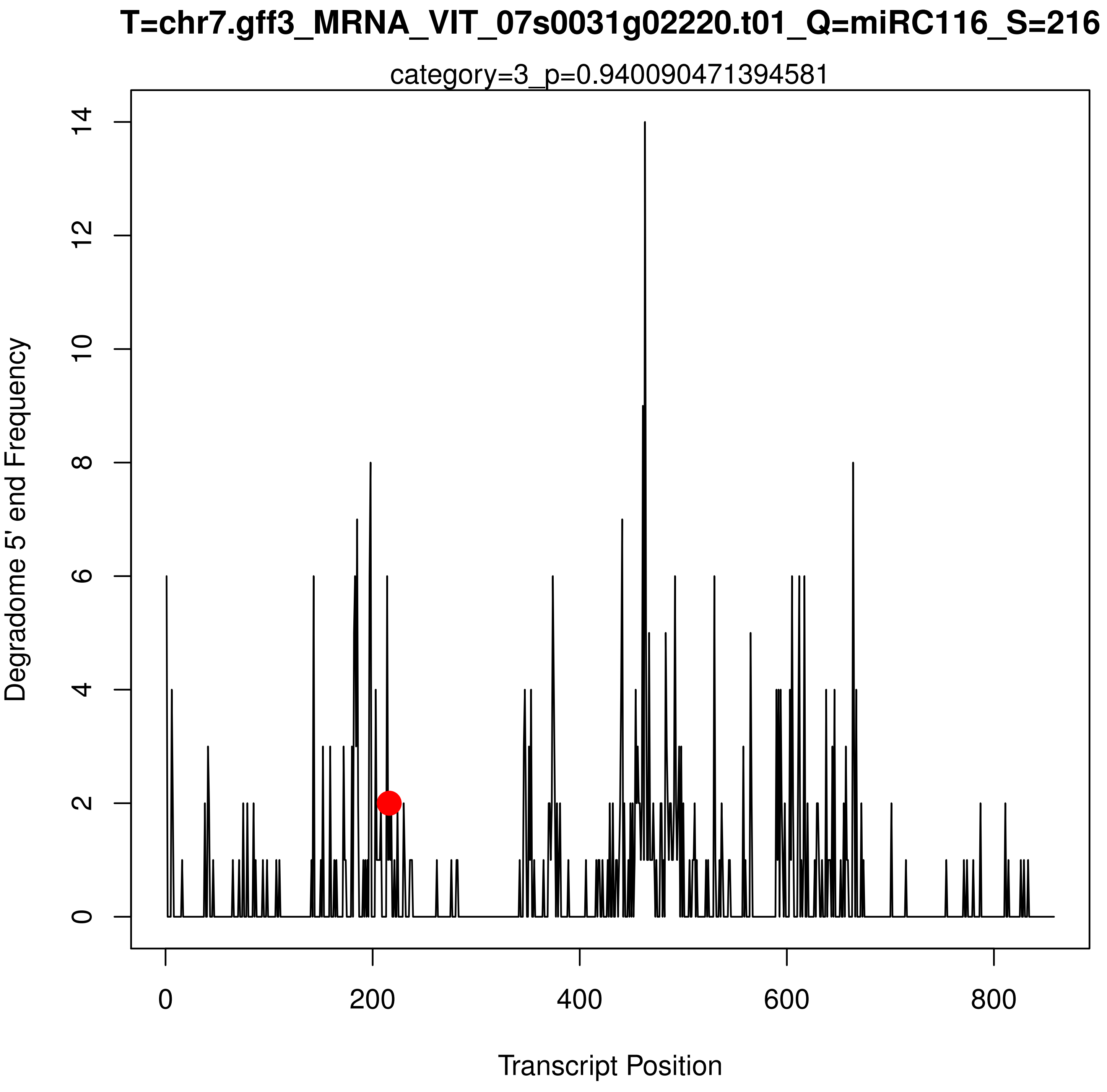
T=chr7.gff3_MRNA_VIT_07s0031g02220.t01_Q=miRC116_S=216
category=3_p=0.940090471394581
Degradome 5' end Frequency
0
2
4
6
8
10
12
14
0
200
400
600
800
Transcript Position

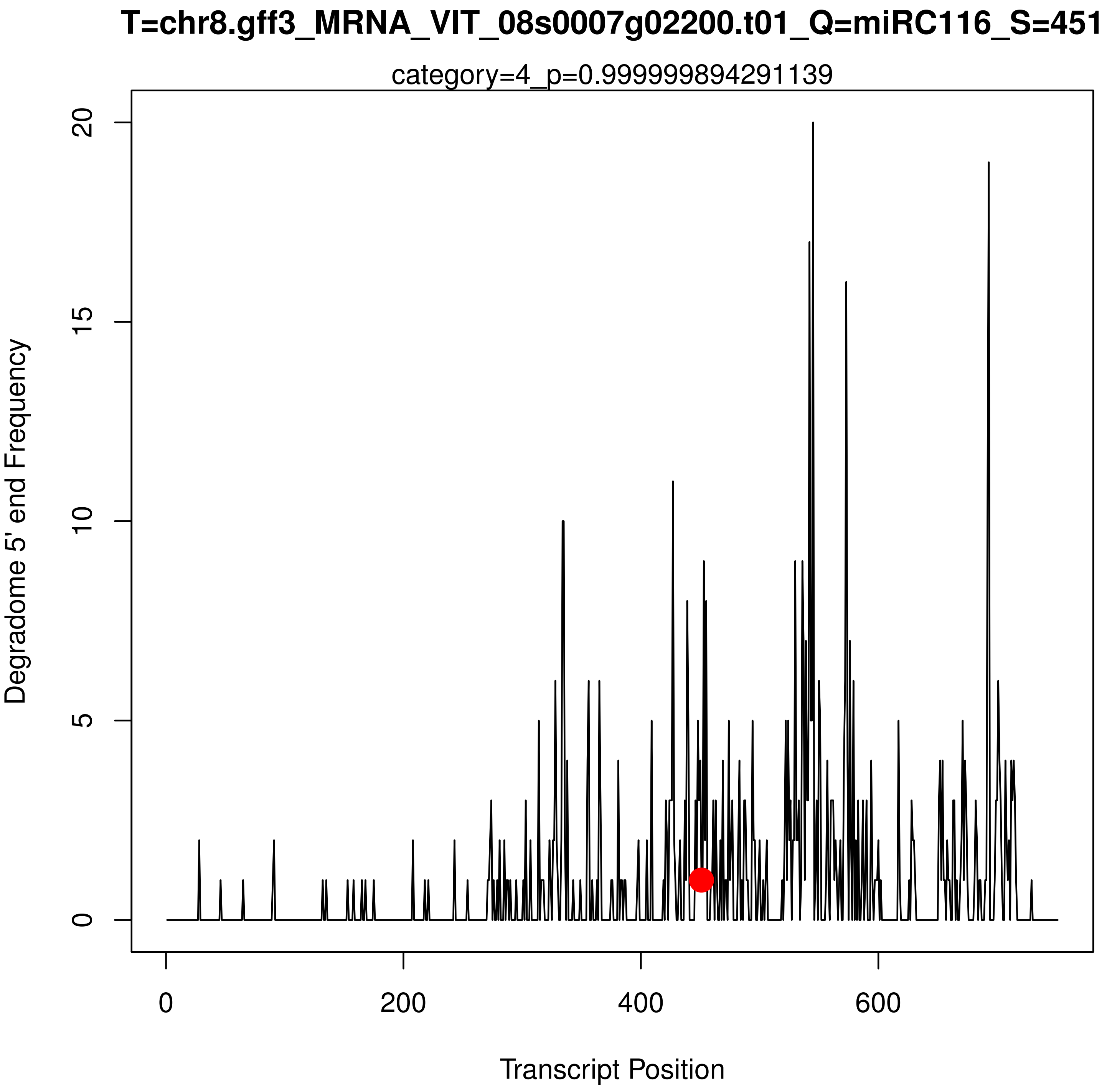
T=chr8.gff3_MRNA_VIT_08s0007g02200.t01_Q=miRC116_S=451
category=4_p=0.999999894291139
Degradome 5' end Frequency
Transcript Position
0
200
400
600
0
5
10
15
20

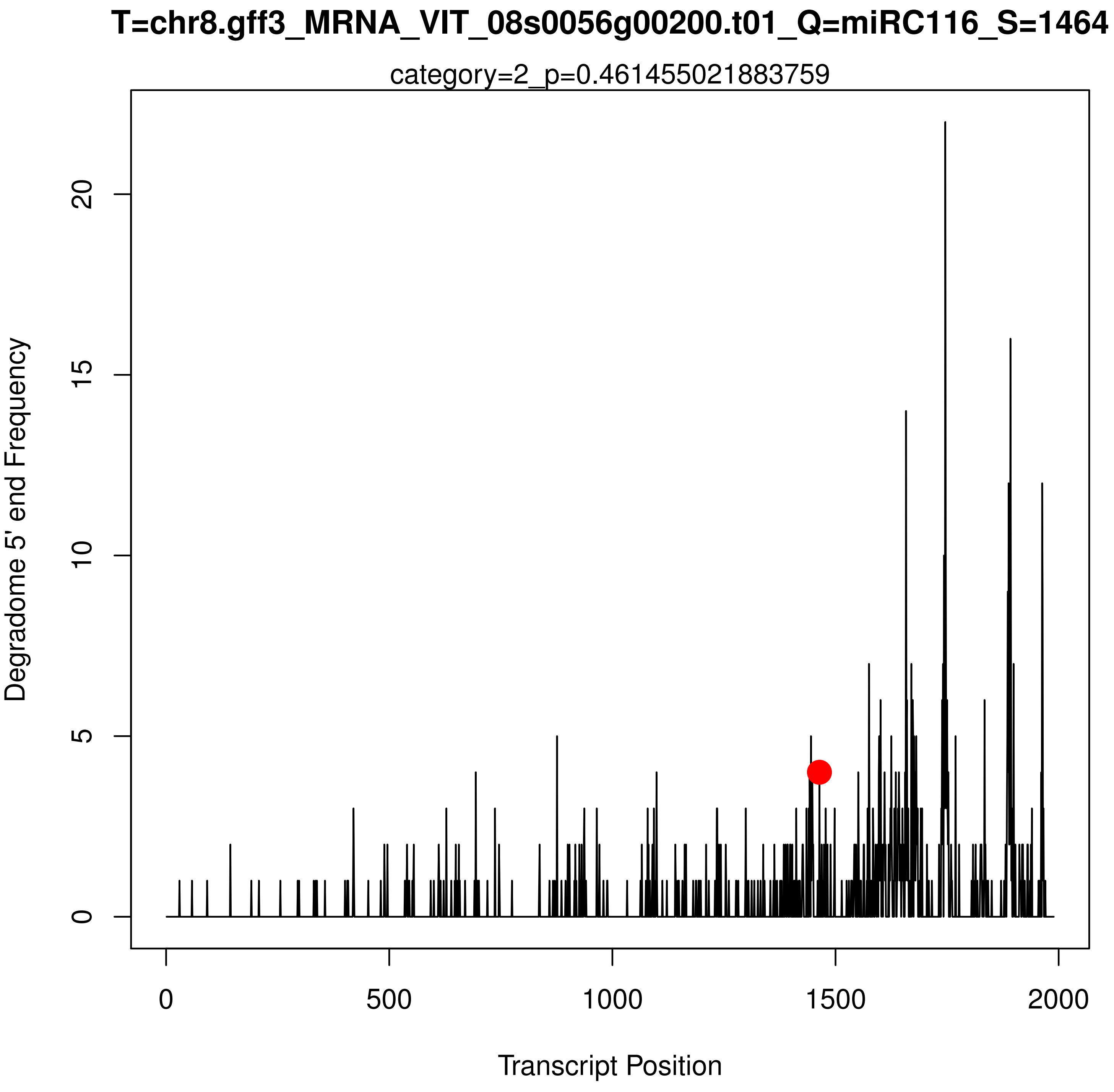
T=chr8.gff3_MRNA_VIT_08s0056g00200.t01_Q=miRC116_S=1464
category=2_p=0.461455021883759
Degradome 5' end Frequency
0
5
10
15
20
0
500
1000
1500
2000
Transcript Position

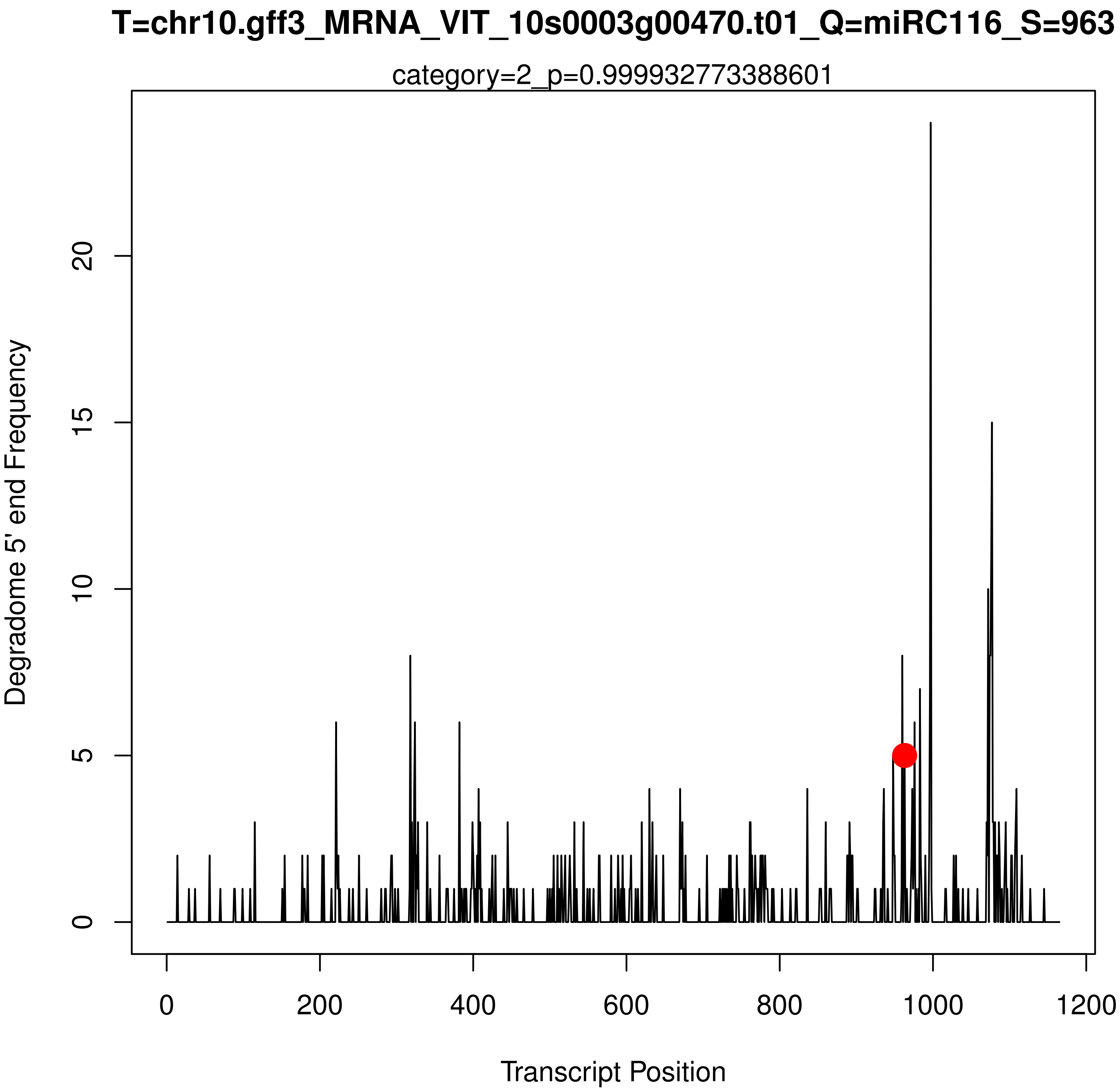
T=chr10.gff3_MRNA_VIT_10s0003g00470.t01_Q=miRC116_S=963
category=2_p=0.999932773388601
Degradome 5' end Frequency
Transcript Position
0
5
10
15
20
0
200
400
600
800
1000
1200

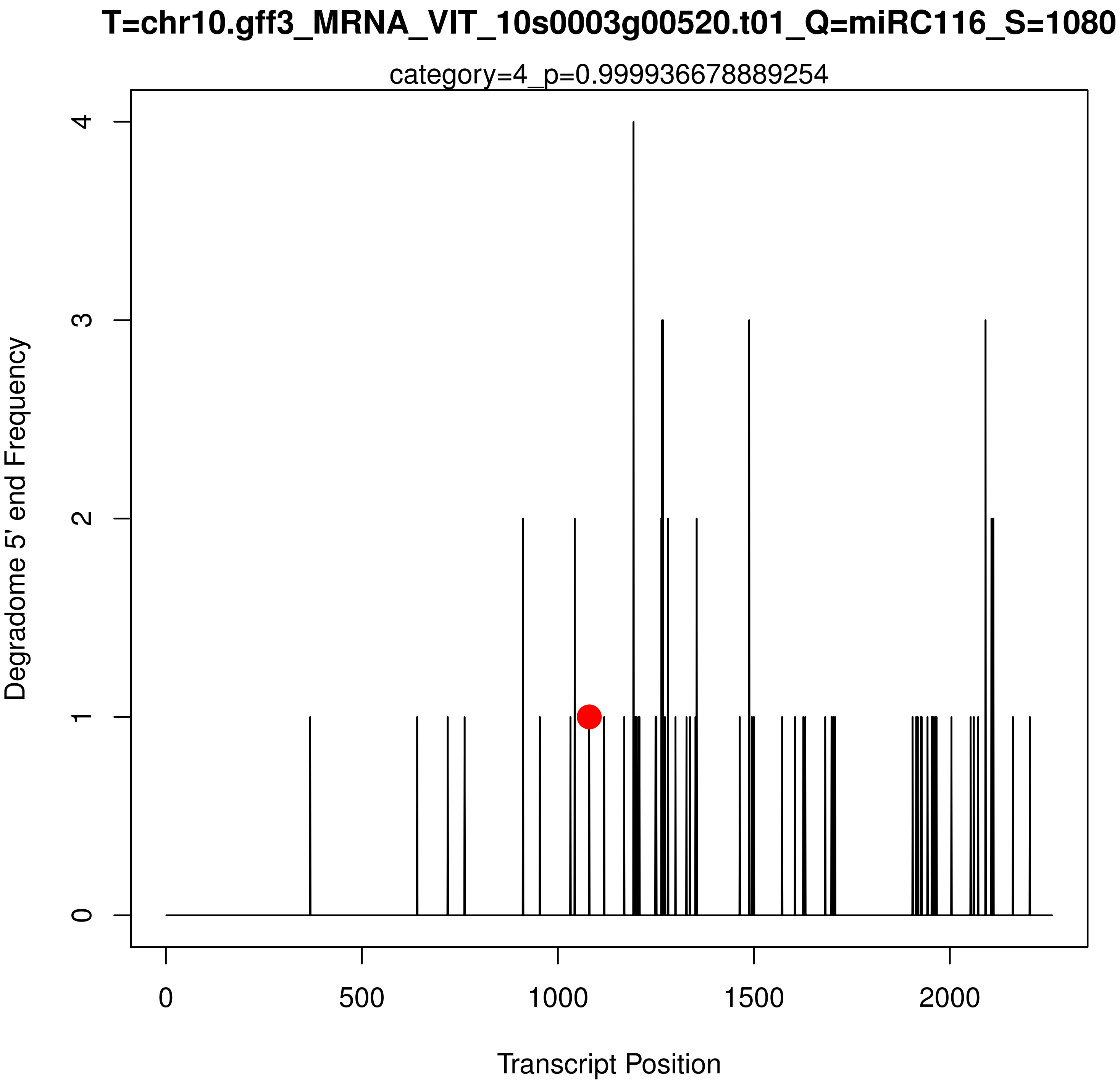
T=chr10.gff3_MRNA_VIT_10s0003g00520.t01_Q=miRC116_S=1080
category=4_p=0.999936678889254
Degradome 5' end Frequency
0
1
2
3
4
0
500
1000
1500
2000
Transcript Position

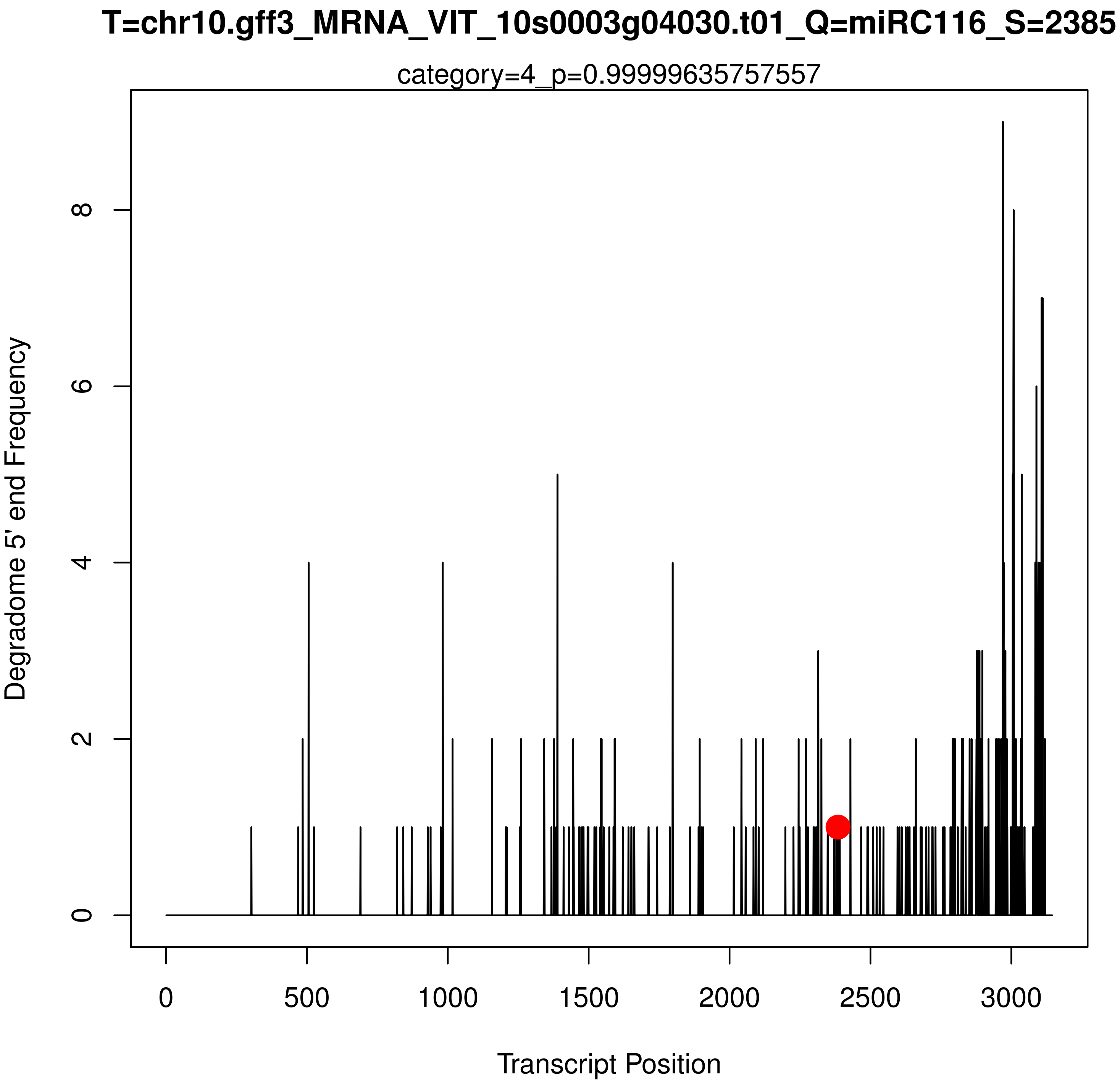

T=chr10.gff3_MRNA_VIT_10s0003g04030.t01_Q=miRC116_S=2385
category=4_p=0.99999635757557
Degradome 5' end Frequency
0
2
4
6
8
0
500
1000
1500
2000
2500
3000
Transcript Position

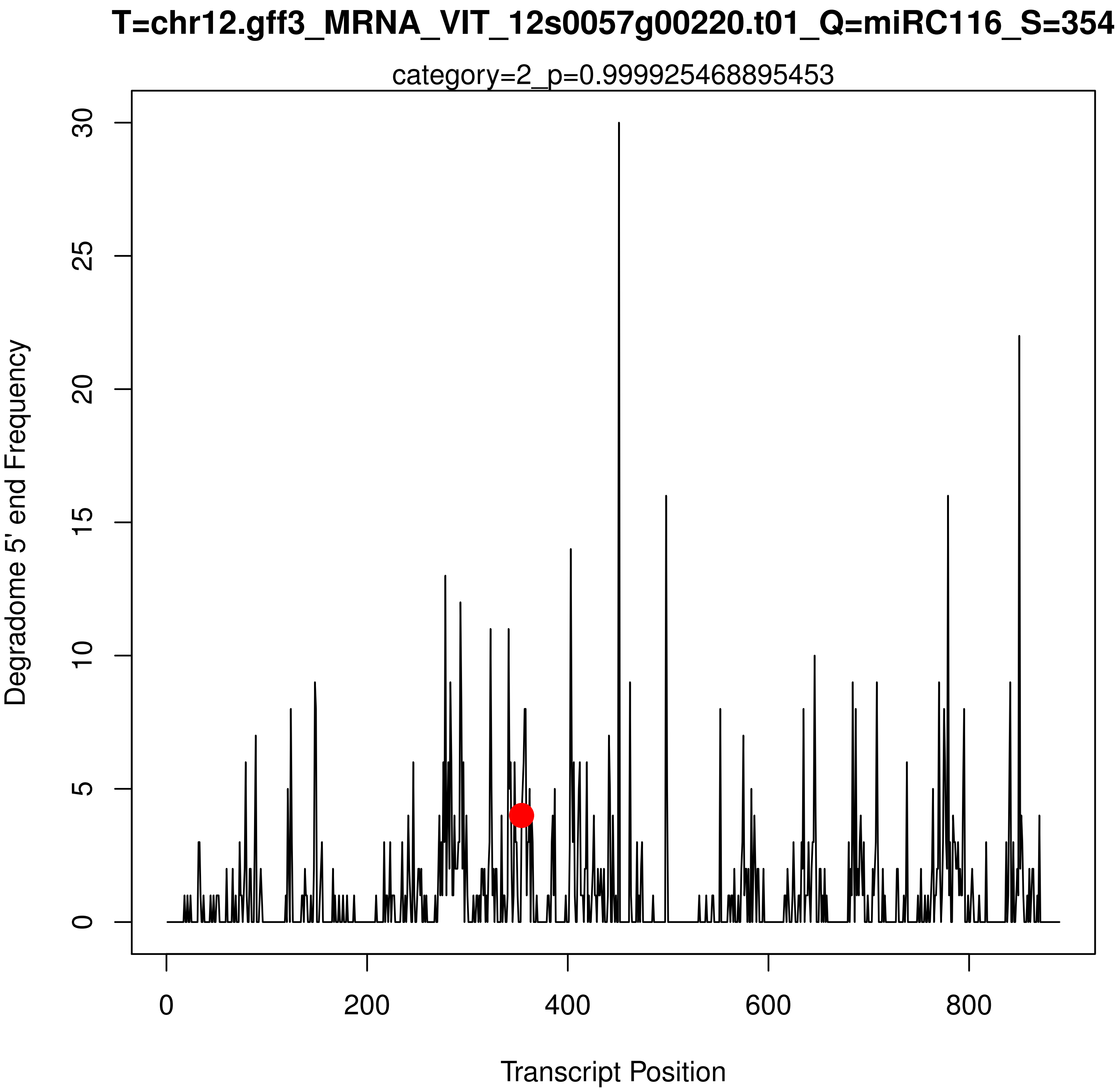

T=chr12.gff3_MRNA_VIT_12s0057g00220.t01_Q=miRC116_S=354
category=2_p=0.999925468895453
Degradome 5' end Frequency
0
5
10
15
20
25
30
0
200
400
600
800
Transcript Position

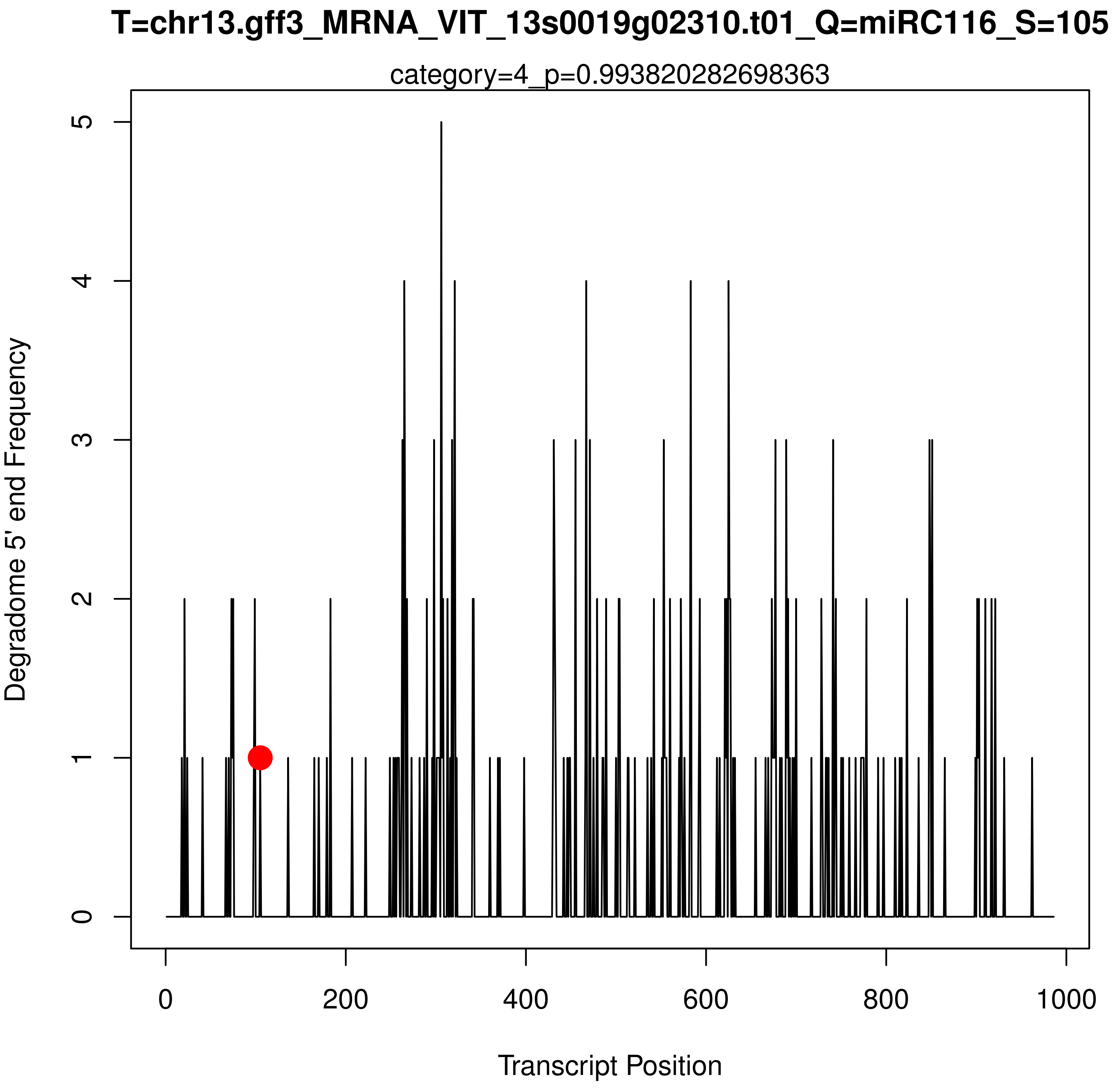

T=chr13.gff3_MRNA_VIT_13s0019g02310.t01_Q=miRC116_S=105
category=4_p=0.993820282698363
Degradome 5' end Frequency
0
1
2
3
4
5
0
200
400
600
800
1000
Transcript Position

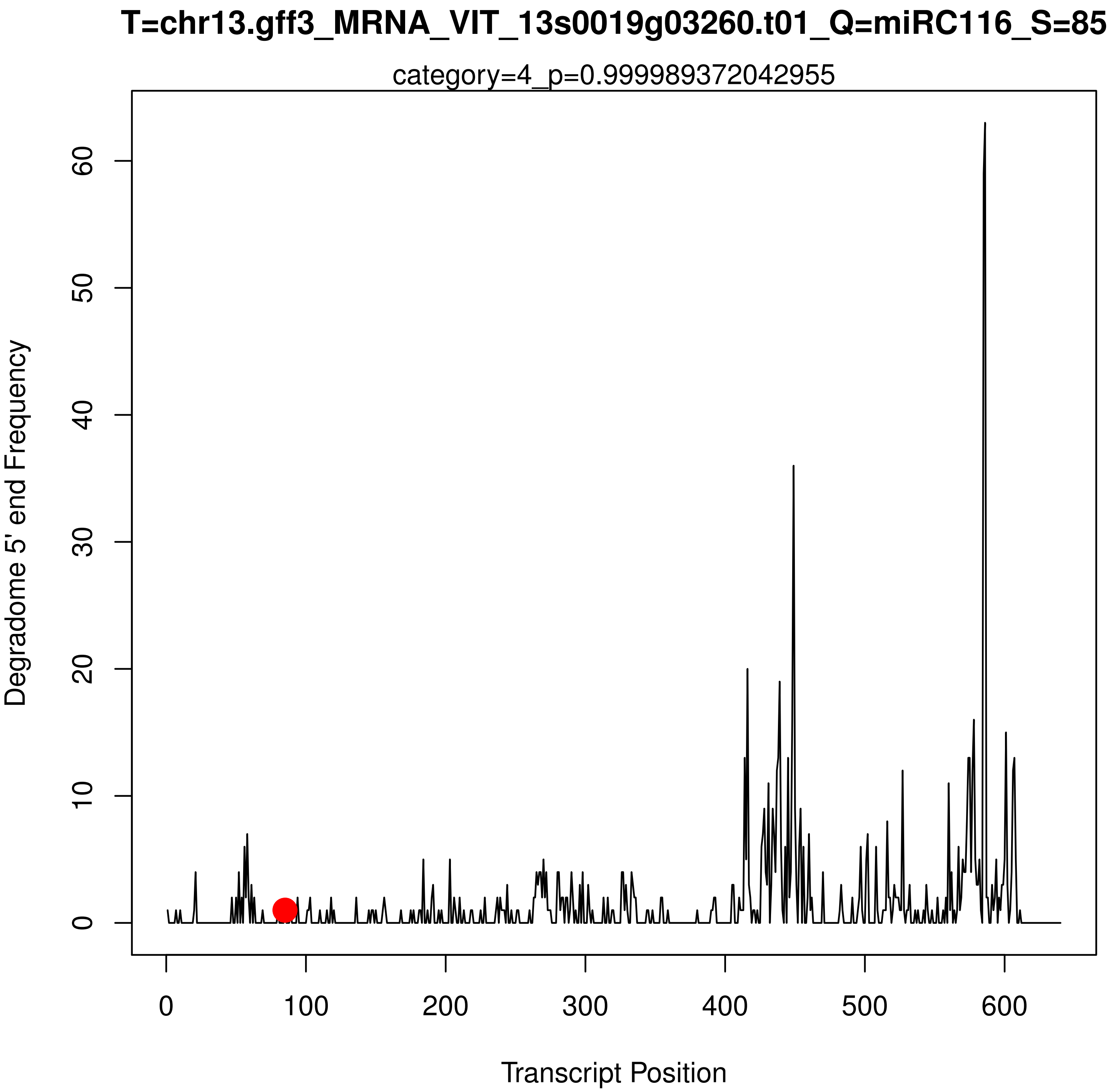
T=chr13.gff3_MRNA_VIT_13s0019g03260.t01_Q=miRC116_S=85
category=4_p=0.999989372042955
Degradome 5' end Frequency
0
10
20
30
40
50
60
0
100
200
300
400
500
600
Transcript Position

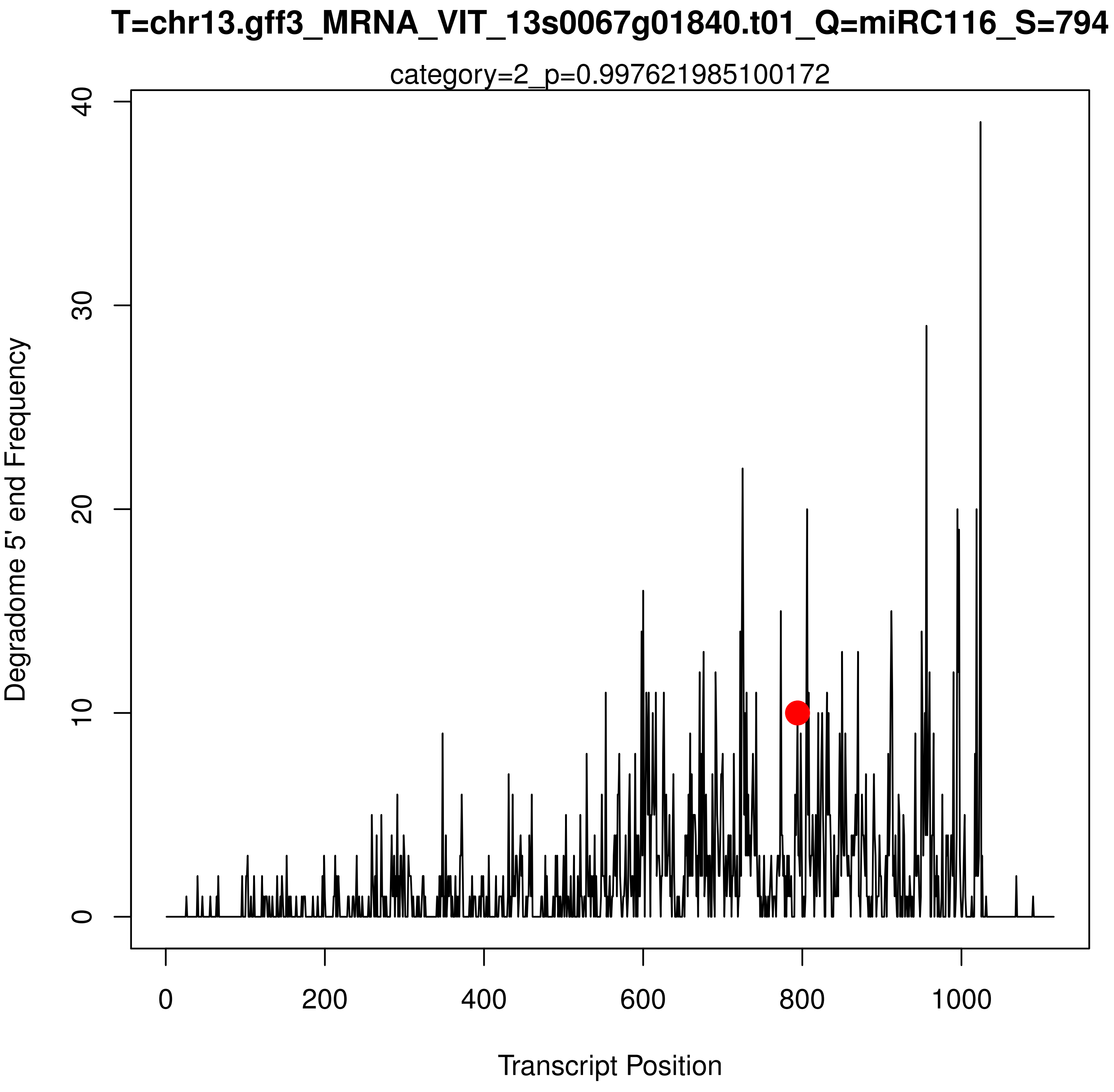

T=chr13.gff3_MRNA_VIT_13s0067g01840.t01_Q=miRC116_S=794
category=2_p=0.997621985100172
Degradome 5' end Frequency
Transcript Position
0
10
20
30
40
0
200
400
600
800
1000

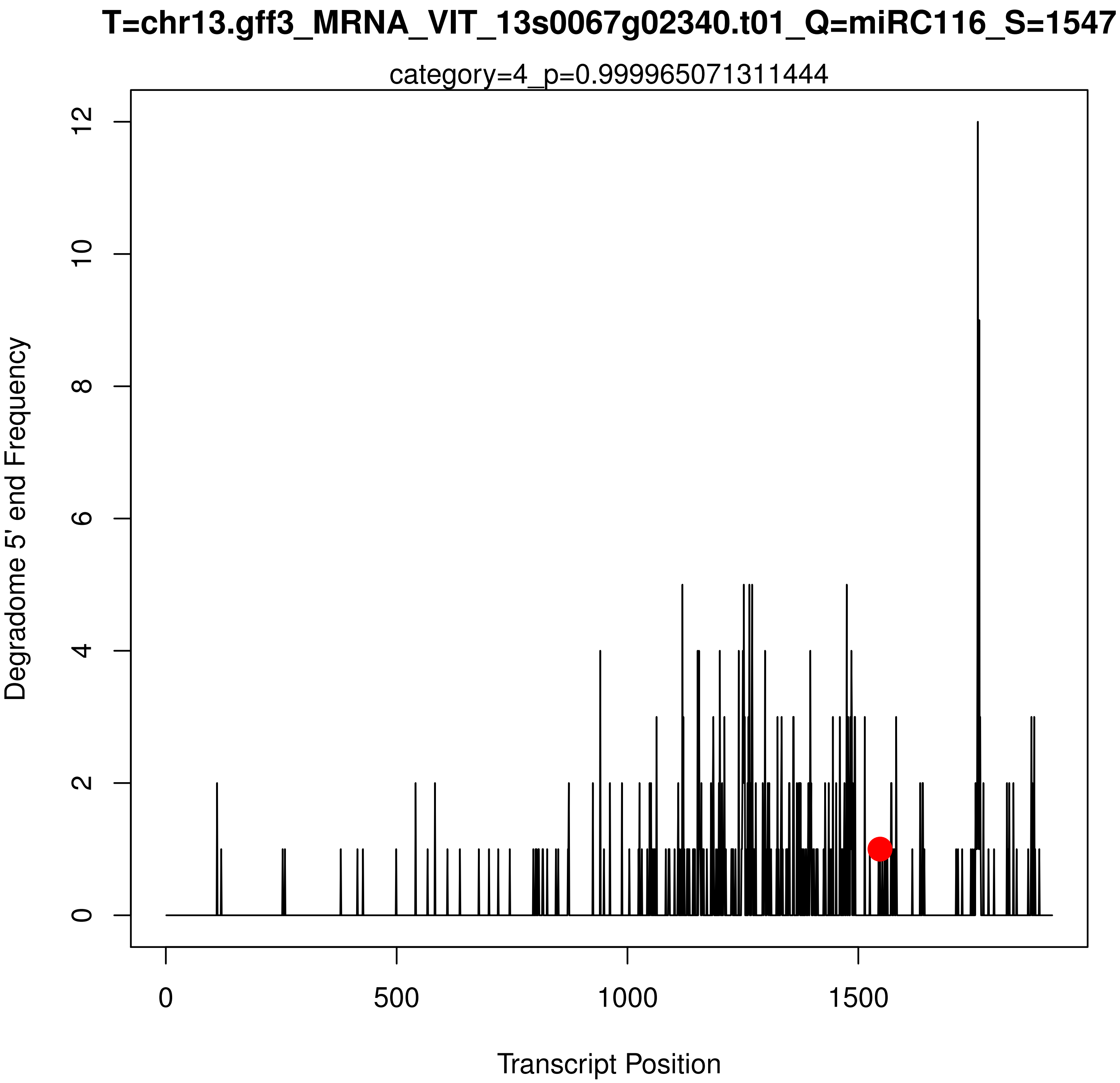
T=chr13.gff3_MRNA_VIT_13s0067g02340.t01_Q=miRC116_S=1547
category=4_p=0.999965071311444
Degradome 5' end Frequency
0
2
4
6
8
10
12
0
500
1000
1500
Transcript Position

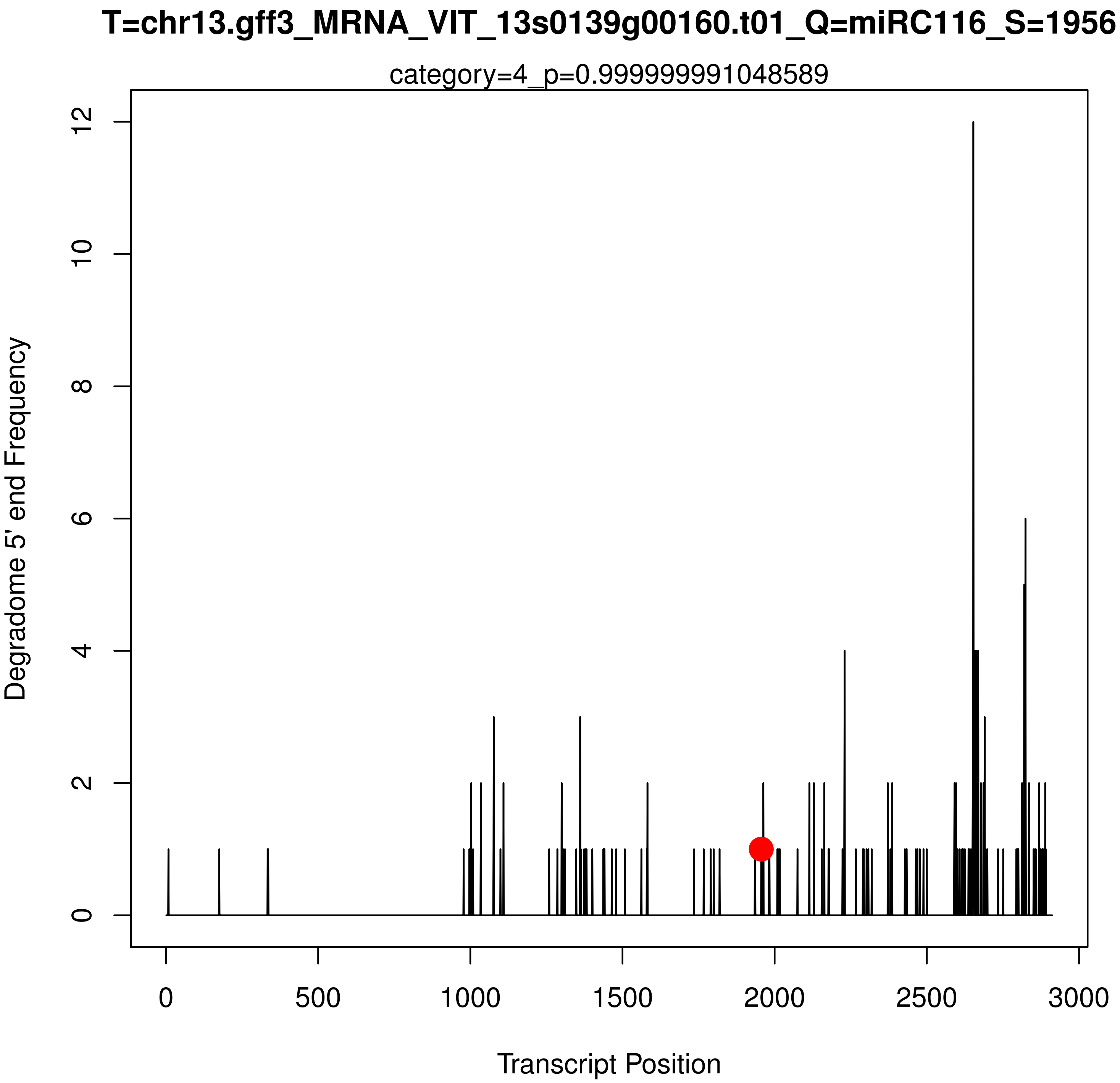
T=chr13.gff3_MRNA_VIT_13s0139g00160.t01_Q=miRC116_S=1956
category=4_p=0.999999991048589
Degradome 5' end Frequency
Transcript Position
0
2
4
6
8
10
12
0
500
1000
1500
2000
2500
3000

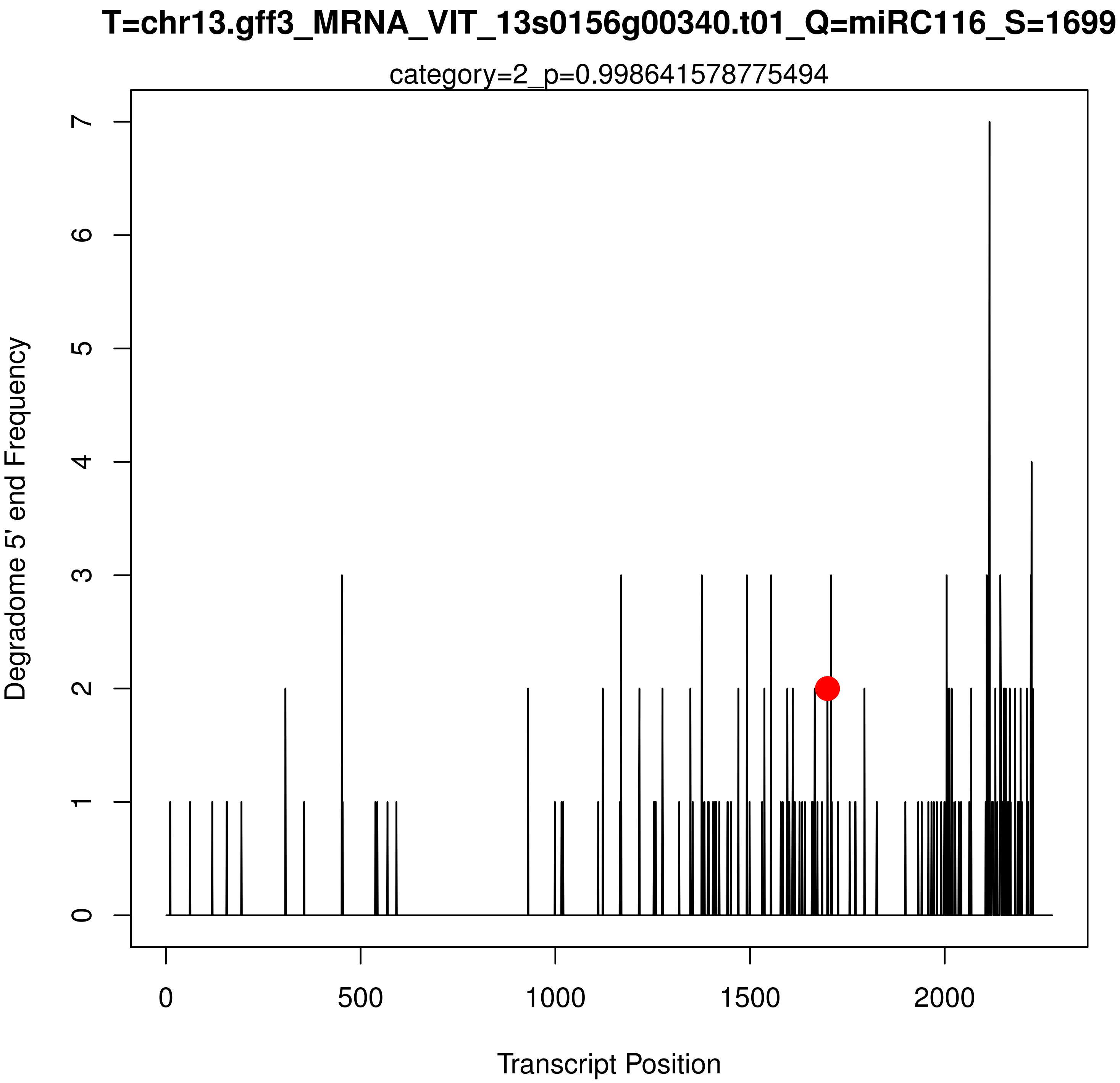

T=chr13.gff3_MRNA_VIT_13s0156g00340.t01_Q=miRC116_S=1699
category=2_p=0.998641578775494
Degradome 5' end Frequency
0
1
2
3
4
5
6
7
0
500
1000
1500
2000
Transcript Position

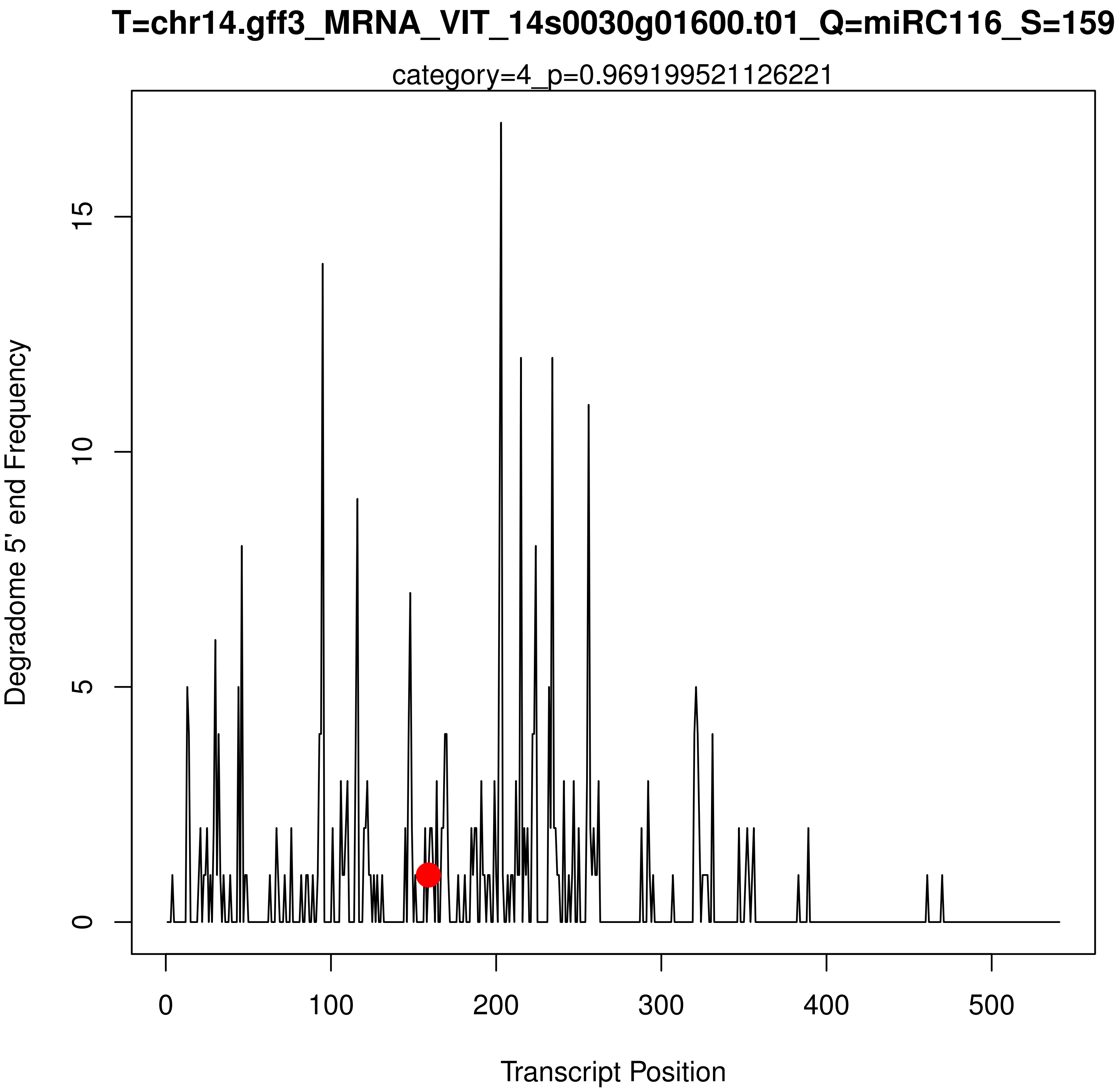
T=chr14.gff3_MRNA_VIT_14s0030g01600.t01_Q=miRC116_S=159
category=4_p=0.969199521126221
Degradome 5' end Frequency
0
5
10
15
0
100
200
300
400
500
Transcript Position

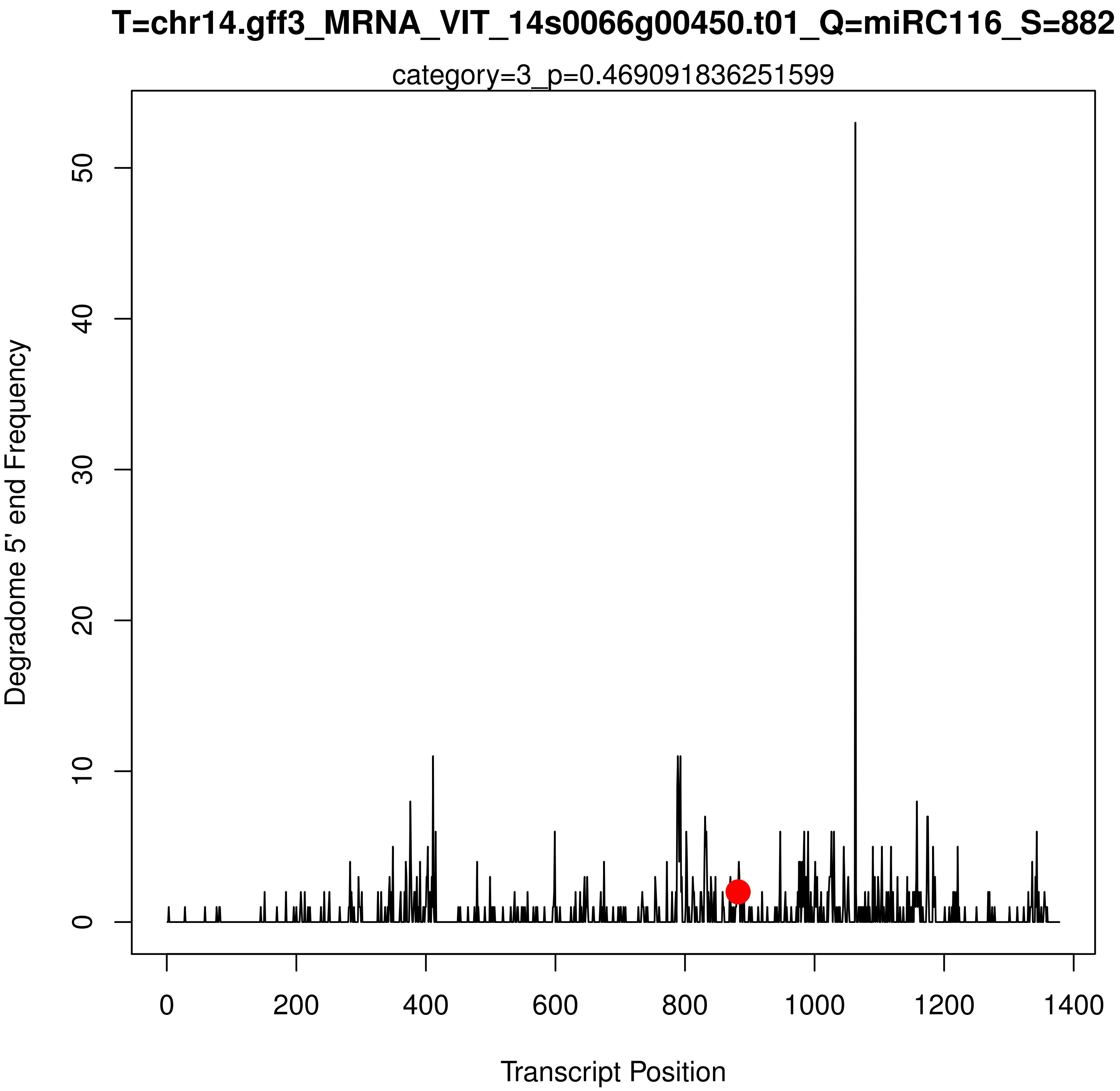
T=chr14.gff3_MRNA_VIT_14s0066g00450.t01_Q=miRC116_S=882
category=3_p=0.469091836251599
Degradome 5' end Frequency
0
10
20
30
40
50
0
200
400
600
800
1000
1200
1400
Transcript Position

T=chr14.gff3_MRNA_VIT_14s0108g01380.t01_Q=miRC116_S=675

category=4_p=0.999999999905509

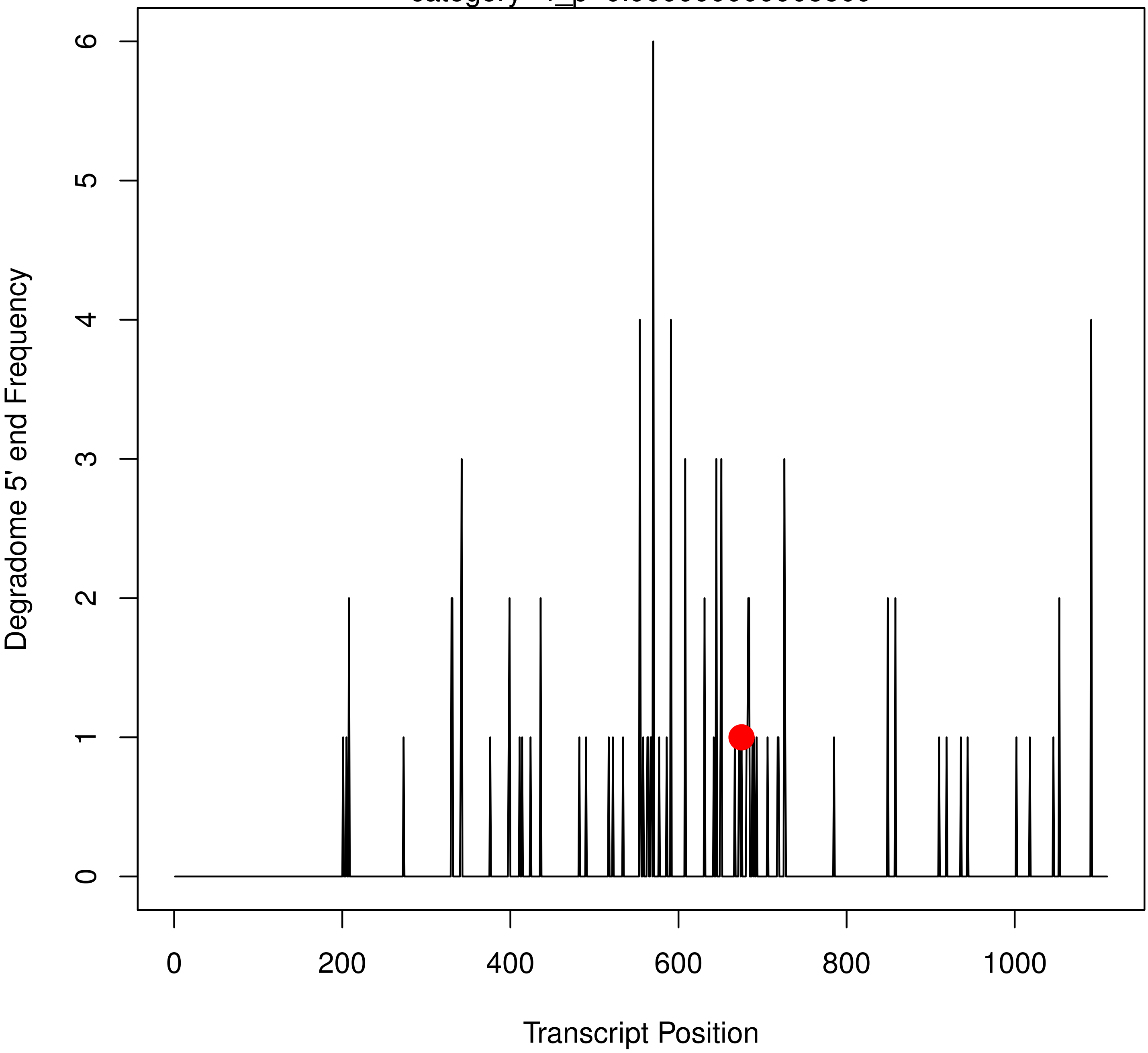

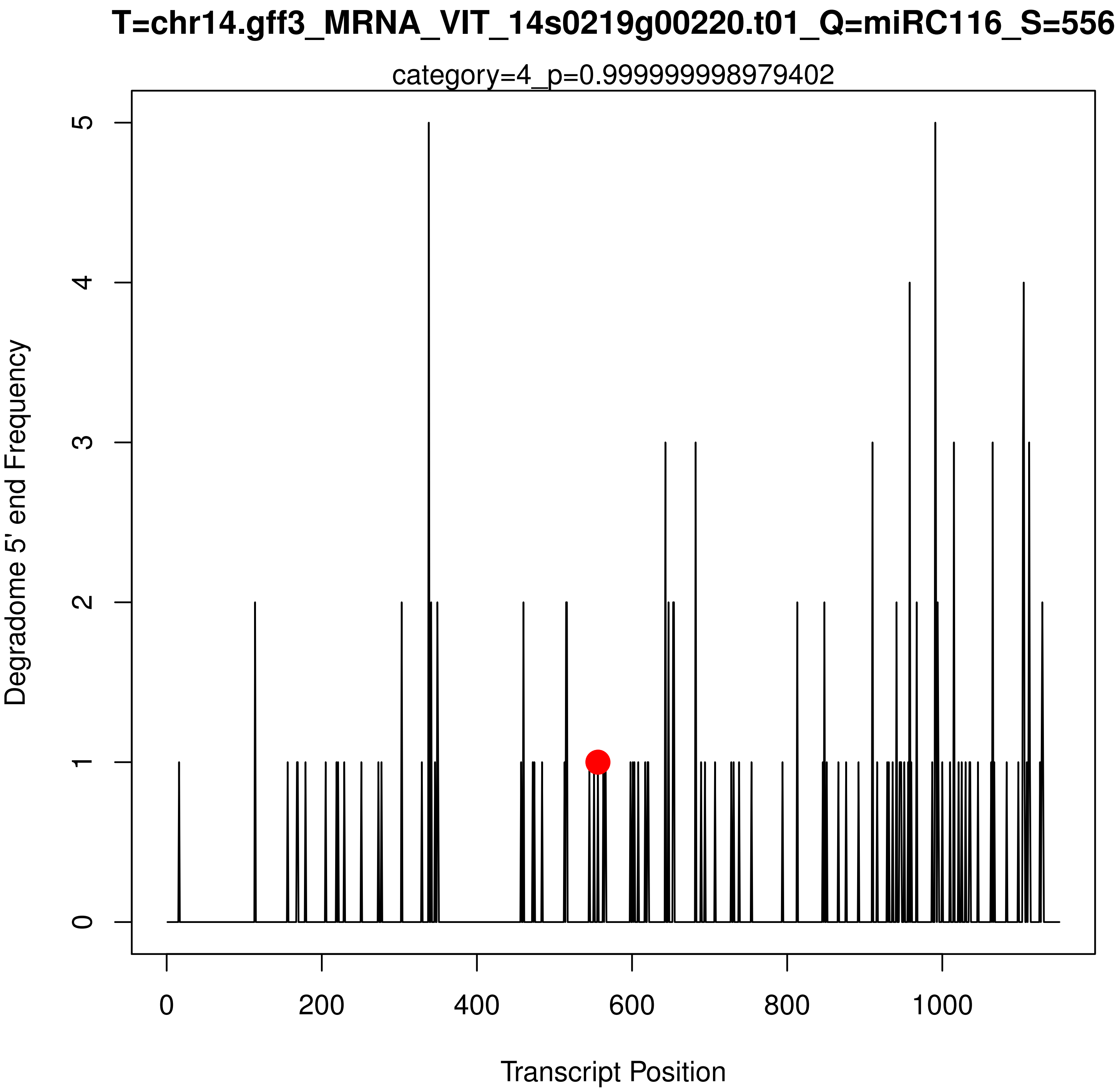
T=chr14.gff3_MRNA_VIT_14s0219g00220.t01_Q=miRC116_S=556
category=4_p=0.999999998979402
Degradome 5' end Frequency
0
1
2
3
4
5
0
200
400
600
800
1000
Transcript Position

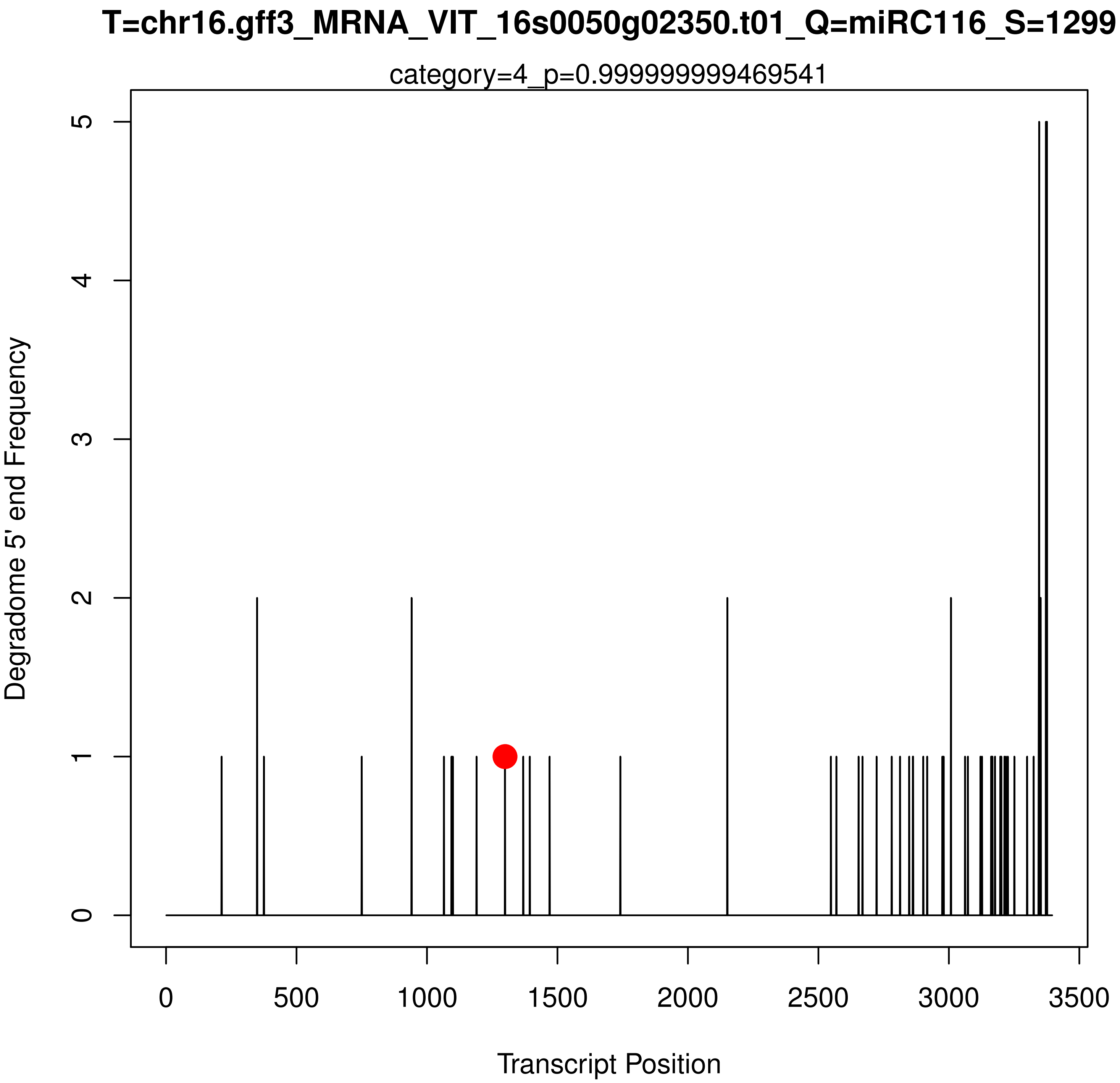
T=chr16.gff3_MRNA_VIT_16s0050g02350.t01_Q=miRC116_S=1299
category=4_p=0.999999999469541
Degradome 5' end Frequency
0
1
2
3
4
5
0
500
1000
1500
2000
2500
3000
3500
Transcript Position

**T=chr16.gff3_MRNA_VIT_16s0098g00660.t01_Q=miRC116_S=364**

category=2_p=0.968658268477645

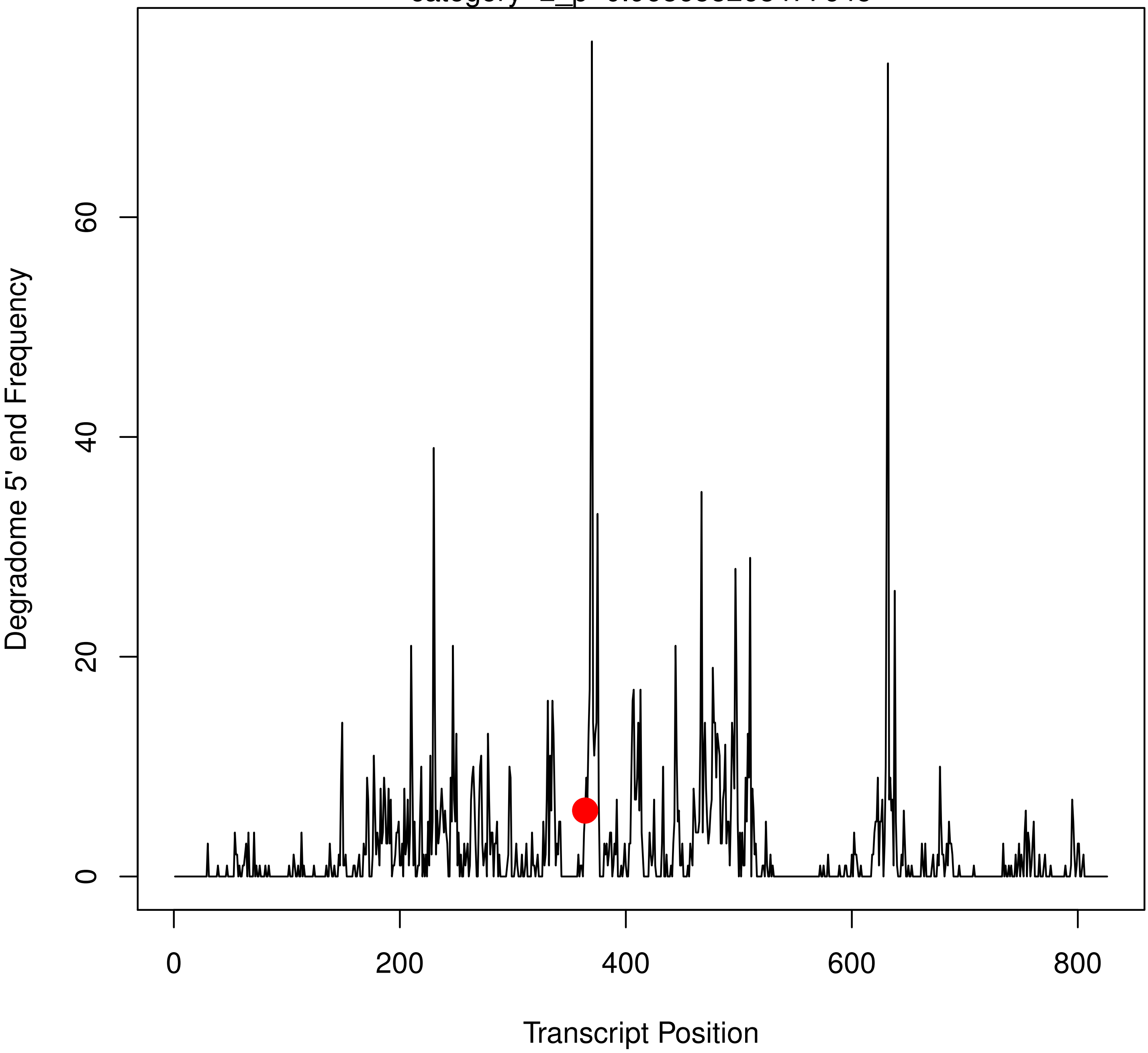

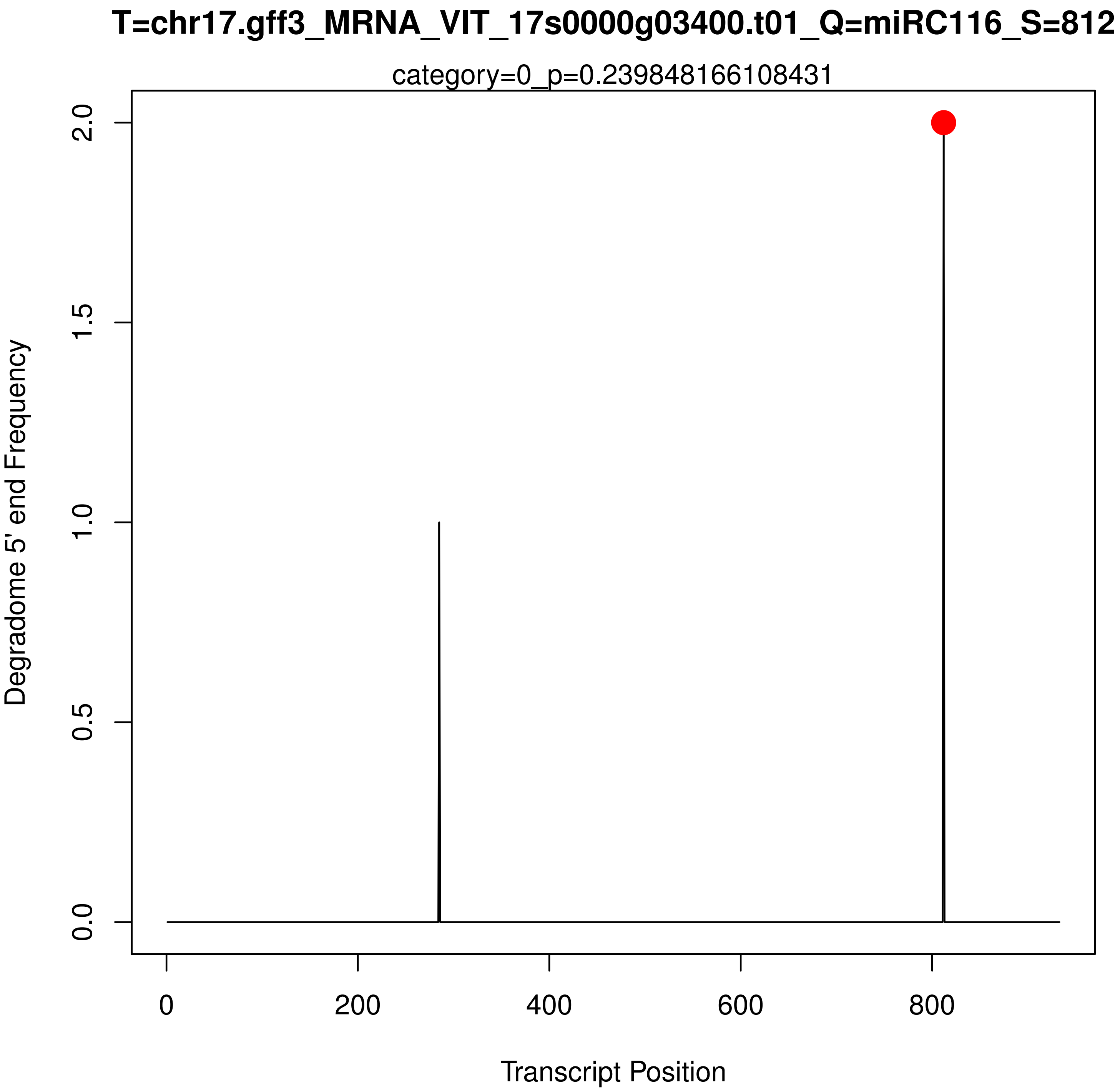
T=chr17.gff3_MRNA_VIT_17s0000g03400.t01_Q=miRC116_S=812
category=0_p=0.239848166108431
Degradome 5' end Frequency
0.0
0.5
1.0
1.5
2.0
0
200
400
600
800
Transcript Position

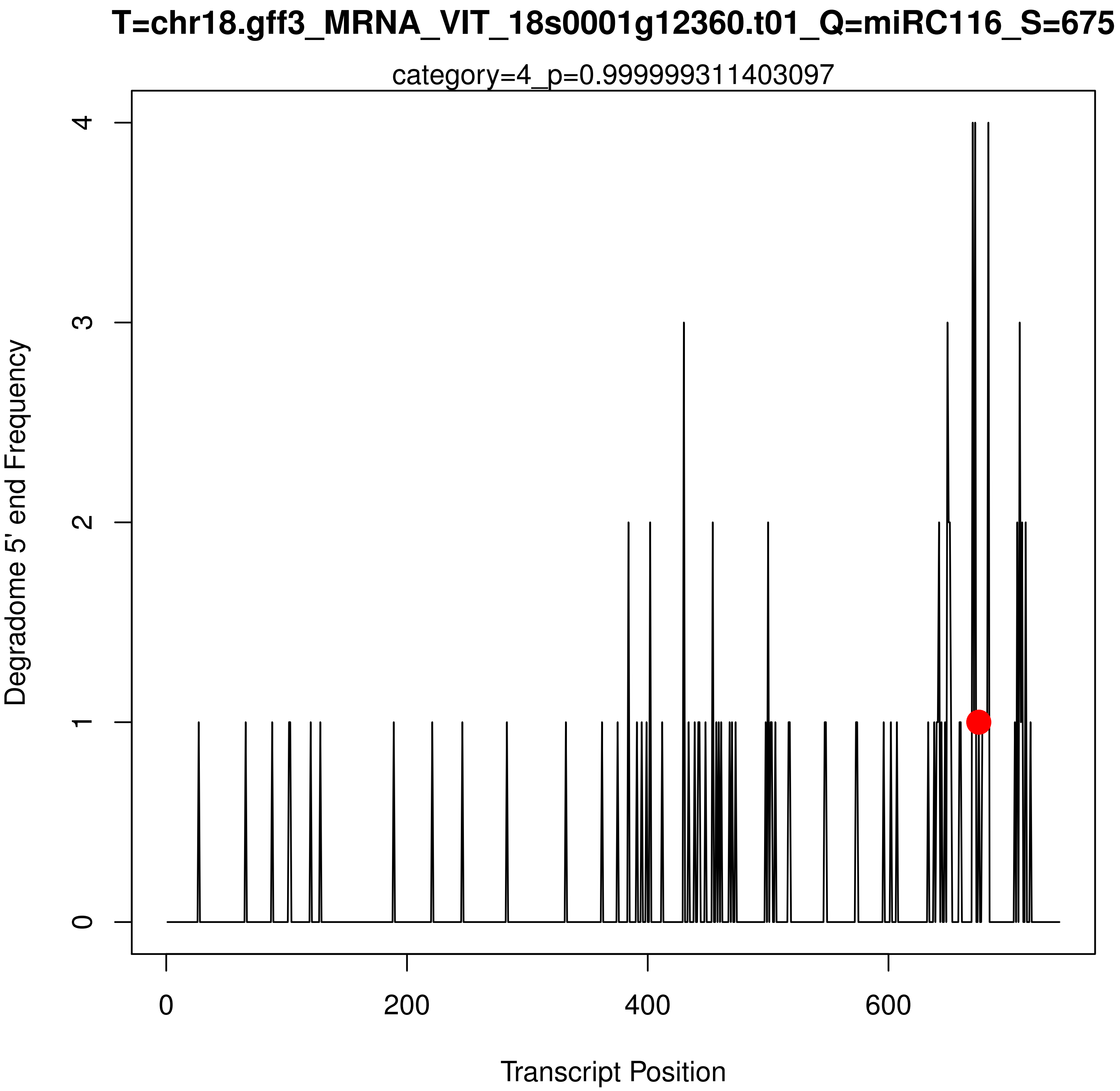
T=chr18.gff3_MRNA_VIT_18s0001g12360.t01_Q=miRC116_S=675
category=4_p=0.999999311403097
Degradome 5' end Frequency
0
1
2
3
4
0
200
400
600
Transcript Position

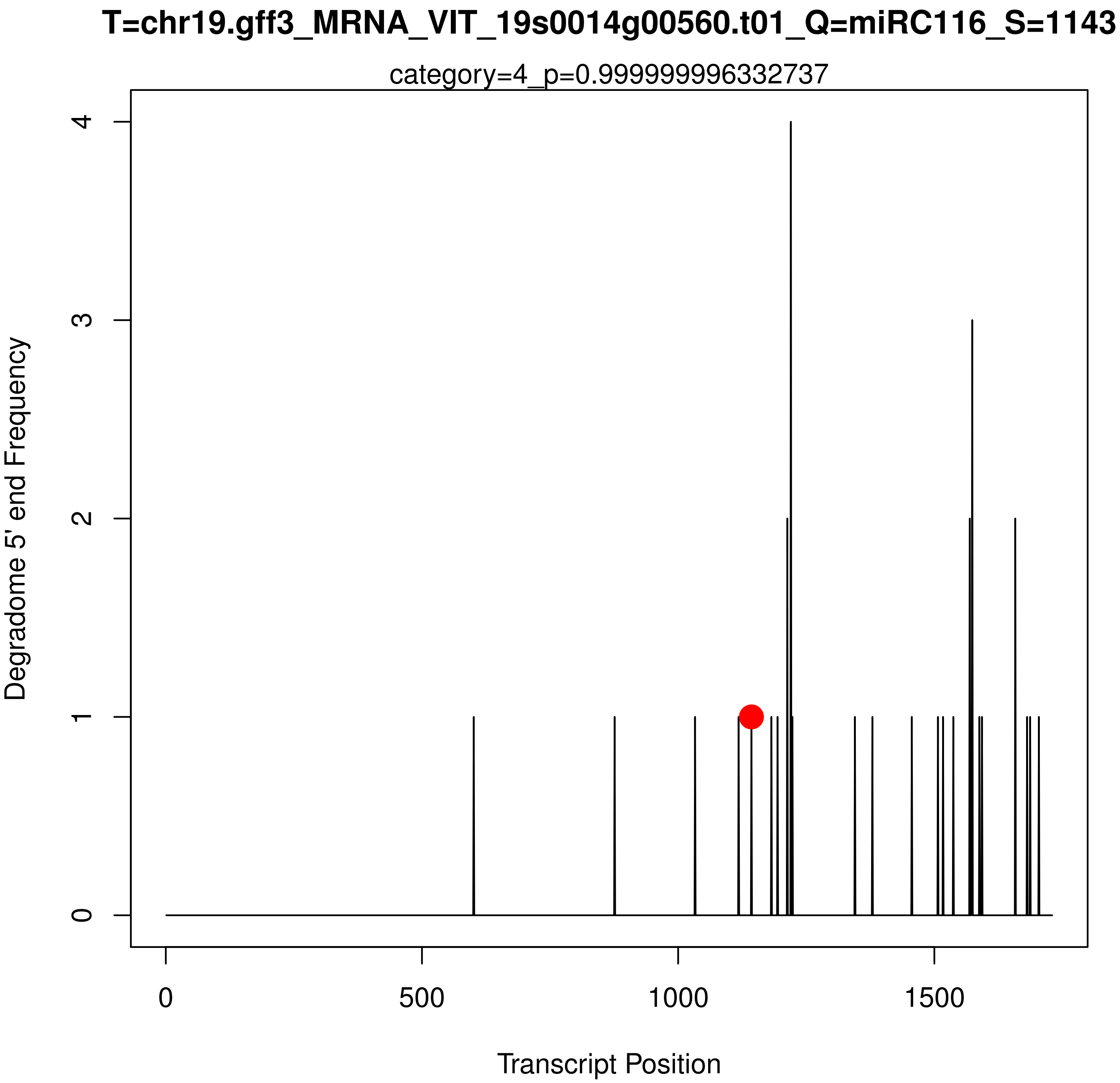
T=chr19.gff3_MRNA_VIT_19s0014g00560.t01_Q=miRC116_S=1143
category=4_p=0.999999996332737
Degradome 5' end Frequency
Transcript Position
0
1
2
3
4
0
500
1000
1500

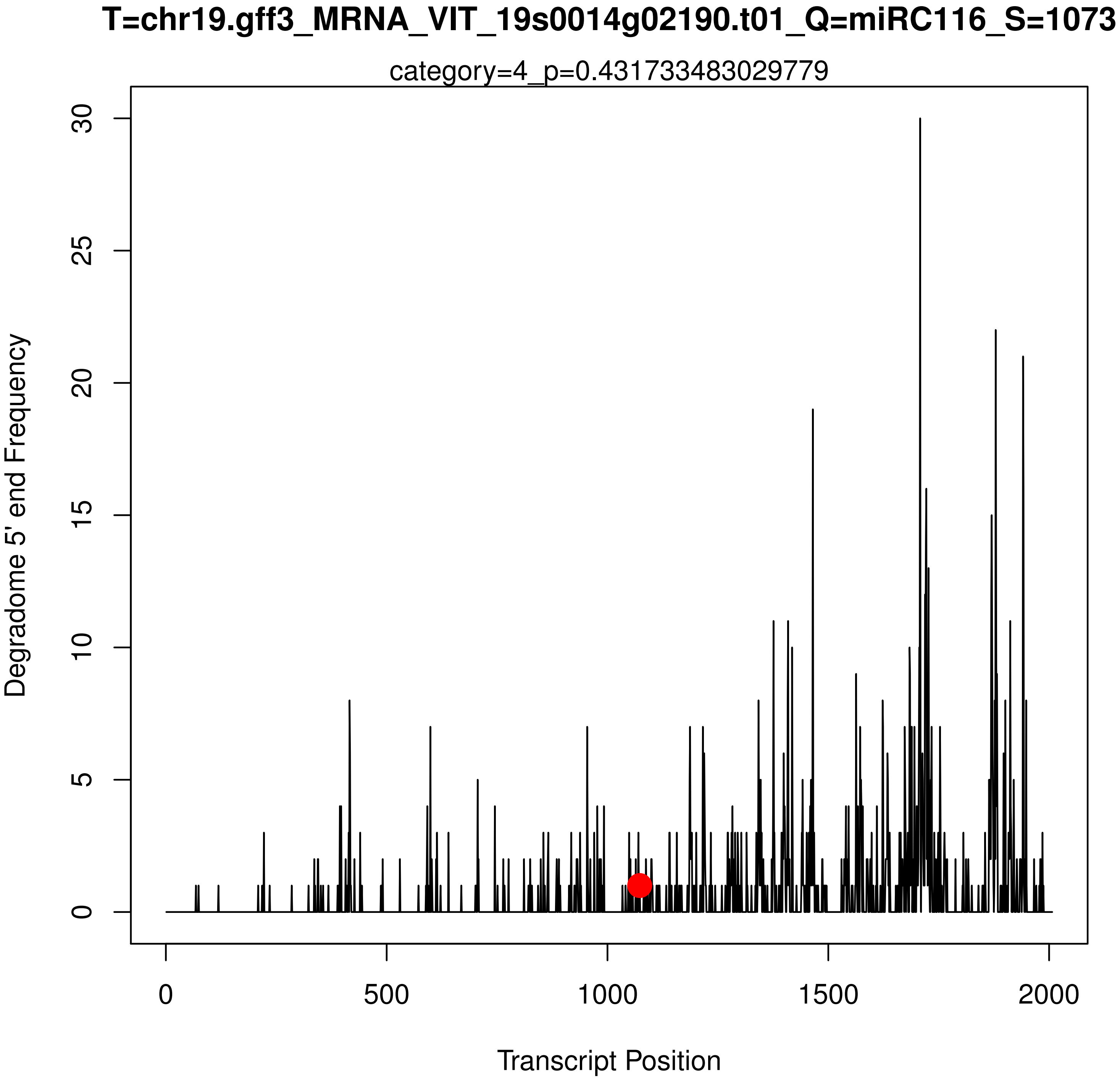
T=chr19.gff3_MRNA_VIT_19s0014g02190.t01_Q=miRC116_S=1073
category=4_p=0.431733483029779
Degradome 5' end Frequency
0
5
10
15
20
25
30
0
500
1000
1500
2000
Transcript Position

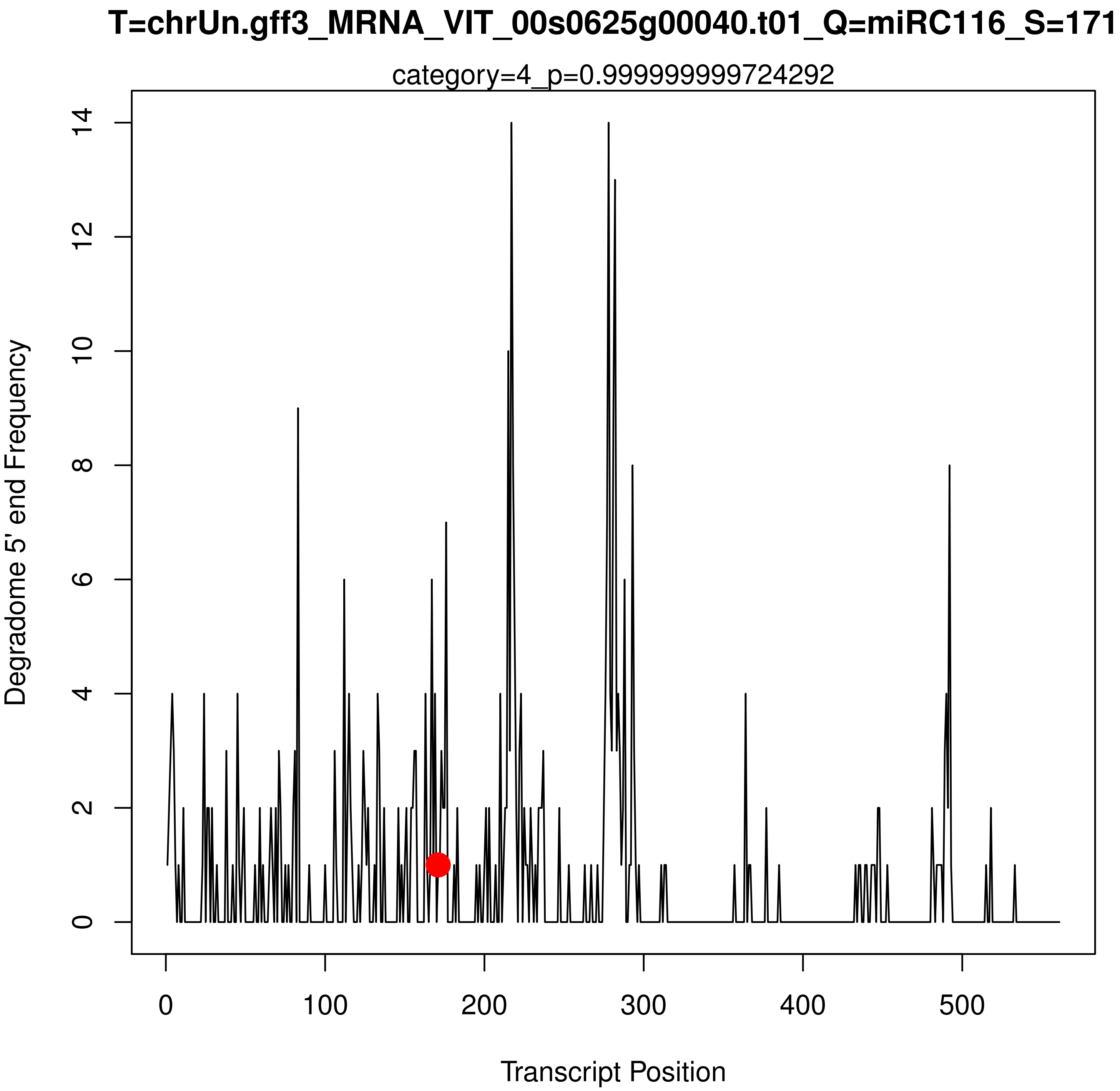
T=chrUn.gff3_MRNA_VIT_00s0625g00040.t01_Q=miRC116_S=171
category=4_p=0.999999999724292
Degradome 5' end Frequency
0
2
4
6
8
10
12
14
0
100
200
300
400
500
Transcript Position

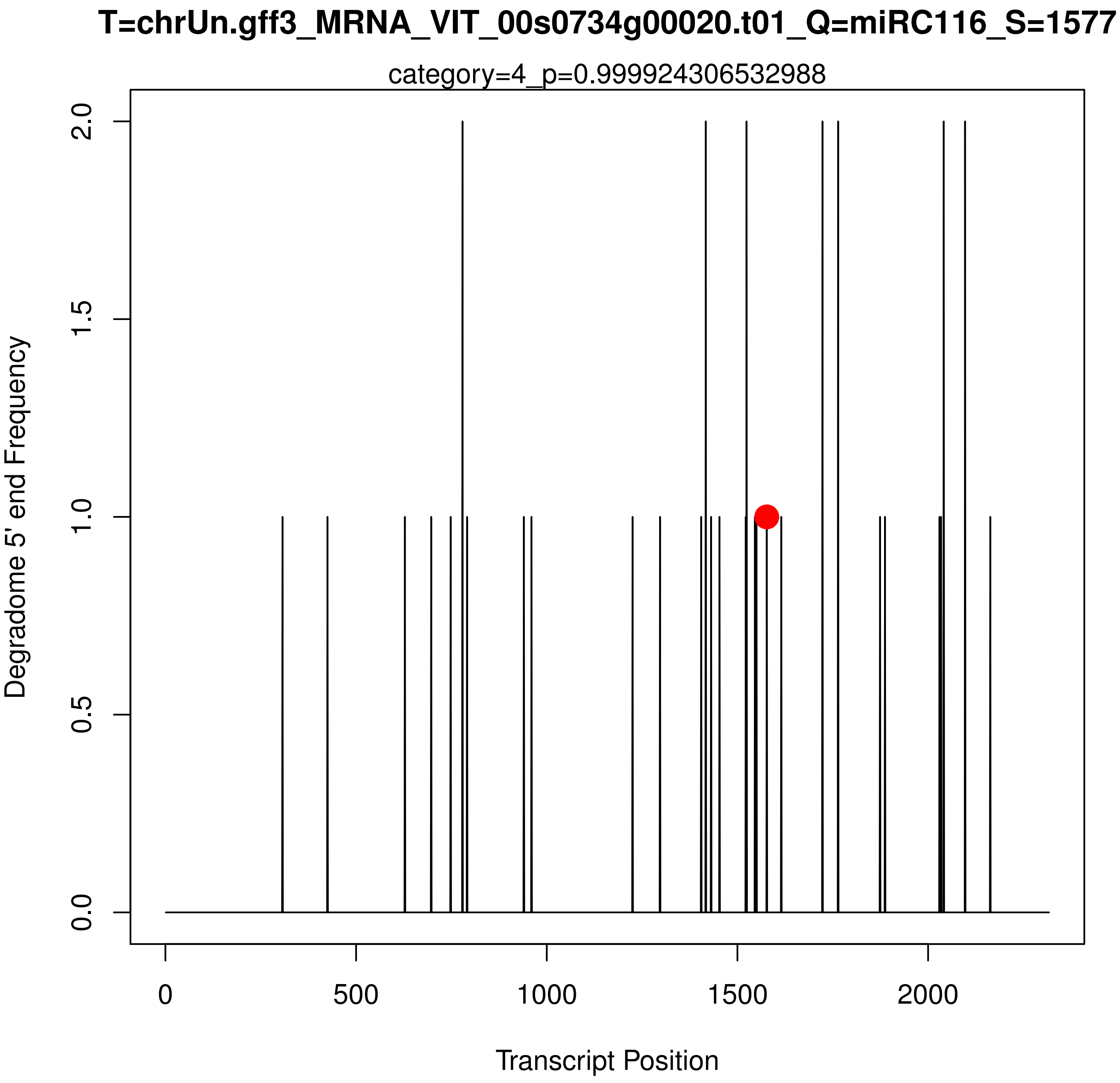
T=chrUn.gff3_MRNA_VIT_00s0734g00020.t01_Q=miRC116_S=1577
category=4_p=0.999924306532988
Degradome 5' end Frequency
0.0
0.5
1.0
1.5
2.0
0
500
1000
1500
2000
Transcript Position

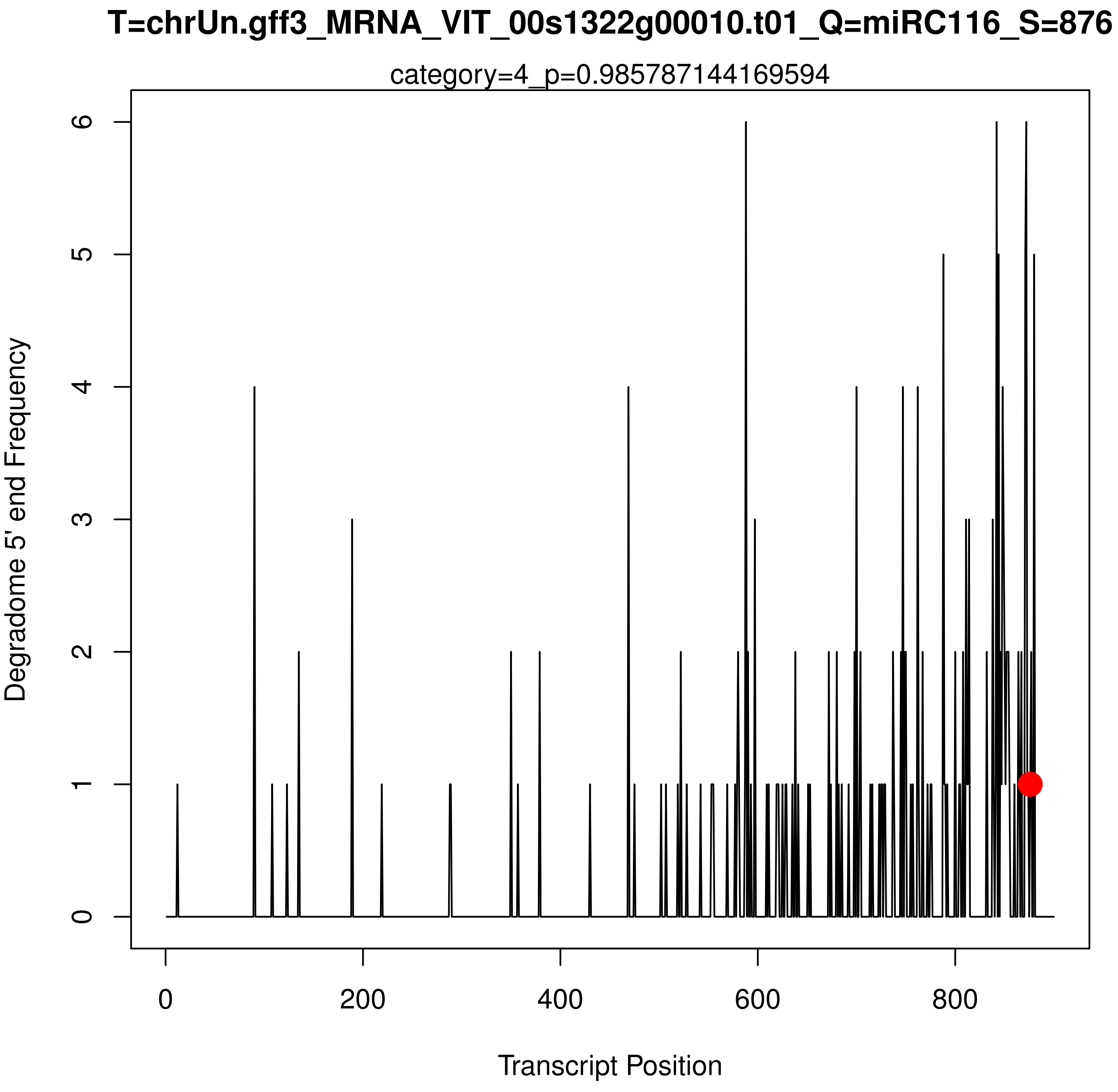

T=chrUn.gff3_MRNA_VIT_00s1322g00010.t01_Q=miRC116_S=876
category=4_p=0.985787144169594
Degradome 5' end Frequency
Transcript Position
0
1
2
3
4
5
6
0
200
400
600
800

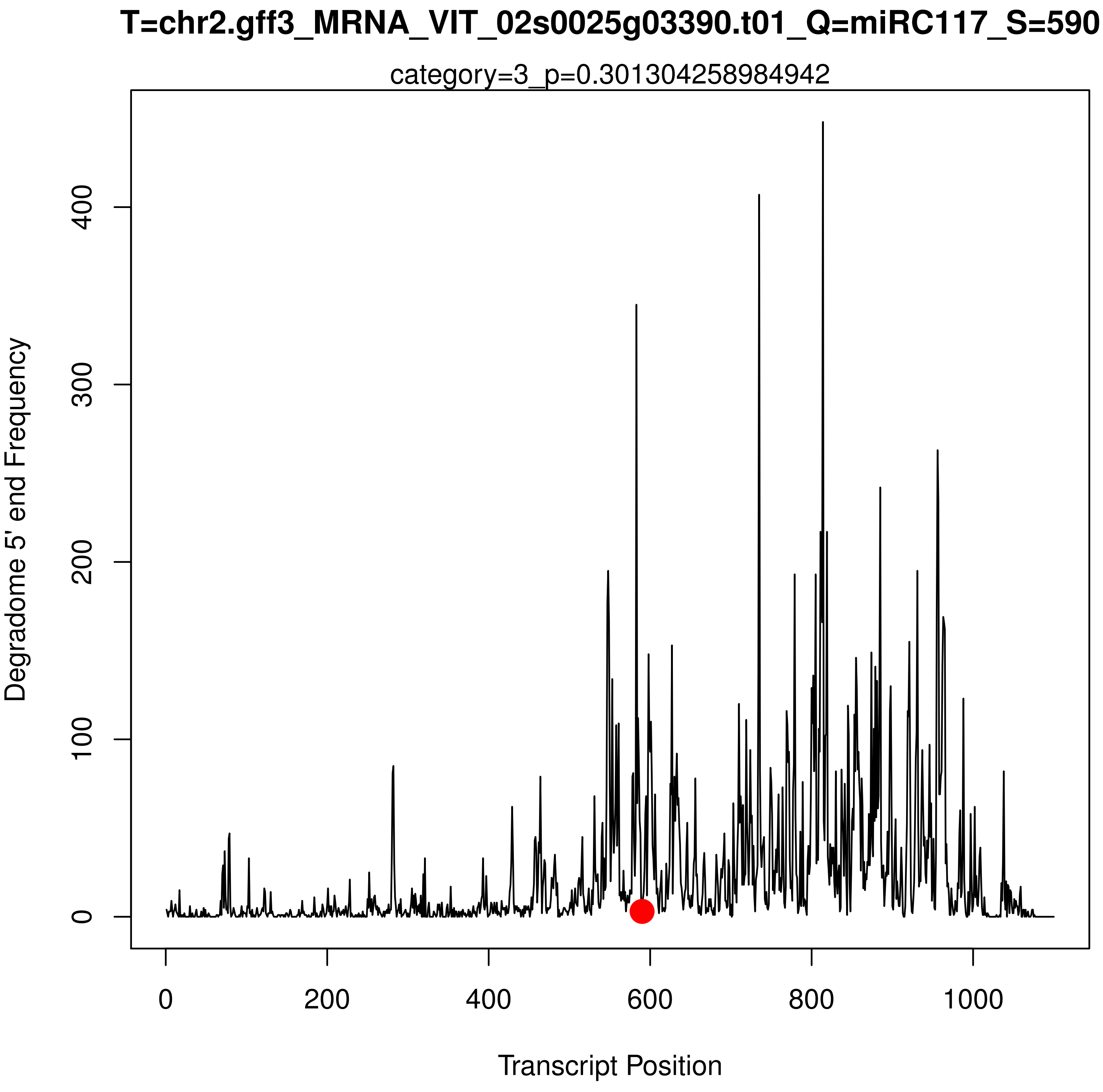
T=chr2.gff3_MRNA_VIT_02s0025g03390.t01_Q=miRC117_S=590
category=3_p=0.301304258984942
Degradome 5' end Frequency
0
100
200
300
400
0
200
400
600
800
1000
Transcript Position

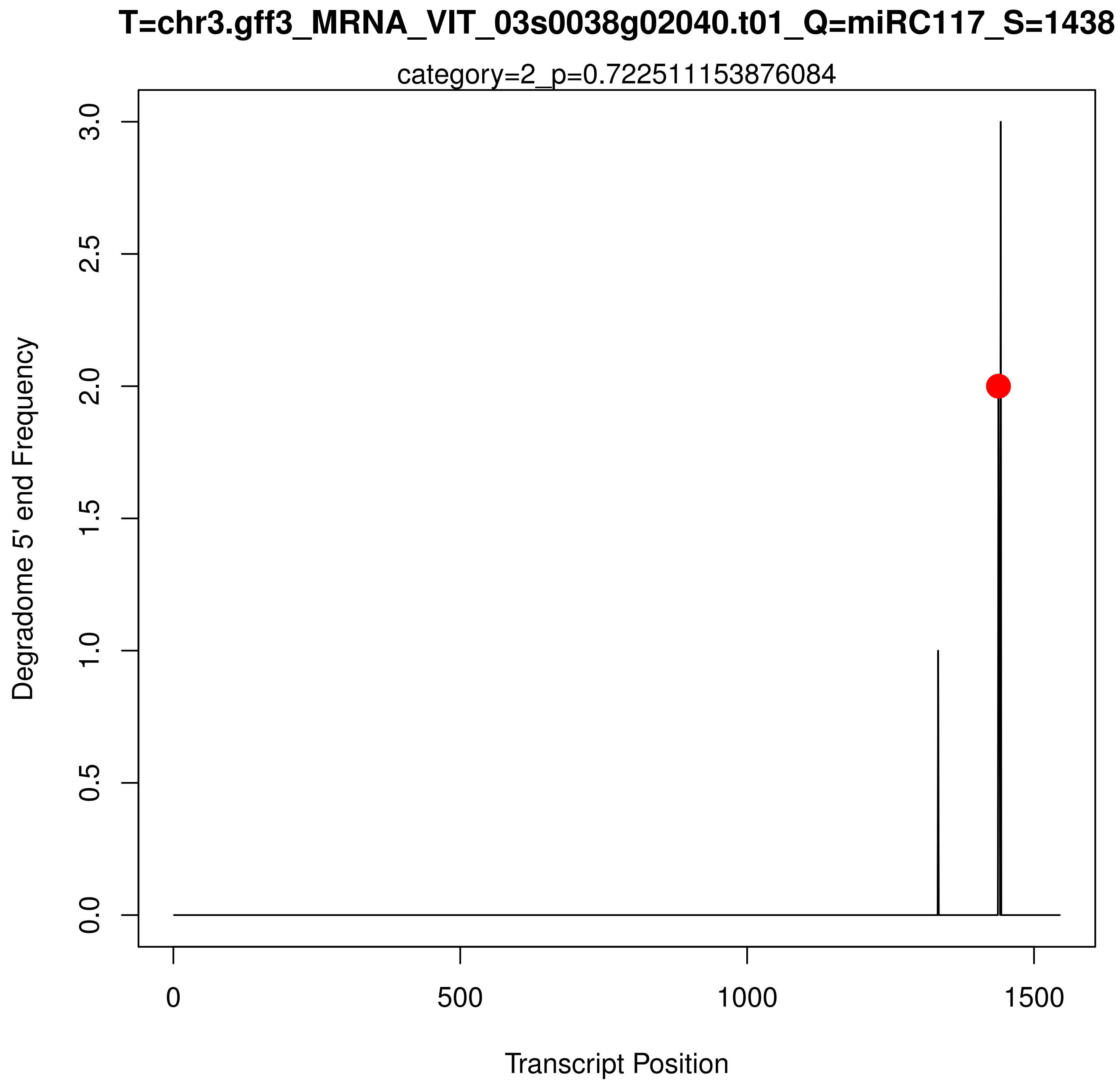
T=chr3.gff3_MRNA_VIT_03s0038g02040.t01_Q=miRC117_S=1438
category=2_p=0.722511153876084
Degradome 5' end Frequency
0.0
0.5
1.0
1.5
2.0
2.5
3.0
0
500
1000
1500
Transcript Position

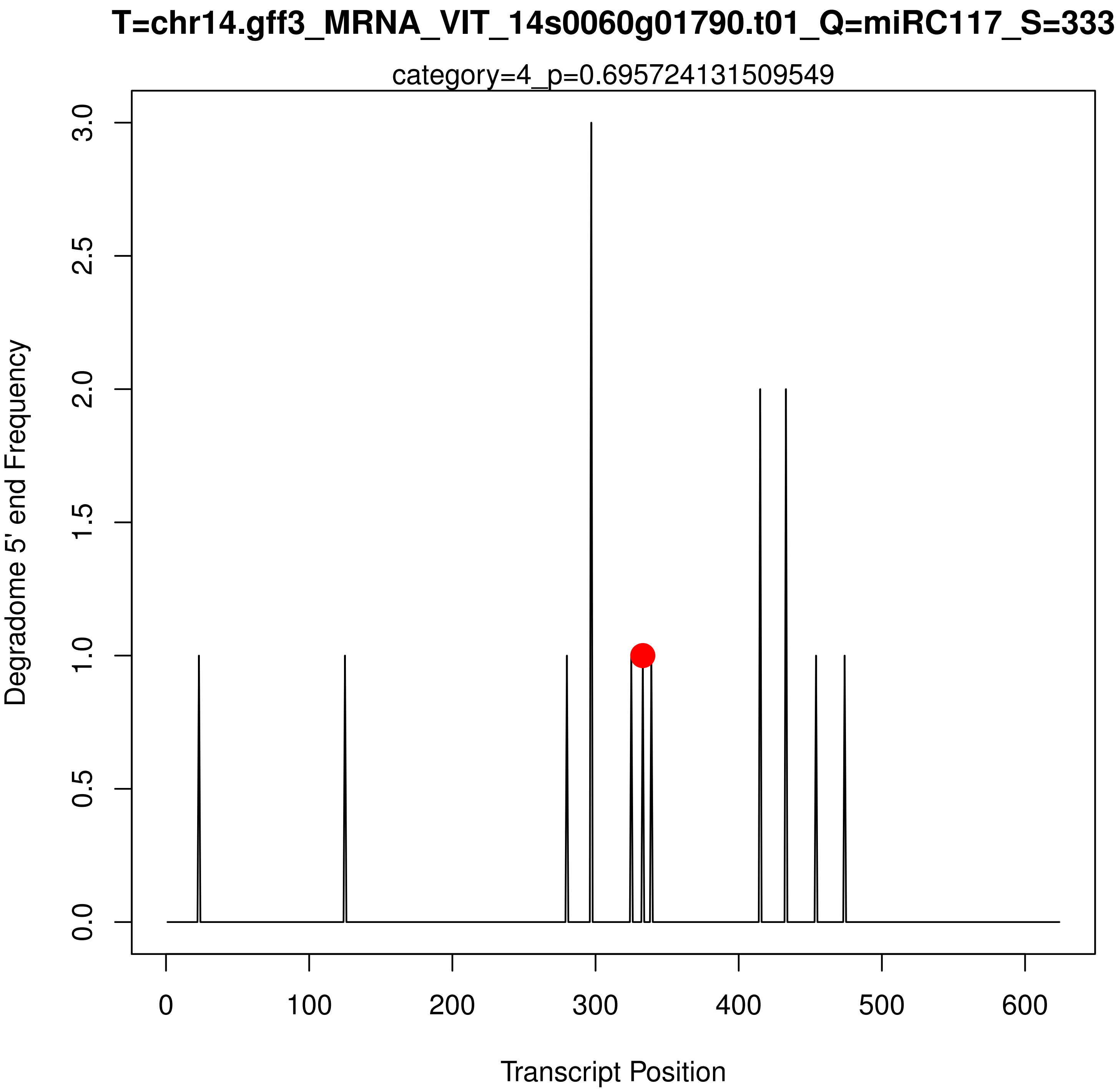
T=chr14.gff3_MRNA_VIT_14s0060g01790.t01_Q=miRC117_S=333
category=4_p=0.695724131509549
Degradome 5' end Frequency
3.0
2.5
2.0
1.5
1.0
0.5
0.0
0
100
200
300
400
500
600
Transcript Position

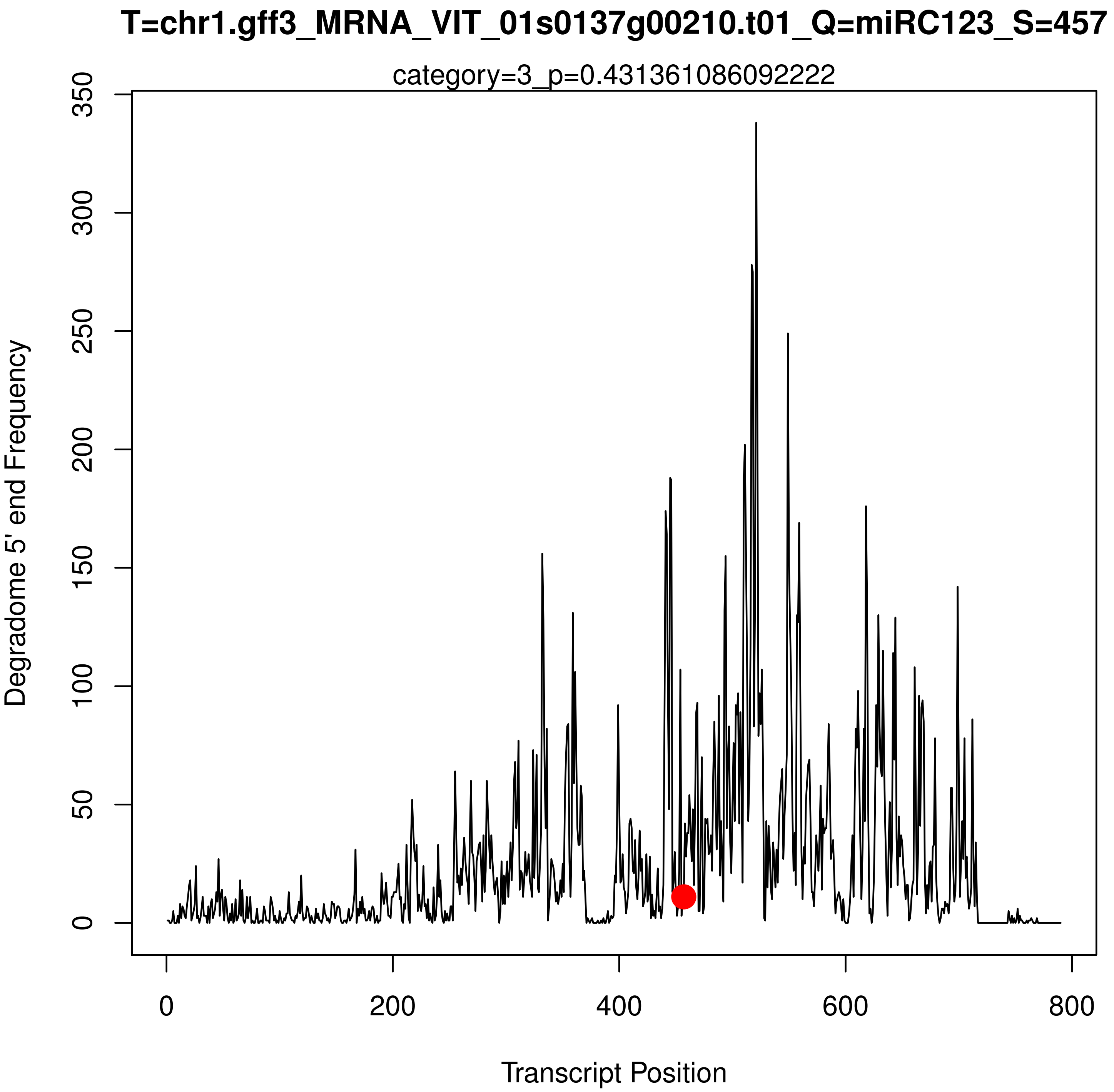

T=chr1.gff3_MRNA_VIT_01s0137g00210.t01_Q=miRC123_S=457
category=3_p=0.431361086092222
Degradome 5' end Frequency
0
50
100
150
200
250
300
350
0
200
400
600
800
Transcript Position

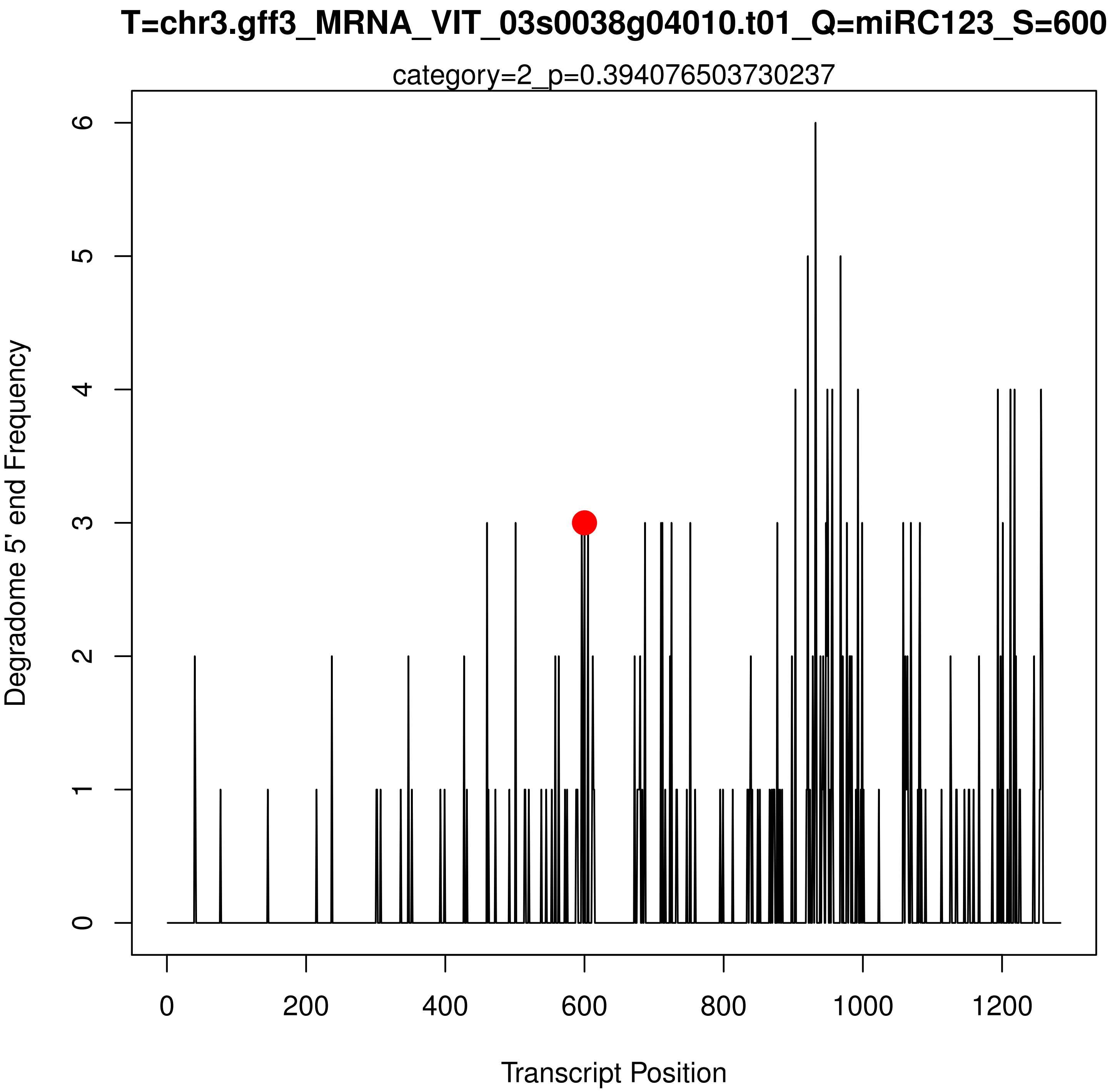

T=chr3.gff3_MRNA_VIT_03s0038g04010.t01_Q=miRC123_S=600
category=2_p=0.394076503730237
Degradome 5' end Frequency
0
1
2
3
4
5
6
0
200
400
600
800
1000
1200
Transcript Position

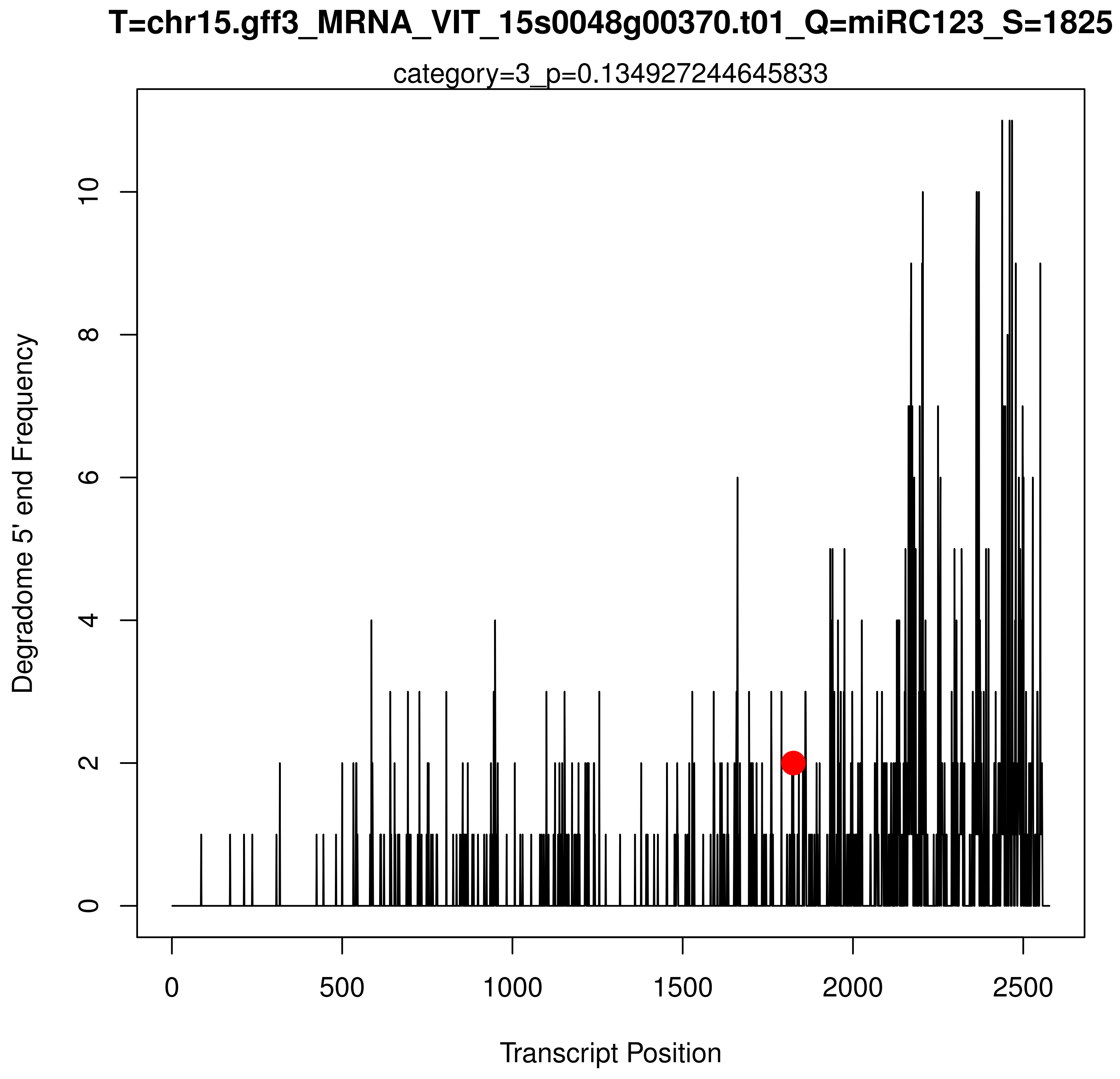
T=chr15.gff3_MRNA_VIT_15s0048g00370.t01_Q=miRC123_S=1825
category=3_p=0.134927244645833
Degradome 5' end Frequency
0
2
4
6
8
10
0
500
1000
1500
2000
2500
Transcript Position

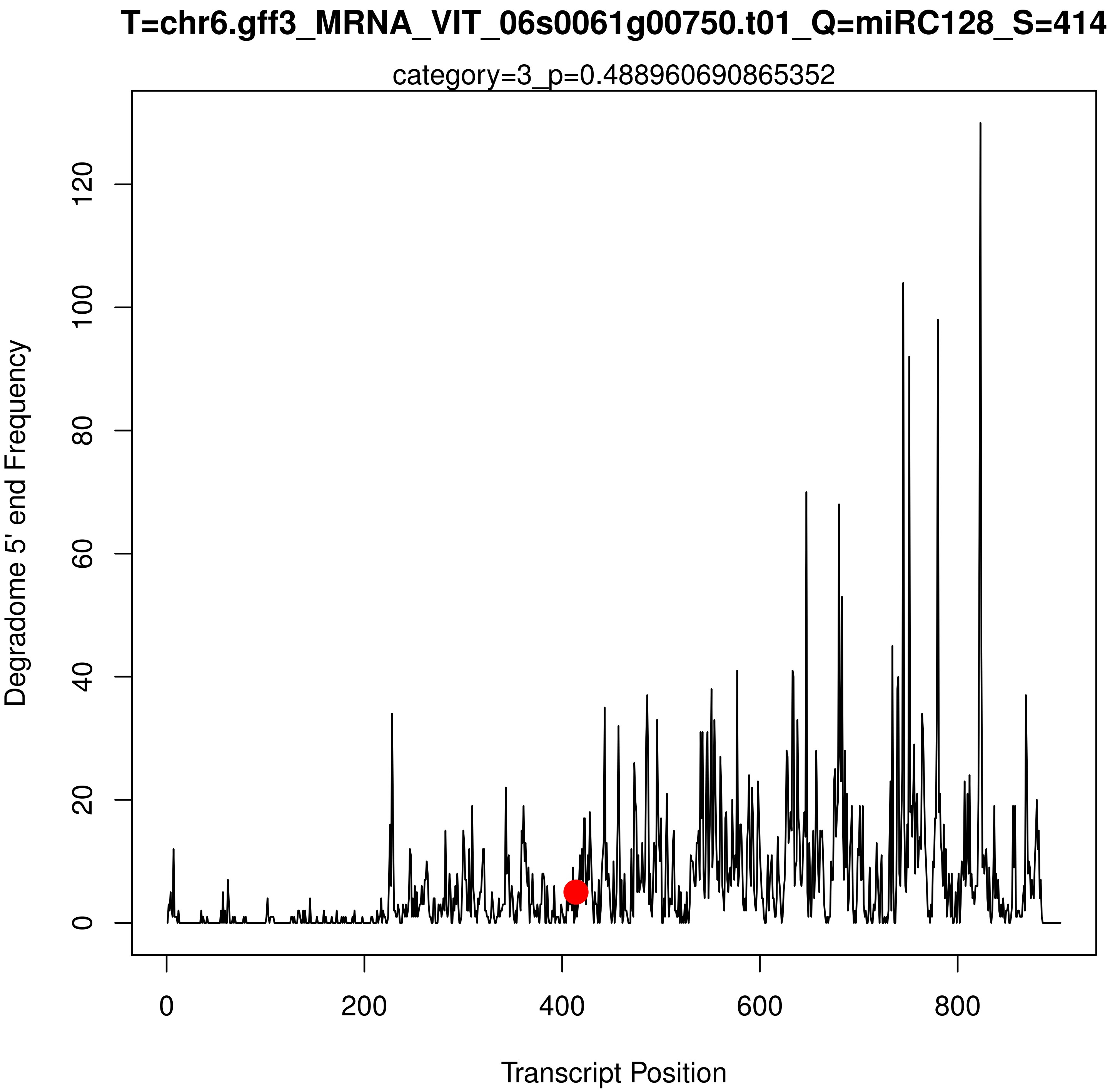
T=chr6.gff3_MRNA_VIT_06s0061g00750.t01_Q=miRC128_S=414
category=3_p=0.488960690865352
Degradome 5' end Frequency
Transcript Position
0
20
40
60
80
100
120
0
200
400
600
800

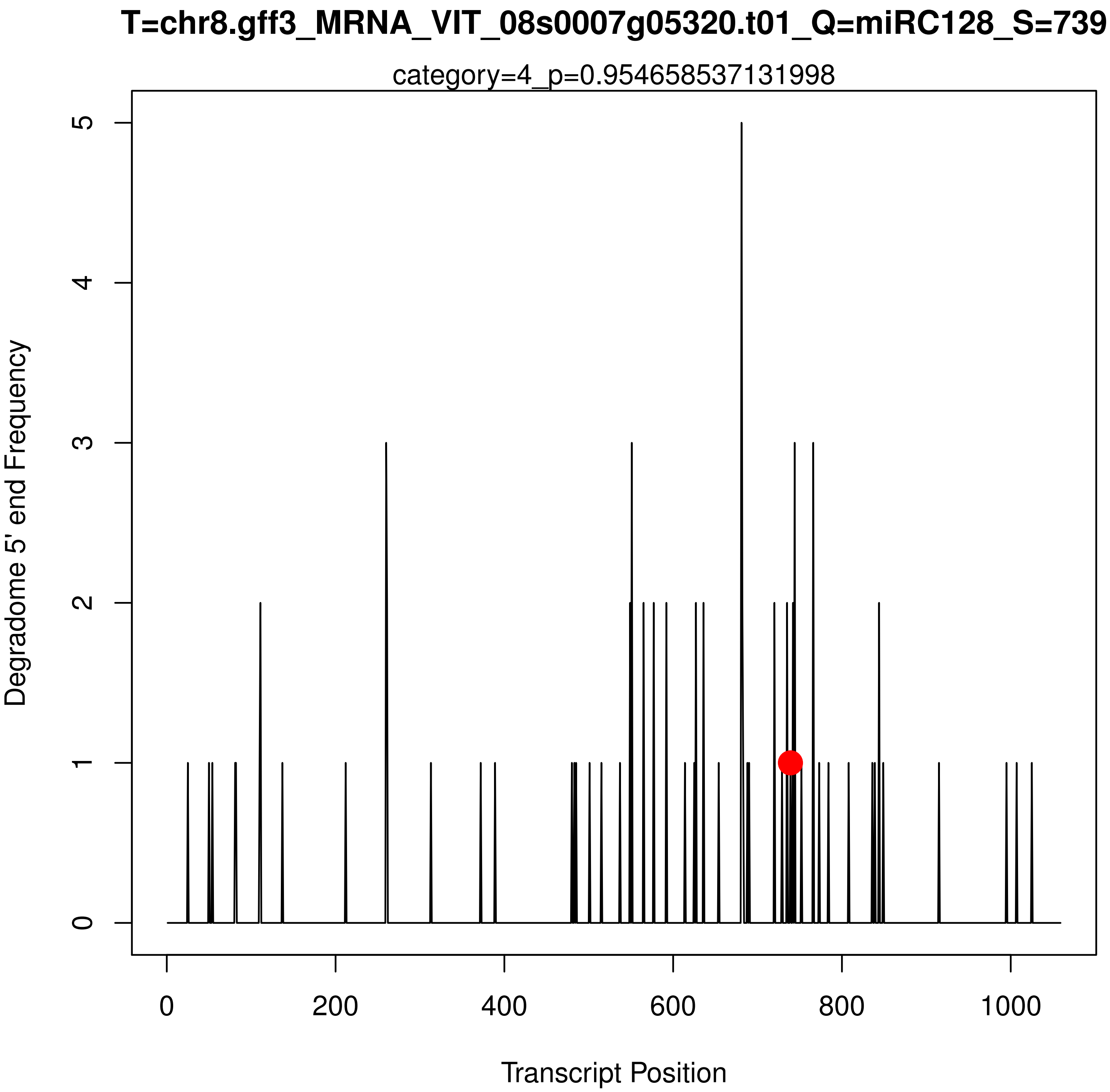

T=chr8.gff3_MRNA_VIT_08s0007g05320.t01_Q=miRC128_S=739
category=4_p=0.954658537131998
Degradome 5' end Frequency
Transcript Position
0
1
2
3
4
5
0
200
400
600
800
1000

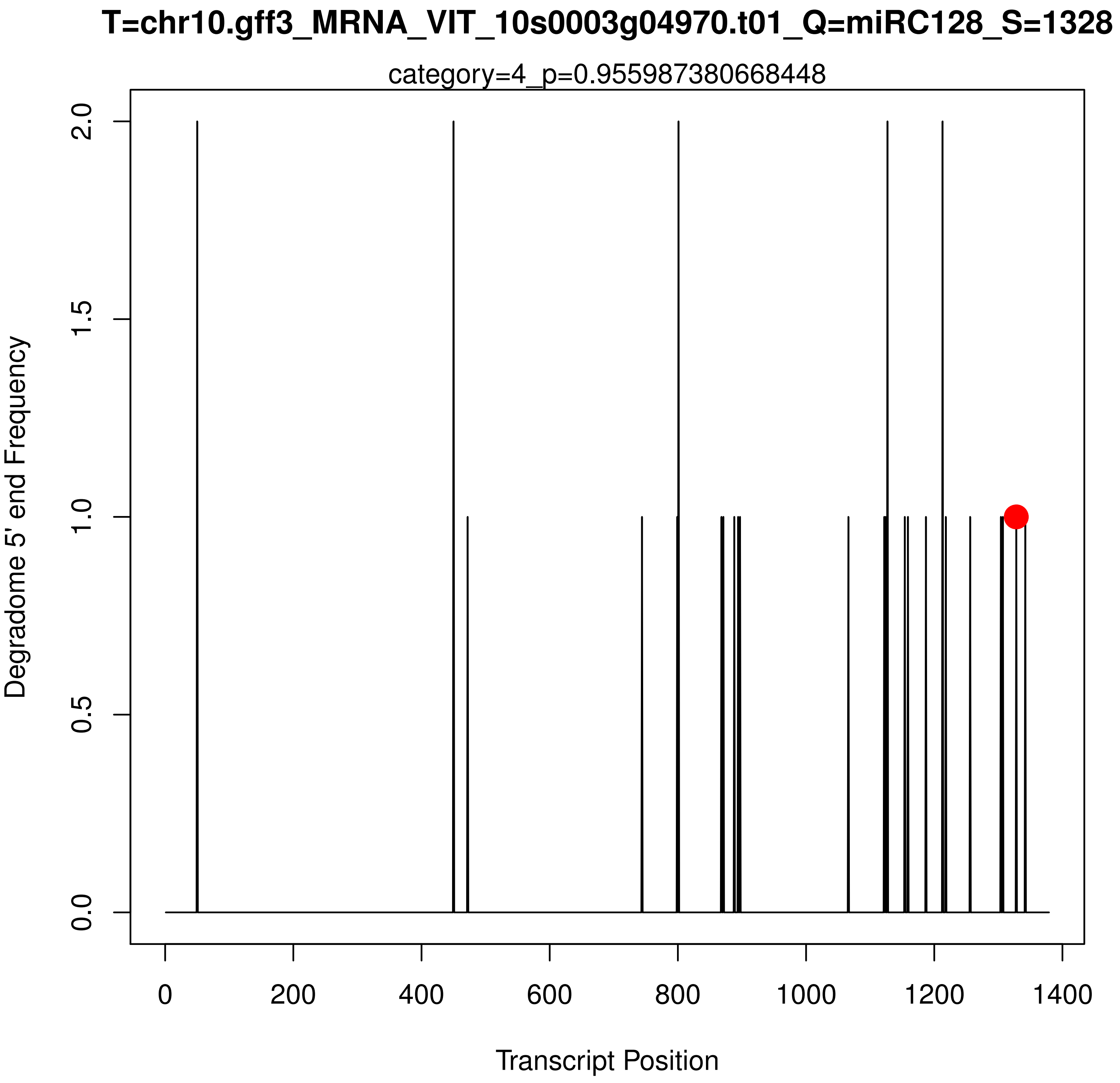
T=chr10.gff3_MRNA_VIT_10s0003g04970.t01_Q=miRC128_S=1328
category=4_p=0.955987380668448
Degradome 5' end Frequency
0.0
0.5
1.0
1.5
2.0
0
200
400
600
800
1000
1200
1400
Transcript Position

**T=chr13.gff3_MRNA_VIT_13s0019g01980.t01_Q= miRC129 _S=932**

category=2_p=0.632856716668226

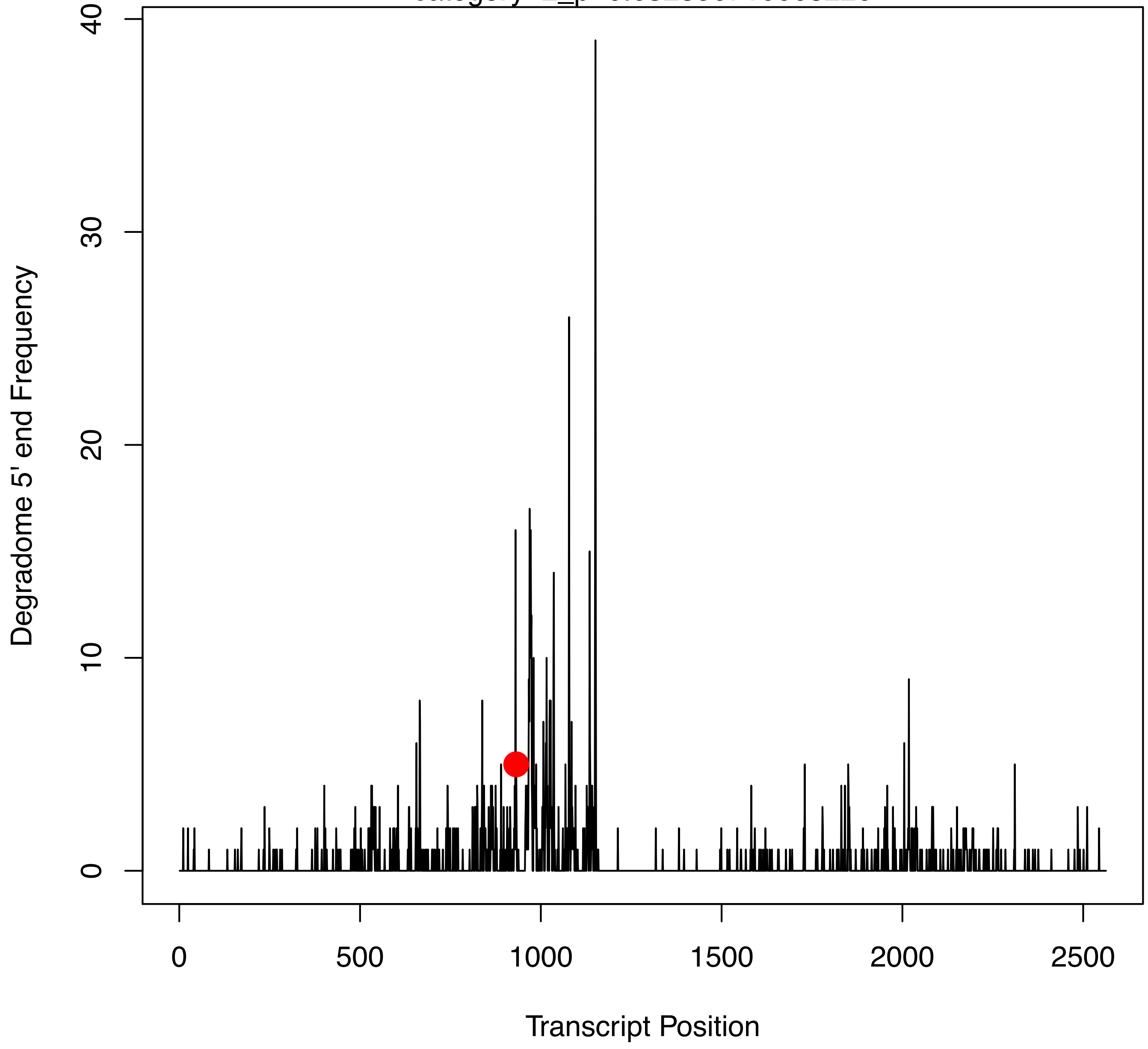

**T=chr5.gff3_MRNA_VIT_05s0020g00380.t01_Q= miRC129 _S=1219**

category=2_p=0.793340203256816

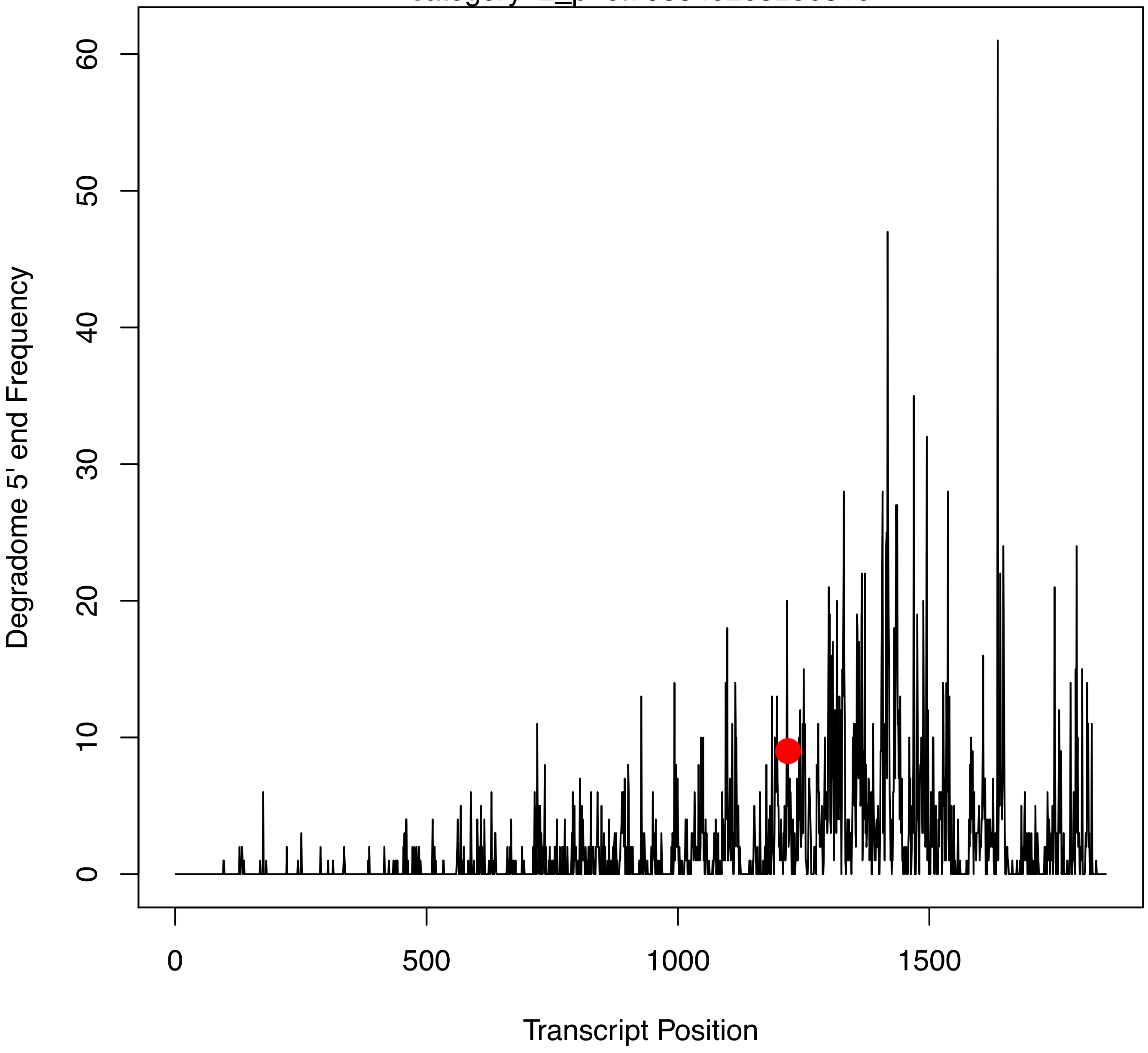

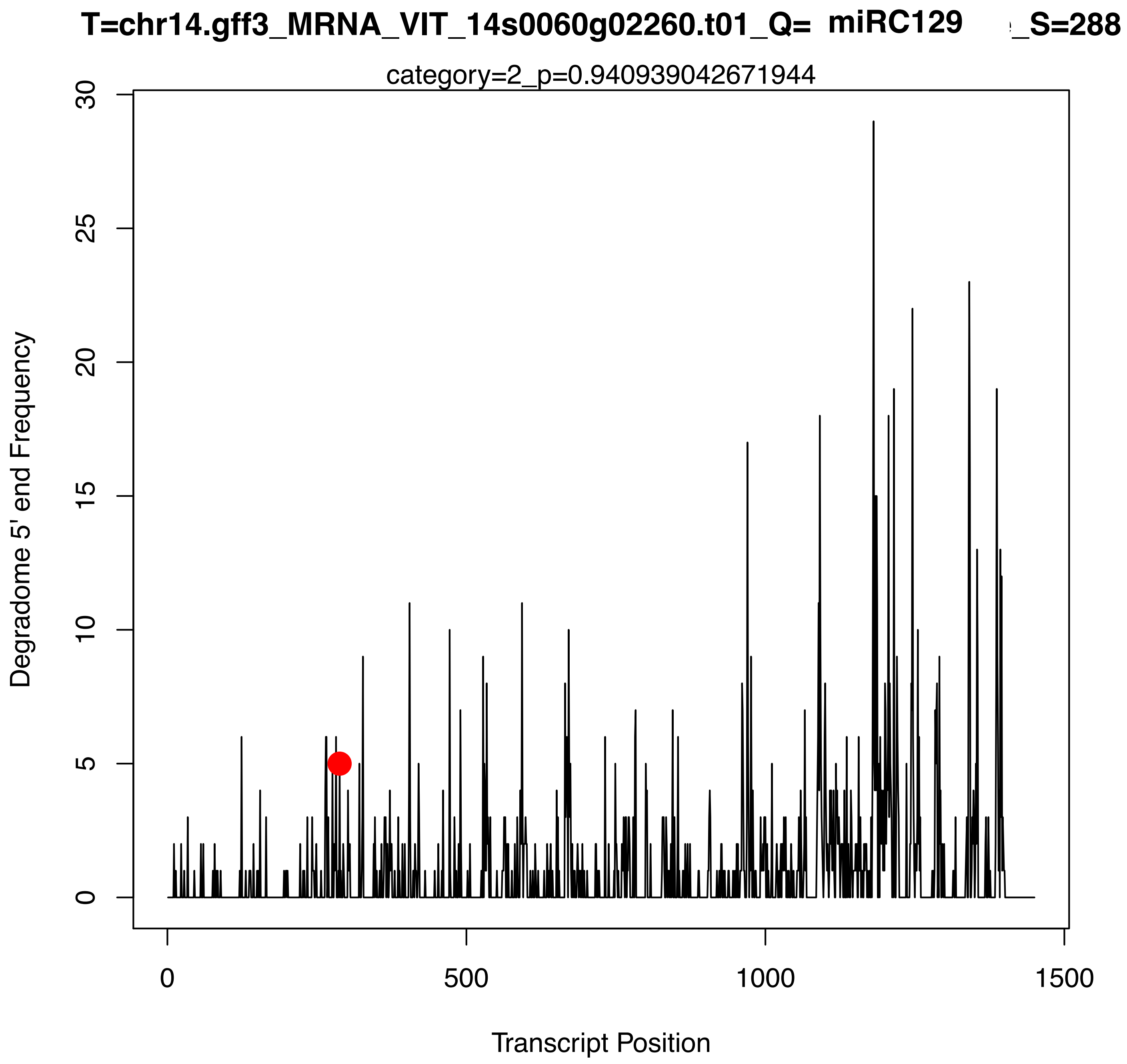
T=chr14.gff3_MRNA_VIT_14s0060g02260.t01_Q= miRC129 _S=288
category=2_p=0.940939042671944
Degradome 5' end Frequency
0
5
10
15
20
25
30
Transcript Position
0
500
1000
1500

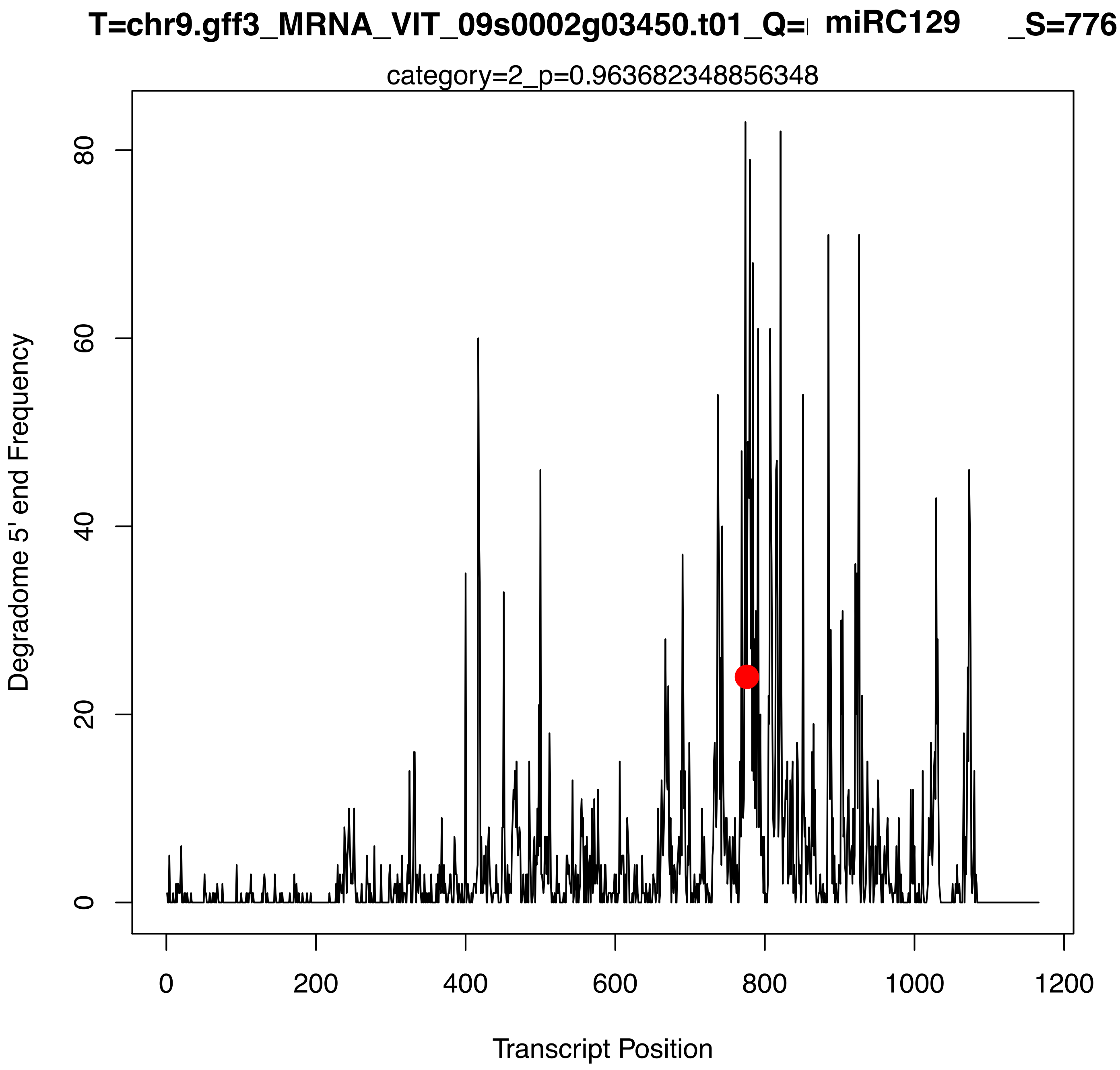
T=chr9.gff3_MRNA_VIT_09s0002g03450.t01_Q= miRC129 _S=776
category=2_p=0.963682348856348
Degradome 5' end Frequency
0
20
40
60
80
0
200
400
600
800
1000
1200
Transcript Position

**T=chrUn.gff3_MRNA_VIT_00s0361g00080.t01_Q= miRC129 _S=1846**

category=2_p=0.966755416778674

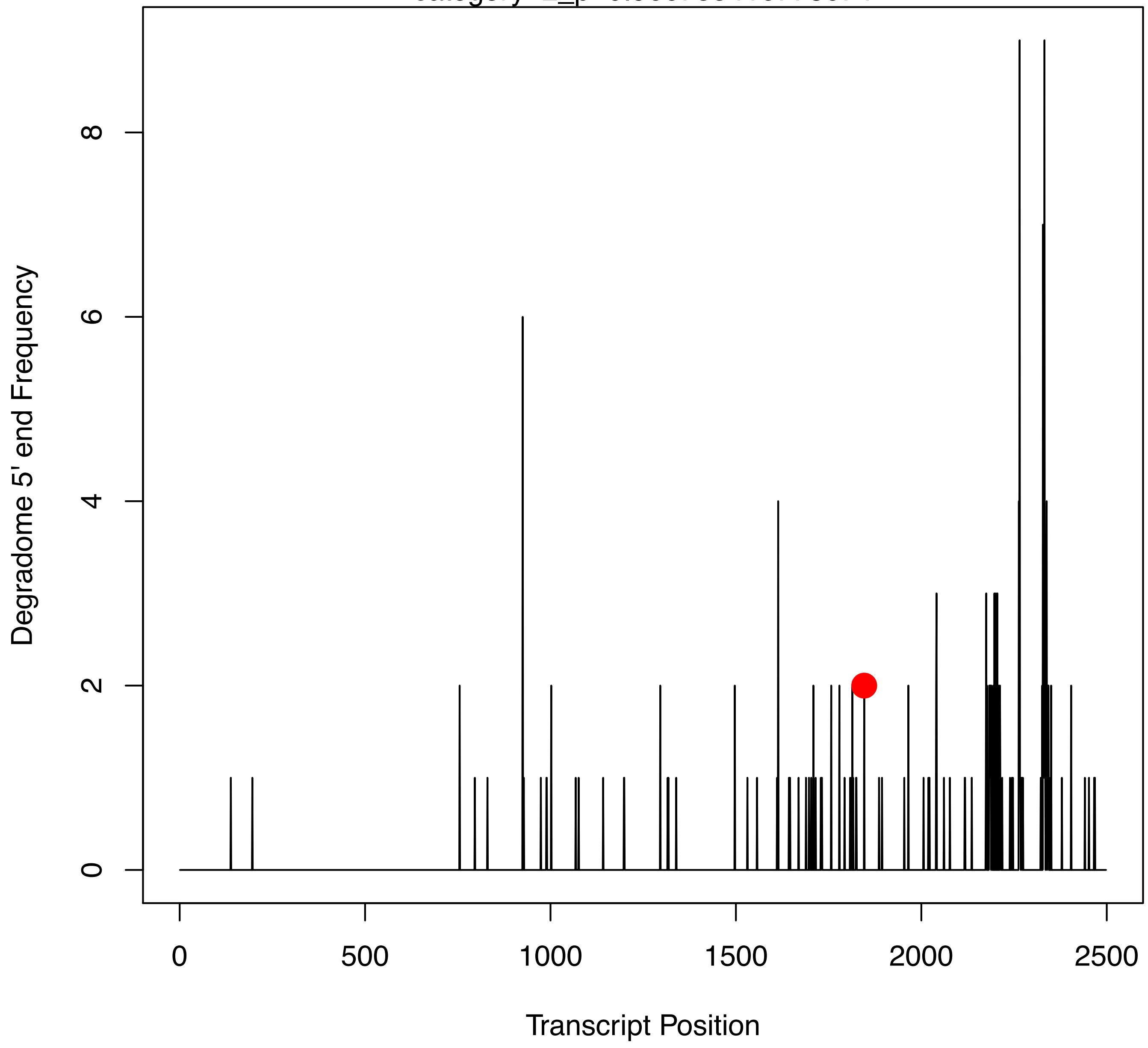

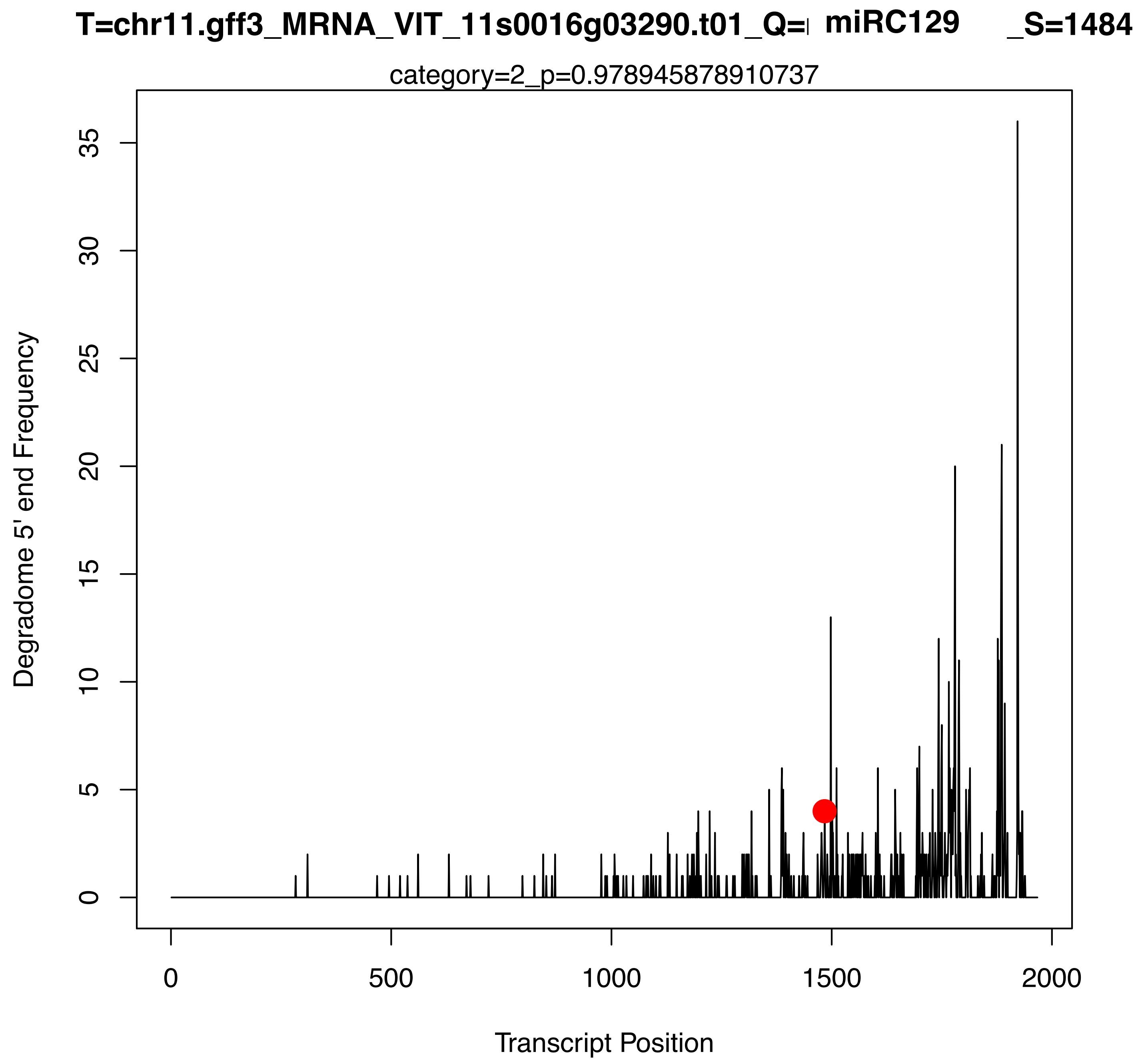
T=chr11.gff3_MRNA_VIT_11s0016g03290.t01_Q=l miRC129 _S=1484
category=2_p=0.978945878910737
Degradome 5' end Frequency
0
5
10
15
20
25
30
35
0
500
1000
1500
2000
Transcript Position

**T=chr5.gff3_MRNA_VIT_05s0020g03180.t01_Q=I miRC129 _S=665**

category=2_p=0.995648959615086

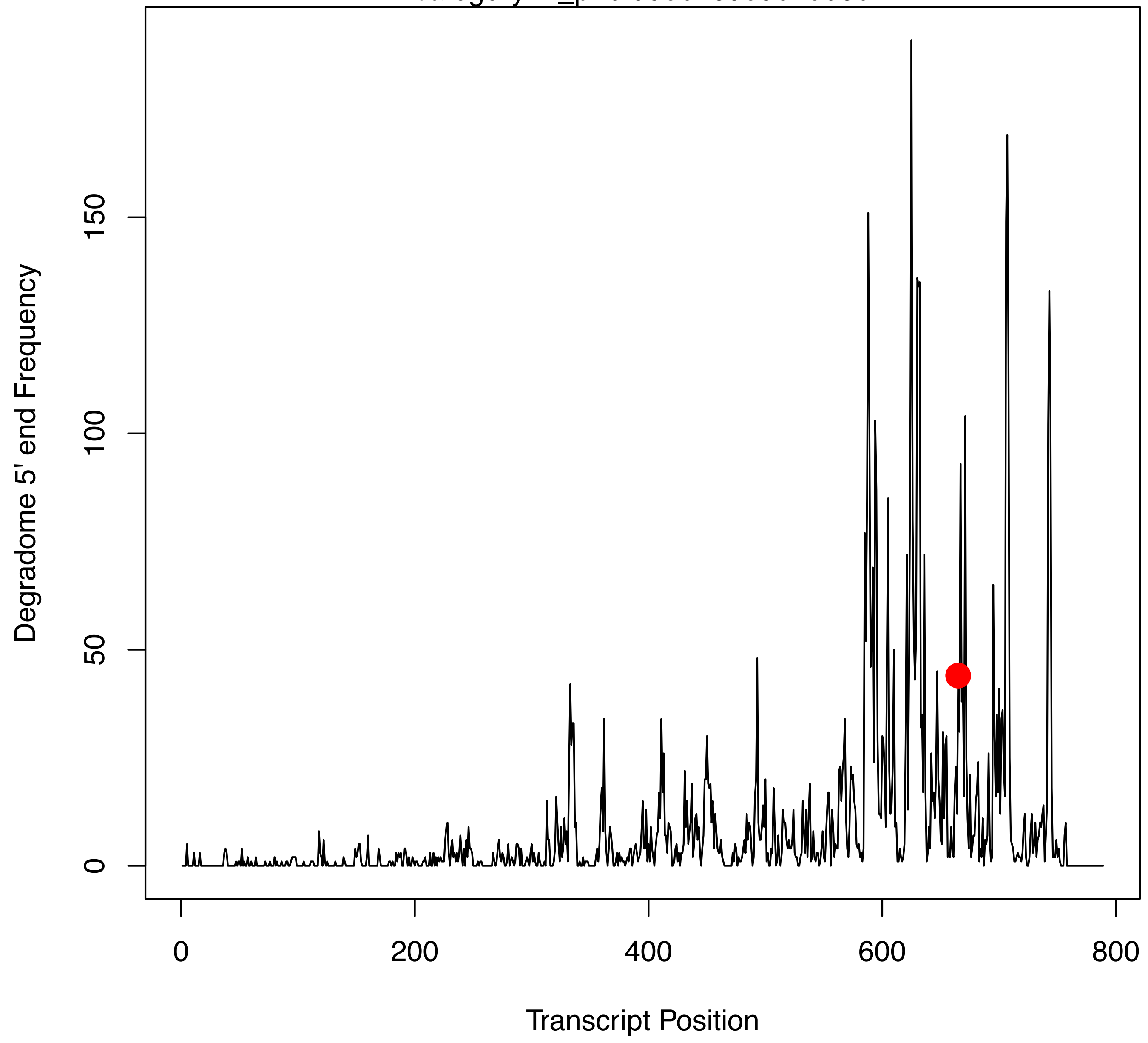

**T=chr17.gff3_MRNA_VIT_17s0000g09370.t01_Q=| miRC129 _S=1187**

category=3_p=0.322302288587418

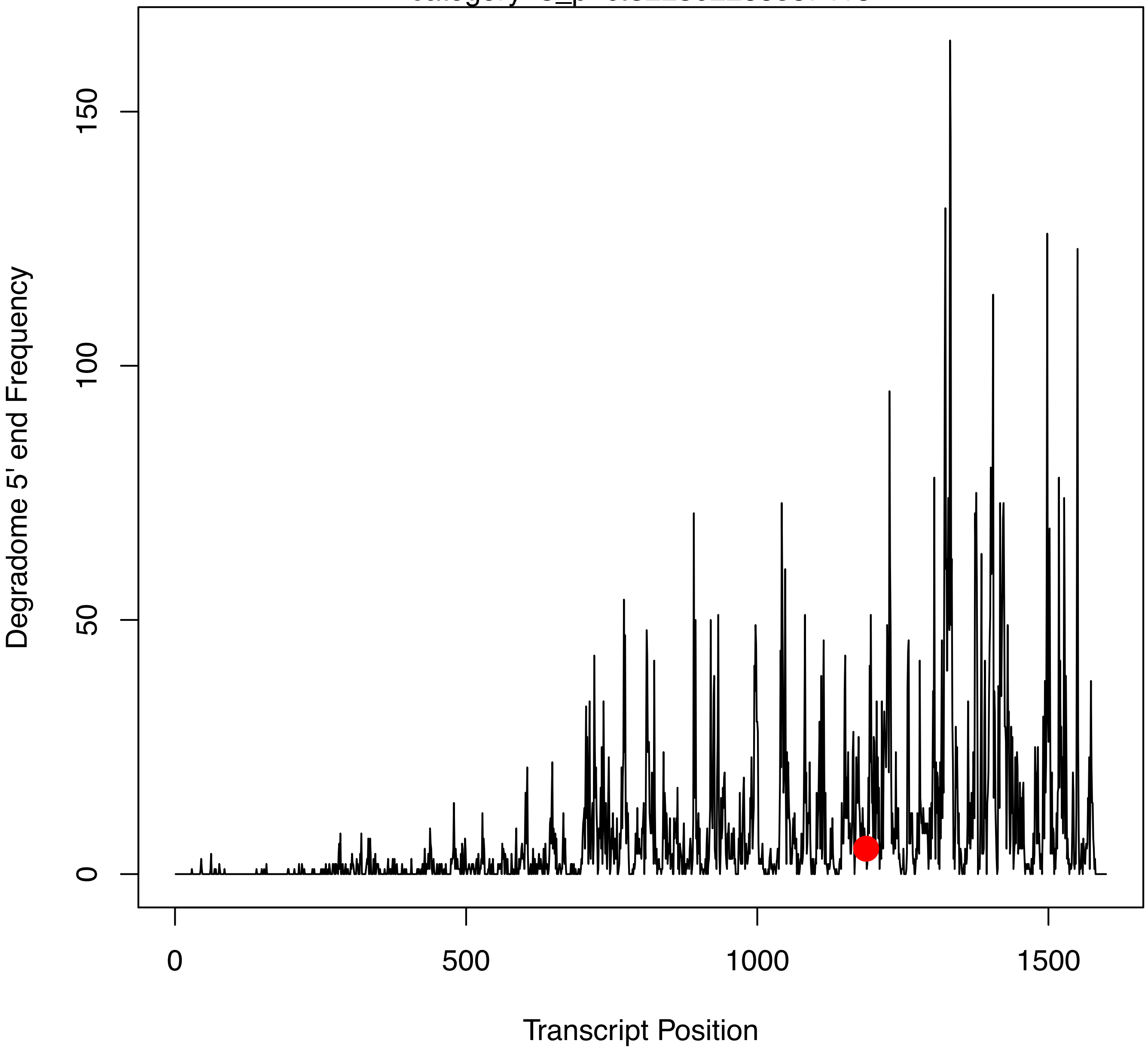

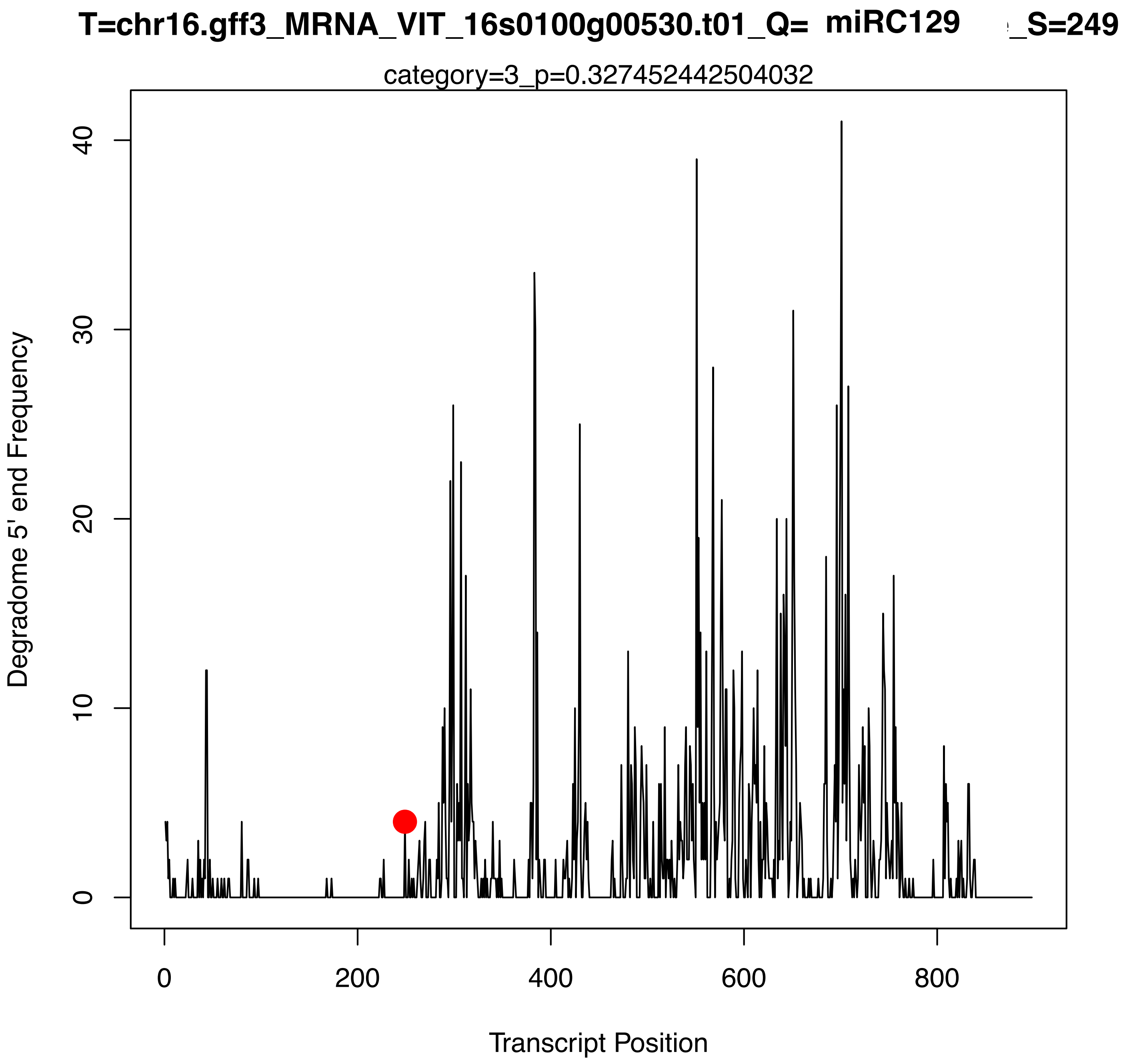

T=chr16.gff3_MRNA_VIT_16s0100g00530.t01_Q= miRC129 _S=249
category=3_p=0.327452442504032
Degradome 5' end Frequency
0
10
20
30
40
0
200
400
600
800
Transcript Position

**T=chr3.gff3_MRNA_VIT_03s0063g02450.t01_Q=miRC129 _S=2334**

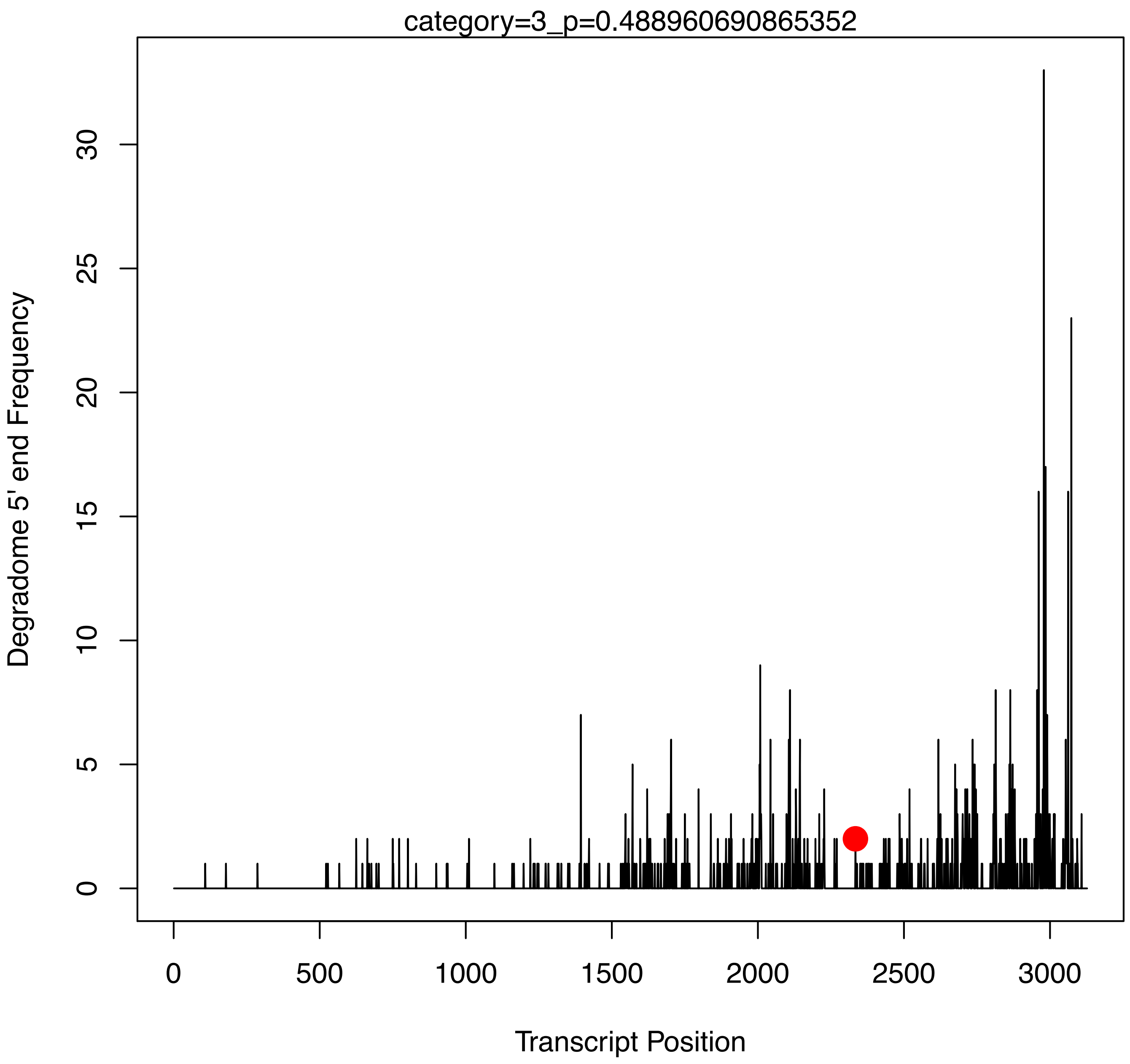

**T=chr6.gff3_MRNA_VIT_06s0004g06860.t01_Q= miRC129 _S=5117**

category=3_p=0.508085967944197

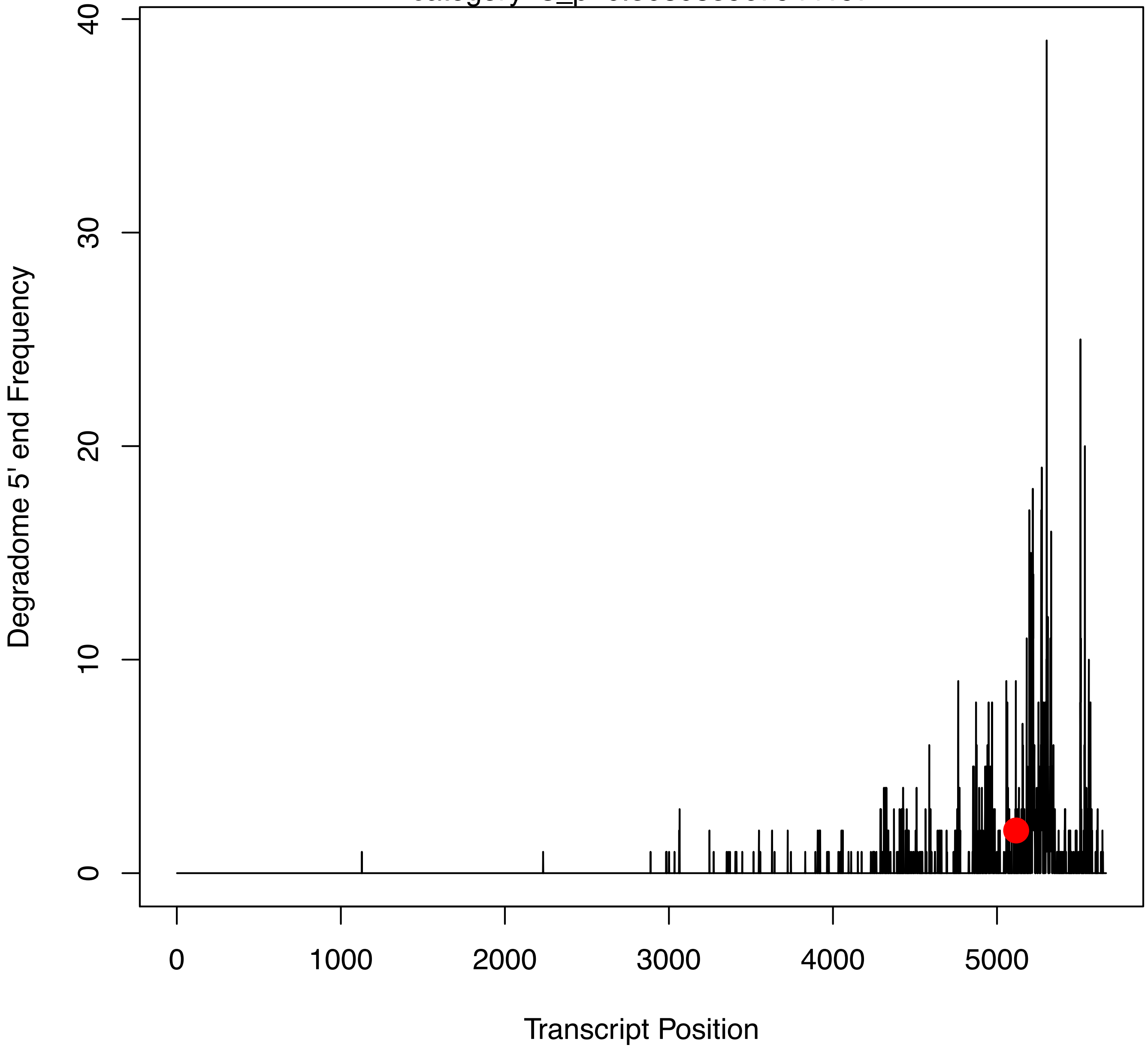

**T=chr13.gff3_MRNA_VIT_13s0019g01900.t01_Q= miRC129 _S=589**

category=3_p=0.526495495340377

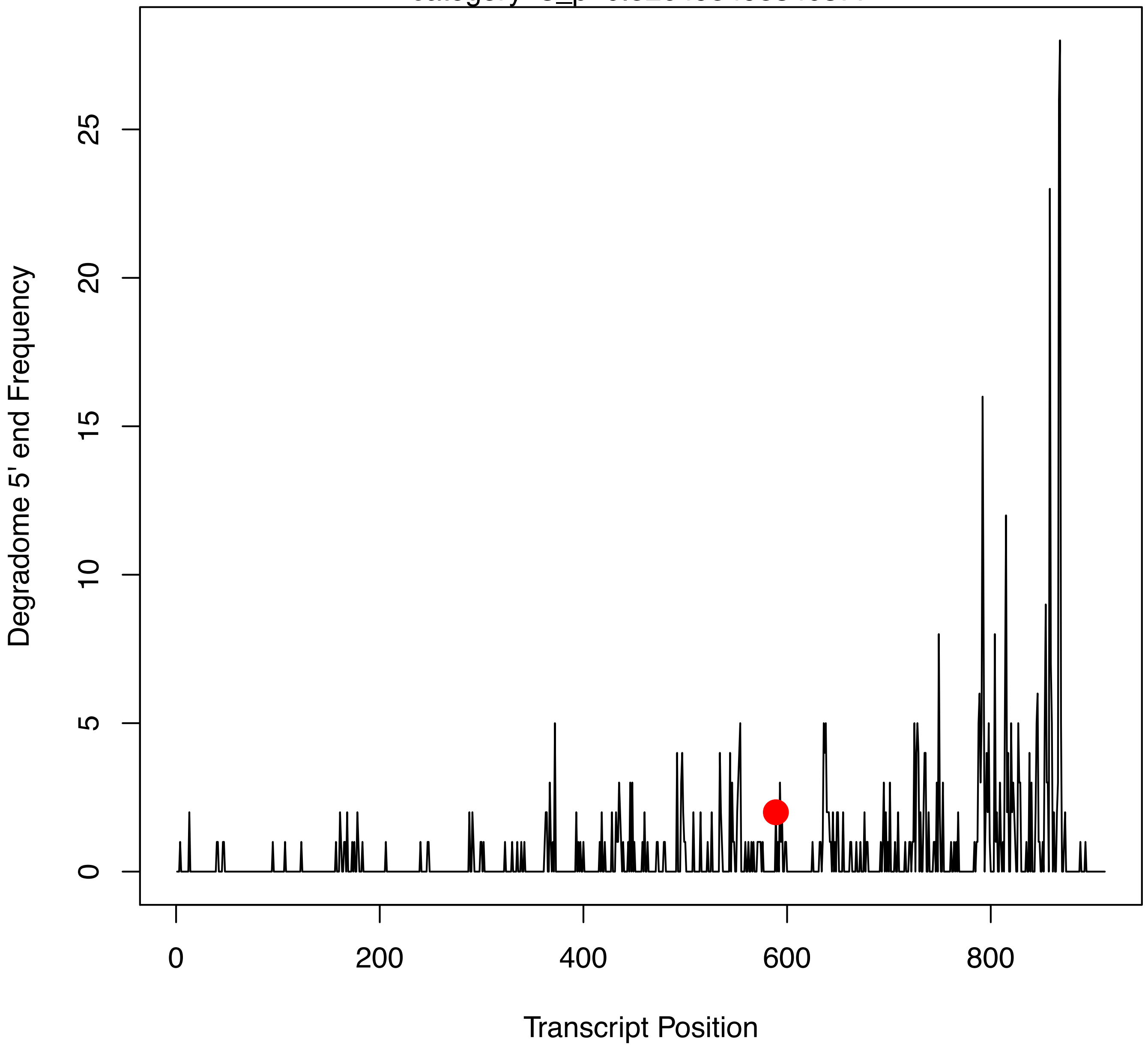

**T=chr8.gff3_MRNA_VIT_08s0007g01630.t01_Q=I miRC129 _S=334**

category=3_p=0.758020585066602

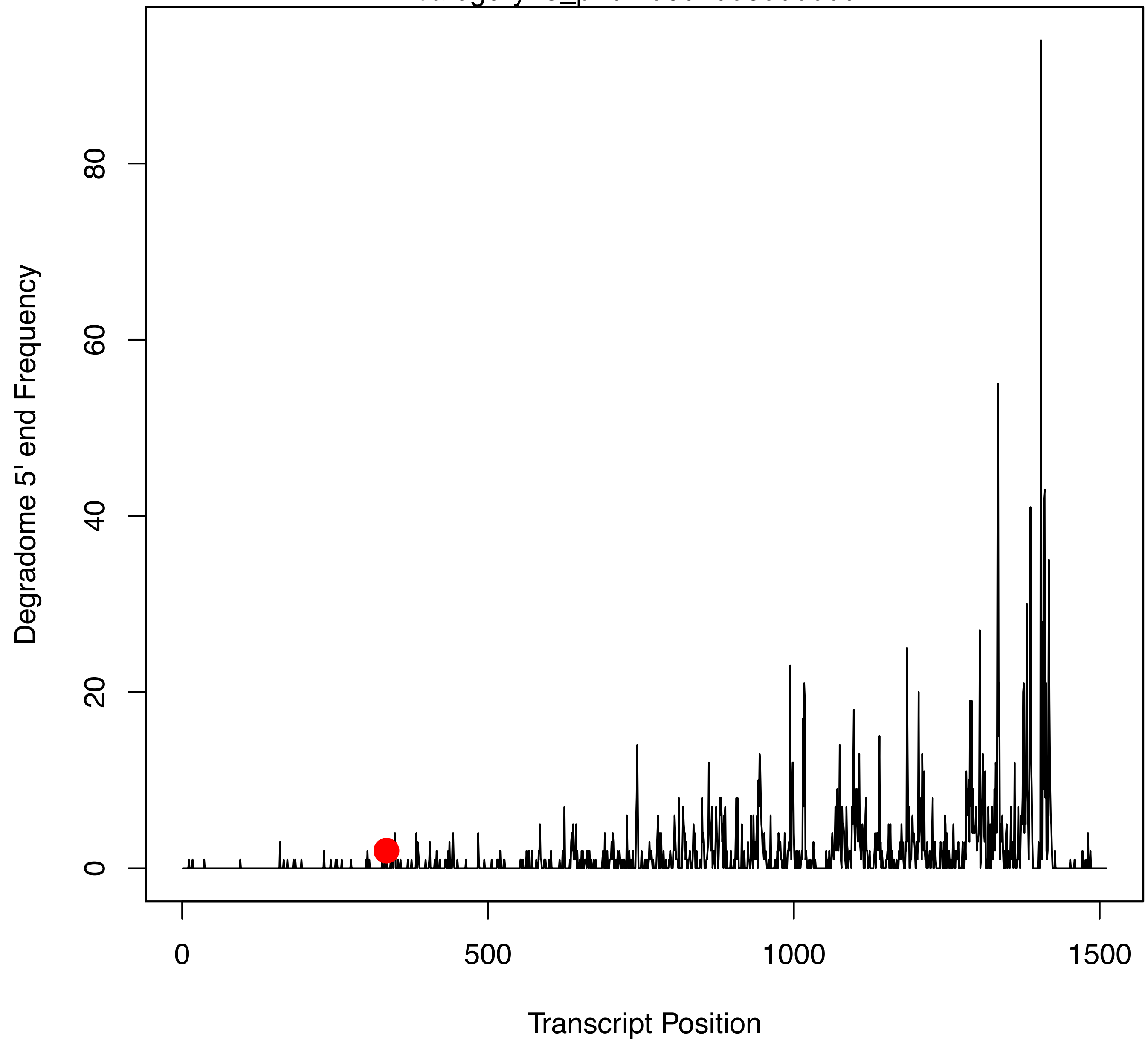

**T=chr14.gff3_MRNA_VIT_14s0066g01320.t01_Q= miRC129 _S=162**

category=3_p=0.932313164541245

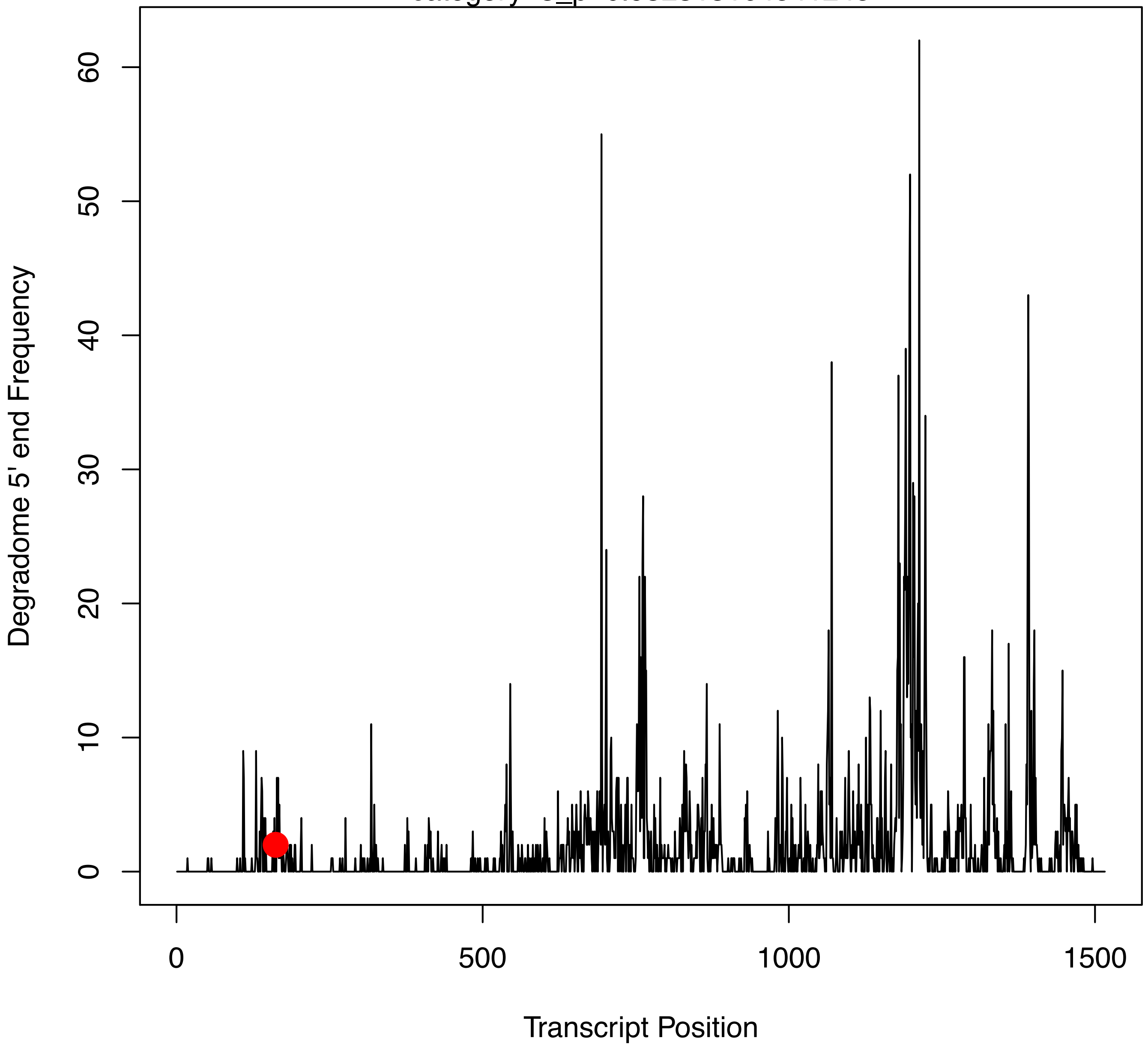

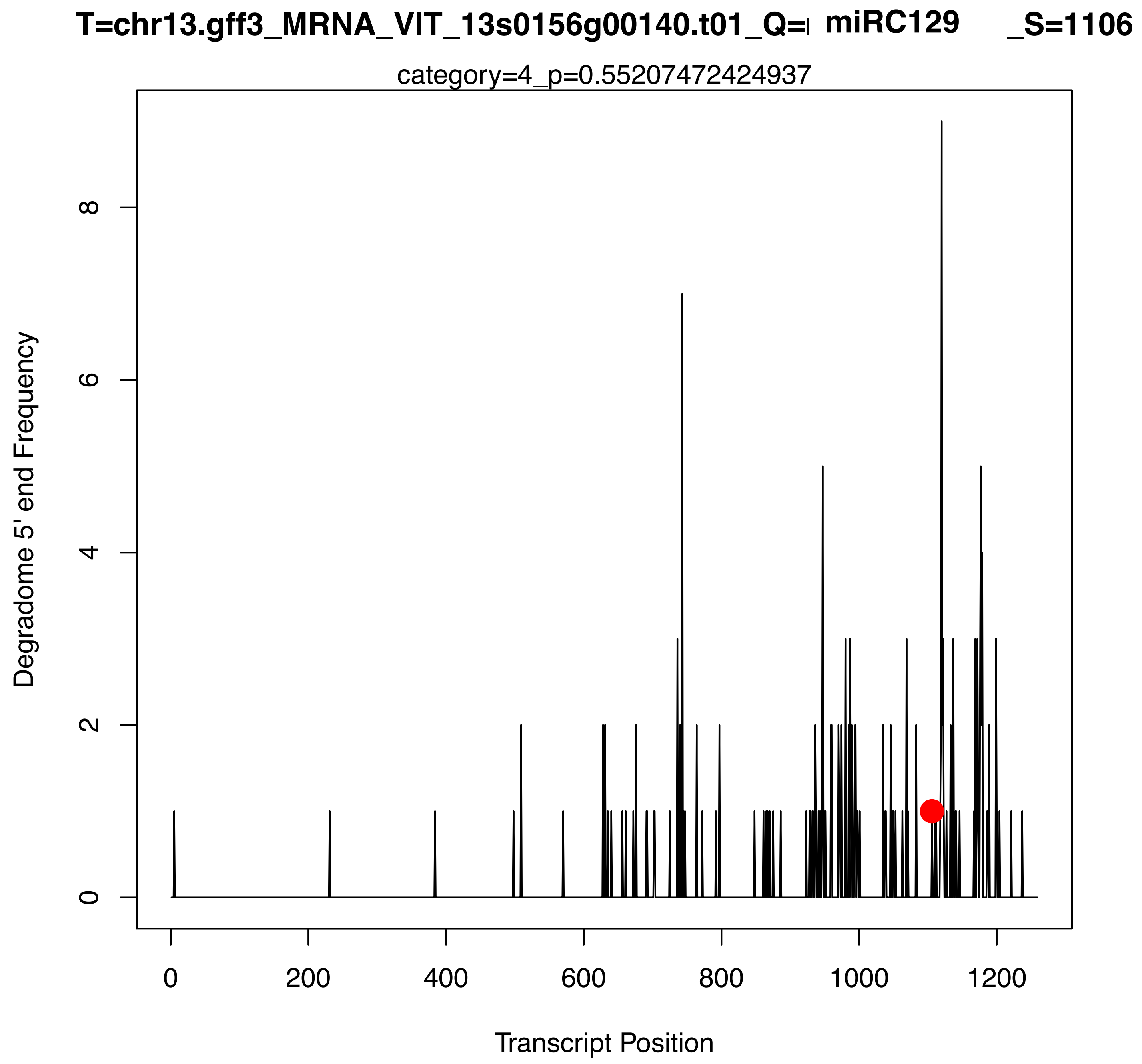
T=chr13.gff3_MRNA_VIT_13s0156g00140.t01_Q= miRC129 _S=1106
category=4_p=0.55207472424937
Degradome 5' end Frequency
0
2
4
6
8
0
200
400
600
800
1000
1200
Transcript Position

T=chr4.gff3_MRNA_VIT_04s0008g05800.t01_Q= miRC129 _S=2997

category=4_p=0.56520228057076

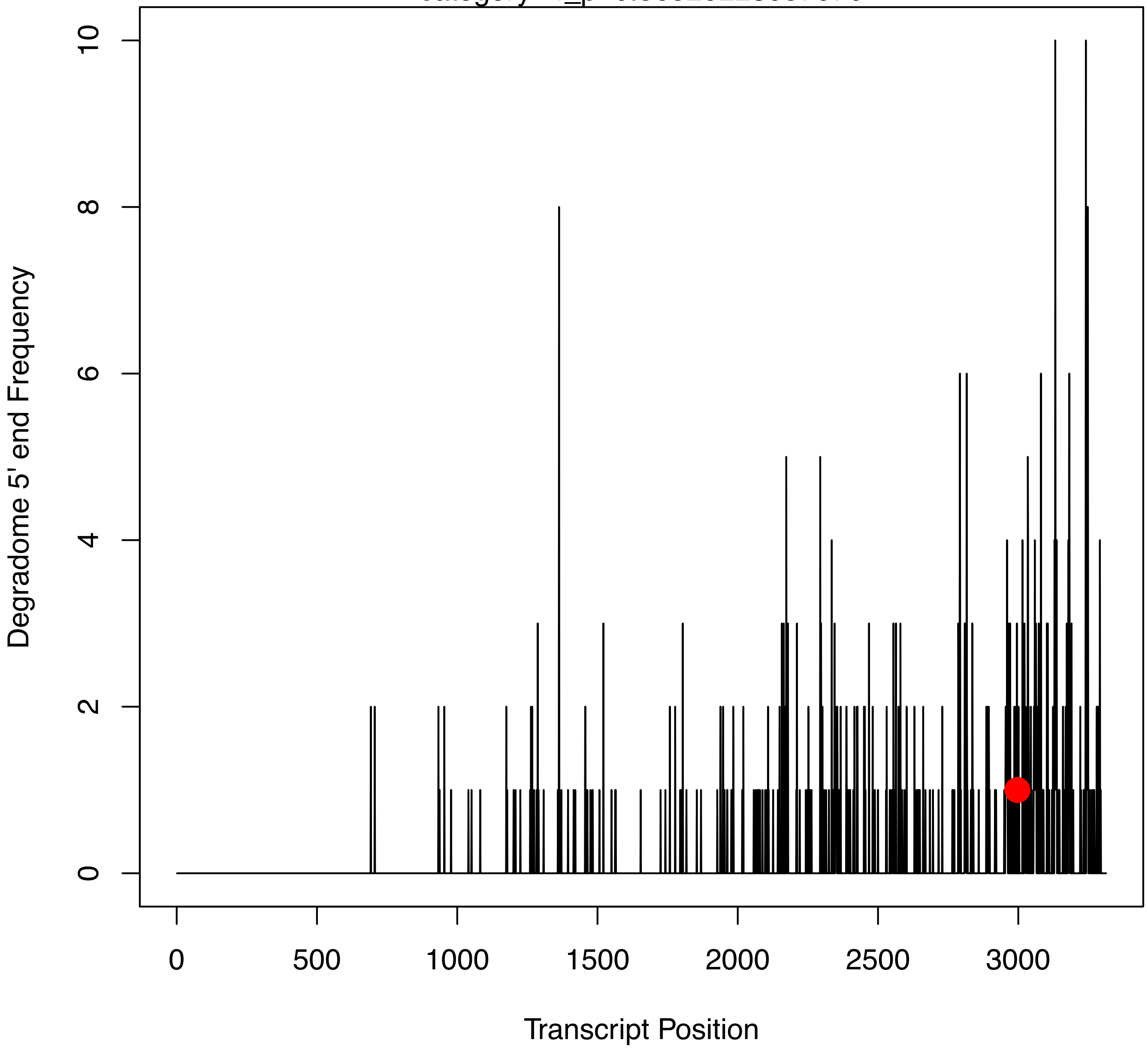

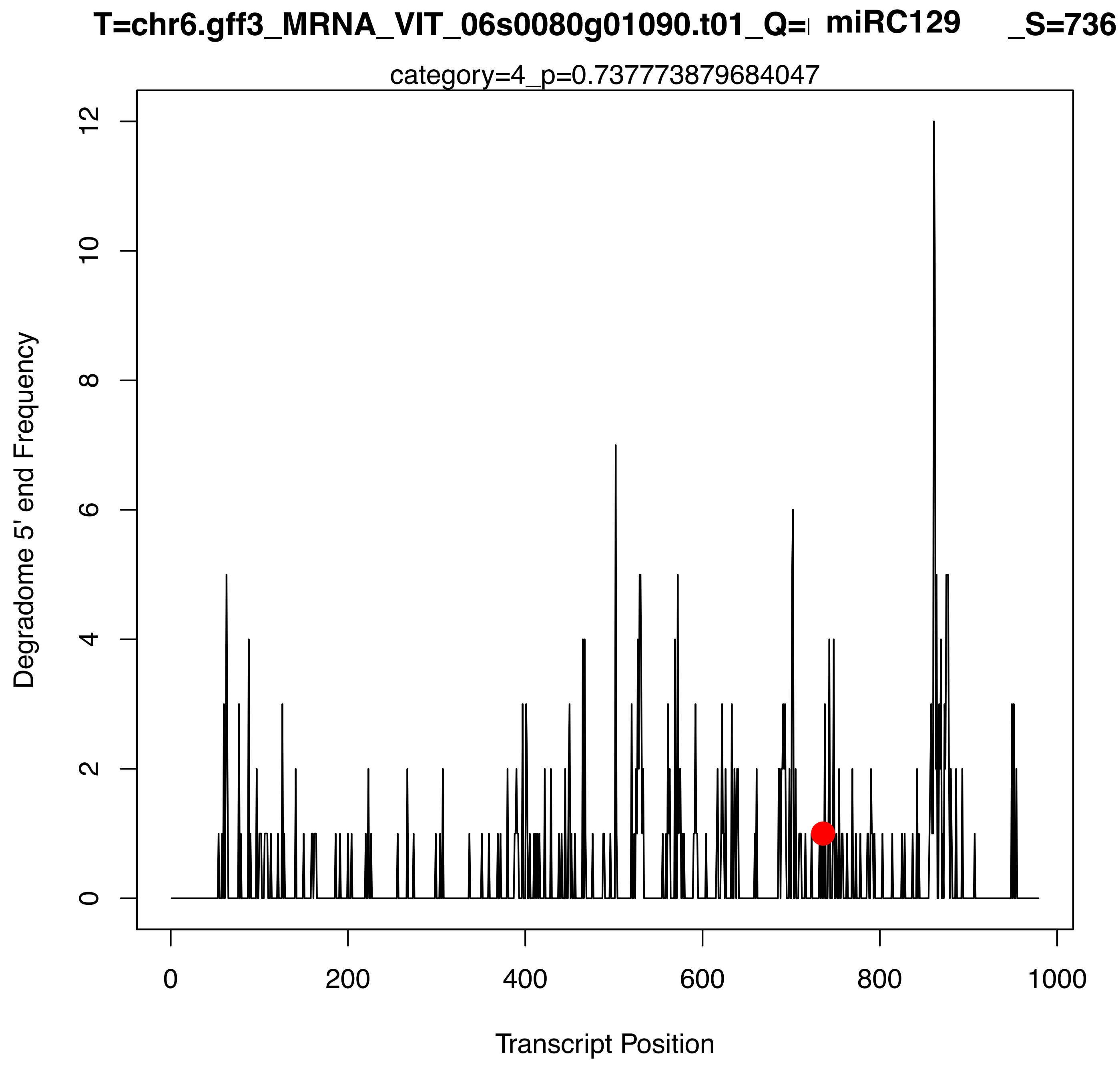
T=chr6.gff3_MRNA_VIT_06s0080g01090.t01_Q=miRC129_S=736
category=4_p=0.737773879684047
Degradome 5' end Frequency
0
2
4
6
8
10
12
0
200
400
600
800
1000
Transcript Position

**T=chrUn.gff3_MRNA_VIT_00s0391g00070.t01_Q=miRC129 _S=1644**

category=4_p=0.793305247196719

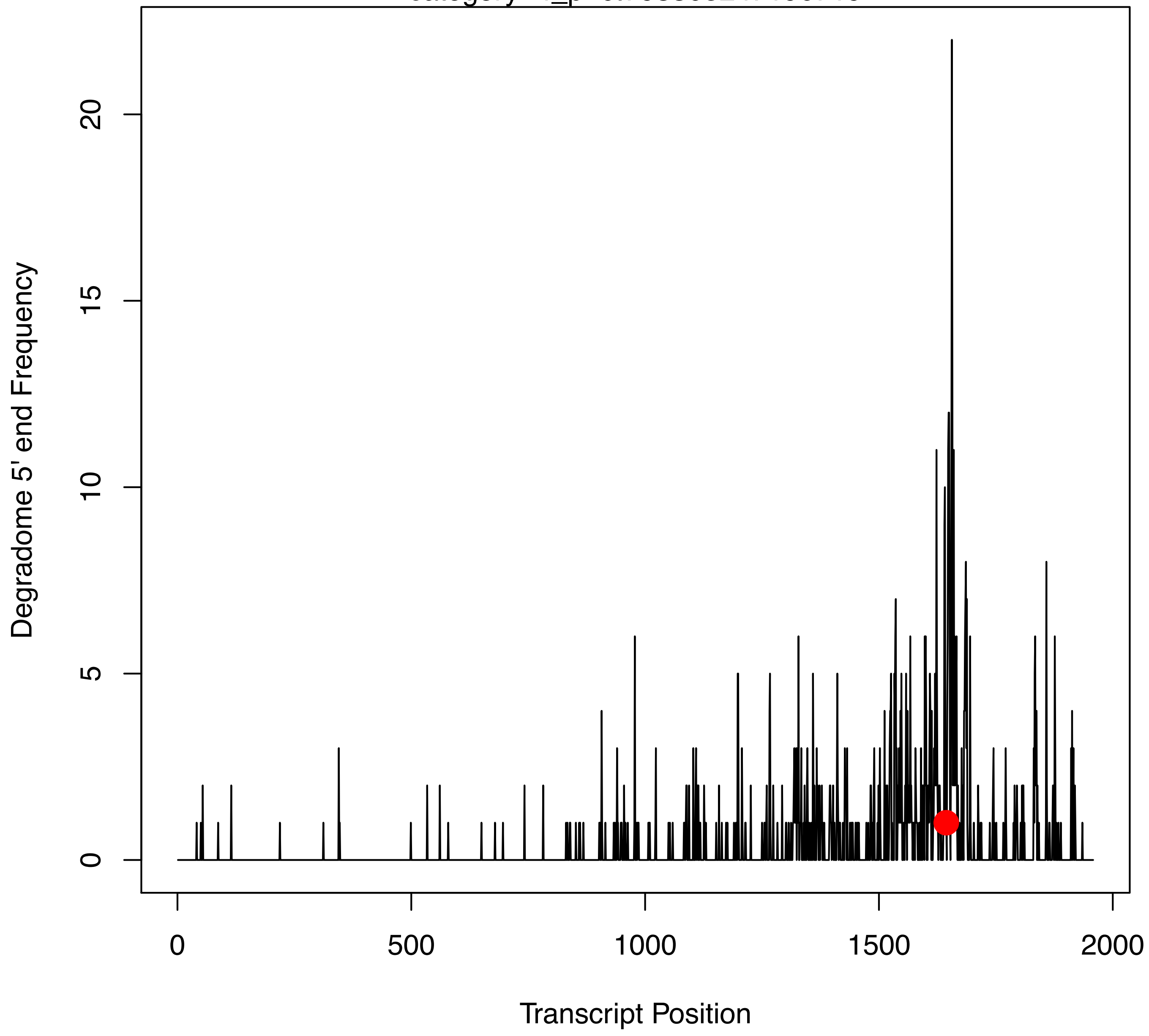

**T=chr4.gff3_MRNA_VIT_04s0023g02720.t01_Q= miRC129 _S=185**

category=4_p=0.821869662598727

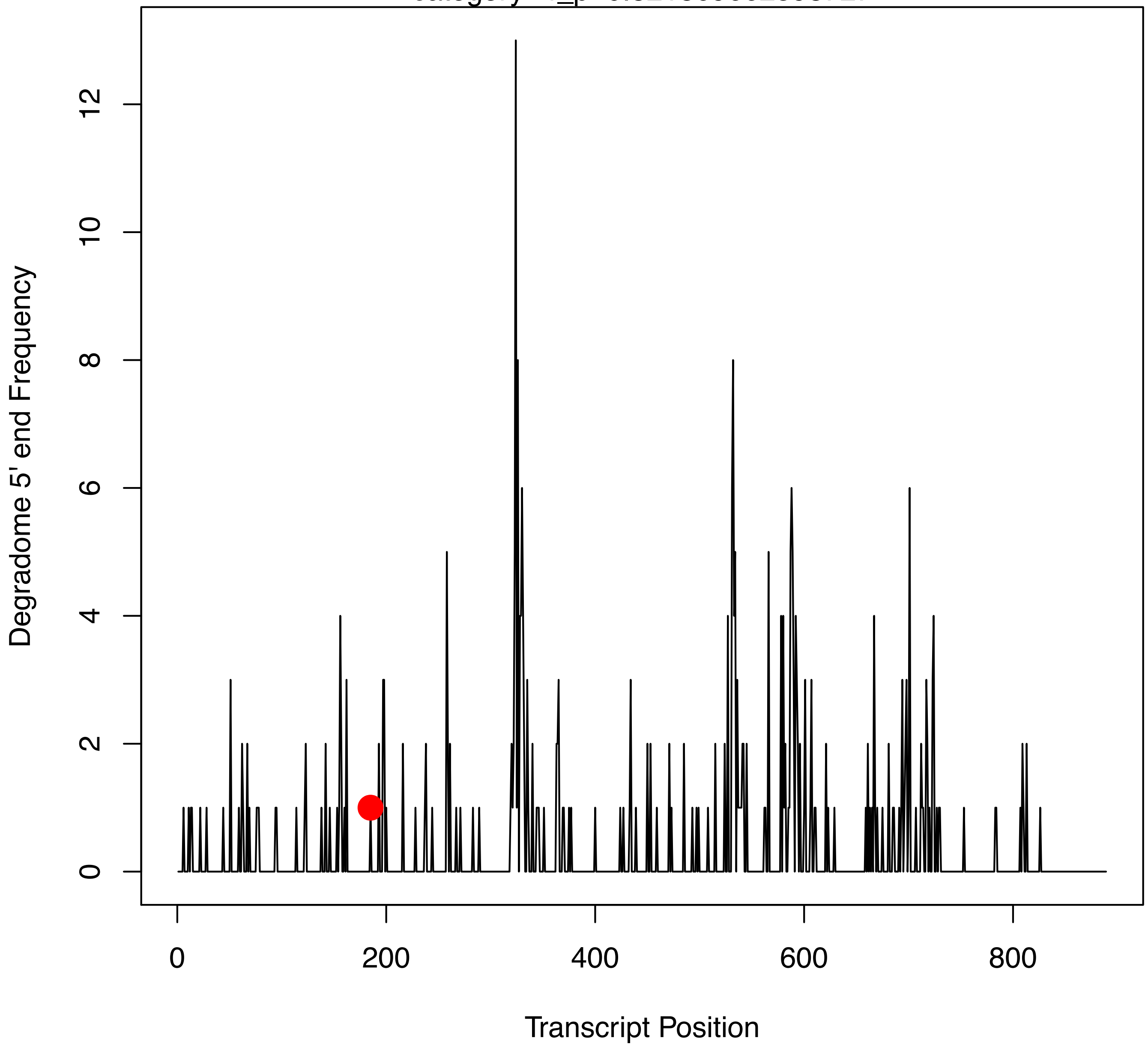

**T=chr14.gff3_MRNA_VIT_14s0066g00220.t01_Q=_miRC129_S=1199**

category=4_p=0.979077218976903

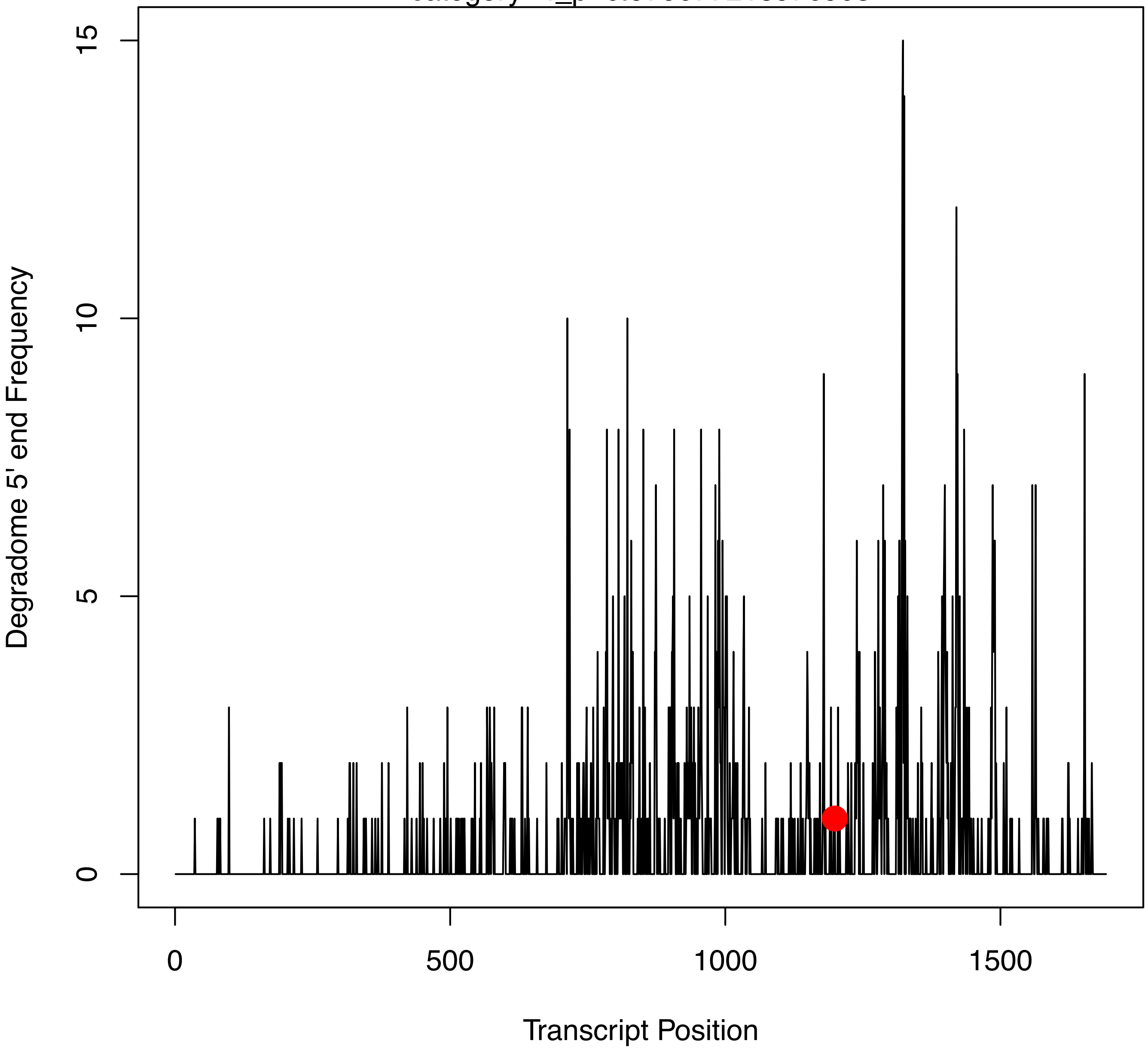

T=chr12.gff3_MRNA_VIT_12s0134g00400.t01_Q= miRC129 _S=53

category=4_p=0.984915949586867

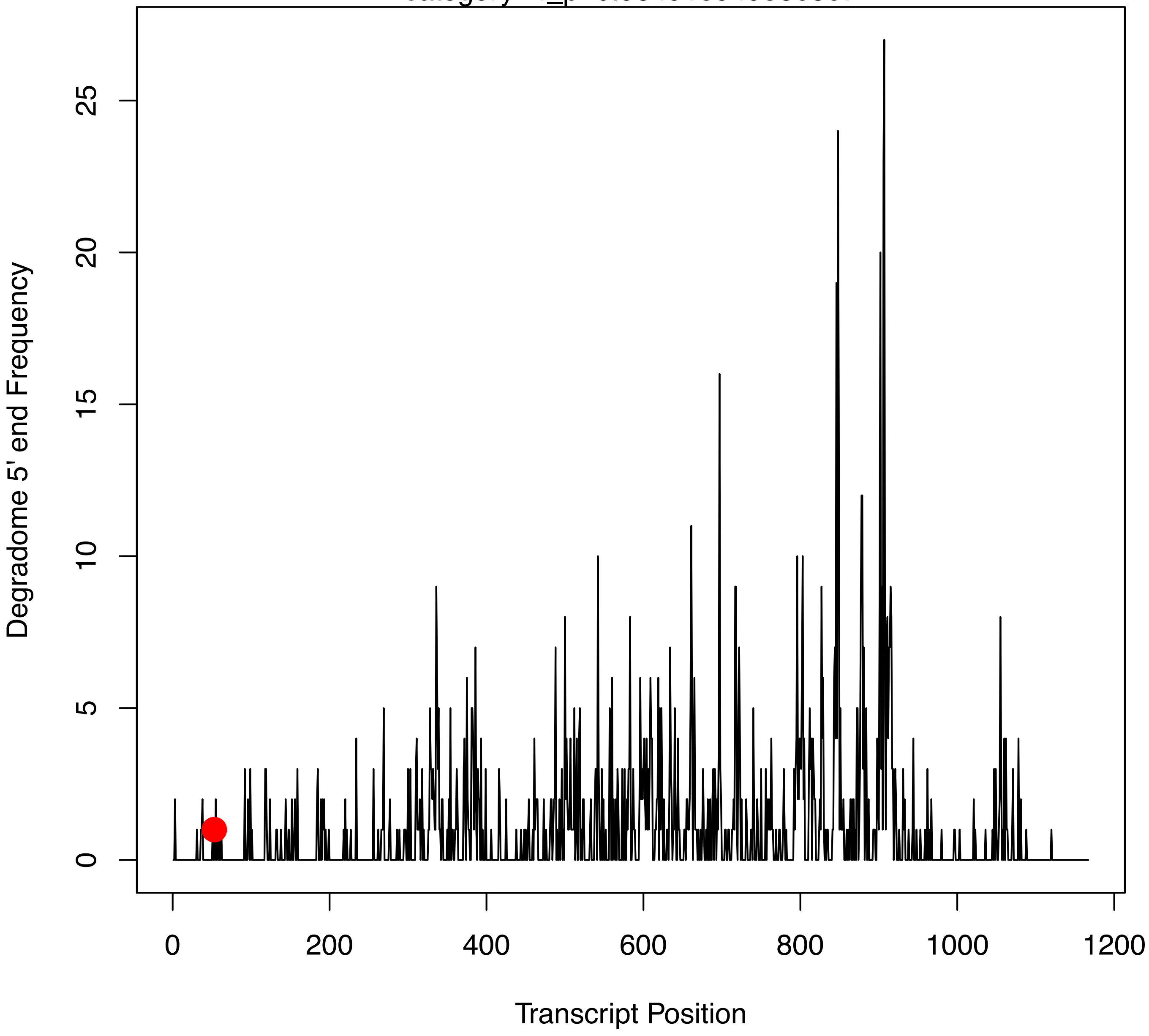

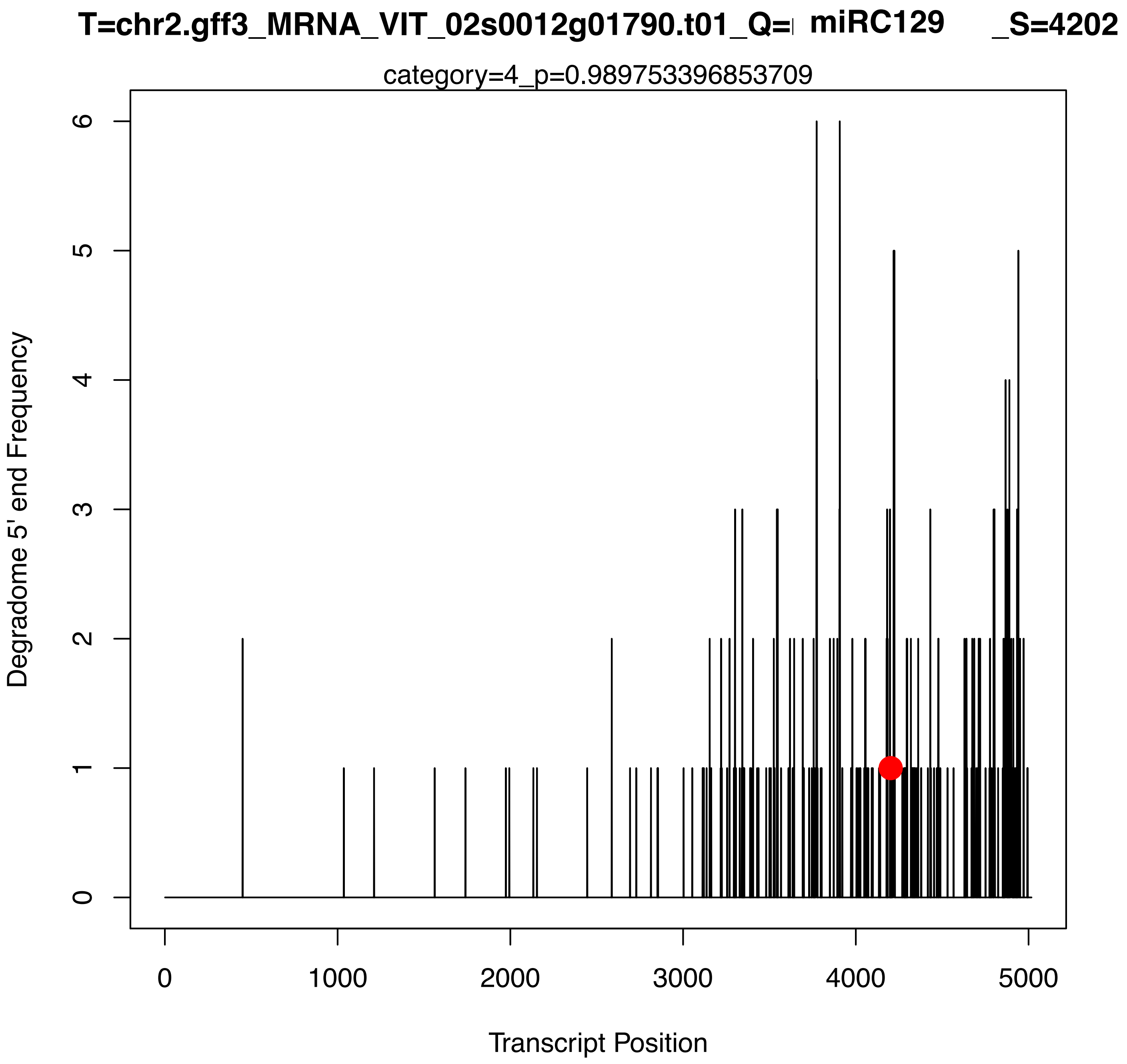
T=chr2.gff3_MRNA_VIT_02s0012g01790.t01_Q= miRC129 _S=4202
category=4_p=0.989753396853709
Degradome 5' end Frequency
0
1
2
3
4
5
6
Transcript Position
0
1000
2000
3000
4000
5000

T=chr10.gff3_MRNA_VIT_10s0071g00270.t01_Q= miRC129 _S=491

category=4_p=0.99238978732271

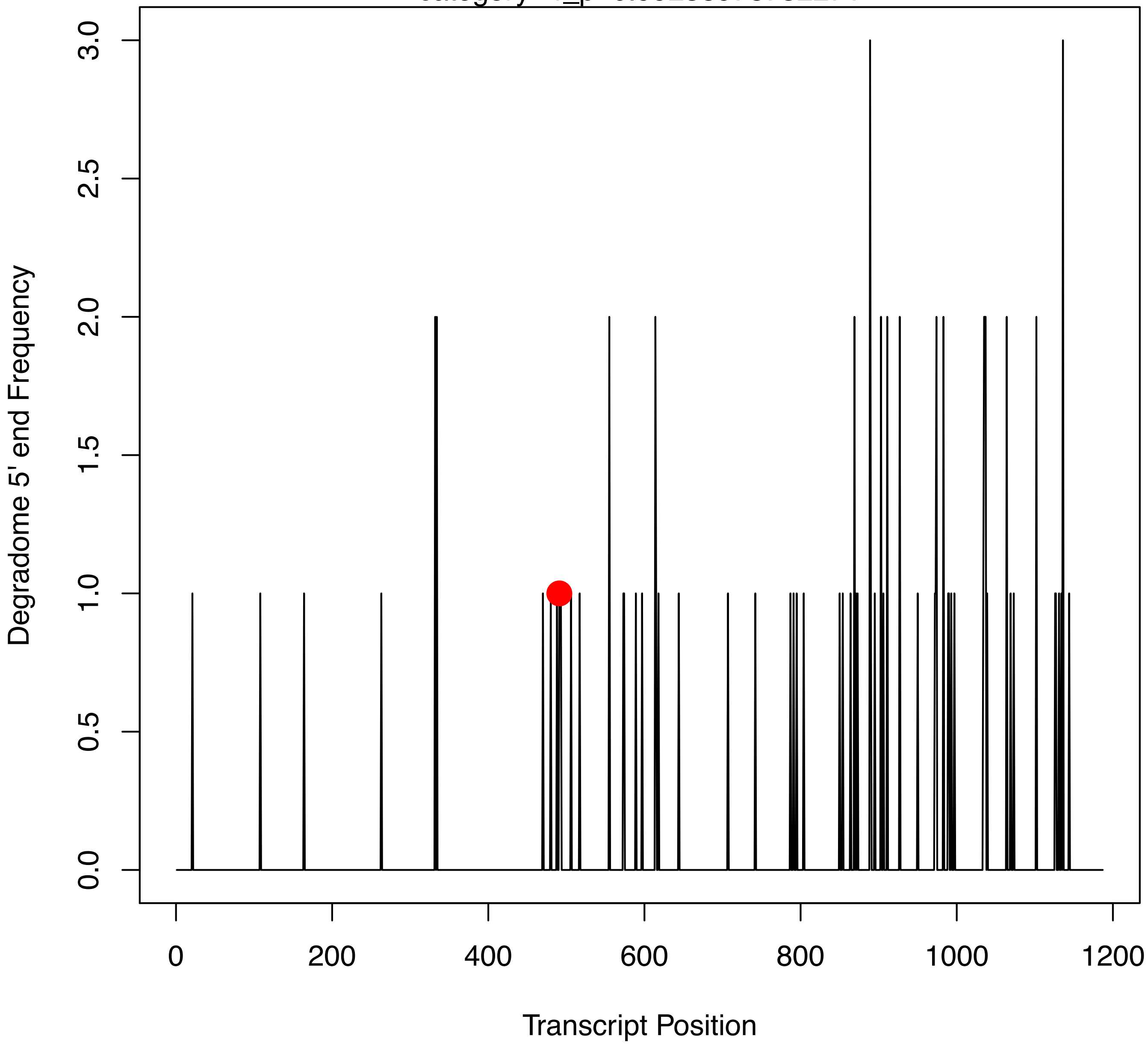

**T=chrUn.gff3_MRNA_VIT_00s2269g00010.t01_Q= miRC129 _S=1149**

category=4_p=0.993633702632962

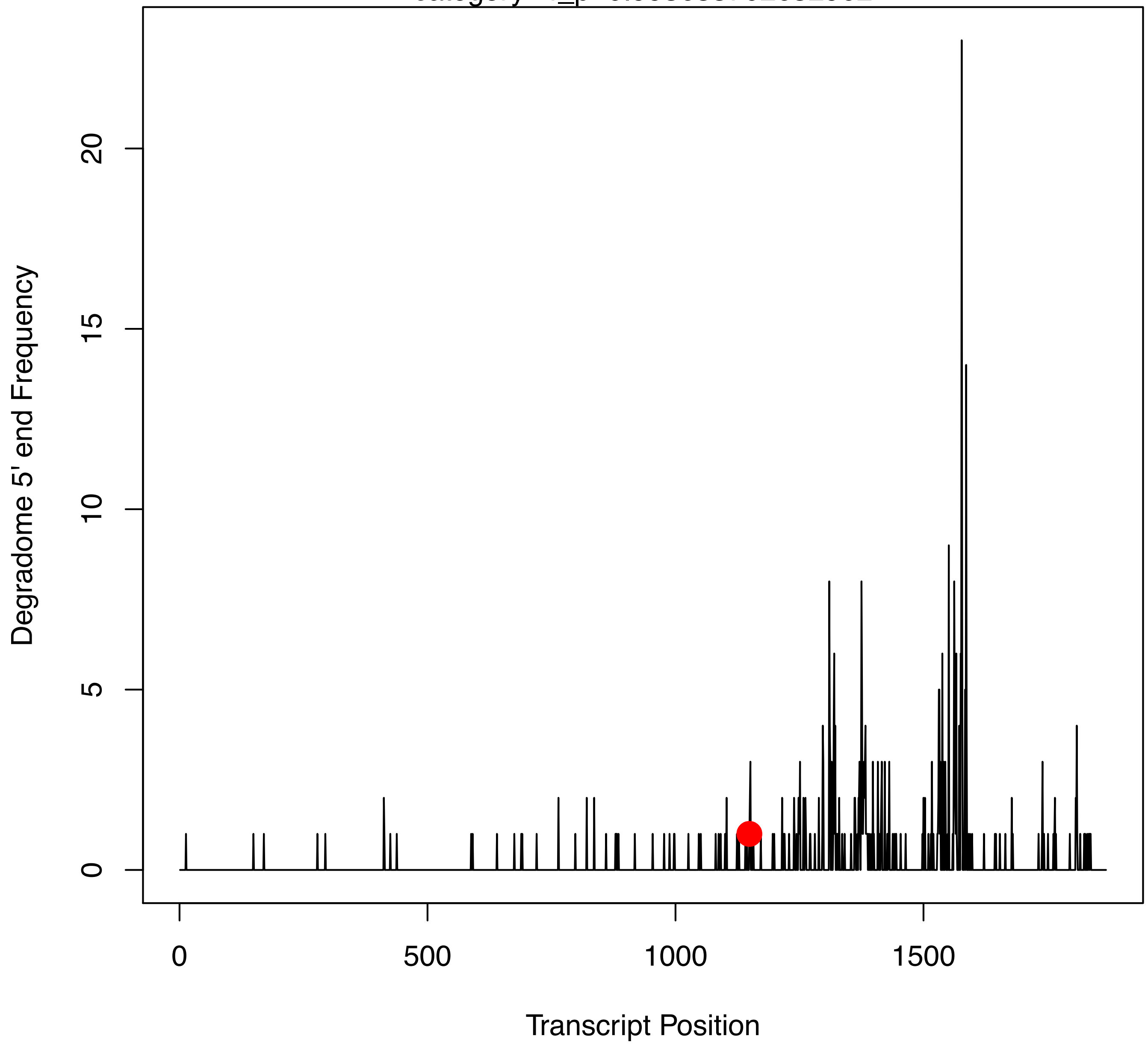

**T=chr19.gff3_MRNA_VIT_19s0090g01070.t01_Q= miRC129 _S=1228**

category=4_p=0.999051330501146

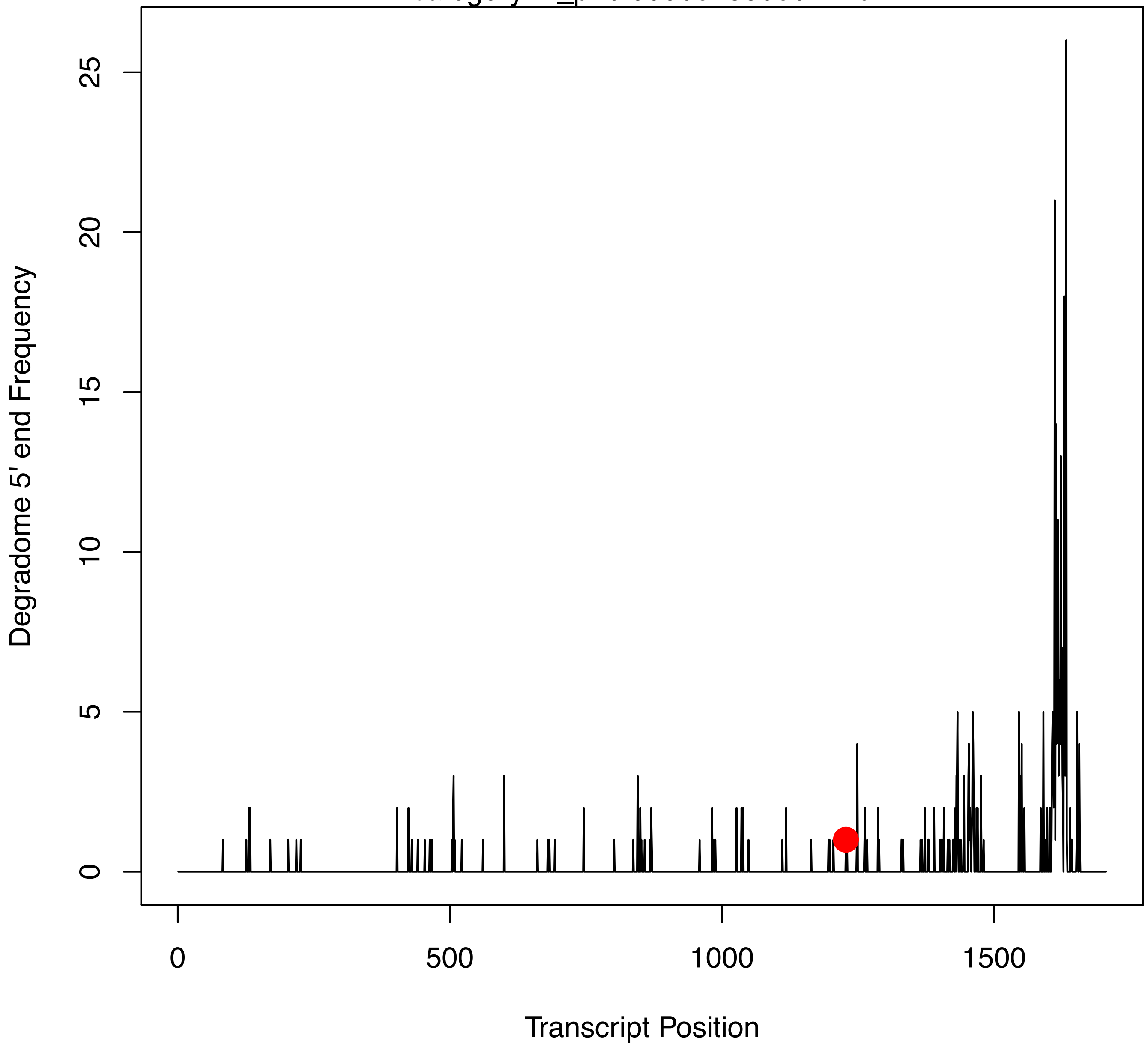

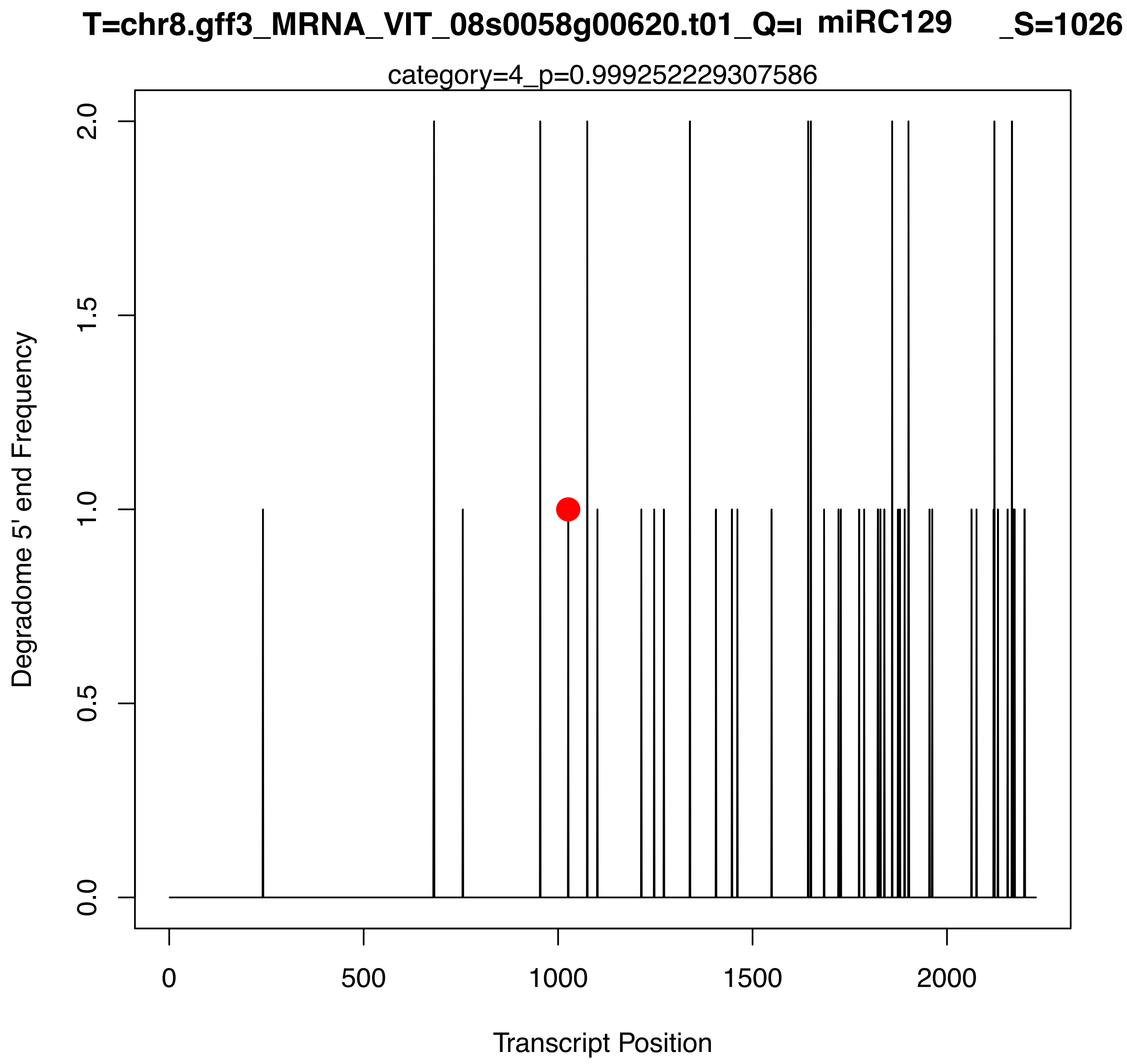
T=chr8.gff3_MRNA_VIT_08s0058g00620.t01_Q=ı miRC129 _S=1026
category=4_p=0.999252229307586
Degradome 5' end Frequency
0.0
0.5
1.0
1.5
2.0
0
500
1000
1500
2000
Transcript Position

**T=chr17.gff3_MRNA_VIT_17s0000g02840.t01_Q= miRC129 _S=725**

category=4_p=0.999791895719301

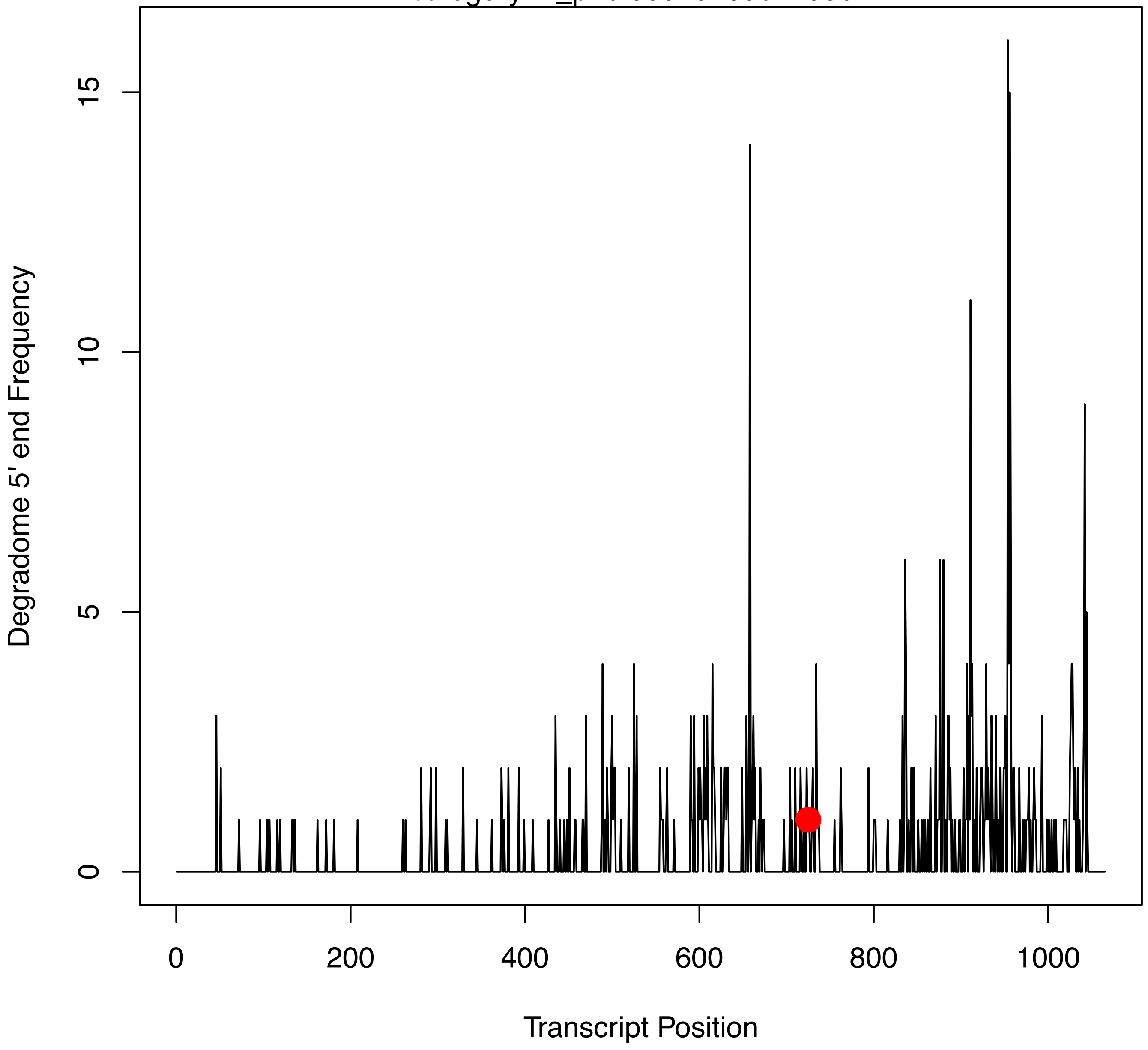

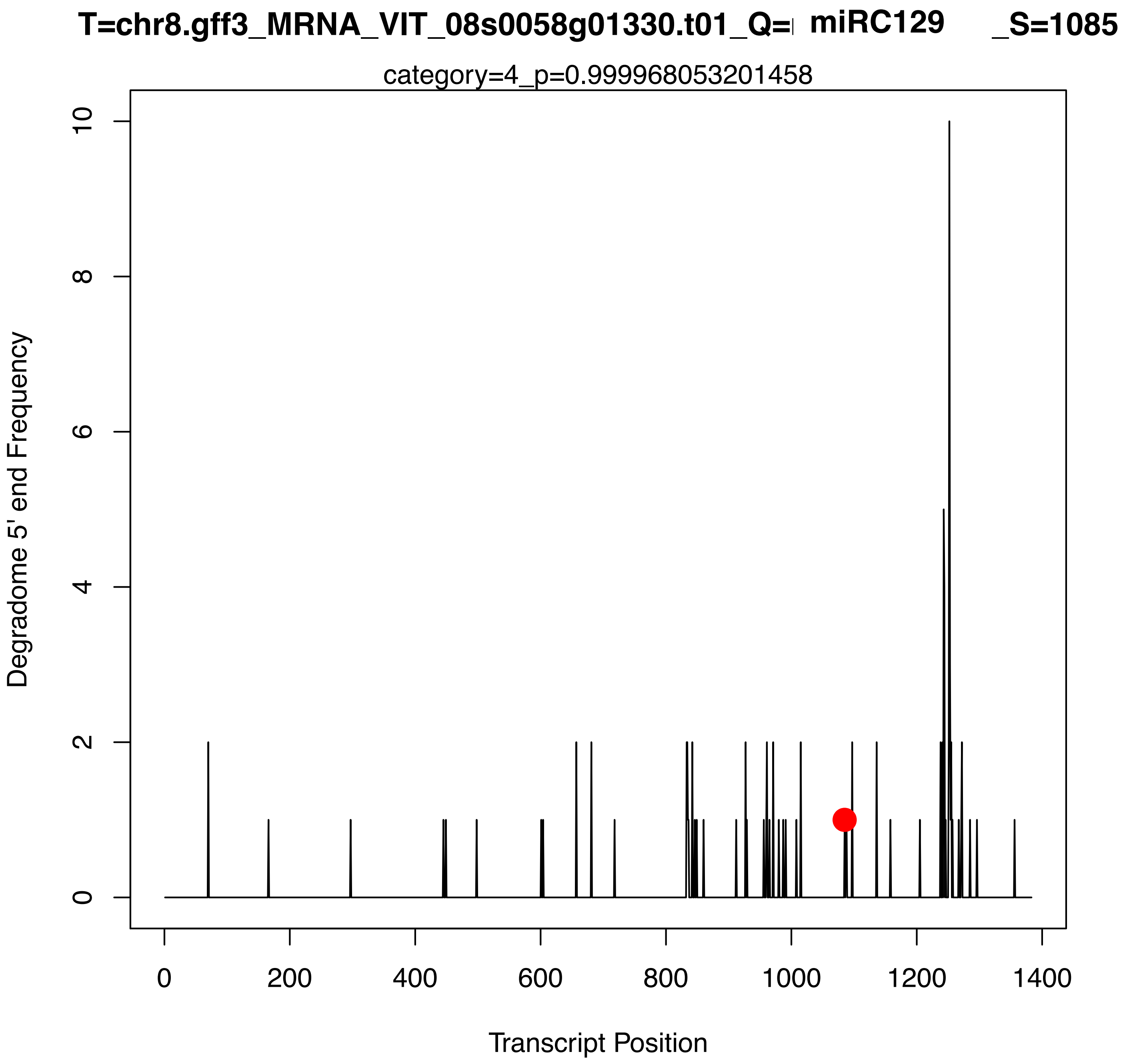
T=chr8.gff3_MRNA_VIT_08s0058g01330.t01_Q= miRC129 _S=1085
category=4_p=0.999968053201458
Degradome 5' end Frequency
0
2
4
6
8
10
0
200
400
600
800
1000
1200
1400
Transcript Position

**T=chr5.gff3_MRNA_VIT_05s0020g02420.t01_Q=ı miRC129 _S=1523**

category=4_p=0.999994309305997

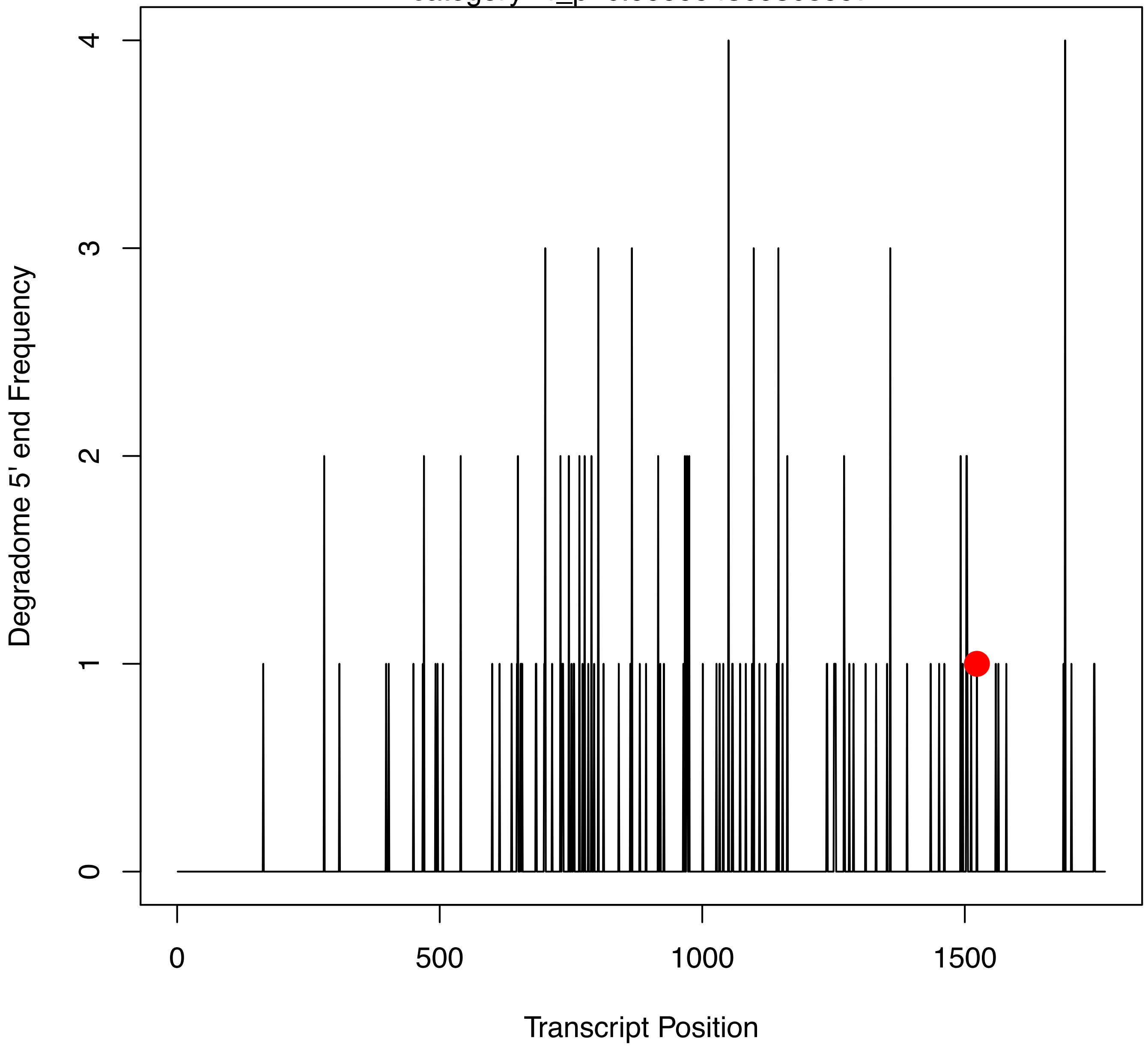

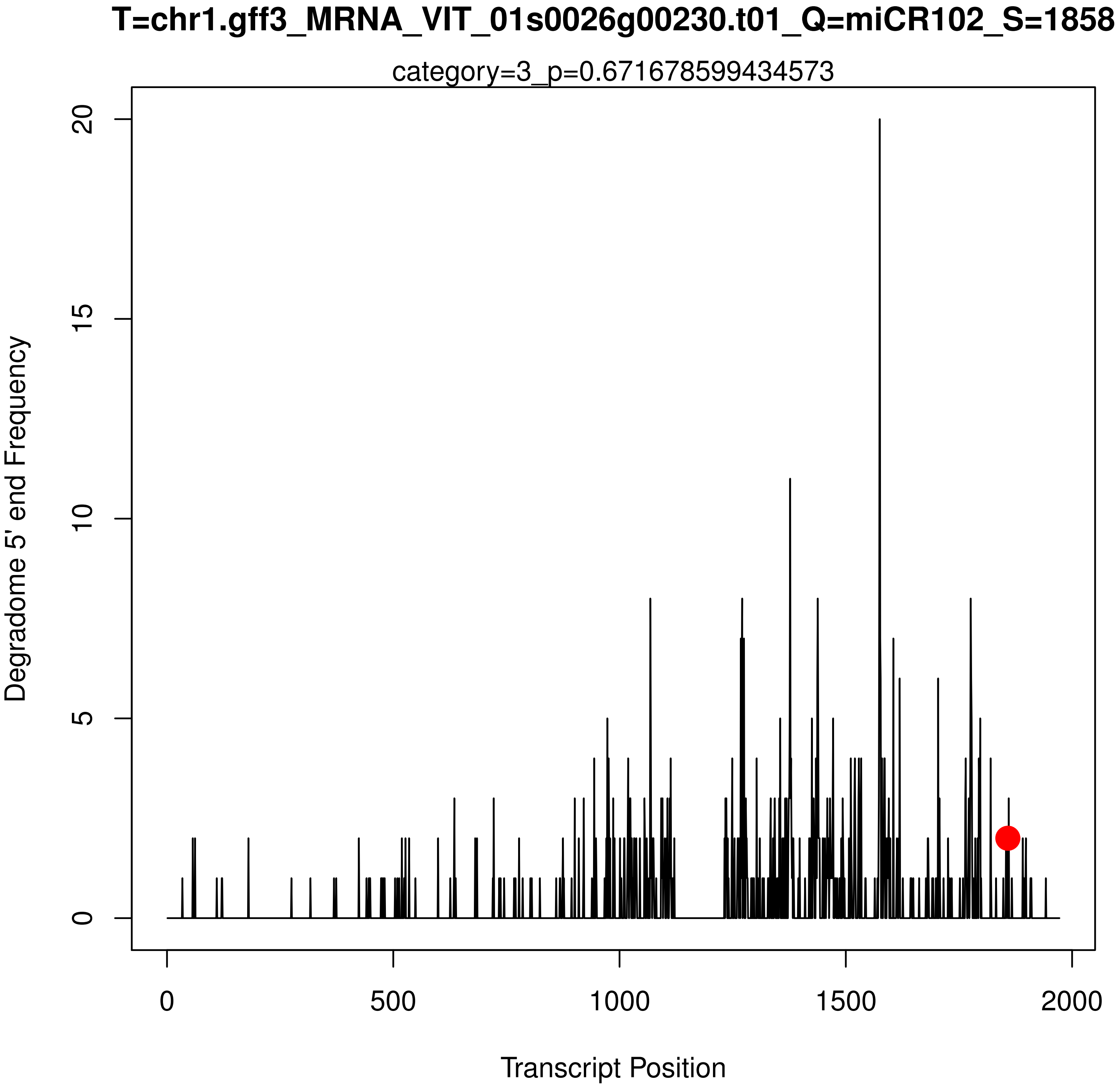
T=chr1.gff3_MRNA_VIT_01s0026g00230.t01_Q=miCR102_S=1858
category=3_p=0.671678599434573
Degradome 5' end Frequency
0
5
10
15
20
0
500
1000
1500
2000
Transcript Position

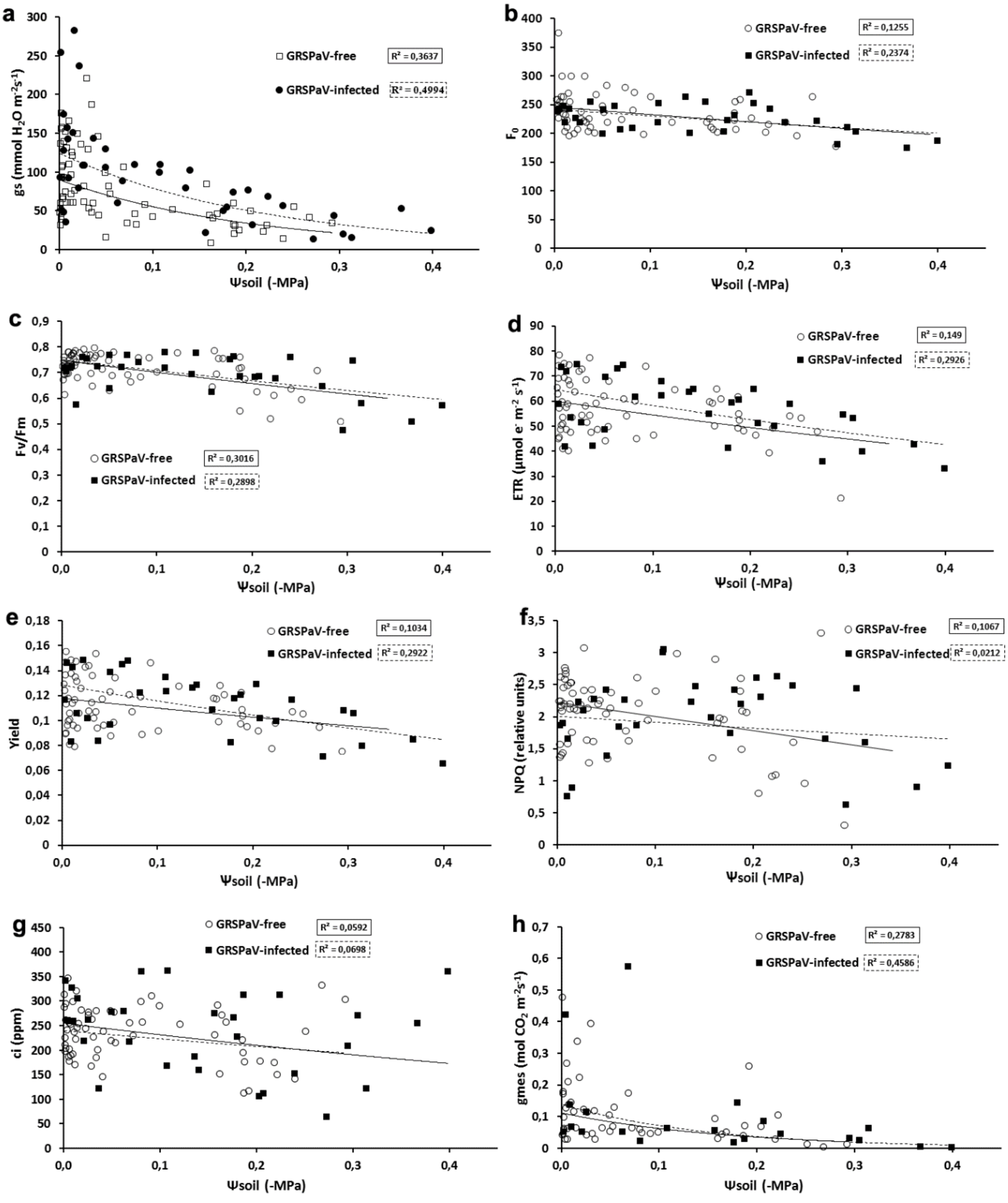


**Supplementary Figure S10**. Relationship between soil water potential (Ψsoil) and: a) estimated stomatal conductance ($g_s$), b) maximum fluorescence in dark adapted leaves (F0), c) maximum quantum efficiency of PSII (Fv/Fm), d) electron transport rate (ETR), e) quantum efficiency of PSII (Yield), f) non-photochemical quenching (NPQ), g) substomatal internal carbon concentration (ci) and h) mesophyll conductance to $CO_2$ (gmes) in *Grapevine rupestris stem pitting-associated* (GRSPaV)-infected (filled squares, dashed trend line) and GRSPaV-free (open circles, continuous trend line) plants.
